# Identification of immunotherapy-related subtypes, characterization of tumor microenvironment infiltration, and development of a prognostic signature in gastric carcinoma

**DOI:** 10.18632/aging.205968

**Published:** 2024-06-25

**Authors:** Jianxin Li, Ting Han, Jieyi Yang, Xin Wang, Yinchun Wang, Rui Yang, Qingqiang Yang

**Affiliations:** 1 Department of General Surgery (Gastrointestinal Surgery), The Affiliated Hospital of Southwest Medical University, Luzhou 646000, Sichuan, P.R. China

**Keywords:** gastric carcinoma, immunotherapy, immune, signature, prognosis

## Abstract

Background: Recent advances in immunotherapy have elicited a considerable amount of attention as viable therapeutic options for several cancer types, the present study aimed to explore the immunotherapy-related genes (IRGs) and develop a prognostic risk signature in gastric carcinoma (GC) based on these genes.

Methods: IRGs were identified by comparing immunotherapy responders and non-responders in GC. Then, GC patients were divided into distinct subtypes by unsupervised clustering method based on IRGs, and the differences in immune characteristics and prognostic stratification between these subtypes were analyzed. An immunotherapy-related risk score (IRRS) signature was developed and validated for risk classification and prognosis prediction based on The Cancer Genome Atlas (TCGA) and Gene Expression Omnibus (GEO) cohorts. Besides, the predictive ability of the IRRS in immunotherapy response was also determined.

Results: A total of 63 IRGs were identified, and 371 GC patients were stratified into two molecular subgroups with significantly different prognosis and immune characteristics. Then, an IRRS signature comprised of three IRGs (CENP8, NRP1, and SERPINE1) was constructed to predict the prognosis of GC patients in TCGA cohort. Importantly, external validation in multiple GEO cohorts further confirmed the universal applicability of the IRRS in distinct populations. Furthermore, we found that the IRRS was significantly correlated with patient’s responsiveness to immunotherapy, GC patients with low IRRS are more likely to benefit from existing immunotherapy.

Conclusions: The risk score could serve as a robust prognostic biomarker, provide therapeutic benefits for immunotherapy and may be helpful for clinical decision making in GC patients.

## INTRODUCTION

Gastric carcinoma (GC) is one of the most frequently diagnosed digestive system malignant tumor that ranks fifth for morbidity and fourth for tumor-related mortality around the world [1]. The prognosis of GC patients is closely correlated with the pathological stage of the tumor, the 5-year survival rate for patients diagnosed with advanced pathological stage or distant metastatic carcinoma declines to approximately 5% because of missed the opportunity to be subjected to surgical treatment, and the median survival of those who did not receive adjuvant therapy is less than 1 year [2, 3]. However, a proportion of GC patients are diagnosed with advanced stage when first visit, which is responsible for the poor prognosis [4]. Besides, some patients will suffer relapse from micro-metastatic lesions that have disseminated at the time of surgery. More disconcertingly, because the heterogeneity in individuals is great, only a subset of GC patients is sensitive to non-surgical management, including chemotherapy, radiotherapy, and targeted therapy, and many who initially respond develop resistance over time [5]. The traditional risk classification is mainly dependent on the tumor, lymph node, metastasis (TNM) staging system, which ignores the great heterogeneity of the primary tumor. Thus, exploring novel biomarkers to accurately forecast the progression and prognosis of GC is urgently needed.

Nowadays, immunotherapeutic techniques have achieved great success as anti-cancer therapy for many types of malignant tumors through reactivating host immunity against tumor cells [6]. It is widely accepted that tumor microenvironment (TME) act as a decisive role in initiation and progression of malignant tumors. Immune dysfunction of the host significantly impaired the body’s anti-tumor immunological surveillance, which lead to the development of cancer [7]. Among the distinct developed immunotherapeutic strategies, immune checkpoint inhibitors (ICIs) targeting programmed death 1 (PD-1), programmed death ligand 1 (PD-L1) and cytotoxic T lymphocyte-associated antigen-4 (CTLA–4) have shown promising and durable clinical responses in several solid tumors [8]. PD-1 is a common immunosuppressive member on the membrane of immune cell, especially the activated T cell. PD-L1 is the main ligand of PD-1, which physiologically inhibit excessive immune responses by activating PD-1 to prevent normal tissue from damage [9]. However, this protective mechanism is disrupted in cancer-immunity cycle, where the overexpressed PD-L1 binds to PD-1, inhibits the activation of T cell including through induction of T cell apoptosis, thereby inhibiting the anti-cancer immunity [10]. Immunotherapy blocking the PD-1/PD-L1 regulation axis can efficaciously inhibit its tumor-promoting activity, and the inhibitors of PD-1/PD-L1 have shown clinical efficacy in many tumors over the past decade. For example, pembrolizumab, a humanized IgG4 kappa anti-PD-1 antibody, was approved by the Food and Drug Administration (FDA) for treating metastatic melanoma and advanced urothelial carcinoma [11]. Anti-PD-L1 antibodies, atezolizumab and durvalumab, have been approved for treating non-small-cell lung cancer as first-line/second-line treatment strategies [12]. In terms of GC, recent advance in understanding the TME of cancer has significantly facilitated the development of immunotherapy for advanced GC. National Comprehensive Cancer Network (NCCN) guidelines recommend pembrolizumab plus trastuzumab for first-line treatment of HER-2 positive metastatic GC based on the results of KEYNOTE-811 phase III trial [13]. In addition, immunotherapy has been included in the first-line/second-line treatment of GC in 2021 by the Chinese Society of Clinical Oncology (CSCO) [14]. On the contrary, a phase III trial (KEYNOTE 062) found that chemotherapy combined with ICIs cannot improve the overall survival (OS) of patients with advanced GC compared with chemotherapy alone [15]. Currently, biomarkers used to identify anti-PD-1/PD-L1 therapy responders mainly include microsatellite instability and PD-L1 expression, with only a subset of patients showing clinical responses. Moreover, many who initially respond develop resistance over time. Therefore, the development of novel molecular biomarkers to forecast clinical responses and identify suitable patients who benefit from anti-cancer immunotherapy is of great clinical significance.

The classification of patients by applying bioinformatic analysis based on next-generation sequencing is a novel strategy that can identify the immune characteristics and predict the prognosis of tumors, and some studies have been launched to acquire a better understanding of the TME and immunotherapeutic responsiveness of GC. However, there has been no appropriate research that construct a prognostic model to predict the survival outcomes and immune characteristics of GC based on immunotherapy-related genes (IRGs). Besides, clinical application of previously identified signatures is limited due to lack of validation with external dataset. In this study, we first identified IRGs in GC through analyzing immunotherapy cohort. Subsequently, we used unsupervised clustering method to identify immunotherapy-related subtypes and compared the differences in TME and immunotherapy response between the two clusters. Finally, a three-gene signature for GC was developed to predict prognosis and immunotherapy responsiveness. Importantly, its predictive abilities for prognosis and treatment efficiency of immunotherapy were evaluated in distinct external validation cohorts.

## MATERIALS AND METHODS

### Data acquisition

The immunotherapy cohort of gastric carcinoma (PRJEB25780) was obtained from the Tumor Immune Dysfunction and Exclusion (TIDE, http://tide.dfci.harvard.edu/) database to identify immunotherapy-related genes [16, 17]. The RNA-sequencing profiles and corresponding clinicopathological information of GC were obtained from The Cancer Genome Atlas (TCGA, https://portal.gdc.cancer.gov/) [18]. Detailed information of somatic mutation and copy number variation (CNV) data files were retrieved for further analysis. The immunophenoscore (IPS) of GC samples was obtained from the Stomach Adenocarcinoma (STAD) project of The Cancer Immunome Atlas (TCIA, https://tcia.at/) [19]. Four cohorts (GSE15459 [20], GSE84437 [21], GSE62254 [22], and GSE26253 [23]) were collected from Gene Expression Omnibus (GEO, https://www.ncbi.nlm.nih.gov/geo/) to validate the predictive efficiency of the risk score signature in prognosis prediction [24]. IMvigor210 cohort includes RNA-sequencing profiles and detailed clinical parameters of urothelial carcinoma patients who received anti-PD-L1 therapy [25]. GSE78220 is an immunotherapy cohort where patients with melanoma were treated with anti-PD-1 agents [26]. We collected IMvigor210 and GSE78220 cohorts for evaluating the predictive efficiency of the risk score signature in immunotherapy response. The raw count data obtained from GEO database were normalized by applying the “limma” package in R [27].

### Identification of IRGs in GC

The IRGs were defined as genes that were differentially expressed between immunotherapeutic responder and non-responder groups. In our study, patients with GC in PRJEB25780 cohort were divided into responder and non-responder groups according to their responsiveness to anti-PD-1 immunotherapy. Then, the “limma” package in R was applied to screen out IRGs, the cutoff conditions were set to adjusted *P*-value < 0.05 and absolute value of log2 fold change (log2 FC) ≥ 0.585. Besides, we performed Kyoto Encyclopedia of Genes and Genomes (KEGG) pathway analysis to further clarify the potential functional annotation of the IRGs based on the Database for Annotation, Visualization and Integrated Discovery (DAVID, http://david.abcc.ncifcrf.gov/) database [28]. Somatic mutations and CNV are important events leading to genetic alterations. Thus, we first analyzed the overall genetic alteration atlas of the IRGs based on cBioPortal (https://www.cbioportal.org) platform [29]. Then, the detailed mutation and CNV information of the IRGs were analyzed.

### Consensus clustering analysis of IRGs

The “ConsensusClusterPlus” package with 1000 repetitions was used for performing unsupervised clustering analysis to identify distinct molecular subtypes in the TCGA cohort based on IRGs expression [30]. Then, differences in prognosis and clinicopathological parameters between subgroups were analyzed.

### Correlations between the subtypes and TME infiltration

The “CIBERSORT” algorithm was used to quantify the infiltrated abundances of 22 immunocytes for each GC patient with TCGA expression data [31]. The “ESTIMATE” algorithm was used to evaluate the purity of tumors from different clusters [32]. We compared the differences in immunocyte and immune score between different clusters to verify the immune characteristics of the IRG clusters. In addition, we applied a series of indexes which was usually used for predicting the response to immunotherapy, including tumor mutation burden (TMB), immune checkpoint biomarkers (ICBs), TIDE score, microsatellite instability and IPS, to assess the correlation between the subtypes and the effect of immunotherapy.

### Creating and confirming the predictive risk score signature

Differentially expressed genes (DEGs) between the immunotherapy-related subgroups were identified using the “limma” package in R, the threshold was set as adjusted *P*-value < 0.05 and absolute value of log2 FC ≥ 0.585. Similarly, gene ontology (GO) enrichment analysis and KEGG pathway enrichment analysis were conducted to clarify the pathways that were considerably enriched. Subsequently, univariate Cox and LASSO regression analyses were carried out to screen out the optimal prognostic biomarkers among these DEGs and included them in the immunotherapy-related risk score (IRRS) signature. The following formula can calculate the risk score of all cases: $RiskScore=\sum_{1}^{n}\text{Exp}(i)*Coef(i)$, where Exp is the expression value of the gene, and Coef is the LASSO regression analysis coefficient of each gene in the signature. Kaplan-Meier survival analysis was utilized to explore the predictive ability of the IRRS in GC prognosis. Area under the receiver operating characteristic (ROC) curve was utilized to evaluate the diagnostic efficacies. Univariate and multivariate Cox proportional hazards regression analyses were used to assess whether the signature could be served as an independent prognostic factor. Besides, the correlations between the IRRS and the clinicopathological parameters, including age, gender, grade, stage, and microsatellite instability, were determined using chi-square tests.

Importantly, the risk score was also calculated in four external cohorts (GSE15459, GSE84437, GSE62254, and GSE26253) to validate the predictive ability of the IRRS signature in distinct populations.

### Establishment of a nomogram

To enhance the clinical utility of the risk signature, a nomogram containing the IRRS and other independent prognostic factors was constructed using the “rms” package in R, and then was applied to predict the 1-, 3-, and 5-year survival rate of GC patients. ROC and calibration curves were used to evaluate the accuracy of the nomogram.

### The role of IRRS in the prediction of immunotherapeutic benefits

We compared the differences in several immunotherapeutic response indexes (ICBs expression, TMB score, TIDE score, microsatellite instability and IPS) between the risk groups to assess the predictive ability of the risk signature in the prediction of immunotherapeutic benefits. Patients in IMvigor210 and GSE78220 cohorts were classified into responder (including partial response (PR) and complete response (CR)) and non-responder (including progressive disease (PD) and stable disease (SD)) groups according to the patients’ response to immunotherapy. Then, we calculated the risk score of each patient in IMvigor210 and GSE78220 cohorts based on the formula generated in TCGA cohort and analyzed its impact on the prognosis and the efficacy of immunotherapy.

### Statistical analysis

All visualization and statistical analyses were performed by using R software (version 4.2.1, https://www.r-project.org/) and the corresponding feature packages. Two-sided *P*-value < 0.05 was considered as significant thresholds for all statistical tests.

### Availability of data and materials

The datasets generated and/or analyzed during the current study are available in the Gene Expression Omnibus (GEO, https://www.ncbi.nlm.nih.gov/geo/), The Cancer Genome Atlas (TCGA, https://portal.gdc.cancer.gov/), and Tumor Immune Dysfunction and Exclusion (TIDE, http://tide.dfci.harvard.edu/) projects.

## RESULTS

### Identification of IRGs in GC

**Figure 1:**
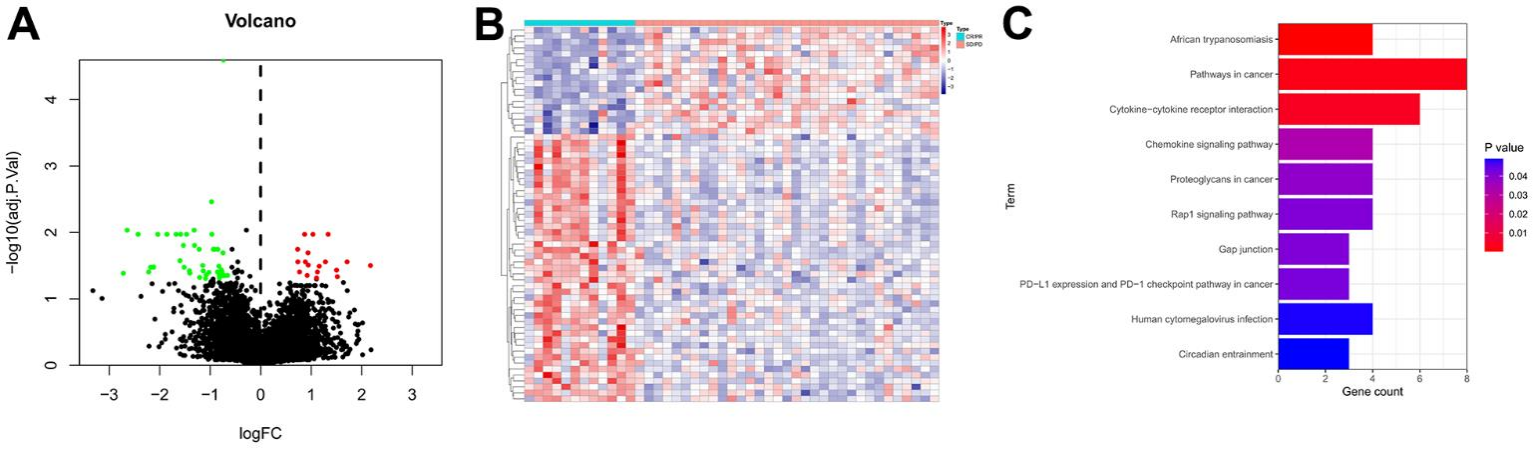
**Identification of IRGs.** (**A**) Volcano plot of IRGs. The red dots represent the upregulated genes, the green dots represent the downregulated genes, and the black dots represent genes with no significant difference in expression. (**B**) Expression heatmap of the IRGs. Red represents upregulated genes, and blue represents downregulated genes. (**C**) KEGG enrichment analysis of the IRGs.

The RNA-sequencing profiles and corresponding clinical parameters of GC patients in PRJEB25780 dataset were obtained from the TIDE database. Then, a total of 63 genes that differentially expressed between the responder and non-responder groups were identified as IRGs (Supplementary Table 1). The volcano plot of the IRGs was displayed in Figure 1A, 1B presented the expression heatmap of these IRGs. KEGG pathway annotation analysis revealed that the IRGs were primarily enriched in Pathways in cancer, Cytokine-cytokine receptor interaction, Rap1 signaling pathway, and PD-L1 expression and PD-1 checkpoint pathway in cancer (Figure 1C).

### Genetic variation landscapes of IRGs in GC

**Figure 2:**
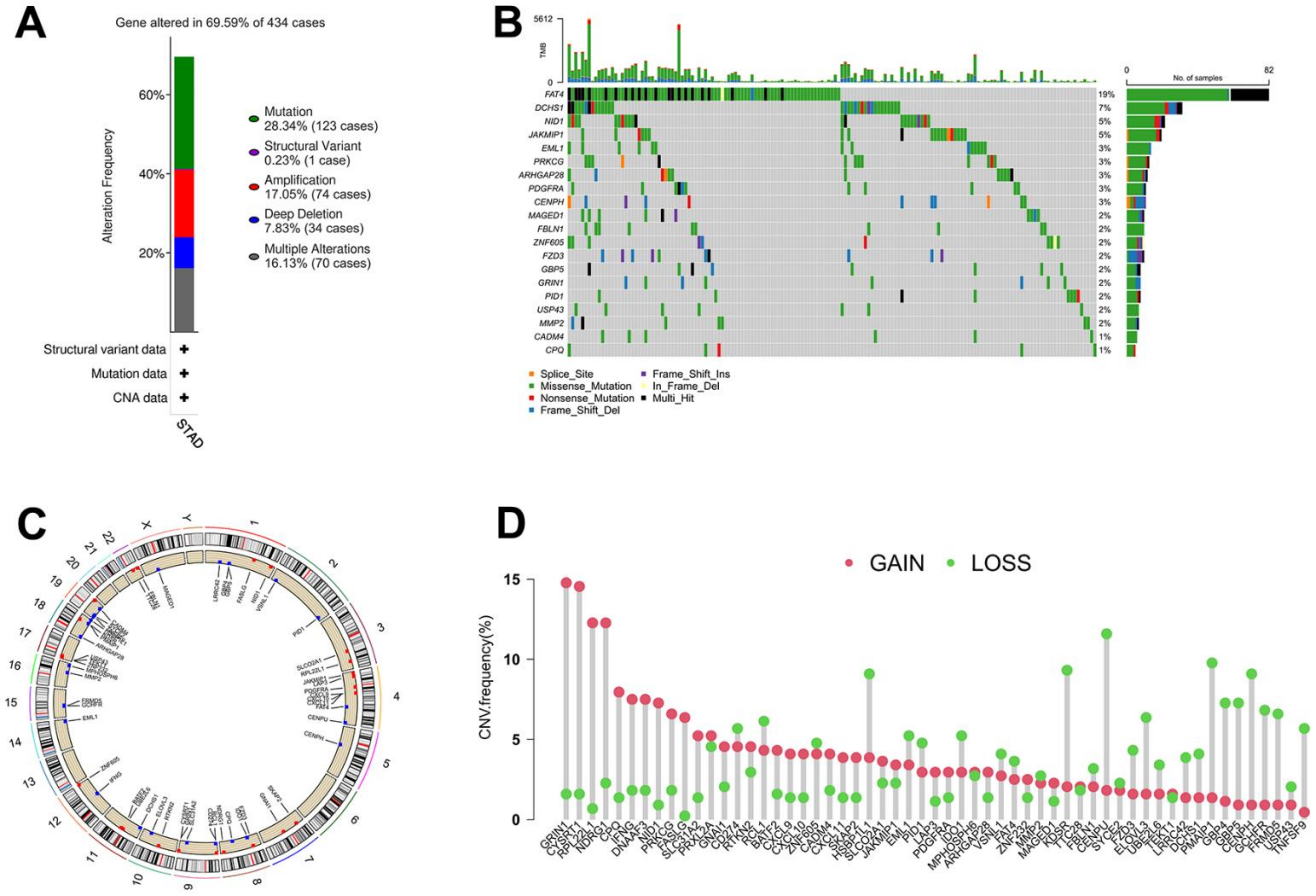
**The alterations of IRGs in GC.** (**A**) Gene alteration frequency of IRGs in GC based on TCGA project. (**B**) Landscape of genomic aberrations of the top 20 IRGs in GC. (**C**) The sites of CNV variation in IRGs on the 23 chromosomes. (**D**) Frequencies of CNV gain, loss, and non-CNV among IRGs.

The somatic mutation and CNV frequencies of the 63 IRGs in GC patients were analyzed based on TCGA cohort. As presented in Figure 2A, 2B, genetic alterations of the IRGs occurred in 302 of 434 (69.59%) GC patients. Among them, FAT4 (19%), DCHS1 (7%), NID1 (5%), and JAKMIP1 (5%) possess the highest mutation frequency, and their main mutation types are missense mutation, frame shift del, and nonsense mutation. The changes in IRGs with CNV features on chromosomes was showed in Figure 2C, 57 out of the 63 IRGs had frequent copy number alterations, and most of the IRGs were accumulated on copy number loss rather than copy number gain (Figure 2D).

### Identification of IRG subtypes in GC

**Figure 3:**
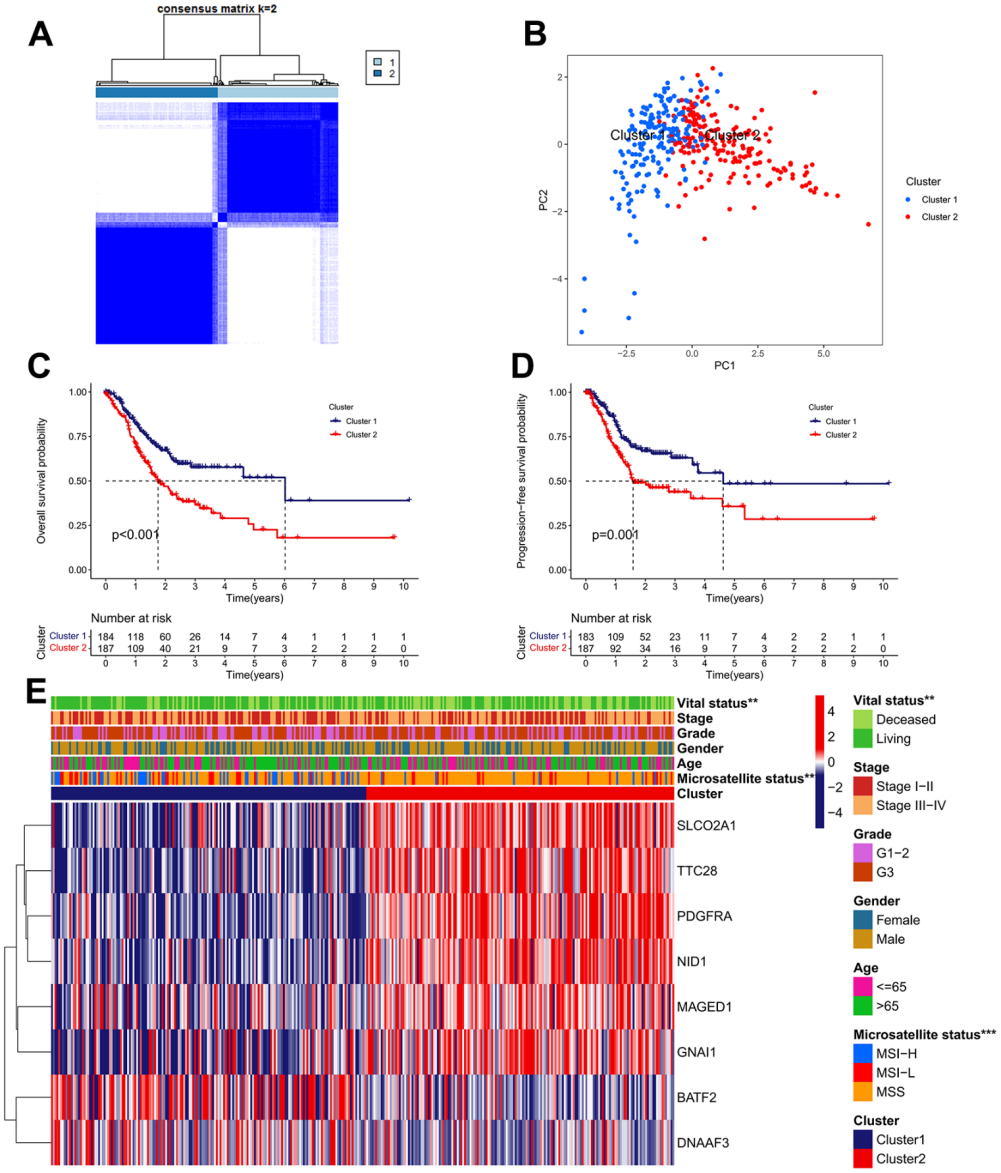
**Identification of IRG subtypes in GC.** (**A**) Consensus matrix heatmap defining two clusters (k = 2) and their correlation area. (**B**) A considerable transcriptome divergence between the two subtypes is seen by PCA analysis. (**C**) The Kaplan–Meier curve of OS analysis between different cluster groups. (**D**) The Kaplan–Meier curve of PFS analysis between different cluster groups. (**E**) Differences in clinicopathologic features and expression levels of IRGs between the two subtypes.

An unsupervised clustering strategy was used to classify 371 GC samples into cluster 1 (n=184) and cluster 2 (n=187) subgroups based on the expression patterns of 63 IRGs (Figure 3A). The principal component analysis (PCA) revealed that the discrimination between cluster 1 and cluster 2 is great (Figure 3B). Kaplan-Meier survival analysis revealed that patients in cluster 1 possess better OS and progression free survival (PFS) rate than those in cluster 2 (Figure 3C, 3D). The correlations between the clinical features and expression patterns of the IRGs was presented in the heatmap (Figure 3E). Patients of cluster 1 had a higher proportion of microsatellite instability-high (MSI-H) and alive outcome than cluster 2.

### Analysis of TME infiltration in distinct subtypes

**Figure 4:**
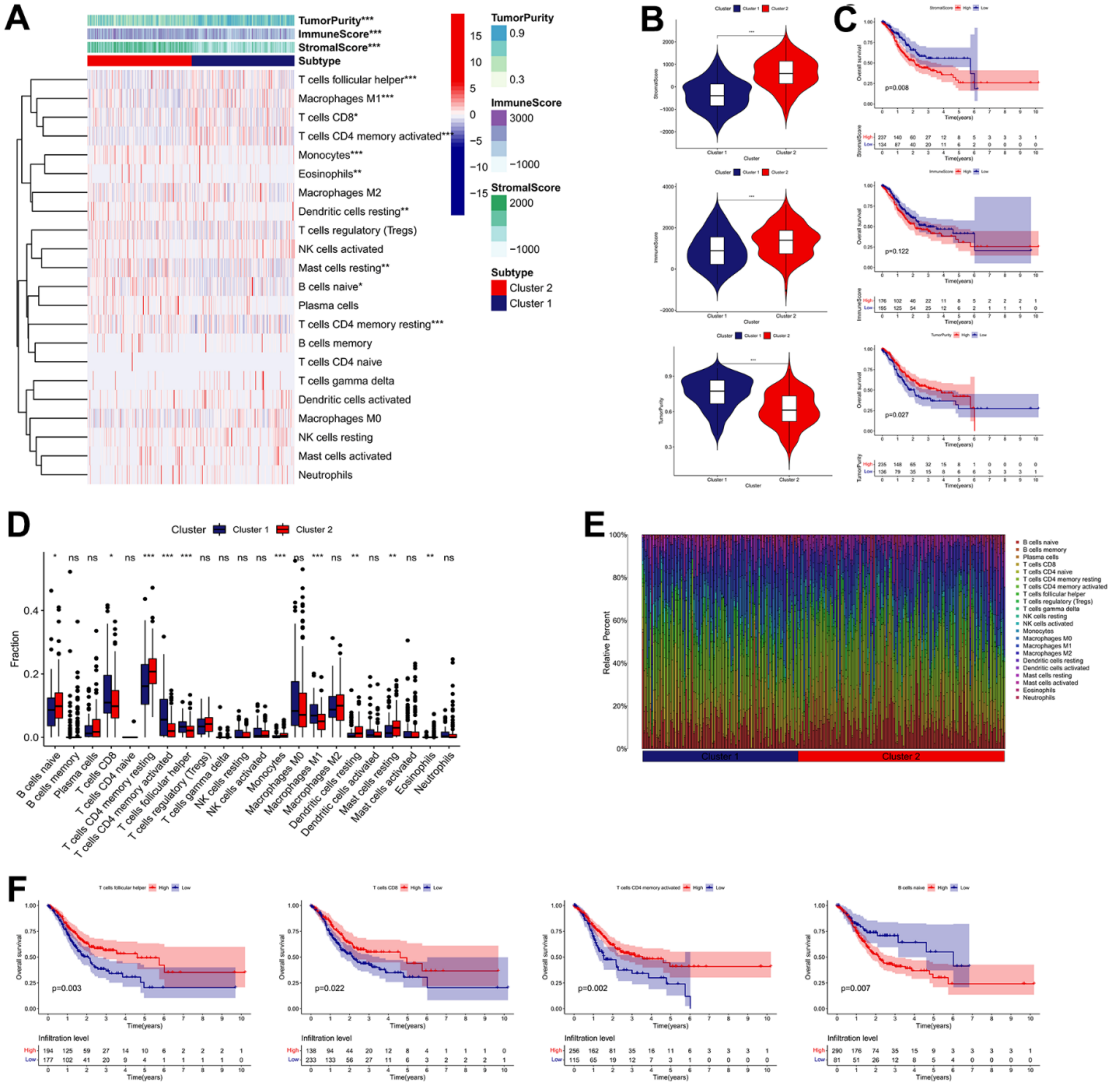
**Immune landscape of IRG subtypes in the TCGA cohort.** (**A**) Differences in TME scores and infiltration levels of immune cells between the two distinct subgroups. (**B**) The comparisons of stromal score, immune score, and tumor purity between different subgroups. (**C**) The Kaplan–Meier survival regarding the stromal score, immune score, and tumor purity in GC patients. (**D**) The boxplot of the differences of the immune cells infiltration between two distinct subgroups. (**E**) Relative proportion of immune infiltration in two distinct subgroups. (**F**) The Kaplan–Meier survival analysis of the correlation between the infiltration levels of the immune cells and OS in GC. *P < 0.05, **P < 0.01, ***P < 0.001.

We carried out the “CIBERSORT” and “ESTIMATE” methods to determine the difference in TME infiltration, including 22 subtypes of immune cells and TME scores, between the two clusters in order to learn more about how IRGs work in the TME. As presented in Figure 4A, the difference in TME infiltration between these two clusters is great. Samples in cluster 1 seemed to exhibit remarkably lower stromal scores, lower immune scores, and higher tumor purity scores, compared with those of cluster 2 (Figure 4B). Kaplan-Meier survival analysis revealed that high stromal scores and low tumor purity scores were remarkably associated with poor prognosis in GC patients (Figure 4C). With regard to immune cell infiltration, the cluster 1 subgroup was characterized by the high infiltration of follicular helper T cells, activated memory CD4+ T cells, CD8+ T cells and M1 macrophages, whereas the cluster 2 was characterized by the high infiltration of resting memory CD4+ T cells, naive B cells, resting Dendritic cells, Monocytes, resting Mast cells, and Eosinophils (Figure 4D, 4E). Among them, elevated infiltration abundances of CD8+ T cells, follicular helper T cells and activated memory CD4+ T cells were significantly correlated with favorable clinical outcome of GC, while elevated infiltration abundance of naive B cells was unfavorable (Figure 4F).

**Figure 5:**
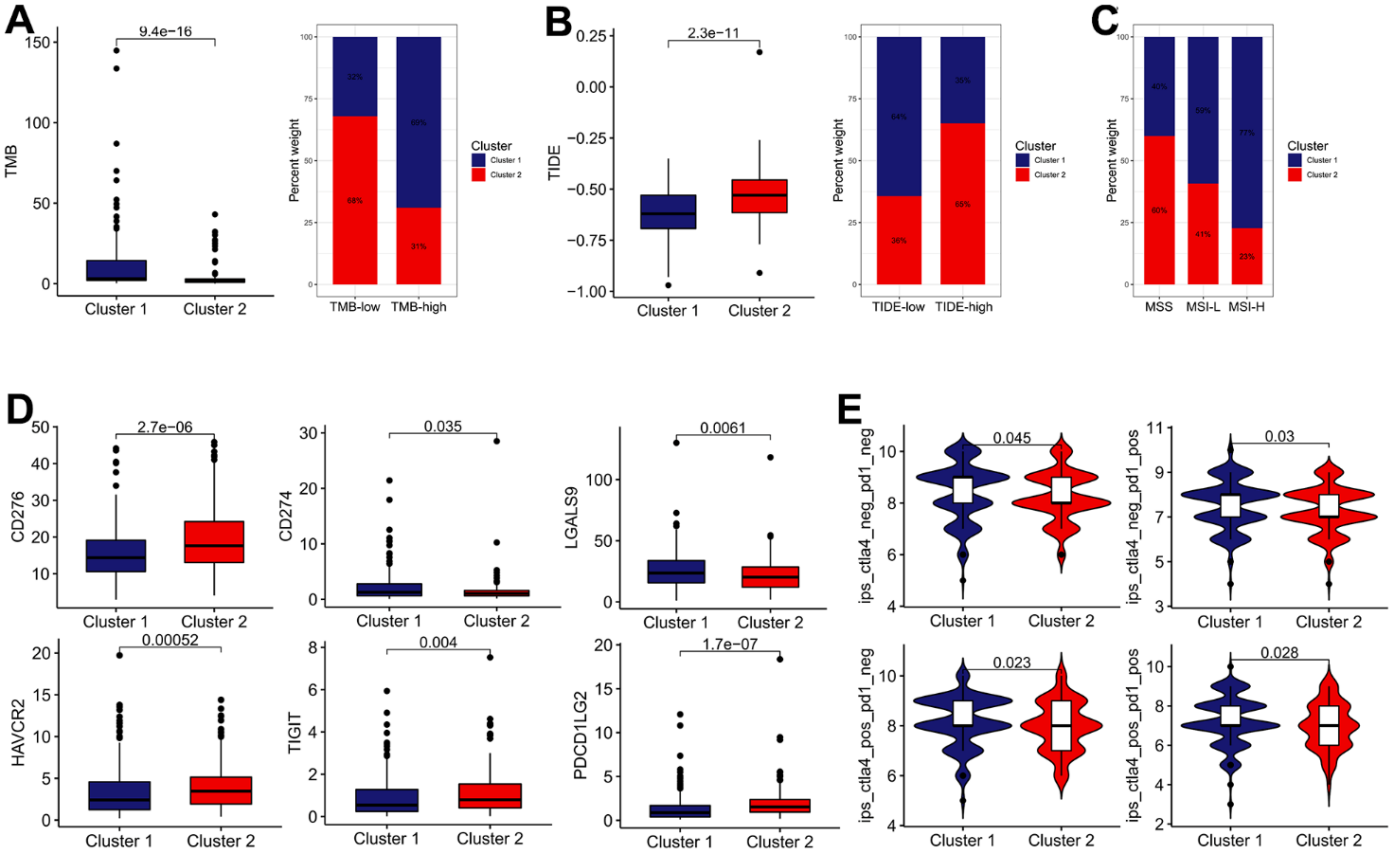
**The estimation of two IRGs subtypes in immunotherapy response.** (**A**) The difference in TMB between two distinct subtypes. (**B**) The difference in TIDE score between two distinct subtypes. (**C**) Relative proportion of microsatellite instability in two distinct subtypes. (**D**) The expression of ICBs in two distinct subtypes. (**E**) The difference of IPS in two distinct subtypes.

Subsequently, we determined whether the IRG subtypes had a significant correlation with the immunotherapy effect. Patients in cluster 1 possess higher TMB score than those in cluster 2, whereas the cluster 2 possess higher TIDE score (Figure 5A, 5B). Besides, the cluster 1 was characterized by the high proportion of MSI-H and low proportion of microsatellite stability (MSS), compared with cluster 2 (Figure 5C). In terms of ICBs, CD274 (also known as PD-L1) and LGALS9 expression in cluster 1 were significantly elevated, while the expression of CD276, HAVCR2, TIGIT, and PDCD1LG2 in cluster1 were downregulated (Figure 5D). Moreover, we calculated the IPS (CTLA4-/PD-1-, CTLA4+/PD-1-, CTLA4-/PD-1+ and CTLA4+/PD-1+) to reveal the immunogenicity of patients in each subgroup. As a result, the IPS were remarkably elevated in the cluster 1 subgroup, which appeared to have stronger immunogenicity. (Figure 5E). In sum, these findings implied that effective immune response was more activated in the cluster 1 group, which mean that the cluster 1 group exhibited a better response to immunotherapy.

### Construction of the IRRS signature

**Figure 6:**
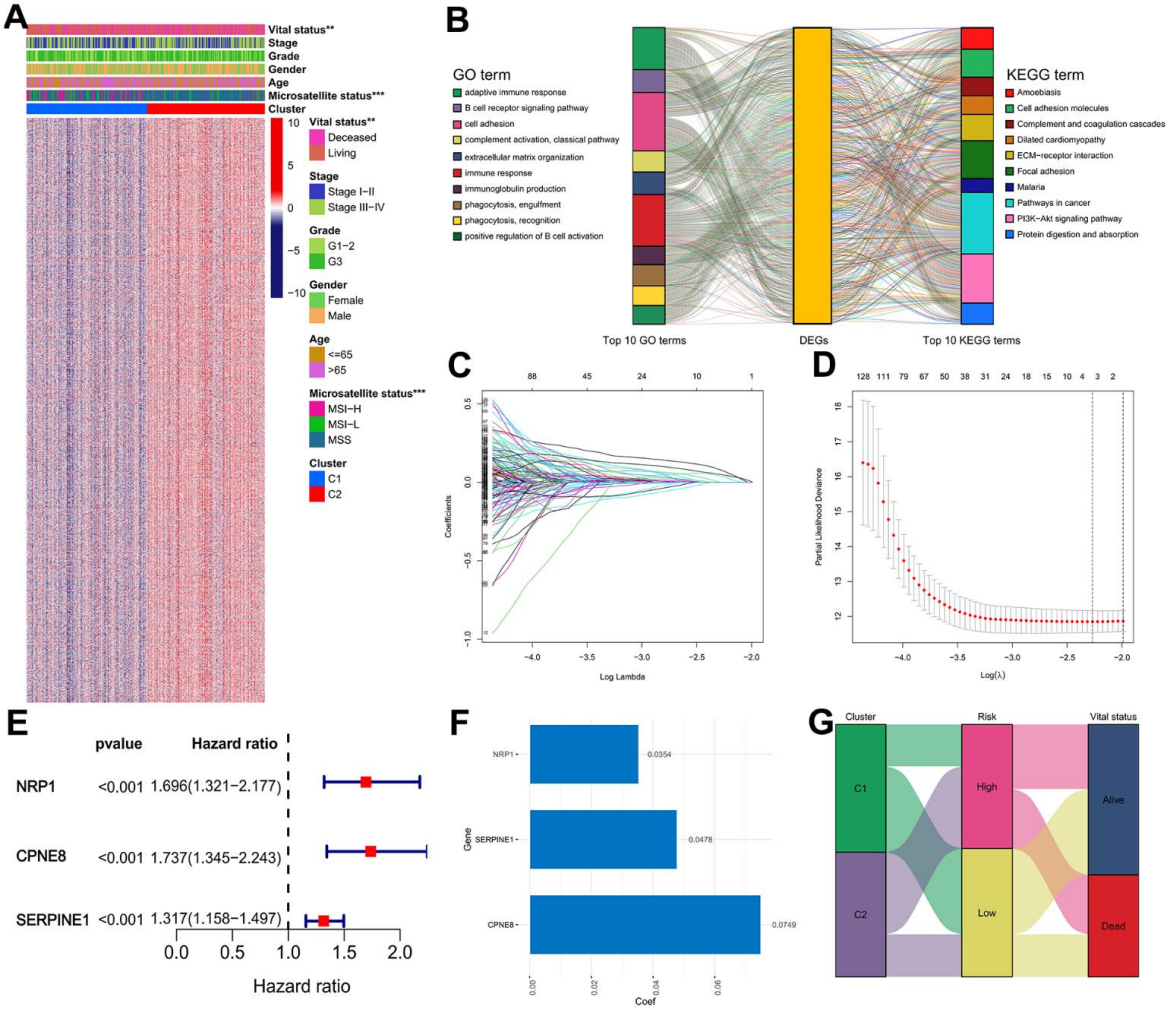
**Construction of the IRRS model in the TCGA cohort.** (**A**) Expression heatmap of the DEGs between the two distinct subtypes. (**B**) GO and KEGG enrichment studies of DEGs between different cluster groups. (**C**) LASSO coefficient profiles of 3 selected genes in the 10-fold cross-validation. (**D**) Partial likelihood deviance was revealed by the LASSO regression model in the 10-fold cross-validation. (**E**) Forest plot of hazard ratios for three selected prognostic variables. (**F**) The coefficients of the three prognostic variables in the LASSO regression model. (**G**) Sankey plot displayed the correlations among the cluster subgroups, IRRS and vital status.

As listed in Supplementary Table 2, 3065 DEGs (|log FC| ≥ 0.585, adjusted *P* < 0.05) between cluster 1 and cluster 2 subgroups were identified, and the expression heatmap of these DEGs was presented in Figure 6A. GO annotation analysis revealed that the DEGs are significantly annotated in immune-associated crosstalk, such as immune response, adaptive immune response, B cell receptor signaling pathway, and phagocytosis (Figure 6B and Supplementary Table 3). KEGG pathway annotation analysis found that the DEGs are primarily enriched in several areas of carcinogenesis-associated pathways, including ECM-receptor interaction, Focal adhesion, PI3K-Akt signaling pathway, and Pathways in cancer (Figure 6B and Supplementary Table 4). Subsequently, 625 genes presented significant correlations with the OS of GC patients were filtered out for further research using univariate Cox analysis (Supplementary Table 5), and three of which were eventually screened out for constructing the IRRS signature via utilizing LASSO regression analysis with minimized lambda (Figure 6C, 6D). The forest plot illustrated the correlations between the expression levels of the three genes in the IRRS signature and the prognosis of GC patients (Figure 6E). As presented in Figure 6F, we applied the following equation to calculate the risk score of each patient: IRRS = (Exp_NRP1_ × 0.0354) + (Exp_SERPINE1_ × 0.0478) + (Exp_CPNE8_ × 0.0749). According to the median value of the risk score, 371 samples in the TCGA GC cohort were divided into the low- (n = 186) and high-risk (n = 185) groups, and the association of IRRS, IRG subtypes, and vital status of these patients was showed in the Sankey plot (Figure 6G).

### IRRS for the prognostic prediction of GC

**Figure 7:**
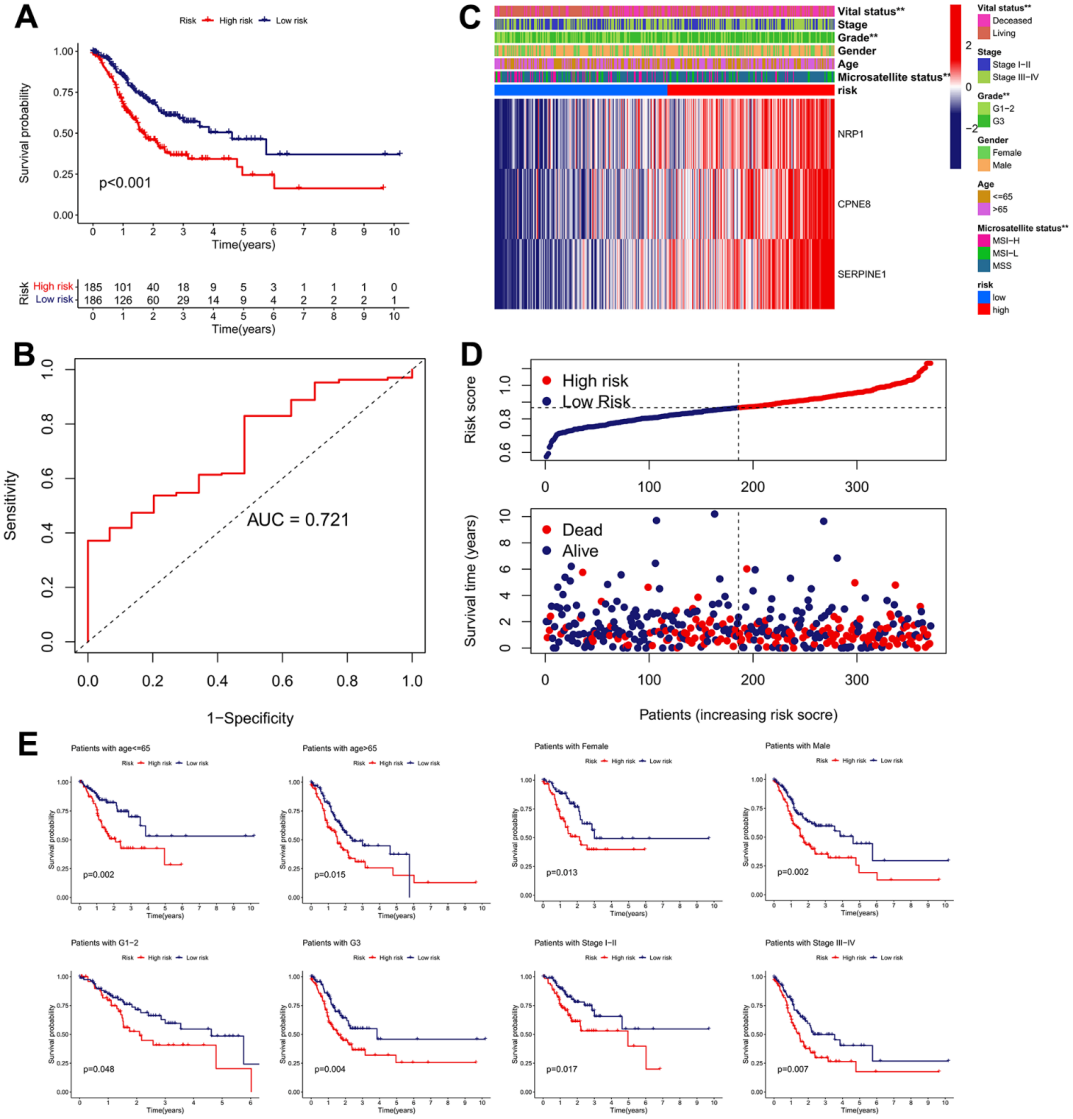
**Clinical significance of the IRRS model in TCGA cohort.** (**A**) The Kaplan–Meier survival analysis of the signature for predicting the OS of patients in TCGA cohort. (**B**) Time-dependent ROC analysis of the signature for predicting the OS of patients in TCGA cohort. (**C**) Differences in clinicopathologic features and expression levels of prognostic variables between the low- and high-risk groups. (**D**) The distribution of the IRRS and the vital status of patients in the TCGA cohort. (**E**) The subgroup survival analysis according to the age, gender, histologic grade, and tumor stage.

We determined the clinical significance of the IRRS in GC based on TCGA cohort. The Kaplan–Meier survival analysis revealed that patients in the high-risk subgroup possessed a significant poorer OS than patients in the low-risk subgroup (Figure 7A). The ROC curve for 5-year OS of the IRRS signature exhibited an area under ROC (AUC) value of 0.721, implying that the signature had moderate sensitivity and specificity (Figure 7B). Patients in the high-risk subgroup possessed a higher proportion of histologic G3, MSS, and deceased outcome than those in the low-risk subgroup, and the results of the chi-square test revealed that NRP1, CPNE8 and SERPINE1 were all overexpressed in the high-risk subgroup (Figure 7C). Besides, we found that patients’ risk of death increased with increasing risk score (Figure 7D). Furthermore, we conducted a subgroup analysis on the basis of age, gender, histologic grade, and TNM stage. As expected, the OS of GC patients was better in the low-risk subgroup than in the high-risk subgroup in each subgroup (Figure 7E).

### Validation of the IRRS in external cohorts

**Figure 8:**
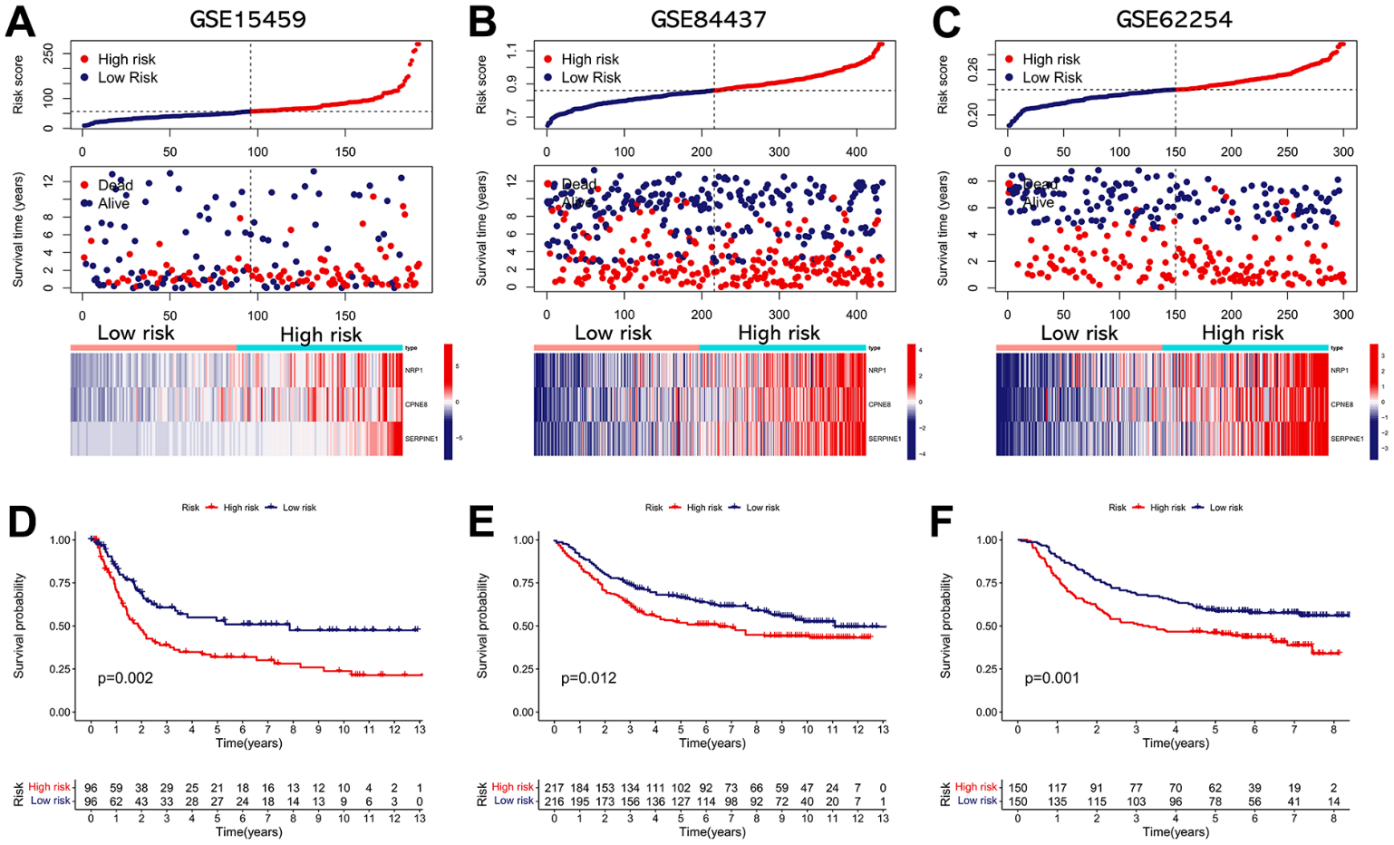
**External validation of the IRRS model in predicting OS of GC patients based on three independent cohorts.** (**A**) The distribution of the risk score, the vital status of patients, and the expression heatmap of three prognostic variables in the GSE15459 cohort. (**B**) The distribution of the risk score, the vital status of patients, and the expression heatmap of three prognostic variables in the GSE84437 cohort. (**C**) The distribution of the risk score, the vital status of patients, and the expression heatmap of three prognostic variables in the GSE62254 cohort. (**D**) The Kaplan–Meier survival analysis of the IRRS signature for predicting the OS of patients in the GSE15459 cohort. (**E**) The Kaplan–Meier survival analysis of the IRRS signature for predicting the OS of patients in the GSE84437 cohort. (**F**) The Kaplan–Meier survival analysis of the IRRS signature for predicting the OS of patients in the GSE62254 cohort.

**Figure 9:**
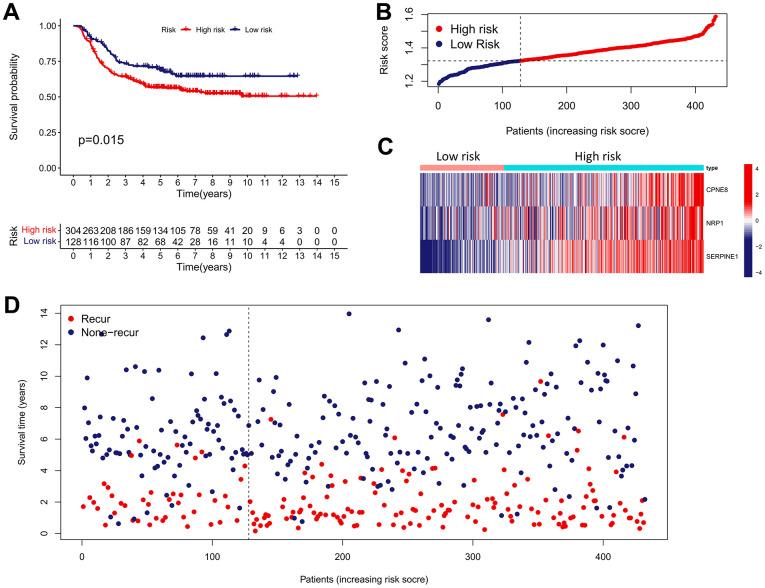
**External validation of the IRRS model in predicting RFS of GC based on the GSE26253 cohort.** (**A**) The Kaplan–Meier survival analysis of the IRRS signature for predicting the RFS of GC patients based on the GSE26253 cohort. (**B**) The distribution of the risk score of GC patients in the GSE26253 cohort. (**C**) The expression heatmap of three prognostic variables in the GSE26253 cohort. (**D**) The distribution of the recurrent status of GC patients in the GSE26253 cohort.

We performed validation analysis for evaluating whether the IRRS has clinical application value in distinct populations. First, the risk score for individual patients in GSE15459, GSE84437, and GSE62254 cohorts was calculated according to the calculation formula derived from TCGA cohort. Similarly, GC patients from these validation cohorts were divided into low- and high-risk subgroups according to the median value of the risk score (Figure 8A–8C). As shown in Figure 8D–8F, obvious differences in the OS probability were observed between the low- and high-risk subgroups in each validation cohort, GC patients with higher risk score seemed to exhibit poorer prognosis. In addition, we further determined whether the IRRS has predictive ability in relapse-free survival (RFS) of GC patients from GSE26253 cohort. Consistent with the results in OS, patients with higher risk score possessed higher rates of tumor recurrence (Figure 9). To sum up, these findings indicated the reliable capability of the IRRS in distinguishing differences in clinical outcomes of GC.

### The IRRS can be served as an independent risk predictor in GC

**Figure 10:**
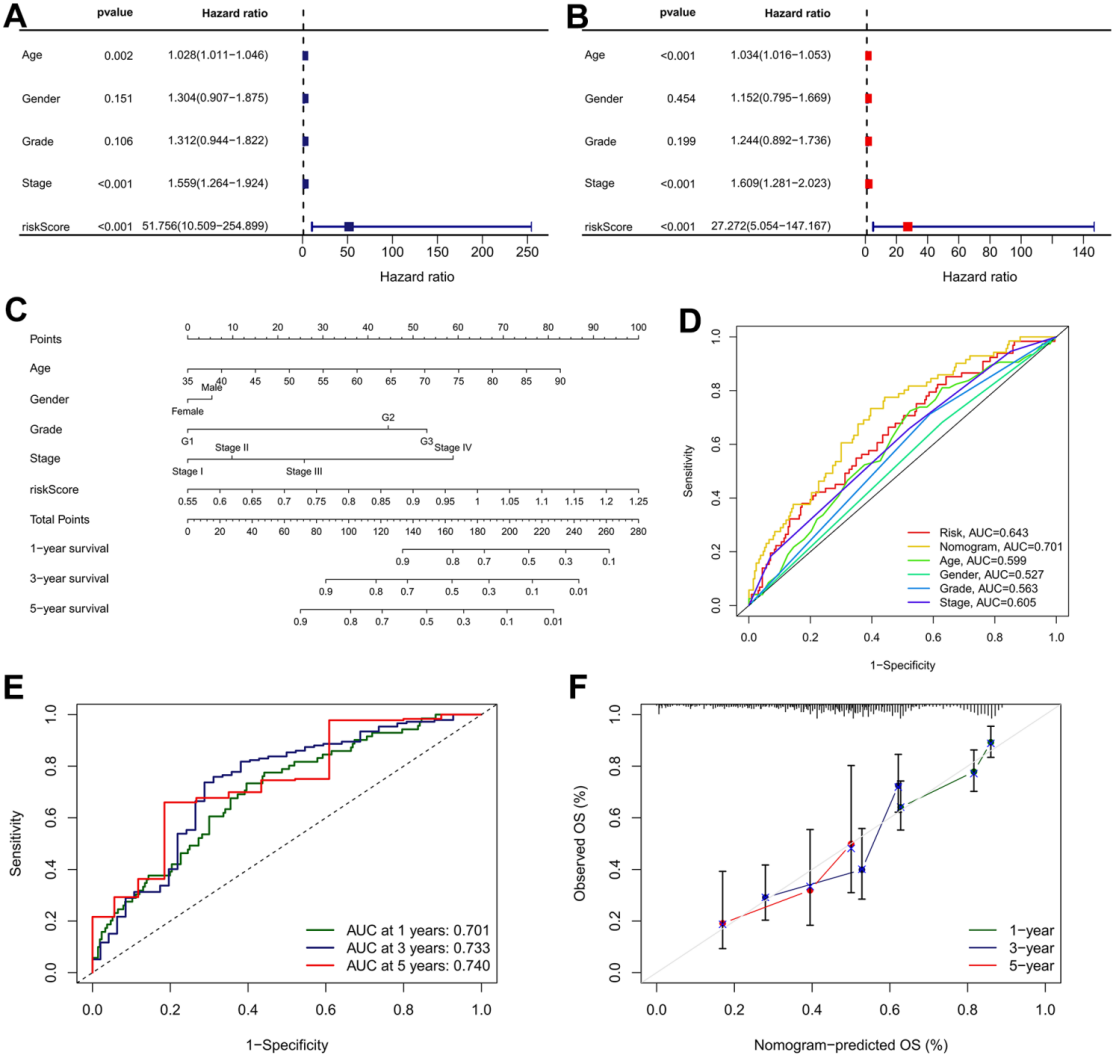
**Nomogram developed for predicting the probability of 1-, 3- and 5-year OS in TCGA cohort.** (**A**) Univariate Cox analysis containing IRRS and clinicopathological parameters. (**B**) Multivariate Cox analysis containing IRRS and clinicopathological parameters. (**C**) The comprehensive nomogram for predicting probabilities of GC patients with 1-, 3- and 5-year OS in TCGA dataset. (**D**) A comparison of ROC curve showed the superiority of the nomogram. (**E**) ROC curves chart of the comprehensive nomogram predicting the 1-, 3- and 5-year survival rate. (**F**) Calibration plot of nomogram for predicting probabilities of 1-, 3-, and 5-year survival of GC patients. Nomogram-predicted probability of survival is plotted on the x-axis; actual survival is plotted on the y-axis.

To investigate whether the IRRS can be served as a clinically independent risk predictor for GC patients, the IRRS, age, gender, histologic grade, and TNM stage were enrolled as covariates to perform the univariate and multivariate Cox analyses. The results indicated that age, TNM stage and IRRS are independent prognostic predictor that could be used for predicting the prognosis of GC patients (Figure 10A, 10B). Then, a nomogram was built to improve the clinical power of the IRRS by combining the prognostic factors above (Figure 10C). Multi-variables ROC analysis indicated that the nomogram had optimum predictive performance compared with other single factors (Figure 10D). Time-dependent ROC of 1-, 3-, and 5-year OS also revealed that the nomogram had high predictive power (Figure 10E). Besides, calibration charts revealed that the predictive capability of the nomogram in 1-, 3-, and 5-year periods was highly accurate, confirming its utility in forecasting the clinical outcome of GC patients (Figure 10F).

### Risk score-based treatment strategy for GC

**Figure 11:**
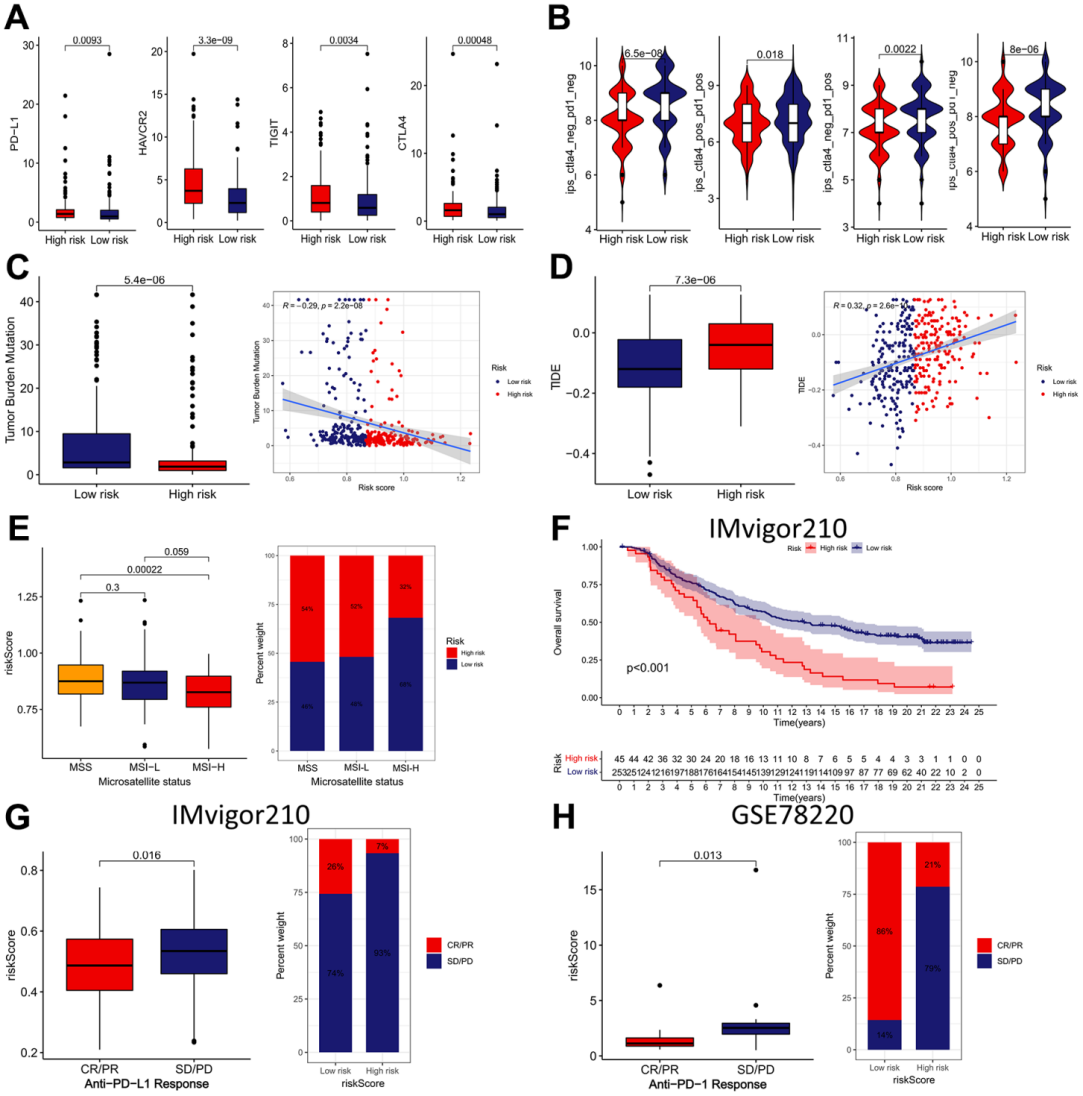
**The immunotherapeutic benefit of the IRRS model.** (**A**) The expression of four ICBs (PD-L1, HAVCR2, TIGIT, and CTLA4) in low- and high-risk groups. (**B**) The difference in IPS scores between low- and high-risk groups. (**C**) Box plots and scatter diagram of the TMB score and IRRS. (**D**) Box plots and scatter diagram of the TIDE score and IRRS. (**E**) Boxplot and Bar diagram of the microsatellite instability and IRRS. (**F**) Kaplan–Meier curve of OS for patients with high and low IRRS subtypes in IMvigor210 cohort. (**G**) Boxplot and Bar diagram displayed the response to immunotherapy in low- and high-risk groups in IMvigor210 cohort. (**H**) Boxplot and Bar diagram displayed the response to immunotherapy in low- and high-risk groups in GSE78220 cohort.

The expression of four ICBs (PD-L1, HAVCR2, TIGIT, and CTLA4) were compared between the low- and high-risk subgroups. As shown in Figure 11A, the results found that the presented immunomodulators were significantly upregulated in the high-risk subgroup. Subsequently, we determined the association between the IRRS and IPS (CTLA4-/PD-1-, CTLA4+/PD-1-, CTLA4-/PD-1+ and CTLA4+/PD-1+), which are recognized indexes to predict patients’ responses to ICIs by evaluating the immunogenicity. The results demonstrated that the IPS were remarkably elevated in the low-risk subgroup, implying that GC patients in the low-risk subgroup might be more sensitive to anti-cancer immunotherapy (Figure 11B). We also compared the distribution of TMB and TIDE scores between the two IRRS subgroups and revealed that the TMB score was lower in the high-risk subgroup, and the TIDE score was contrary, indicating that patients in the high-risk subgroup might respond worse to anti-cancer immunotherapy (Figure 11C, 11D). Besides, we explored the correlation between IRRS and microsatellite instability, and revealed that patients in the low-risk subgroup possess a higher percentage of MSI-H, and MSS more happened in patients in the high-risk subgroup (Figure 11E). In sum, all these results implied that GC patients in low-risk subgroup exhibited a better response to anti-cancer immunotherapy, IRRS might be a potential biomarker for immunotherapy of GC.

Subsequently, IMvigor210 and GSE78220 cohorts were applied as external validation cohort for verifying the predictive ability of the IRRS in anti-tumor immunotherapy. In the IMvigor210 cohort, patients in the low-risk subgroup had remarkably better prognosis than those in the high-risk subgroup (Figure 11F).

Besides, we analyzed the differences in immunotherapy response subtypes (CR, PR, SD, and PD) between different IRRS subgroups and found that patients with lower risk score possess a higher percentage of CR/PR, and SD/PD more happened in patients with higher risk score (Figure 11G, 11H). These findings further confirmed that the IRRS can be served as a great biomarker to forecast the sensitive to immunotherapy.

## DISCUSSION

Despite great advancements in treatment of GC, the interpatient heterogeneity still poses a great challenge for forecasting the clinical outcome of the patients and adopting appropriate treatment strategies. Cancer immunotherapy is a rapidly developing research field which brings new hope to distinct tumor patients, including GC, and has gained great progress in both research field and clinical practice. With the development of cancer immunotherapy, several immunotherapy strategies are available for GC, and more strategies are under clinical research. However, the most recent immunotherapy strategies for the treatment of GC patients were not promising in most cases due to the limited therapeutic biomarkers and delayed diagnosis.

In this study, our goal is distinguishing the immune subtypes of GC to develop an immune landscape for choosing appropriate patients for immunotherapy and construct a novel risk model for forecasting the prognosis of GC patients. We first identified IRGs in GC by exploring the RNA-sequencing profiles of immunotherapy cohort. Subsequently, the GC patients were divided into two subtypes by applying unsupervised clustering method based on the expression pattern of the IRGs. Our results revealed that patients in cluster 1 had remarkably prolonged OS and PFS time than those in cluster 2. The TME is comprised of immune cells, stromal cells, fibroblasts, endothelial cells as well as tumor cells that closely interact with tumor prognosis and treatment options [33]. Thus, we further explored the correlation between two IRG subtypes and TME infiltration considering the significant role of TME. As a result, patients in cluster 1 seemed to exhibit remarkably lower stromal scores, lower immune scores, and higher tumor purity scores, compared to those of cluster 2. On the other hand, tumor immune infiltration analyses found that the infiltration abundances of activated memory CD4+ T cells, CD8+ T cells, follicular helper T cells and M1 macrophages are remarkably higher in cluster 1, consistent with the higher infiltration of CD8+ T cells, activated memory CD4+ T cells, and follicular helper T cells being correlated with improved prognosis in patients with GC, whereas a significantly higher infiltration abundances of naive B cells, resting memory CD4+ T cells, Monocytes, resting Dendritic cells, resting Mast cells, and Eosinophils in cluster 2 corresponds to the results that the elevated infiltration abundance of naive B cells was correlated with worse clinical outcome of GC patients. CD8+ T cells can specifically detect and eliminate cancer cells by secreting effector cytokines (tumor necrosis factor (TNF) and interferon-γ (IFNγ)) or cytotoxic molecules (such as perforin and granzymes) [34]. Follicular helper T cells are a subgroup of CD4+ T cells, which induced anti-tumor immunity by facilitating the differentiation and maturation of tumor-killing cells [35]. M1 macrophages are a subtype of tumor-associated macrophages, which have anti-tumor effects by macrophage-mediated cytotoxicity or antibody-dependent cell-mediated cytotoxicity (ADCC) [36]. Activated naive B cells were reported to promote the progression of malignant pleural effusion via the PD-1/PD-L1 regulation axis [37]. Monocytes can promote the dissemination of tumor cells through inducing immune tolerance and angiogenesis [38]. Taken together, these results seemed to imply that cluster 1 was characterized by immune activation due to the high infiltration abundances of CD8+ T cells, follicular helper T cells, and M1 macrophages, and leads to a better prognosis, whereas cluster 2, with high infiltration of naive B cells and Monocytes, was characterized by immunosuppression and therefore giving rise to a poor prognosis.

Considering the impact of IRG cluster subgroups on the immune characteristics and clinical outcomes of GC patients, we further developed a three-gene (NRP1, CPNE8, and SERPINE1) signature based on DEGs between the two IRG cluster subgroups to predict the prognosis of GC. NRP1, one kind of transmembrane glycoprotein, has been well-described to participate in various biological processes, such as cell migration, cardiovascular development, angiogenesis, neuronal guidance, and immunology [39]. NRP1 expression levels were significantly upregulated in GC tissues and had positive correlation with the advanced tumor stage and worse clinical outcomes in GC patients [40]. NRP1 knockdown was reported to suppress the migration and invasion abilities of GC cells *in vivo*. CPNE8, a member of the copine family that has been reported to be overexpressed in GC, was positively correlated with aggressive clinical features, poor prognosis, and poor efficacy of immunotherapy in GC [41]. SERPINE1 is a member of the Serine protease inhibitor family and a main regulator of the plasminogen activator system [42]. SERPINE1 was overexpressed in GC and remarkably associated with advanced tumor stage and unfavorable prognosis, with the knockdown of SERPINE1 remarkably inhibiting the proliferation, invasion, and metastasis of GC cells *in vivo* and *in vitro* [43]. In our study, we combined the expression patterns of these three genes for developing a IRRS signature to predict the prognosis of GC patients. As a result, patients were classified into high- and low-risk subgroups according to the IRRS calculated using the risk genes. Survival analysis indicated that patients in the high-risk subgroup had worse clinical outcomes than those in the low-risk subgroup. Excitingly, external validation in four independent cohorts for verifying the universal applicability of the IRRS further affirmed that patients with low IRRS were accompanied by significantly prolonged OS or RFS time. In addition, univariate and multivariate Cox analyses found that the IRRS serves as an independent risk predictor in GC. In sum, these results implied that the IRRS can help clinicians develop personalized therapeutic strategies for GC in the future. For instance, patients with high IRRS required continual follow-up to monitor the relapse of GC.

Nowadays, a series of biomarkers for predicting the responsiveness of immunotherapy for cancer have been identified, including ICB, TMB, TIDE score, microsatellite instability and IPS. The ICBs are a class of inhibitory or stimulatory molecules mainly expressed on immune cells, which are crucial for inducing the self-tolerance and regulating the immune responsiveness of effectors in different tissues to avoid the tissue damage [44]. However, in the immunosuppressive TME, tumor cells can restrain the activity of immune cells and achieve immune escape by up-regulating the expression of ICBs on immune cells [45]. In the past two decades, emerging studies confirmed that the blockade of ICBs (PD-1, PD-L1, CTLA4, TIM-3, TIGIT etc.) has rapidly become the most promising anti-cancer strategies for multiple types of cancer, including GC [13, 14, 46]. The TMB is defined as the number of mutations seen in a section of DNA in a tumor cell and reported as mutations per megabase (mut/Mb) [47]. High TMB may contribute to the generation of new antigens that can be detected by immune cells, and thereby triggering anti-tumor immune response [48]. Thus, TMB has been recognized as a potential biomarker for cancer patients treated with ICIs, and the FDA has approved pembrolizumab in solid cancers with high TMB (defined as10 mut/Mb) [49]. The TIDE algorithm is developed for predicting the potential clinical responses to ICI treatment by integrating the gene expression signatures of T cell dysfunction and T cell exclusion [17, 50]. High TIDE scores indicate poor efficacy of ICI therapy, and TIDE has been demonstrated to be more accurate than the ICB expression levels and TMB at predicting clinical outcomes in cancer patients receiving immunotherapies [51]. In terms of microsatellite instability, it is known that MSI-H leads to accumulation of somatic mutations in tumor cells, thereby causing a battery of molecular and biological variations including high TMB, enhanced expression of new antigens and abundant tumor-infiltrating lymphocytes (TIL) [52]. Thus, patients with cancer in the MSI-H group had significantly increased sensitivity to immunotherapy in contrast to the MSS/MSI-L group, and the FDA granted pembrolizumab for the treatment of all MSI-H solid tumors, including GC [53]. IPS is defined based on tumor immune infiltration characteristics and bridges immune cell infiltration with immunogen subtypes, which can forecast the response to immunotherapy strategies including CTLA4 and PD-1 inhibitors [19]. Higher IPS scores are positively associated with the increased immunogenicity [54]. In this study, we revealed that the IRRS was remarkably associated with ICBs expression, TMB, TIDE score, microsatellite instability and IPS, which indirectly implied that the IRRS may serves as a significant role in forecasting the responsiveness of anti-cancer immunotherapy. Especially, we found that the IRRS can be used as an effective predictor for predicting the effects of immunotherapy in urothelial carcinoma cohort with anti-PD-L1 treatment (IMvigor210) and malignant melanoma cohort with anti-PD-1 treatment (GSE78220). This evidence further confirmed that the IRRS can be served as a great biomarker to predict the response to anti-cancer immunotherapy.

This study inevitably existed several limitations. Firstly, although we have developed a novel prognostic model on the basis of IRG and verified its applicability in external cohorts, the correlation between its members and immunotherapy remains largely unclear, functional and mechanistic experiments are required to verify and explain the regulatory mechanisms of these genes on immunotherapy in GC. Secondly, since the data analyzed in our study were retrospectively collected from different databases, it is difficult to cover all variations among patients from different regions due to interpatient tumor heterogeneity. Thus, a well-designed, prospective, multicenter study is required to further validate the accuracy of the IRRS signature, which will be time-consuming.

## CONCLUSIONS

In conclusion, this study identified immunotherapy-related genes in GC based on immunotherapy cohort and analyzed the role of these genes in GC prognosis and correlation with TME and developed a prognosis prediction signature. The prognostic prediction signature exhibits compelling clinical values in forecasting the prognosis and immunotherapy responsiveness for GC, which can be used as a powerful index for prognosis prediction and treatment guidance.

## Supplementary Material

Supplementary Table 1

Supplementary Table 2

Supplementary Table 3

Supplementary Table 4

Supplementary Table 5

## References

[1] Sung H, Ferlay J, Siegel RL, Laversanne M, Soerjomataram I, Jemal A, Bray F. Global Cancer Statistics 2020: GLOBOCAN Estimates of Incidence and Mortality Worldwide for 36 Cancers in 185 Countries. CA Cancer J Clin. 2021; 71:209–49. 10.3322/caac.21660 33538338

[2] Smyth EC, Nilsson M, Grabsch HI, van Grieken NC, Lordick F. Gastric cancer. Lancet. 2020; 396:635–48. 10.1016/S0140-6736(20)31288-5 32861308

[3] Thrift AP, El-Serag HB. Burden of Gastric Cancer. Clin Gastroenterol Hepatol. 2020; 18:534–42. 10.1016/j.cgh.2019.07.045 31362118 PMC8859863

[4] Oki E, Tokunaga S, Emi Y, Kusumoto T, Yamamoto M, Fukuzawa K, Takahashi I, Ishigami S, Tsuji A, Higashi H, Nakamura T, Saeki H, Shirabe K, et al, and Kyushu Study Group of Clinical Cancer. Surgical treatment of liver metastasis of gastric cancer: a retrospective multicenter cohort study (KSCC1302). Gastric Cancer. 2016; 19:968–76. 10.1007/s10120-015-0530-z 26260876

[5] Nikolaou M, Pavlopoulou A, Georgakilas AG, Kyrodimos E. The challenge of drug resistance in cancer treatment: a current overview. Clin Exp Metastasis. 2018; 35:309–18. 10.1007/s10585-018-9903-0 29799080

[6] Picard E, Verschoor CP, Ma GW, Pawelec G. Relationships Between Immune Landscapes, Genetic Subtypes and Responses to Immunotherapy in Colorectal Cancer. Front Immunol. 2020; 11:369. 10.3389/fimmu.2020.00369 32210966 PMC7068608

[7] Dai S, Xu S, Ye Y, Ding K. Identification of an Immune-Related Gene Signature to Improve Prognosis Prediction in Colorectal Cancer Patients. Front Genet. 2020; 11:607009. 10.3389/fgene.2020.607009 33343640 PMC7746810

[8] Darvin P, Toor SM, Sasidharan Nair V, Elkord E. Immune checkpoint inhibitors: recent progress and potential biomarkers. Exp Mol Med. 2018; 50:1–11. 10.1038/s12276-018-0191-1 30546008 PMC6292890

[9] Keir ME, Butte MJ, Freeman GJ, Sharpe AH. PD-1 and its ligands in tolerance and immunity. Annu Rev Immunol. 2008; 26:677–704. 10.1146/annurev.immunol.26.021607.090331 18173375 PMC10637733

[10] Liu J, Chen Z, Li Y, Zhao W, Wu J, Zhang Z. PD-1/PD-L1 Checkpoint Inhibitors in Tumor Immunotherapy. Front Pharmacol. 2021; 12:731798. 10.3389/fphar.2021.731798 34539412 PMC8440961

[11] Jiang Y, Chen M, Nie H, Yuan Y. PD-1 and PD-L1 in cancer immunotherapy: clinical implications and future considerations. Hum Vaccin Immunother. 2019; 15:1111–22. 10.1080/21645515.2019.1571892 30888929 PMC6605868

[12] Qu J, Mei Q, Liu L, Cheng T, Wang P, Chen L, Zhou J. The progress and challenge of anti-PD-1/PD-L1 immunotherapy in treating non-small cell lung cancer. Ther Adv Med Oncol. 2021; 13:1758835921992968. 10.1177/1758835921992968 33643442 PMC7890731

[13] Janjigian YY, Kawazoe A, Yañez P, Li N, Lonardi S, Kolesnik O, Barajas O, Bai Y, Shen L, Tang Y, Wyrwicz LS, Xu J, Shitara K, et al. The KEYNOTE-811 trial of dual PD-1 and HER2 blockade in HER2-positive gastric cancer. Nature. 2021; 600:727–30. 10.1038/s41586-021-04161-3 34912120 PMC8959470

[14] Wang FH, Zhang XT, Li YF, Tang L, Qu XJ, Ying JE, Zhang J, Sun LY, Lin RB, Qiu H, Wang C, Qiu MZ, Cai MY, et al. The Chinese Society of Clinical Oncology (CSCO): Clinical guidelines for the diagnosis and treatment of gastric cancer, 2021. Cancer Commun (Lond). 2021; 41:747–95. 10.1002/cac2.12193 34197702 PMC8360643

[15] Shitara K, Van Cutsem E, Bang YJ, Fuchs C, Wyrwicz L, Lee KW, Kudaba I, Garrido M, Chung HC, Lee J, Castro HR, Mansoor W, Braghiroli MI, et al. Efficacy and Safety of Pembrolizumab or Pembrolizumab Plus Chemotherapy vs Chemotherapy Alone for Patients With First-line, Advanced Gastric Cancer: The KEYNOTE-062 Phase 3 Randomized Clinical Trial. JAMA Oncol. 2020; 6:1571–80. 10.1001/jamaoncol.2020.3370 32880601 PMC7489405

[16] Kim ST, Cristescu R, Bass AJ, Kim KM, Odegaard JI, Kim K, Liu XQ, Sher X, Jung H, Lee M, Lee S, Park SH, Park JO, et al. Comprehensive molecular characterization of clinical responses to PD-1 inhibition in metastatic gastric cancer. Nat Med. 2018; 24:1449–58. 10.1038/s41591-018-0101-z 30013197

[17] Fu J, Li K, Zhang W, Wan C, Zhang J, Jiang P, Liu XS. Large-scale public data reuse to model immunotherapy response and resistance. Genome Med. 2020; 12:21. 10.1186/s13073-020-0721-z 32102694 PMC7045518

[18] Tomczak K, Czerwińska P, Wiznerowicz M. The Cancer Genome Atlas (TCGA): an immeasurable source of knowledge. Contemp Oncol (Pozn). 2015; 19:A68–77. 10.5114/wo.2014.47136 25691825 PMC4322527

[19] Charoentong P, Finotello F, Angelova M, Mayer C, Efremova M, Rieder D, Hackl H, Trajanoski Z. Pan-cancer Immunogenomic Analyses Reveal Genotype-Immunophenotype Relationships and Predictors of Response to Checkpoint Blockade. Cell Rep. 2017; 18:248–62. 10.1016/j.celrep.2016.12.019 28052254

[20] Ooi CH, Ivanova T, Wu J, Lee M, Tan IB, Tao J, Ward L, Koo JH, Gopalakrishnan V, Zhu Y, Cheng LL, Lee J, Rha SY, et al. Oncogenic pathway combinations predict clinical prognosis in gastric cancer. PLoS Genet. 2009; 5:e1000676. 10.1371/journal.pgen.1000676 19798449 PMC2748685

[21] Yoon SJ, Park J, Shin Y, Choi Y, Park SW, Kang SG, Son HY, Huh YM. Deconvolution of diffuse gastric cancer and the suppression of CD34 on the BALB/c nude mice model. BMC Cancer. 2020; 20:314. 10.1186/s12885-020-06814-4 32293340 PMC7160933

[22] Cristescu R, Lee J, Nebozhyn M, Kim KM, Ting JC, Wong SS, Liu J, Yue YG, Wang J, Yu K, Ye XS, Do IG, Liu S, et al. Molecular analysis of gastric cancer identifies subtypes associated with distinct clinical outcomes. Nat Med. 2015; 21:449–56. 10.1038/nm.3850 25894828

[23] Oh SC, Sohn BH, Cheong JH, Kim SB, Lee JE, Park KC, Lee SH, Park JL, Park YY, Lee HS, Jang HJ, Park ES, Kim SC, et al. Clinical and genomic landscape of gastric cancer with a mesenchymal phenotype. Nat Commun. 2018; 9:1777. 10.1038/s41467-018-04179-8 29725014 PMC5934392

[24] Barrett T, Wilhite SE, Ledoux P, Evangelista C, Kim IF, Tomashevsky M, Marshall KA, Phillippy KH, Sherman PM, Holko M, Yefanov A, Lee H, Zhang N, et al. NCBI GEO: archive for functional genomics data sets--update. Nucleic Acids Res. 2013; 41:D991–5. 10.1093/nar/gks1193 23193258 PMC3531084

[25] Necchi A, Joseph RW, Loriot Y, Hoffman-Censits J, Perez-Gracia JL, Petrylak DP, Derleth CL, Tayama D, Zhu Q, Ding B, Kaiser C, Rosenberg JE. Atezolizumab in platinum-treated locally advanced or metastatic urothelial carcinoma: post-progression outcomes from the phase II IMvigor210 study. Ann Oncol. 2017; 28:3044–50. 10.1093/annonc/mdx518 28950298 PMC5834063

[26] Hugo W, Zaretsky JM, Sun L, Song C, Moreno BH, Hu-Lieskovan S, Berent-Maoz B, Pang J, Chmielowski B, Cherry G, Seja E, Lomeli S, Kong X, et al. Genomic and Transcriptomic Features of Response to Anti-PD-1 Therapy in Metastatic Melanoma. Cell. 2016; 165:35–44. 10.1016/j.cell.2016.02.065 26997480 PMC4808437

[27] Ritchie ME, Phipson B, Wu D, Hu Y, Law CW, Shi W, Smyth GK. limma powers differential expression analyses for RNA-sequencing and microarray studies. Nucleic Acids Res. 2015; 43:e47. 10.1093/nar/gkv007 25605792 PMC4402510

[28] Huang da W, Sherman BT, Lempicki RA. Systematic and integrative analysis of large gene lists using DAVID bioinformatics resources. Nat Protoc. 2009; 4:44–57. 10.1038/nprot.2008.211 19131956

[29] Gao J, Aksoy BA, Dogrusoz U, Dresdner G, Gross B, Sumer SO, Sun Y, Jacobsen A, Sinha R, Larsson E, Cerami E, Sander C, Schultz N. Integrative analysis of complex cancer genomics and clinical profiles using the cBioPortal. Sci Signal. 2013; 6:pl1. 10.1126/scisignal.2004088 23550210 PMC4160307

[30] Wilkerson MD, Hayes DN. ConsensusClusterPlus: a class discovery tool with confidence assessments and item tracking. Bioinformatics. 2010; 26:1572–3. 10.1093/bioinformatics/btq170 20427518 PMC2881355

[31] Newman AM, Liu CL, Green MR, Gentles AJ, Feng W, Xu Y, Hoang CD, Diehn M, Alizadeh AA. Robust enumeration of cell subsets from tissue expression profiles. Nat Methods. 2015; 12:453–7. 10.1038/nmeth.3337 25822800 PMC4739640

[32] Yoshihara K, Shahmoradgoli M, Martínez E, Vegesna R, Kim H, Torres-Garcia W, Treviño V, Shen H, Laird PW, Levine DA, Carter SL, Getz G, Stemke-Hale K, et al. Inferring tumour purity and stromal and immune cell admixture from expression data. Nat Commun. 2013; 4:2612. 10.1038/ncomms3612 24113773 PMC3826632

[33] Hinshaw DC, Shevde LA. The Tumor Microenvironment Innately Modulates Cancer Progression. Cancer Res. 2019; 79:4557–66. 10.1158/0008-5472.CAN-18-3962 31350295 PMC6744958

[34] St Paul M, Ohashi PS. The Roles of CD8^+^ T Cell Subsets in Antitumor Immunity. Trends Cell Biol. 2020; 30:695–704. 10.1016/j.tcb.2020.06.003 32624246

[35] Lin X, Ye L, Wang X, Liao Z, Dong J, Yang Y, Zhang R, Li H, Li P, Ding L, Li T, Zhang W, Xu S, et al. Follicular Helper T Cells Remodel the Immune Microenvironment of Pancreatic Cancer via Secreting CXCL13 and IL-21. Cancers (Basel). 2021; 13:3678. 10.3390/cancers13153678 34359579 PMC8345153

[36] Pan Y, Yu Y, Wang X, Zhang T. Tumor-Associated Macrophages in Tumor Immunity. Front Immunol. 2020; 12:775758. 10.3389/fimmu.2020.583084 34956205 PMC8704129

[37] Wu XZ, Shi XY, Zhai K, Yi FS, Wang Z, Wang W, Pei XB, Xu LL, Wang Z, Shi HZ. Activated naïve B cells promote development of malignant pleural effusion by differential regulation of T_H_1 and T_H_17 response. Am J Physiol Lung Cell Mol Physiol. 2018; 315:L443–55. 10.1152/ajplung.00120.2018 29847991

[38] Ugel S, Canè S, De Sanctis F, Bronte V. Monocytes in the Tumor Microenvironment. Annu Rev Pathol. 2021; 16:93–122. 10.1146/annurev-pathmechdis-012418-013058 33497262

[39] Wang Y, Zhang L, Sun XL, Lu YC, Chen S, Pei DS, Zhang LS. NRP1 contributes to stemness and potentiates radioresistance via WTAP-mediated m6A methylation of Bcl-2 mRNA in breast cancer. Apoptosis. 2023; 28:233–46. 10.1007/s10495-022-01784-3 36333630

[40] Zhang L, Xing Y, Gao Q, Sun X, Zhang D, Cao G. Combination of NRP1-mediated iRGD with 5-fluorouracil suppresses proliferation, migration and invasion of gastric cancer cells. Biomed Pharmacother. 2017; 93:1136–43. 10.1016/j.biopha.2017.06.103 28738522

[41] Zhang P, Cao X, Guan M, Li D, Xiang H, Peng Q, Zhou Y, Weng C, Fang X, Liu X, Mao H, Li Q, Liu G, Lu L. CPNE8 Promotes Gastric Cancer Metastasis by Modulating Focal Adhesion Pathway and Tumor Microenvironment. Int J Biol Sci. 2022; 18:4932–49. 10.7150/ijbs.76425 35982908 PMC9379401

[42] Declerck PJ, Gils A. Three decades of research on plasminogen activator inhibitor-1: a multifaceted serpin. Semin Thromb Hemost. 2013; 39:356–64. 10.1055/s-0033-1334487 23504606

[43] Chen S, Li Y, Zhu Y, Fei J, Song L, Sun G, Guo L, Li X. SERPINE1 Overexpression Promotes Malignant Progression and Poor Prognosis of Gastric Cancer. J Oncol. 2022; 2022:2647825. 10.1155/2022/2647825 35132319 PMC8817868

[44] Zhang Y, Zheng J. Functions of Immune Checkpoint Molecules Beyond Immune Evasion. Adv Exp Med Biol. 2020; 1248:201–26. 10.1007/978-981-15-3266-5_9 32185712

[45] Li B, Chan HL, Chen P. Immune Checkpoint Inhibitors: Basics and Challenges. Curr Med Chem. 2019; 26:3009–25. 10.2174/0929867324666170804143706 28782469

[46] Topalian SL, Drake CG, Pardoll DM. Immune checkpoint blockade: a common denominator approach to cancer therapy. Cancer Cell. 2015; 27:450–61. 10.1016/j.ccell.2015.03.001 25858804 PMC4400238

[47] Fusco MJ, West HJ, Walko CM. Tumor Mutation Burden and Cancer Treatment. JAMA Oncol. 2021; 7:316. 10.1001/jamaoncol.2020.6371 33331847

[48] Sharma P, Siddiqui BA, Anandhan S, Yadav SS, Subudhi SK, Gao J, Goswami S, Allison JP. The Next Decade of Immune Checkpoint Therapy. Cancer Discov. 2021; 11:838–57. 10.1158/2159-8290.CD-20-1680 33811120

[49] Marcus L, Fashoyin-Aje LA, Donoghue M, Yuan M, Rodriguez L, Gallagher PS, Philip R, Ghosh S, Theoret MR, Beaver JA, Pazdur R, Lemery SJ. FDA Approval Summary: Pembrolizumab for the Treatment of Tumor Mutational Burden-High Solid Tumors. Clin Cancer Res. 2021; 27:4685–9. 10.1158/1078-0432.CCR-21-0327 34083238 PMC8416776

[50] Jiang P, Gu S, Pan D, Fu J, Sahu A, Hu X, Li Z, Traugh N, Bu X, Li B, Liu J, Freeman GJ, Brown MA, et al. Signatures of T cell dysfunction and exclusion predict cancer immunotherapy response. Nat Med. 2018; 24:1550–8. 10.1038/s41591-018-0136-1 30127393 PMC6487502

[51] Xia Y, Zhang R, Wang M, Li J, Dong J, He K, Guo T, Ju X, Ru J, Zhang S, Sun Y. Development and validation of a necroptosis-related gene prognostic score to predict prognosis and efficiency of immunotherapy in gastric cancer. Front Immunol. 2022; 13:977338. 10.3389/fimmu.2022.977338 36159818 PMC9504871

[52] Chang L, Chang M, Chang HM, Chang F. Microsatellite Instability: A Predictive Biomarker for Cancer Immunotherapy. Appl Immunohistochem Mol Morphol. 2018; 26:e15–21. 10.1097/PAI.0000000000000575 28877075

[53] Lemery S, Keegan P, Pazdur R. First FDA Approval Agnostic of Cancer Site - When a Biomarker Defines the Indication. N Engl J Med. 2017; 377:1409–12. 10.1056/NEJMp1709968 29020592

[54] Hajiran A, Chakiryan N, Aydin AM, Zemp L, Nguyen J, Laborde JM, Chahoud J, Spiess PE, Zaman S, Falasiri S, Fournier M, Teer JK, Dhillon J, et al. Reconnaissance of tumor immune microenvironment spatial heterogeneity in metastatic renal cell carcinoma and correlation with immunotherapy response. Clin Exp Immunol. 2021; 204:96–106. 10.1111/cei.13567 33346915 PMC7944355

