# Supplementary material for: Identification of immunotherapy-related subtypes, characterization of tumor microenvironment infiltration, and development of a prognostic signature in gastric carcinoma
Source: Aging (Albany NY). 2024 Jun 25;16(14):11185–207. doi: 10.18632/aging.205968 (PMC11315391; doi:10.18632/aging.205968)
Supplement: Supplementary Table 1 [file aging-16-205968-s001.pdf]

SUPPLEMENTARY TABLE

Supplementary Table 1. Differentially expressed genes between the responder and non-responder groups.

Gene symbol	Full name
PRKCG	protein kinase C gamma
RCL1	RNA terminal phosphate cyclase like 1
MAGED1	MAGE family member D1
HSBP1L1	heat shock factor binding protein 1 like 1
FBLN1	fibulin 1
GBP1P1	guanylate binding protein 1 pseudogene 1
GBP4	guanylate binding protein 4
GBP5	guanylate binding protein 5
FASLG	Fas ligand
GRIN1	glutamate ionotropic receptor NMDA type subunit 1
RAET1K	retinoic acid early transcript 1K pseudogene
RPL22L1	ribosomal protein L22 like 1
WARS1	tryptophanyl-tRNA synthetase 1
PDGFRA	platelet derived growth factor receptor alpha
TNFSF9	TNF superfamily member 9
ZNF232	zinc finger protein 232
TTC28	tetratricopeptide repeat domain 28
OR2I1P	olfactory receptor family 2 subfamily I member 1 pseudogene
CENPU	centromere protein U
LRRC42	leucine rich repeat containing 42
LAP3	leucine aminopeptidase 3
JAKMIP1	janus kinase and microtubule interacting protein 1
VSNL1	visinin like 1
SKAP2	src kinase associated phosphoprotein 2
CYSRT1	cysteine rich tail 1
USP43	ubiquitin specific peptidase 43
NID1	nidogen 1
CXCL11	C-X-C motif chemokine ligand 11
UBE2L6	ubiquitin conjugating enzyme E2 L6
PMAIP1	phorbol-12-myristate-13-acetate-induced protein 1
ZNF605	zinc finger protein 605
GNAI1	G protein subunit alpha i1
CXCR2P1	C-X-C motif chemokine receptor 2 pseudogene 1
EML1	EMAP like 1
TEKT1	tektin 1
IFNG	interferon gamma
GCHFR	GTP cyclohydrolase I feedback regulator
PID1	phosphotyrosine interaction domain containing 1
SYCE2	synaptonemal complex central element protein 2
FAT4	FAT atypical cadherin 4
CD274	CD274 molecule
FZD3	frizzled class receptor 3
NDRG1	N-myc downstream regulated 1

BATF2	basic leucine zipper ATF-like transcription factor 2
SLC31A2	solute carrier family 31 member 2
CXCL9	C-X-C motif chemokine ligand 9
DCHS1	dachsous cadherin-related 1
LGALS17A	galectin 14 pseudogene
RTKN2	rhotekin 2
CENPH	centromere protein H
CPQ	carboxypeptidase Q
FRMD5	FERM domain containing 5
IDO1	indoleamine 2,3-dioxygenase 1
KDSR	3-ketodihydrosphingosine reductase
ELOVL3	ELOVL fatty acid elongase 3
ARHGAP28	Rho GTPase activating protein 28
SLCO2A1	solute carrier organic anion transporter family member 2A1
MMP2	matrix metallopeptidase 2
MPHOSPH6	M-phase phosphoprotein 6
DNAAF3	dynein axonemal assembly factor 3
PRXL2A	peroxiredoxin like 2A
CXCL10	C-X-C motif chemokine ligand 10
CADM4	cell adhesion molecule 4
