# Supplementary material for: Identification of immunotherapy-related subtypes, characterization of tumor microenvironment infiltration, and development of a prognostic signature in gastric carcinoma
Source: Aging (Albany NY). 2024 Jun 25;16(14):11185–207. doi: 10.18632/aging.205968 (PMC11315391; doi:10.18632/aging.205968)
Supplement: Supplementary Table 2 [file aging-16-205968-s002.docx]

| **Supplementary Table 2. DEGs between cluster 1 and cluster 2 subgroups.** | | | | | |
| --- | --- | --- | --- | --- | --- |
| Gene symbol | Mean1 | Mean2 | logFC | *P*-value | FDR |
| CYGB | 8.117524 | 15.66554 | 0.948483 | 2.78E-24 | 8.29E-23 |
| AL139089.1 | 1.299554 | 0.864729 | -0.5877 | 1.23E-06 | 4.25E-06 |
| FAM19A5 | 1.290514 | 3.532921 | 1.452915 | 1.24E-24 | 3.88E-23 |
| IGKV1D-17 | 1.236031 | 2.978416 | 1.26883 | 3.07E-07 | 1.18E-06 |
| SALL2 | 0.515944 | 1.861471 | 1.851158 | 4.01E-32 | 4.54E-30 |
| SCUBE1 | 0.403396 | 0.936997 | 1.215846 | 5.50E-10 | 3.29E-09 |
| SLC5A9 | 0.607944 | 1.043261 | 0.77909 | 1.49E-05 | 4.27E-05 |
| FAM162B | 0.791506 | 1.690649 | 1.094904 | 1.60E-27 | 7.46E-26 |
| MYOCD | 0.617767 | 3.849325 | 2.639471 | 1.82E-25 | 6.41E-24 |
| ITPKB | 5.207677 | 11.0034 | 1.079238 | 9.52E-19 | 1.56E-17 |
| IGKV1OR2-6 | 0.798588 | 3.138063 | 1.974352 | 3.01E-09 | 1.62E-08 |
| DPEP2 | 0.662807 | 1.178123 | 0.829829 | 3.62E-13 | 3.32E-12 |
| LINC00578 | 0.558323 | 2.021517 | 1.856266 | 3.49E-22 | 8.32E-21 |
| BMX | 0.360333 | 0.795697 | 1.142889 | 3.03E-19 | 5.22E-18 |
| CDH3 | 11.32175 | 6.877802 | -0.71908 | 9.63E-05 | 0.000239 |
| CTGF | 65.14065 | 132.3783 | 1.023036 | 2.58E-18 | 4.05E-17 |
| ZNF423 | 0.661617 | 2.162947 | 1.708931 | 6.44E-37 | 3.68E-34 |
| LDHAP5 | 0.669973 | 0.437879 | -0.61357 | 1.31E-06 | 4.53E-06 |
| AMIGO1 | 1.48683 | 2.532281 | 0.768198 | 6.44E-15 | 7.13E-14 |
| IGKV1-16 | 47.26475 | 115.9605 | 1.294797 | 3.63E-08 | 1.62E-07 |
| ADGRL2 | 3.371102 | 6.802148 | 1.01277 | 1.04E-26 | 4.35E-25 |
| TLX1 | 1.801284 | 0.842038 | -1.09707 | 0.001146 | 0.002328 |
| STRIP2 | 2.256864 | 1.225666 | -0.88075 | 1.97E-11 | 1.45E-10 |
| SYDE2 | 1.021043 | 1.601224 | 0.649131 | 9.11E-05 | 0.000227 |
| SOD3 | 27.69982 | 63.57971 | 1.19869 | 8.40E-20 | 1.56E-18 |
| RN7SL600P | 1.425051 | 2.258149 | 0.664127 | 6.35E-05 | 0.000163 |
| AP001189.3 | 0.523955 | 1.626885 | 1.634597 | 2.22E-30 | 1.79E-28 |
| MST1L | 0.858193 | 1.441189 | 0.747886 | 0.002656 | 0.005008 |
| FGL1 | 2.232396 | 3.963282 | 0.828102 | 3.08E-07 | 1.18E-06 |
| TRBV11-2 | 0.529476 | 0.889065 | 0.747723 | 1.34E-06 | 4.62E-06 |
| AL928742.1 | 0.497577 | 1.38381 | 1.475653 | 2.66E-12 | 2.20E-11 |
| AL390208.1 | 0.622739 | 0.934238 | 0.585163 | 5.37E-05 | 0.00014 |
| OR7E47P | 0.846682 | 1.672804 | 0.982375 | 2.83E-16 | 3.60E-15 |
| CD5 | 3.025693 | 4.850036 | 0.68073 | 5.87E-08 | 2.53E-07 |
| AP000580.1 | 1.444417 | 0.89028 | -0.69816 | 0.000127 | 0.000308 |
| COL3A1 | 206.0884 | 480.5979 | 1.221567 | 5.85E-18 | 8.80E-17 |
| AC104619.1 | 0.84176 | 0.533947 | -0.65671 | 1.42E-05 | 4.10E-05 |
| NGFR | 1.181719 | 4.60682 | 1.962884 | 3.28E-18 | 5.10E-17 |
| MAP7D3 | 1.261179 | 2.229452 | 0.821916 | 2.96E-18 | 4.63E-17 |
| RFTN1 | 5.779451 | 10.52832 | 0.865271 | 2.23E-31 | 2.21E-29 |
| ANKRD65 | 1.578767 | 2.499417 | 0.662794 | 1.91E-18 | 3.05E-17 |
| SCRG1 | 0.403668 | 2.367693 | 2.552241 | 9.65E-17 | 1.28E-15 |
| RETN | 0.366589 | 0.84221 | 1.200015 | 0.026932 | 0.040891 |
| KLRB1 | 2.355532 | 3.542623 | 0.588765 | 1.24E-06 | 4.29E-06 |
| TTLL11 | 1.181629 | 1.78314 | 0.593643 | 4.28E-09 | 2.24E-08 |
| ACTC1 | 0.806976 | 2.24182 | 1.474072 | 7.12E-13 | 6.28E-12 |
| INMT | 1.124509 | 4.284176 | 1.929723 | 4.93E-32 | 5.43E-30 |
| RTL5 | 0.546003 | 1.87862 | 1.782693 | 1.09E-35 | 3.50E-33 |
| PLAC9 | 2.133307 | 4.873526 | 1.191874 | 2.04E-19 | 3.59E-18 |
| TCN2 | 6.352109 | 10.10404 | 0.669624 | 1.04E-15 | 1.24E-14 |
| AL451050.2 | 0.816458 | 0.52265 | -0.64353 | 3.88E-10 | 2.38E-09 |
| PDZD2 | 0.625462 | 1.297508 | 1.052748 | 8.17E-29 | 4.76E-27 |
| PPP1R3F | 0.635298 | 0.977859 | 0.622193 | 6.30E-10 | 3.72E-09 |
| HSPE1P4 | 1.111624 | 0.66207 | -0.74761 | 1.01E-08 | 4.97E-08 |
| KCNQ1 | 24.13969 | 15.95248 | -0.59763 | 0.001755 | 0.003433 |
| TAGAP | 2.194633 | 3.855131 | 0.8128 | 1.72E-10 | 1.12E-09 |
| C4A | 1.41398 | 3.758183 | 1.410274 | 1.12E-23 | 3.12E-22 |
| C2orf40 | 1.498015 | 9.554914 | 2.673191 | 3.19E-20 | 6.20E-19 |
| KRT8 | 464.2003 | 309.4386 | -0.58509 | 1.10E-11 | 8.41E-11 |
| NXPE3 | 1.347475 | 2.337856 | 0.794928 | 1.60E-21 | 3.52E-20 |
| IGHV3OR16-8 | 1.401092 | 3.936572 | 1.490388 | 1.20E-11 | 9.11E-11 |
| RNA5SP108 | 0.612573 | 0.396258 | -0.62844 | 3.09E-06 | 1.00E-05 |
| PCDHGB5 | 0.392522 | 0.733499 | 0.902022 | 8.29E-25 | 2.65E-23 |
| P2RX1 | 0.829481 | 1.735677 | 1.065217 | 1.03E-16 | 1.36E-15 |
| HHIPL1 | 0.494 | 0.951795 | 0.946142 | 5.40E-24 | 1.56E-22 |
| FBXO17 | 1.004478 | 2.250413 | 1.163743 | 1.74E-21 | 3.80E-20 |
| AC130371.2 | 0.415721 | 0.675882 | 0.701155 | 4.26E-12 | 3.42E-11 |
| HTR2B | 0.59882 | 1.178612 | 0.976894 | 3.13E-12 | 2.55E-11 |
| CSPG4 | 4.242798 | 11.06193 | 1.382515 | 2.81E-17 | 3.94E-16 |
| CKS2 | 106.8858 | 58.92337 | -0.85916 | 1.51E-22 | 3.78E-21 |
| SELE | 1.113012 | 2.904375 | 1.383759 | 1.65E-11 | 1.23E-10 |
| SEPT4 | 0.581499 | 1.167998 | 1.006188 | 1.83E-29 | 1.21E-27 |
| KL | 0.283837 | 0.883624 | 1.638369 | 1.56E-33 | 2.60E-31 |
| FGFR2 | 7.56879 | 12.59012 | 0.734157 | 0.000606 | 0.001303 |
| APBB1 | 1.451063 | 4.321095 | 1.574287 | 3.19E-36 | 1.20E-33 |
| FES | 2.35865 | 3.973839 | 0.752572 | 4.89E-24 | 1.42E-22 |
| MERTK | 2.623988 | 4.30205 | 0.713264 | 4.57E-17 | 6.28E-16 |
| FAM110D | 0.804584 | 1.841813 | 1.194812 | 1.42E-21 | 3.13E-20 |
| TRAV8-6 | 0.547307 | 0.885882 | 0.694763 | 7.60E-08 | 3.22E-07 |
| IGLC7 | 16.92668 | 43.77568 | 1.37083 | 0.000289 | 0.000659 |
| MAP3K12 | 0.948214 | 1.778992 | 0.907776 | 1.68E-31 | 1.71E-29 |
| IGHD6-25 | 1.487479 | 2.6894 | 0.854415 | 0.000241 | 0.000558 |
| IGHV1-69D | 40.65286 | 128.3983 | 1.659197 | 4.12E-08 | 1.82E-07 |
| AC116347.1 | 3.082936 | 2.001912 | -0.62293 | 2.66E-07 | 1.04E-06 |
| DACT3 | 1.090131 | 4.790334 | 2.135625 | 1.95E-25 | 6.86E-24 |
| EIF4EBP1 | 72.33101 | 47.29042 | -0.61307 | 7.71E-11 | 5.26E-10 |
| COL9A2 | 4.585929 | 2.834451 | -0.69414 | 0.021942 | 0.034011 |
| GJA4 | 4.825069 | 9.566446 | 0.987434 | 2.15E-22 | 5.31E-21 |
| AC015922.3 | 3.503655 | 6.036133 | 0.784764 | 2.62E-14 | 2.73E-13 |
| AEBP1 | 41.77739 | 93.85508 | 1.167712 | 6.25E-23 | 1.61E-21 |
| TBCEL | 1.377091 | 2.455428 | 0.834351 | 1.92E-24 | 5.85E-23 |
| TLR6 | 0.587509 | 0.893718 | 0.605209 | 4.54E-09 | 2.37E-08 |
| EFEMP1 | 8.157052 | 31.99555 | 1.971751 | 3.60E-36 | 1.30E-33 |
| UST | 1.845311 | 2.974088 | 0.688583 | 1.06E-10 | 7.10E-10 |
| RBPMS | 7.565908 | 14.73916 | 0.962069 | 1.28E-16 | 1.69E-15 |
| ANTXR2 | 6.381645 | 9.863761 | 0.62821 | 1.00E-07 | 4.18E-07 |
| MND1 | 3.79477 | 2.279556 | -0.73526 | 1.67E-15 | 1.97E-14 |
| SLC38A4 | 0.627669 | 0.974501 | 0.634659 | 0.002884 | 0.005396 |
| NKX6-2 | 0.350616 | 1.232061 | 1.813111 | 0.000398 | 0.000884 |
| SYDE1 | 3.283156 | 7.72189 | 1.233871 | 3.11E-32 | 3.62E-30 |
| CDC6 | 13.58324 | 7.03857 | -0.94847 | 1.69E-09 | 9.45E-09 |
| SLC12A4 | 3.926031 | 6.068907 | 0.628365 | 1.30E-19 | 2.34E-18 |
| ABCA12 | 1.147938 | 0.597598 | -0.9418 | 0.019345 | 0.030344 |
| LRFN1 | 1.177464 | 1.833286 | 0.638749 | 9.89E-10 | 5.70E-09 |
| GPR174 | 0.647906 | 1.005666 | 0.634296 | 0.000177 | 0.000419 |
| SLC23A1 | 0.400307 | 0.814511 | 1.024827 | 8.73E-07 | 3.11E-06 |
| RGS4 | 0.983155 | 2.633615 | 1.421554 | 7.68E-16 | 9.31E-15 |
| VCAM1 | 3.931176 | 6.638565 | 0.75591 | 4.42E-19 | 7.50E-18 |
| IGKV3-7 | 1.745526 | 4.195934 | 1.26533 | 2.75E-08 | 1.25E-07 |
| NACAD | 0.376403 | 1.243067 | 1.723552 | 3.51E-31 | 3.40E-29 |
| GSTA2 | 1.446781 | 4.799462 | 1.730026 | 2.29E-09 | 1.26E-08 |
| BOC | 0.783011 | 3.938695 | 2.330612 | 1.69E-32 | 2.15E-30 |
| MSRB3 | 3.214232 | 12.3922 | 1.946887 | 1.22E-28 | 6.70E-27 |
| NRP1 | 5.586778 | 9.279302 | 0.732 | 1.12E-19 | 2.03E-18 |
| CPT1C | 0.69084 | 1.151859 | 0.737541 | 3.91E-19 | 6.65E-18 |
| RPL31P12 | 1.033511 | 0.620874 | -0.73518 | 0.000115 | 0.000282 |
| SGIP1 | 0.500522 | 0.942383 | 0.91288 | 2.22E-18 | 3.49E-17 |
| ABCC6 | 1.133423 | 1.747022 | 0.624211 | 2.08E-06 | 6.93E-06 |
| PGM5P4 | 0.302992 | 1.071731 | 1.82259 | 7.28E-18 | 1.08E-16 |
| PODN | 4.579114 | 20.61866 | 2.17081 | 3.09E-39 | 3.74E-36 |
| RIPOR2 | 0.989835 | 2.069537 | 1.064048 | 1.24E-15 | 1.47E-14 |
| AVPR1A | 0.549141 | 1.349588 | 1.29727 | 7.22E-23 | 1.84E-21 |
| GGT5 | 4.3677 | 11.02345 | 1.33563 | 2.22E-29 | 1.45E-27 |
| RPL26P6 | 8.092903 | 5.195029 | -0.63953 | 0.000853 | 0.001783 |
| FBN1 | 5.292294 | 15.47154 | 1.547651 | 9.61E-29 | 5.42E-27 |
| MAP6 | 0.373692 | 1.288054 | 1.785269 | 3.30E-32 | 3.81E-30 |
| PRDM11 | 0.886661 | 1.425076 | 0.684585 | 1.16E-13 | 1.13E-12 |
| VPS9D1-AS1 | 8.57324 | 5.317323 | -0.68914 | 5.05E-12 | 4.04E-11 |
| PEAR1 | 1.383875 | 2.819086 | 1.026514 | 5.34E-24 | 1.55E-22 |
| GP1BA | 0.70005 | 1.086258 | 0.633837 | 3.85E-09 | 2.03E-08 |
| WNT9A | 0.699051 | 2.103005 | 1.588982 | 2.63E-18 | 4.12E-17 |
| EVI2A | 3.003148 | 4.682488 | 0.6408 | 1.73E-10 | 1.12E-09 |
| KCNH3 | 0.522485 | 0.851487 | 0.704595 | 1.37E-06 | 4.72E-06 |
| SPINK4 | 67.29489 | 144.62 | 1.103698 | 0.011202 | 0.018496 |
| SH3BGR | 1.129105 | 2.347521 | 1.055959 | 2.21E-06 | 7.34E-06 |
| DAB2 | 6.368817 | 12.54574 | 0.9781 | 5.37E-26 | 2.08E-24 |
| CPXM1 | 4.691788 | 11.26458 | 1.263584 | 3.73E-15 | 4.22E-14 |
| SLC18A2 | 0.457233 | 1.304017 | 1.51196 | 1.67E-13 | 1.60E-12 |
| ANTXR1 | 12.75347 | 30.23974 | 1.245556 | 1.87E-24 | 5.68E-23 |
| LPAR1 | 2.353194 | 5.309398 | 1.173928 | 1.76E-25 | 6.27E-24 |
| KCND1 | 0.501188 | 1.088244 | 1.118578 | 6.47E-28 | 3.18E-26 |
| TNFSF4 | 1.001371 | 1.567706 | 0.646679 | 4.01E-09 | 2.11E-08 |
| TMEM100 | 0.379948 | 1.889193 | 2.313896 | 6.75E-28 | 3.30E-26 |
| PAICS | 22.66248 | 14.46072 | -0.64817 | 2.74E-18 | 4.29E-17 |
| AC005884.2 | 4.46284 | 2.635397 | -0.75994 | 3.91E-05 | 0.000105 |
| IGKV2-29 | 4.275923 | 12.63522 | 1.563143 | 0.002869 | 0.005369 |
| CX3CR1 | 0.375447 | 1.3736 | 1.87128 | 1.62E-24 | 5.00E-23 |
| AMH | 2.328093 | 1.338349 | -0.79869 | 3.00E-05 | 8.17E-05 |
| MN1 | 1.445294 | 5.314329 | 1.878525 | 2.83E-28 | 1.47E-26 |
| IGKV1-9 | 50.43757 | 92.76803 | 0.879129 | 7.60E-09 | 3.83E-08 |
| AC104046.1 | 0.717899 | 0.455952 | -0.6549 | 9.22E-05 | 0.00023 |
| IGLV1-41 | 2.164789 | 5.003516 | 1.208716 | 4.74E-08 | 2.07E-07 |
| LINC01235 | 0.595959 | 1.120814 | 0.911262 | 8.84E-08 | 3.70E-07 |
| GALNT15 | 0.428262 | 1.379623 | 1.68771 | 1.99E-22 | 4.94E-21 |
| FAM151A | 0.488394 | 0.880528 | 0.850324 | 0.001135 | 0.002308 |
| AC091179.1 | 0.681756 | 1.582588 | 1.214959 | 0.028567 | 0.043126 |
| SEMA6D | 0.720869 | 1.416964 | 0.974994 | 4.50E-18 | 6.87E-17 |
| CXCL12 | 4.256311 | 10.77038 | 1.339394 | 4.75E-26 | 1.85E-24 |
| MINCR | 3.40054 | 2.097857 | -0.69685 | 1.37E-11 | 1.03E-10 |
| NAP1L3 | 0.284578 | 1.052614 | 1.887081 | 2.81E-33 | 4.33E-31 |
| SELP | 1.357585 | 5.119468 | 1.914952 | 3.66E-32 | 4.19E-30 |
| CACNG4 | 1.453209 | 2.503301 | 0.78459 | 6.07E-10 | 3.60E-09 |
| DSG1 | 0.843654 | 0.29001 | -1.54055 | 0.007446 | 0.012778 |
| AC004816.1 | 2.605738 | 1.471202 | -0.8247 | 2.26E-11 | 1.66E-10 |
| RASD2 | 1.440399 | 3.38622 | 1.233207 | 1.81E-23 | 4.97E-22 |
| COPZ2 | 2.529705 | 5.518429 | 1.125289 | 3.96E-20 | 7.62E-19 |
| MTCO2P22 | 6.831259 | 4.34113 | -0.65408 | 0.001376 | 0.002754 |
| IGLV1-47 | 99.16067 | 179.5133 | 0.856251 | 3.47E-08 | 1.55E-07 |
| BX293535.1 | 0.419326 | 0.680887 | 0.699341 | 0.000574 | 0.00124 |
| TLR4 | 2.508607 | 3.773779 | 0.589124 | 6.69E-11 | 4.60E-10 |
| F2 | 0.225829 | 1.223123 | 2.437265 | 0.001495 | 0.002969 |
| SGCA | 0.916716 | 3.47236 | 1.921369 | 2.05E-18 | 3.25E-17 |
| ANO5 | 0.695541 | 1.343303 | 0.949578 | 4.32E-14 | 4.36E-13 |
| DERL3 | 4.770973 | 8.221831 | 0.785176 | 3.67E-05 | 9.86E-05 |
| MAGI2-AS3 | 0.507986 | 1.419537 | 1.48256 | 5.47E-33 | 7.80E-31 |
| TREM2 | 7.355512 | 11.1484 | 0.59994 | 5.20E-07 | 1.91E-06 |
| POPDC3 | 0.326464 | 0.698806 | 1.097968 | 5.19E-12 | 4.14E-11 |
| LINC00702 | 0.193428 | 1.190151 | 2.621272 | 1.67E-19 | 2.97E-18 |
| MRC2 | 8.580163 | 18.67593 | 1.122103 | 6.39E-22 | 1.49E-20 |
| WDFY4 | 1.130397 | 2.086335 | 0.884142 | 1.80E-14 | 1.90E-13 |
| ERICH5 | 1.539655 | 2.734089 | 0.828454 | 2.13E-08 | 9.93E-08 |
| SLC13A2 | 1.775584 | 4.878444 | 1.458127 | 6.63E-08 | 2.84E-07 |
| CLDN5 | 3.301364 | 6.931758 | 1.070159 | 1.71E-21 | 3.75E-20 |
| GAPT | 0.690069 | 1.580306 | 1.195392 | 9.43E-20 | 1.74E-18 |
| TRBV24-1 | 0.473931 | 0.772162 | 0.704226 | 0.0001 | 0.000248 |
| JAK3 | 3.708096 | 5.702159 | 0.62083 | 6.22E-10 | 3.68E-09 |
| SNRPFP1 | 2.254837 | 1.432378 | -0.65461 | 9.54E-07 | 3.37E-06 |
| CAPN6 | 6.429135 | 12.55178 | 0.965196 | 2.76E-14 | 2.86E-13 |
| UCN3 | 0.476176 | 6.696879 | 3.813923 | 3.80E-05 | 0.000102 |
| ADH1B | 0.905907 | 6.865848 | 2.922002 | 6.05E-27 | 2.62E-25 |
| MS4A6A | 4.646364 | 7.334364 | 0.65857 | 5.06E-11 | 3.55E-10 |
| FAM110B | 0.539705 | 1.690289 | 1.647027 | 1.42E-32 | 1.85E-30 |
| SH3PXD2B | 6.38611 | 12.13528 | 0.926198 | 3.46E-21 | 7.28E-20 |
| ANXA6 | 12.96108 | 25.5621 | 0.97982 | 7.31E-26 | 2.79E-24 |
| EHD2 | 25.54043 | 52.8995 | 1.050471 | 5.01E-23 | 1.30E-21 |
| PXDN | 6.168451 | 11.56078 | 0.906259 | 6.21E-18 | 9.31E-17 |
| SLC7A2 | 2.51734 | 9.922367 | 1.978784 | 1.07E-18 | 1.74E-17 |
| COL12A1 | 16.97312 | 33.6885 | 0.989004 | 6.35E-13 | 5.64E-12 |
| HMGA1P5 | 0.869757 | 1.343775 | 0.627606 | 0.016088 | 0.025681 |
| SDK1 | 1.184373 | 2.006727 | 0.760722 | 8.60E-17 | 1.15E-15 |
| PPP1R1B | 74.28455 | 27.71818 | -1.42223 | 0.002622 | 0.004954 |
| TF | 0.663382 | 3.581358 | 2.432594 | 1.43E-10 | 9.39E-10 |
| TMTC1 | 0.807921 | 2.944667 | 1.865818 | 2.20E-31 | 2.20E-29 |
| CHAD | 0.598856 | 1.285012 | 1.101501 | 1.40E-07 | 5.69E-07 |
| COL1A1 | 325.2079 | 569.1354 | 0.807409 | 2.29E-07 | 8.99E-07 |
| PCDH17 | 1.922028 | 3.780162 | 0.975819 | 3.63E-24 | 1.07E-22 |
| AF001548.3 | 0.2715 | 1.377601 | 2.343137 | 5.52E-09 | 2.85E-08 |
| ARHGEF17 | 3.867121 | 8.777702 | 1.182583 | 1.41E-34 | 2.98E-32 |
| SMPD1 | 8.453542 | 13.19 | 0.641817 | 2.03E-23 | 5.54E-22 |
| SOGA1 | 4.346469 | 7.134597 | 0.714988 | 4.86E-18 | 7.39E-17 |
| AL109741.1 | 0.449475 | 0.954243 | 1.086117 | 1.65E-22 | 4.10E-21 |
| S100A1 | 0.81697 | 1.696584 | 1.054279 | 2.38E-07 | 9.30E-07 |
| AC068152.1 | 1.536752 | 2.34813 | 0.611628 | 4.58E-13 | 4.15E-12 |
| RTN4IP1 | 6.45359 | 4.027237 | -0.68031 | 6.25E-05 | 0.000161 |
| TMEM200B | 0.875057 | 2.177793 | 1.315418 | 1.11E-29 | 7.70E-28 |
| PCNA | 126.7749 | 80.68532 | -0.65189 | 5.13E-17 | 7.00E-16 |
| AL122035.1 | 1.265095 | 2.288612 | 0.855227 | 3.52E-16 | 4.42E-15 |
| PALMD | 0.939653 | 1.777278 | 0.91947 | 5.51E-23 | 1.42E-21 |
| ATP2B4 | 11.26677 | 28.79252 | 1.35362 | 7.50E-25 | 2.41E-23 |
| PLIN1 | 0.245251 | 1.423166 | 2.536772 | 0.014865 | 0.023914 |
| ZSCAN18 | 0.708588 | 1.978021 | 1.481038 | 6.09E-29 | 3.67E-27 |
| EDNRA | 3.217374 | 8.184201 | 1.346958 | 2.00E-27 | 9.04E-26 |
| KLF9 | 7.291832 | 17.7487 | 1.28336 | 2.21E-26 | 8.79E-25 |
| AC018752.1 | 0.786023 | 1.363536 | 0.794708 | 1.72E-12 | 1.45E-11 |
| CCL26 | 2.004321 | 5.641013 | 1.49284 | 0.005247 | 0.009304 |
| TMEM121 | 0.706513 | 1.651296 | 1.224812 | 4.22E-24 | 1.23E-22 |
| NMNAT2 | 0.679263 | 1.260728 | 0.892214 | 3.13E-12 | 2.55E-11 |
| ROBO4 | 2.286423 | 4.151893 | 0.860677 | 8.24E-22 | 1.89E-20 |
| AC100803.2 | 0.381226 | 0.988178 | 1.374124 | 1.29E-24 | 4.01E-23 |
| TRAV29DV5 | 0.677132 | 1.054779 | 0.639431 | 3.72E-06 | 1.19E-05 |
| MMP19 | 1.962148 | 4.534404 | 1.208479 | 1.50E-27 | 7.06E-26 |
| MYOM1 | 0.643774 | 1.707019 | 1.406852 | 1.10E-15 | 1.31E-14 |
| FAM124B | 0.294279 | 0.778109 | 1.402787 | 4.95E-29 | 3.03E-27 |
| IGKV1-39 | 7.275917 | 19.76664 | 1.441867 | 1.43E-10 | 9.39E-10 |
| SMIM10 | 1.504968 | 3.66627 | 1.284581 | 9.30E-29 | 5.28E-27 |
| DSEL | 0.268185 | 0.815685 | 1.604781 | 5.92E-30 | 4.38E-28 |
| SLCO2B1 | 6.840744 | 10.71602 | 0.647544 | 6.11E-11 | 4.23E-10 |
| TMPRSS11B | 2.800753 | 0.026814 | -6.70671 | 0.031094 | 0.046607 |
| AC053503.4 | 0.360593 | 2.037745 | 2.498531 | 3.27E-13 | 3.03E-12 |
| SALL4 | 1.546041 | 2.69091 | 0.799516 | 7.75E-05 | 0.000196 |
| GPR34 | 2.914644 | 5.611351 | 0.945028 | 1.91E-16 | 2.48E-15 |
| GCNT4 | 1.164902 | 1.783331 | 0.614366 | 3.79E-07 | 1.43E-06 |
| AC123912.1 | 0.392248 | 0.733261 | 0.902561 | 1.59E-08 | 7.56E-08 |
| EPAS1 | 25.42262 | 39.94914 | 0.652051 | 2.26E-21 | 4.85E-20 |
| PI3 | 403.1232 | 200.2681 | -1.00929 | 0.031708 | 0.047396 |
| VTN | 7.078458 | 20.19296 | 1.512345 | 1.75E-20 | 3.46E-19 |
| FAM180A | 0.398372 | 1.414502 | 1.828105 | 8.01E-28 | 3.86E-26 |
| KRT19 | 509.4335 | 324.0993 | -0.65246 | 9.67E-09 | 4.79E-08 |
| UCA1 | 16.15866 | 10.32169 | -0.64663 | 0.000399 | 0.000887 |
| NRP2 | 3.232624 | 6.876445 | 1.088957 | 1.82E-16 | 2.37E-15 |
| DUSP1 | 72.65845 | 136.3913 | 0.908549 | 8.51E-16 | 1.02E-14 |
| TRBV5-4 | 0.498271 | 0.83976 | 0.753047 | 2.73E-06 | 8.95E-06 |
| PEG10 | 4.343307 | 12.41519 | 1.515241 | 2.12E-09 | 1.17E-08 |
| CTSO | 8.966817 | 15.25433 | 0.766551 | 9.18E-21 | 1.85E-19 |
| KCNJ8 | 2.704794 | 5.158692 | 0.931487 | 3.27E-23 | 8.64E-22 |
| BMP6 | 0.70233 | 1.753153 | 1.31973 | 9.91E-31 | 8.76E-29 |
| THSD1 | 0.861863 | 1.518899 | 0.817495 | 1.77E-20 | 3.49E-19 |
| TRPA1 | 1.170845 | 2.52053 | 1.106177 | 0.003738 | 0.006839 |
| FAM167A | 1.29628 | 3.373875 | 1.38003 | 1.24E-05 | 3.59E-05 |
| PIK3CD | 2.893033 | 4.709794 | 0.703081 | 1.51E-11 | 1.13E-10 |
| PCSK9 | 8.005186 | 4.907867 | -0.70584 | 5.32E-05 | 0.000139 |
| C16orf89 | 0.780551 | 3.878531 | 2.312945 | 5.90E-24 | 1.69E-22 |
| S1PR1 | 5.814979 | 14.91144 | 1.358574 | 2.19E-34 | 4.32E-32 |
| SPARC | 144.6782 | 278.7449 | 0.946098 | 8.05E-16 | 9.75E-15 |
| SLC5A5 | 1.620846 | 4.434307 | 1.451962 | 0.000829 | 0.001738 |
| LYVE1 | 0.753782 | 2.274381 | 1.593255 | 2.10E-19 | 3.68E-18 |
| SNHG19 | 38.85857 | 24.8252 | -0.64643 | 1.17E-08 | 5.69E-08 |
| RGMA | 1.235238 | 6.632645 | 2.424795 | 1.08E-26 | 4.45E-25 |
| NTN4 | 5.655066 | 9.156457 | 0.695246 | 3.94E-17 | 5.47E-16 |
| NOVA2 | 0.498849 | 0.952607 | 0.933279 | 3.10E-24 | 9.23E-23 |
| SYNE3 | 0.493148 | 0.87594 | 0.828812 | 5.32E-20 | 1.01E-18 |
| MYB | 7.78856 | 3.672332 | -1.08466 | 7.31E-14 | 7.24E-13 |
| PLXNA4 | 0.325342 | 0.994432 | 1.611914 | 1.60E-27 | 7.46E-26 |
| RPS2P55 | 3.886421 | 2.58017 | -0.59098 | 9.64E-07 | 3.40E-06 |
| ZIC5 | 1.210118 | 0.582894 | -1.05384 | 0.023222 | 0.03578 |
| C1GALT1C1L | 0.446316 | 0.801044 | 0.843818 | 3.71E-08 | 1.65E-07 |
| C11orf96 | 16.13375 | 39.46134 | 1.290358 | 8.53E-21 | 1.73E-19 |
| MFAP2 | 6.012408 | 11.21644 | 0.8996 | 1.77E-12 | 1.49E-11 |
| AC006329.1 | 4.725653 | 2.458413 | -0.94279 | 3.12E-11 | 2.24E-10 |
| GLT8D2 | 1.996537 | 4.817972 | 1.270926 | 1.14E-28 | 6.34E-27 |
| TWIST1 | 1.646095 | 2.628521 | 0.675203 | 4.60E-06 | 1.44E-05 |
| COL4A6 | 0.486179 | 1.190954 | 1.29256 | 4.42E-13 | 4.02E-12 |
| RBMS3 | 0.695754 | 2.284726 | 1.715372 | 2.99E-35 | 8.44E-33 |
| ZNF518B | 0.99413 | 1.56789 | 0.657317 | 5.71E-12 | 4.52E-11 |
| GREM1 | 11.82336 | 37.31373 | 1.658066 | 9.28E-23 | 2.36E-21 |
| ZEB2 | 1.265763 | 2.999102 | 1.244523 | 8.25E-29 | 4.80E-27 |
| MPDZ | 0.717401 | 2.470378 | 1.783881 | 3.85E-35 | 1.04E-32 |
| TMEM119 | 4.374425 | 13.67504 | 1.64438 | 3.79E-30 | 2.91E-28 |
| ZNF347 | 0.693479 | 1.195334 | 0.785489 | 1.46E-13 | 1.40E-12 |
| IGHV3-19 | 1.032151 | 3.280884 | 1.66843 | 3.69E-11 | 2.63E-10 |
| CSF1R | 7.363666 | 15.1333 | 1.03923 | 4.82E-18 | 7.34E-17 |
| PRKD1 | 0.51512 | 1.407786 | 1.450448 | 7.40E-31 | 6.60E-29 |
| KLRG1 | 0.464671 | 0.796945 | 0.77827 | 3.25E-13 | 3.01E-12 |
| ESAM | 10.62854 | 16.10219 | 0.599313 | 2.63E-15 | 3.01E-14 |
| HSPD1P1 | 1.103158 | 0.710095 | -0.63556 | 1.07E-10 | 7.14E-10 |
| CATSPERB | 1.19018 | 0.756731 | -0.65333 | 0.011202 | 0.018496 |
| CCNB1 | 25.30903 | 16.16914 | -0.64641 | 7.15E-14 | 7.10E-13 |
| APBA2 | 0.642396 | 1.240281 | 0.949132 | 7.80E-17 | 1.05E-15 |
| PDE1B | 0.351597 | 0.910181 | 1.372232 | 3.63E-24 | 1.07E-22 |
| ADGRD1 | 0.243051 | 1.220696 | 2.328373 | 2.18E-38 | 1.85E-35 |
| SEMA3E | 0.716007 | 1.531527 | 1.096924 | 1.93E-10 | 1.24E-09 |
| IGKV2OR22-3 | 0.323672 | 1.253284 | 1.95311 | 1.47E-07 | 5.94E-07 |
| FOLR2 | 4.454901 | 10.4336 | 1.227772 | 1.22E-15 | 1.45E-14 |
| SYCE2 | 0.795876 | 0.428021 | -0.89486 | 1.84E-05 | 5.21E-05 |
| AP000808.2 | 0.328417 | 0.672992 | 1.035062 | 1.36E-12 | 1.16E-11 |
| MIR7848 | 0.56519 | 0.860504 | 0.606446 | 0.004415 | 0.00795 |
| KRT16 | 21.86846 | 3.594028 | -2.60518 | 3.45E-05 | 9.32E-05 |
| SELENOM | 14.6706 | 29.21756 | 0.993908 | 2.30E-17 | 3.24E-16 |
| PDGFRA | 3.469149 | 10.44291 | 1.58987 | 2.52E-40 | 6.60E-37 |
| RNA5SP111 | 1.38613 | 2.321741 | 0.744145 | 0.000959 | 0.001985 |
| DZIP1L | 1.174327 | 2.123843 | 0.854843 | 1.88E-15 | 2.20E-14 |
| FGD2 | 0.906175 | 1.419359 | 0.647379 | 1.03E-09 | 5.91E-09 |
| SAMD14 | 0.37539 | 0.739087 | 0.977354 | 9.46E-24 | 2.66E-22 |
| PROS1 | 4.773214 | 8.119908 | 0.766503 | 1.88E-17 | 2.68E-16 |
| DMD | 0.689341 | 2.66165 | 1.949032 | 1.10E-25 | 4.05E-24 |
| MYCT1 | 1.580831 | 3.329509 | 1.074626 | 7.31E-31 | 6.60E-29 |
| COL2A1 | 0.36619 | 3.202124 | 3.128363 | 0.001177 | 0.002385 |
| IGLV5-52 | 0.293083 | 0.779968 | 1.412108 | 2.76E-05 | 7.58E-05 |
| GPR63 | 0.440872 | 0.67683 | 0.618433 | 1.66E-12 | 1.40E-11 |
| ATP4B | 1.814001 | 18.63293 | 3.360608 | 0.005932 | 0.010408 |
| CYBRD1 | 9.283231 | 30.93715 | 1.736641 | 6.72E-37 | 3.68E-34 |
| AL118505.1 | 1.487152 | 0.98942 | -0.5879 | 1.33E-07 | 5.42E-07 |
| PCDHB6 | 0.396255 | 0.614588 | 0.633189 | 9.72E-17 | 1.29E-15 |
| COL23A1 | 0.524137 | 0.867571 | 0.727037 | 5.09E-17 | 6.96E-16 |
| GNAO1 | 0.713036 | 3.363605 | 2.237961 | 1.97E-26 | 7.90E-25 |
| NEFH | 0.208435 | 1.27389 | 2.611569 | 1.90E-18 | 3.03E-17 |
| AC005256.1 | 1.867852 | 0.852811 | -1.13108 | 0.000121 | 0.000295 |
| TNFRSF8 | 0.401116 | 0.633772 | 0.659944 | 1.10E-08 | 5.41E-08 |
| SYNDIG1 | 1.06968 | 2.477893 | 1.211935 | 9.02E-20 | 1.67E-18 |
| THBD | 6.587065 | 13.49671 | 1.0349 | 3.85E-29 | 2.42E-27 |
| ZNF521 | 0.88789 | 2.559668 | 1.527504 | 2.57E-38 | 2.08E-35 |
| FGF10 | 0.279467 | 1.683558 | 2.590764 | 5.28E-28 | 2.63E-26 |
| IGHV3-73 | 31.08379 | 49.7149 | 0.677516 | 1.70E-06 | 5.78E-06 |
| IGKV1-27 | 38.80858 | 79.80646 | 1.04013 | 4.56E-07 | 1.69E-06 |
| MYLK | 5.311125 | 30.24195 | 2.509462 | 2.75E-27 | 1.22E-25 |
| DENND5A | 3.223901 | 6.514424 | 1.01483 | 4.26E-28 | 2.16E-26 |
| MIR3186 | 0.35969 | 0.715768 | 0.99274 | 2.82E-06 | 9.21E-06 |
| RPL7P9 | 34.78631 | 22.20618 | -0.64756 | 4.68E-08 | 2.05E-07 |
| FOXC2 | 0.848288 | 1.497554 | 0.819983 | 2.34E-12 | 1.95E-11 |
| SERPINE2 | 3.236383 | 6.62238 | 1.032967 | 1.54E-20 | 3.06E-19 |
| SMTN | 9.19969 | 24.76441 | 1.428611 | 2.09E-13 | 1.97E-12 |
| GFPT2 | 1.380285 | 3.600365 | 1.383177 | 1.90E-19 | 3.37E-18 |
| KRT18P17 | 0.712112 | 0.415179 | -0.77837 | 1.88E-11 | 1.39E-10 |
| SERP2 | 0.662151 | 1.116976 | 0.754366 | 6.12E-14 | 6.11E-13 |
| ACACB | 1.384629 | 2.893169 | 1.063151 | 3.49E-22 | 8.32E-21 |
| NAV1 | 2.278505 | 3.517092 | 0.626295 | 3.41E-16 | 4.29E-15 |
| AL450405.1 | 46.35956 | 26.52949 | -0.80527 | 1.18E-10 | 7.81E-10 |
| GIPC3 | 1.193824 | 1.997544 | 0.742638 | 7.48E-20 | 1.40E-18 |
| ZNF528-AS1 | 0.578406 | 1.05649 | 0.869124 | 3.82E-20 | 7.37E-19 |
| AL354707.1 | 2.226451 | 1.300367 | -0.77583 | 1.30E-05 | 3.76E-05 |
| EDA2R | 0.333037 | 0.665674 | 0.999133 | 1.32E-20 | 2.63E-19 |
| MYRIP | 0.455842 | 0.891203 | 0.96722 | 5.37E-16 | 6.60E-15 |
| IGKV2OR22-4 | 0.758916 | 3.152496 | 2.054483 | 1.42E-08 | 6.85E-08 |
| SEZ6 | 0.256267 | 0.835763 | 1.705444 | 2.96E-08 | 1.34E-07 |
| JAML | 0.908624 | 1.604356 | 0.820239 | 2.38E-13 | 2.24E-12 |
| SNCAIP | 0.966123 | 1.90166 | 0.976981 | 2.13E-23 | 5.79E-22 |
| LOX | 6.237334 | 10.54677 | 0.7578 | 1.14E-09 | 6.56E-09 |
| PKP3 | 48.02114 | 31.645 | -0.60169 | 7.26E-14 | 7.19E-13 |
| SEC14L4 | 0.852219 | 1.317881 | 0.628924 | 5.26E-07 | 1.94E-06 |
| TMEM136 | 0.465042 | 0.716374 | 0.623352 | 7.11E-18 | 1.06E-16 |
| PARVA | 8.608605 | 16.15863 | 0.908453 | 4.01E-25 | 1.34E-23 |
| HLX | 0.894308 | 1.534959 | 0.779357 | 7.80E-18 | 1.15E-16 |
| IGHV3-72 | 12.79526 | 26.79313 | 1.066253 | 1.60E-07 | 6.42E-07 |
| FCN1 | 0.463581 | 1.29568 | 1.482815 | 5.24E-18 | 7.93E-17 |
| AL139393.2 | 0.983898 | 1.719345 | 0.805278 | 2.22E-10 | 1.40E-09 |
| FGFR1 | 3.140423 | 6.926798 | 1.141229 | 1.70E-33 | 2.79E-31 |
| RASSF8-AS1 | 0.365856 | 1.125383 | 1.621068 | 6.61E-30 | 4.83E-28 |
| MMP12 | 41.82877 | 25.09094 | -0.73733 | 2.91E-05 | 7.95E-05 |
| BATF2 | 12.54326 | 6.037096 | -1.05499 | 1.53E-07 | 6.18E-07 |
| LINC00982 | 0.691541 | 1.197437 | 0.792063 | 5.68E-08 | 2.45E-07 |
| TPM1 | 15.65403 | 35.544 | 1.183072 | 1.63E-10 | 1.06E-09 |
| GPBAR1 | 1.103006 | 3.209758 | 1.541024 | 1.79E-24 | 5.49E-23 |
| PLXND1 | 11.80497 | 18.35885 | 0.637081 | 5.52E-20 | 1.05E-18 |
| ATP10A | 1.151452 | 1.798979 | 0.643725 | 3.02E-17 | 4.23E-16 |
| AC036108.3 | 0.227315 | 1.047861 | 2.204681 | 8.29E-10 | 4.83E-09 |
| ELMO1 | 2.232247 | 3.700481 | 0.729216 | 5.89E-25 | 1.92E-23 |
| DENND2A | 0.874537 | 1.917441 | 1.13259 | 2.04E-29 | 1.35E-27 |
| ITGA8 | 1.238419 | 4.301299 | 1.796274 | 1.63E-21 | 3.58E-20 |
| VSNL1 | 7.14863 | 1.959406 | -1.86725 | 1.47E-09 | 8.31E-09 |
| VEGFC | 2.614417 | 4.693291 | 0.84411 | 2.46E-22 | 6.01E-21 |
| AL356653.1 | 2.888538 | 1.787379 | -0.69249 | 7.32E-11 | 5.01E-10 |
| TMEM236 | 0.316282 | 0.699816 | 1.145764 | 2.56E-05 | 7.05E-05 |
| KCNS3 | 2.629126 | 4.313593 | 0.714307 | 1.43E-14 | 1.53E-13 |
| LINC00861 | 0.369307 | 0.725185 | 0.973528 | 9.73E-09 | 4.81E-08 |
| C6orf223 | 4.882947 | 2.718364 | -0.84501 | 4.46E-07 | 1.66E-06 |
| NYNRIN | 4.703543 | 7.165405 | 0.607301 | 3.11E-12 | 2.54E-11 |
| LINC00392 | 2.663286 | 5.001804 | 0.909241 | 0.016877 | 0.026818 |
| ADAMTSL4-AS1 | 0.428738 | 0.778647 | 0.860871 | 5.04E-08 | 2.19E-07 |
| TSPAN18 | 3.804704 | 10.39672 | 1.450272 | 2.44E-32 | 2.92E-30 |
| IGLC2 | 464.2993 | 809.8484 | 0.802597 | 7.34E-06 | 2.22E-05 |
| ESCO2 | 2.752614 | 1.672342 | -0.71893 | 8.01E-12 | 6.21E-11 |
| LINC02178 | 1.760297 | 0.084546 | -4.37994 | 2.33E-05 | 6.47E-05 |
| TM6SF2 | 1.626745 | 2.545106 | 0.645738 | 0.000973 | 0.00201 |
| NTN1 | 2.43584 | 7.585207 | 1.638769 | 1.21E-21 | 2.71E-20 |
| DUBR | 0.431638 | 0.731835 | 0.761696 | 4.17E-18 | 6.39E-17 |
| GNAI1 | 2.915753 | 4.963989 | 0.767631 | 1.58E-18 | 2.54E-17 |
| STARD8 | 1.082065 | 2.235948 | 1.047099 | 9.22E-30 | 6.51E-28 |
| VSTM2L | 3.189283 | 6.10788 | 0.93744 | 2.29E-18 | 3.60E-17 |
| IGHV3-53 | 13.95241 | 35.0204 | 1.327681 | 3.51E-08 | 1.57E-07 |
| ARMC9 | 1.313371 | 2.147027 | 0.709066 | 1.12E-18 | 1.82E-17 |
| DLG4 | 1.143552 | 2.362546 | 1.04682 | 2.75E-30 | 2.16E-28 |
| RNASE6 | 11.74352 | 19.51098 | 0.732421 | 6.27E-11 | 4.34E-10 |
| OMD | 0.861166 | 4.696527 | 2.44723 | 8.44E-26 | 3.18E-24 |
| PLXNC1 | 2.180917 | 4.135544 | 0.923142 | 3.07E-20 | 5.99E-19 |
| FAM13C | 0.300857 | 0.950384 | 1.659431 | 6.77E-35 | 1.69E-32 |
| FAM129A | 4.727654 | 15.91247 | 1.750962 | 2.50E-21 | 5.34E-20 |
| IGHV1OR15-9 | 0.956278 | 4.154819 | 2.119284 | 1.03E-10 | 6.90E-10 |
| IGKV3-11 | 144.1532 | 272.7195 | 0.919815 | 4.88E-08 | 2.13E-07 |
| GRHL3 | 2.546416 | 0.791747 | -1.68536 | 2.42E-05 | 6.70E-05 |
| SNCA | 0.425556 | 0.899122 | 1.079168 | 1.81E-21 | 3.93E-20 |
| TRIM29 | 16.16157 | 7.222204 | -1.16206 | 0.001648 | 0.003243 |
| SCN7A | 0.132094 | 0.91272 | 2.788606 | 5.45E-22 | 1.28E-20 |
| AC079140.2 | 31.53969 | 19.71799 | -0.67766 | 4.82E-06 | 1.50E-05 |
| SLC24A3 | 2.219081 | 5.624191 | 1.341683 | 5.76E-29 | 3.49E-27 |
| CYP2U1 | 1.092679 | 2.208663 | 1.015303 | 7.62E-24 | 2.17E-22 |
| FAM78A | 1.637764 | 2.468488 | 0.5919 | 4.06E-10 | 2.48E-09 |
| MS4A7 | 4.294138 | 7.192529 | 0.74413 | 1.10E-12 | 9.54E-12 |
| CXCR4 | 26.34157 | 48.17549 | 0.870958 | 5.97E-15 | 6.63E-14 |
| SRPX | 2.327338 | 8.746815 | 1.910077 | 4.15E-29 | 2.59E-27 |
| HSPA2 | 1.898118 | 3.994048 | 1.073282 | 8.03E-20 | 1.50E-18 |
| NR2F2 | 12.84124 | 22.86171 | 0.832148 | 7.11E-18 | 1.06E-16 |
| AC004080.2 | 1.59556 | 0.866151 | -0.88137 | 0.006265 | 0.010929 |
| HGF | 0.523374 | 1.544459 | 1.561186 | 1.23E-29 | 8.43E-28 |
| NLGN2 | 3.166792 | 6.50001 | 1.03742 | 5.93E-22 | 1.39E-20 |
| AP001024.1 | 7.722253 | 5.079734 | -0.60427 | 2.03E-08 | 9.49E-08 |
| DCN | 12.44422 | 44.06811 | 1.824259 | 3.55E-39 | 4.02E-36 |
| MSC-AS1 | 0.437839 | 0.939649 | 1.101721 | 8.89E-26 | 3.33E-24 |
| ISL1 | 1.058866 | 2.299365 | 1.118715 | 1.12E-15 | 1.33E-14 |
| HLF | 0.569068 | 1.589438 | 1.481844 | 9.71E-10 | 5.60E-09 |
| HECW2 | 0.929713 | 1.463638 | 0.654701 | 1.09E-11 | 8.31E-11 |
| TGFBR3 | 3.505986 | 5.724111 | 0.707232 | 2.79E-08 | 1.27E-07 |
| ANGPTL2 | 13.51488 | 31.87634 | 1.237937 | 1.31E-19 | 2.36E-18 |
| FAM83B | 7.200554 | 4.629973 | -0.6371 | 1.02E-06 | 3.58E-06 |
| IGHV3-71 | 0.7757 | 2.562474 | 1.723966 | 1.96E-10 | 1.26E-09 |
| TMIGD3 | 0.908367 | 1.377122 | 0.60031 | 3.27E-07 | 1.25E-06 |
| TIMP4 | 1.051033 | 1.886183 | 0.843661 | 3.31E-08 | 1.49E-07 |
| MIR4786 | 0.409882 | 0.729597 | 0.831894 | 0.000338 | 0.000761 |
| GSPT2 | 1.40921 | 2.643379 | 0.907497 | 1.06E-21 | 2.40E-20 |
| SEPT5 | 1.177505 | 1.918812 | 0.70448 | 6.38E-24 | 1.83E-22 |
| AKAP12 | 4.37317 | 11.20964 | 1.357988 | 1.53E-23 | 4.23E-22 |
| DCLK1 | 0.447689 | 0.889886 | 0.991124 | 1.77E-29 | 1.18E-27 |
| AC010531.6 | 0.8688 | 0.570742 | -0.60619 | 5.89E-07 | 2.15E-06 |
| AP005131.5 | 0.329878 | 0.68181 | 1.04744 | 5.05E-09 | 2.62E-08 |
| ECEL1 | 0.718357 | 1.842588 | 1.35896 | 7.39E-12 | 5.76E-11 |
| MPZL2 | 15.02837 | 9.621272 | -0.64339 | 6.13E-08 | 2.64E-07 |
| THBS4 | 2.776367 | 25.06663 | 3.174498 | 2.25E-25 | 7.81E-24 |
| KANK2 | 7.153639 | 19.7449 | 1.464731 | 7.07E-30 | 5.14E-28 |
| LIMCH1 | 1.341033 | 2.170062 | 0.694391 | 1.05E-14 | 1.14E-13 |
| CPM | 4.368976 | 9.015172 | 1.04506 | 2.20E-12 | 1.83E-11 |
| PARM1 | 6.62683 | 11.05363 | 0.738129 | 8.72E-14 | 8.59E-13 |
| SLC2A1 | 44.94423 | 23.90494 | -0.91083 | 9.50E-07 | 3.36E-06 |
| LTF | 13.23597 | 36.2525 | 1.453616 | 1.99E-10 | 1.27E-09 |
| CRNN | 43.8962 | 0.201971 | -7.7638 | 0.001475 | 0.002934 |
| KCNE4 | 1.292343 | 3.581445 | 1.470553 | 8.58E-24 | 2.43E-22 |
| MSC | 3.768945 | 7.18478 | 0.930783 | 1.77E-19 | 3.15E-18 |
| FAM171B | 1.104194 | 1.900772 | 0.783591 | 3.34E-21 | 7.03E-20 |
| IL1R1 | 7.17128 | 17.50673 | 1.287607 | 4.02E-36 | 1.42E-33 |
| LTBP3 | 8.874406 | 17.91519 | 1.013461 | 3.09E-28 | 1.59E-26 |
| NPM1P6 | 0.769347 | 0.49357 | -0.64038 | 6.54E-08 | 2.80E-07 |
| CDH6 | 0.641951 | 1.251212 | 0.962792 | 6.54E-21 | 1.33E-19 |
| KCTD10 | 5.096416 | 7.861605 | 0.625341 | 1.67E-24 | 5.14E-23 |
| ZBTB4 | 12.27928 | 20.33965 | 0.728069 | 6.85E-21 | 1.40E-19 |
| SRGN | 74.20625 | 121.322 | 0.709229 | 6.58E-09 | 3.35E-08 |
| AC022364.1 | 0.686087 | 1.073909 | 0.646407 | 1.18E-13 | 1.14E-12 |
| SCAMP5 | 4.569719 | 7.077343 | 0.631102 | 6.91E-07 | 2.50E-06 |
| LDLRAD4 | 0.983051 | 2.275438 | 1.210807 | 3.70E-26 | 1.45E-24 |
| PTGER1 | 0.964833 | 3.466133 | 1.844975 | 2.07E-26 | 8.30E-25 |
| FABP5P7 | 4.262191 | 2.026253 | -1.07278 | 0.000221 | 0.000515 |
| PPIAP39 | 0.324847 | 0.692119 | 1.091258 | 4.97E-16 | 6.16E-15 |
| PDLIM7 | 17.32357 | 32.25797 | 0.896919 | 5.06E-13 | 4.56E-12 |
| TBC1D9 | 3.922001 | 7.10986 | 0.858231 | 4.90E-27 | 2.13E-25 |
| ADA2 | 9.319283 | 15.38939 | 0.723645 | 6.31E-13 | 5.61E-12 |
| RNA5SP283 | 3.125026 | 4.80047 | 0.619307 | 3.21E-05 | 8.71E-05 |
| IGHV2-70D | 5.124487 | 11.23971 | 1.133126 | 3.90E-09 | 2.06E-08 |
| TMEM35A | 0.291356 | 1.319496 | 2.179134 | 1.71E-20 | 3.38E-19 |
| BHLHA15 | 2.533038 | 5.224472 | 1.044417 | 2.64E-05 | 7.25E-05 |
| PEAK3 | 0.371591 | 0.66 | 0.828752 | 2.70E-14 | 2.80E-13 |
| RNU5F-1 | 2.200201 | 1.266687 | -0.79658 | 5.83E-07 | 2.13E-06 |
| SLC4A11 | 7.211819 | 4.170786 | -0.79004 | 5.77E-07 | 2.11E-06 |
| FABP5 | 27.16368 | 14.14685 | -0.9412 | 4.98E-05 | 0.000131 |
| IGLV1-36 | 7.09404 | 20.06584 | 1.500062 | 6.81E-07 | 2.46E-06 |
| KCTD15 | 2.46094 | 4.105024 | 0.738181 | 6.85E-15 | 7.57E-14 |
| MYL9 | 70.05227 | 322.5528 | 2.203032 | 9.46E-24 | 2.66E-22 |
| AL078595.1 | 1.279767 | 0.623824 | -1.03667 | 1.04E-06 | 3.66E-06 |
| NCS1 | 4.502283 | 10.31211 | 1.195611 | 9.89E-09 | 4.89E-08 |
| AKR1C2 | 2.329493 | 3.834347 | 0.718965 | 0.00027 | 0.000619 |
| GRHL1 | 2.32876 | 1.221906 | -0.93043 | 2.45E-06 | 8.10E-06 |
| MRVI1 | 3.402105 | 13.68695 | 2.008301 | 3.94E-35 | 1.04E-32 |
| DACT2 | 1.08253 | 1.63162 | 0.591898 | 1.10E-06 | 3.84E-06 |
| GIMAP7 | 6.651218 | 11.6779 | 0.812091 | 2.22E-16 | 2.86E-15 |
| CHN1 | 1.932432 | 3.73116 | 0.949207 | 1.05E-21 | 2.38E-20 |
| CASC19 | 4.132929 | 2.445035 | -0.75731 | 0.020109 | 0.031436 |
| CD79A | 20.54569 | 35.05539 | 0.770801 | 2.48E-09 | 1.35E-08 |
| AC040162.2 | 1.967213 | 1.306779 | -0.59014 | 5.42E-05 | 0.000141 |
| XCR1 | 0.360928 | 0.722179 | 1.000646 | 1.62E-07 | 6.48E-07 |
| HSPE1P6 | 1.006913 | 0.595666 | -0.75736 | 1.33E-06 | 4.59E-06 |
| IGHV3-13 | 13.54924 | 24.89609 | 0.877707 | 5.84E-06 | 1.80E-05 |
| TBX2 | 2.871569 | 4.524909 | 0.656049 | 3.21E-23 | 8.51E-22 |
| MEP1B | 0.348977 | 0.910143 | 1.382963 | 0.011898 | 0.019542 |
| IGKV3D-20 | 22.50586 | 34.03075 | 0.596538 | 1.39E-08 | 6.70E-08 |
| UBD | 40.76696 | 23.29391 | -0.80745 | 0.027508 | 0.041687 |
| EID1 | 32.03759 | 48.99275 | 0.612803 | 4.54E-19 | 7.68E-18 |
| DOCK11 | 3.066977 | 5.26019 | 0.778297 | 2.47E-14 | 2.58E-13 |
| METTL7A | 11.84891 | 19.17969 | 0.694825 | 3.48E-15 | 3.94E-14 |
| AC009299.2 | 1.067039 | 2.713667 | 1.346631 | 9.52E-21 | 1.92E-19 |
| ADCY4 | 0.99366 | 1.800693 | 0.857728 | 1.41E-21 | 3.11E-20 |
| ZNF662 | 0.348069 | 0.769928 | 1.145349 | 6.97E-21 | 1.42E-19 |
| RTN1 | 0.553012 | 1.224725 | 1.147075 | 3.04E-21 | 6.44E-20 |
| SMOC2 | 9.340869 | 30.90173 | 1.726059 | 8.01E-22 | 1.84E-20 |
| NKX2-3 | 0.442785 | 1.07452 | 1.279015 | 5.17E-17 | 7.06E-16 |
| IGLL3P | 0.586544 | 1.056442 | 0.848903 | 1.48E-06 | 5.08E-06 |
| LIN7A | 0.445271 | 0.771639 | 0.793241 | 1.96E-24 | 5.95E-23 |
| SNX29 | 2.050866 | 3.364871 | 0.714318 | 2.26E-23 | 6.10E-22 |
| RBP4 | 12.24137 | 27.59643 | 1.172716 | 1.08E-05 | 3.16E-05 |
| ZNF43 | 1.009681 | 1.745844 | 0.790025 | 7.10E-14 | 7.05E-13 |
| PPP1R13L | 14.53539 | 9.495447 | -0.61426 | 2.75E-07 | 1.06E-06 |
| FAP | 1.570666 | 3.112915 | 0.986889 | 4.23E-12 | 3.40E-11 |
| IGHV1OR15-2 | 3.008528 | 5.564962 | 0.887314 | 1.46E-09 | 8.27E-09 |
| SPON1 | 6.621657 | 26.90584 | 2.022655 | 4.98E-33 | 7.16E-31 |
| WFDC2 | 31.2547 | 47.30588 | 0.597946 | 2.96E-05 | 8.07E-05 |
| ADAMTSL3 | 0.296688 | 1.344247 | 2.179781 | 1.80E-33 | 2.93E-31 |
| SEMA6B | 3.13026 | 6.041084 | 0.948525 | 3.76E-18 | 5.81E-17 |
| ADAMTSL4 | 2.738392 | 4.296122 | 0.649706 | 1.20E-14 | 1.30E-13 |
| ISLR | 19.47141 | 50.65444 | 1.379331 | 4.57E-21 | 9.51E-20 |
| XKR9 | 1.844374 | 1.132762 | -0.70329 | 2.00E-05 | 5.63E-05 |
| GJA1 | 13.72069 | 24.10894 | 0.813215 | 5.30E-17 | 7.23E-16 |
| AC018475.1 | 3.832508 | 2.172888 | -0.81868 | 8.43E-13 | 7.38E-12 |
| AC092070.2 | 1.741579 | 2.788812 | 0.679254 | 2.02E-09 | 1.12E-08 |
| STX19 | 3.377073 | 2.101505 | -0.68435 | 7.82E-06 | 2.36E-05 |
| PPP1R12C | 7.603408 | 11.67851 | 0.619138 | 2.89E-09 | 1.56E-08 |
| ITPR1 | 1.375829 | 3.2033 | 1.219258 | 9.45E-26 | 3.53E-24 |
| ZNF154 | 0.705143 | 1.299207 | 0.881643 | 1.40E-16 | 1.84E-15 |
| HDAC5 | 5.378634 | 8.182708 | 0.605339 | 2.17E-16 | 2.80E-15 |
| F2RL2 | 4.381831 | 8.937417 | 1.028324 | 1.97E-14 | 2.08E-13 |
| EVL | 2.904743 | 4.916206 | 0.759135 | 5.47E-18 | 8.25E-17 |
| CASTOR3 | 1.691421 | 2.74372 | 0.697897 | 2.84E-12 | 2.33E-11 |
| S100A11P2 | 1.319755 | 0.786815 | -0.74617 | 9.27E-06 | 2.75E-05 |
| F10 | 1.815965 | 4.688677 | 1.368445 | 4.57E-17 | 6.28E-16 |
| CXorf36 | 1.42141 | 3.215534 | 1.177736 | 4.98E-31 | 4.72E-29 |
| SMARCA1 | 4.626972 | 7.618109 | 0.719365 | 2.43E-15 | 2.79E-14 |
| ZNF853 | 1.242557 | 3.50606 | 1.496539 | 1.60E-31 | 1.66E-29 |
| NAP1L5 | 1.229346 | 2.071948 | 0.753096 | 9.88E-16 | 1.18E-14 |
| EBF3 | 0.396141 | 1.023004 | 1.368728 | 3.97E-27 | 1.74E-25 |
| KRT18P11 | 1.180978 | 0.639275 | -0.88547 | 1.64E-12 | 1.39E-11 |
| LINC02595 | 2.172446 | 1.397173 | -0.63681 | 4.87E-07 | 1.80E-06 |
| MIR30C2 | 0.43076 | 0.698728 | 0.697849 | 6.35E-07 | 2.31E-06 |
| C2orf74 | 0.539731 | 0.865445 | 0.681201 | 1.98E-11 | 1.46E-10 |
| USP2 | 0.663818 | 1.229281 | 0.888955 | 6.54E-12 | 5.15E-11 |
| DUSP3 | 17.05005 | 25.79067 | 0.597073 | 1.87E-15 | 2.19E-14 |
| FAM198A | 0.313517 | 1.215721 | 1.955199 | 1.48E-34 | 3.09E-32 |
| C1QTNF2 | 0.343706 | 1.151352 | 1.744083 | 1.05E-35 | 3.48E-33 |
| C19orf12 | 5.00218 | 8.092234 | 0.693981 | 7.78E-09 | 3.91E-08 |
| IGHJ3P | 8.630109 | 17.62406 | 1.030096 | 7.39E-08 | 3.14E-07 |
| GPNMB | 19.85074 | 34.75035 | 0.807835 | 1.14E-10 | 7.59E-10 |
| PROC | 1.461318 | 2.391166 | 0.710444 | 0.000354 | 0.000793 |
| CD48 | 4.46983 | 7.185994 | 0.684968 | 1.29E-07 | 5.26E-07 |
| FAM69B | 1.990774 | 3.672105 | 0.883278 | 1.62E-14 | 1.72E-13 |
| IGHD2-15 | 0.587727 | 1.426476 | 1.279237 | 0.000415 | 0.00092 |
| REM1 | 0.834087 | 1.951616 | 1.2264 | 9.85E-26 | 3.65E-24 |
| AL596244.1 | 0.720564 | 1.37282 | 0.929944 | 1.35E-11 | 1.02E-10 |
| PDZK1 | 1.038277 | 1.769779 | 0.769378 | 0.000246 | 0.000567 |
| RIPOR3 | 0.568776 | 0.999864 | 0.813873 | 2.17E-15 | 2.51E-14 |
| LINC01436 | 1.080138 | 1.94229 | 0.846543 | 3.51E-11 | 2.51E-10 |
| ITGA9 | 1.713207 | 5.487728 | 1.67951 | 3.94E-33 | 5.87E-31 |
| IGLV2-34 | 1.038295 | 2.951219 | 1.507094 | 5.16E-07 | 1.90E-06 |
| CLCA4 | 8.776226 | 0.68548 | -3.67841 | 4.23E-05 | 0.000112 |
| CAVIN3 | 19.83403 | 30.59339 | 0.625243 | 1.04E-11 | 7.95E-11 |
| IGLL5 | 26.79326 | 50.66365 | 0.919081 | 4.63E-07 | 1.72E-06 |
| C1S | 22.92578 | 56.31317 | 1.296501 | 3.70E-32 | 4.21E-30 |
| TFPI2 | 1.040808 | 3.480271 | 1.741496 | 3.48E-17 | 4.85E-16 |
| SLAMF1 | 0.731763 | 1.105691 | 0.595501 | 4.41E-09 | 2.30E-08 |
| LINC00242 | 0.386261 | 0.632755 | 0.712072 | 6.14E-09 | 3.14E-08 |
| ALPK3 | 1.586405 | 2.687404 | 0.760452 | 3.27E-16 | 4.13E-15 |
| GAS5 | 40.10639 | 26.60104 | -0.59235 | 5.26E-12 | 4.19E-11 |
| SH3BGRL | 40.71669 | 70.05904 | 0.782951 | 1.25E-16 | 1.65E-15 |
| CES3 | 4.651066 | 2.893098 | -0.68495 | 0.003715 | 0.0068 |
| RNF217 | 0.600702 | 1.249297 | 1.056395 | 2.80E-23 | 7.50E-22 |
| PTMAP4 | 27.81948 | 11.91658 | -1.22313 | 3.32E-16 | 4.19E-15 |
| IGHV7-56 | 0.459829 | 1.542831 | 1.74641 | 4.68E-08 | 2.05E-07 |
| IGLJ2 | 0.622263 | 1.338791 | 1.105335 | 2.71E-07 | 1.05E-06 |
| TUSC3 | 1.921781 | 3.773841 | 0.97359 | 1.21E-27 | 5.78E-26 |
| AL121761.1 | 9.836595 | 4.475181 | -1.13621 | 0.00184 | 0.003582 |
| IGKV2D-24 | 1.835602 | 3.551019 | 0.95198 | 1.06E-05 | 3.12E-05 |
| DPT | 1.829194 | 8.421253 | 2.202827 | 3.71E-23 | 9.72E-22 |
| PLEKHO1 | 4.431268 | 8.403281 | 0.923233 | 8.11E-16 | 9.81E-15 |
| CHAC2 | 4.728285 | 2.982049 | -0.66501 | 5.48E-12 | 4.36E-11 |
| IGHG3 | 94.39175 | 180.0251 | 0.931465 | 3.50E-07 | 1.33E-06 |
| ALDH1A3 | 2.539379 | 5.260658 | 1.050767 | 5.68E-23 | 1.46E-21 |
| SLC16A2 | 4.494608 | 7.507358 | 0.74011 | 8.65E-19 | 1.42E-17 |
| PTCH1 | 2.054861 | 3.423545 | 0.73645 | 1.01E-16 | 1.34E-15 |
| TMEM252 | 0.316729 | 1.340265 | 2.081197 | 1.48E-20 | 2.95E-19 |
| IGHG2 | 180.2593 | 341.1975 | 0.920533 | 3.16E-09 | 1.69E-08 |
| SEMA6A | 2.699101 | 4.508343 | 0.740118 | 1.17E-12 | 1.00E-11 |
| EPM2A | 0.66923 | 1.288057 | 0.944622 | 2.61E-16 | 3.33E-15 |
| FBXL22 | 0.625078 | 2.212275 | 1.823424 | 1.03E-19 | 1.89E-18 |
| AL158825.2 | 1.250469 | 0.817004 | -0.61405 | 0.000116 | 0.000283 |
| CCNA2 | 19.55055 | 12.98628 | -0.59022 | 6.03E-12 | 4.76E-11 |
| C3orf18 | 0.654167 | 1.401018 | 1.098745 | 1.67E-26 | 6.73E-25 |
| PCOLCE | 11.75391 | 23.45966 | 0.997041 | 3.23E-18 | 5.02E-17 |
| DEGS2 | 12.55747 | 8.261557 | -0.60406 | 4.40E-05 | 0.000116 |
| PCLAF | 11.17136 | 6.823193 | -0.71129 | 2.38E-15 | 2.73E-14 |
| C14orf132 | 0.997686 | 3.743466 | 1.907717 | 1.06E-33 | 1.81E-31 |
| GPX2 | 353.0152 | 234.6402 | -0.58928 | 8.47E-05 | 0.000213 |
| GPR68 | 2.356297 | 4.091502 | 0.796109 | 7.40E-15 | 8.12E-14 |
| NNAT | 1.462931 | 5.910258 | 2.014359 | 8.48E-07 | 3.02E-06 |
| WBP1L | 10.55921 | 16.17921 | 0.61564 | 3.45E-26 | 1.36E-24 |
| RBP2 | 2.897336 | 13.80683 | 2.252583 | 0.000371 | 0.000829 |
| IGHD2-2 | 0.971751 | 1.961352 | 1.013189 | 0.000315 | 0.000711 |
| RPS4XP11 | 2.369178 | 1.572746 | -0.5911 | 8.07E-07 | 2.89E-06 |
| AC103563.1 | 1.054756 | 2.472222 | 1.228899 | 1.28E-09 | 7.28E-09 |
| JAM3 | 2.67868 | 8.025389 | 1.583049 | 3.94E-39 | 4.17E-36 |
| SLC46A1 | 0.839398 | 1.45118 | 0.7898 | 2.56E-13 | 2.40E-12 |
| GKN2 | 22.96564 | 71.29225 | 1.634268 | 0.022366 | 0.034597 |
| HSPA12A | 0.774126 | 1.353089 | 0.805617 | 2.23E-19 | 3.91E-18 |
| RNU6-618P | 1.075712 | 0.713693 | -0.59192 | 0.003165 | 0.005869 |
| IGKV1-8 | 5.380568 | 11.85414 | 1.139561 | 1.95E-09 | 1.08E-08 |
| PRX | 0.5725 | 0.892331 | 0.640303 | 1.46E-12 | 1.24E-11 |
| BDKRB1 | 0.63617 | 0.957955 | 0.590545 | 3.79E-07 | 1.43E-06 |
| NLRP1 | 1.396336 | 2.317855 | 0.731145 | 6.29E-16 | 7.69E-15 |
| CDH5 | 5.612179 | 11.5225 | 1.037821 | 7.32E-29 | 4.28E-27 |
| TMEM273 | 0.988967 | 1.882 | 0.928272 | 3.16E-20 | 6.15E-19 |
| CPA3 | 4.686171 | 14.3906 | 1.618646 | 7.13E-25 | 2.30E-23 |
| AP005018.2 | 0.729456 | 3.123206 | 2.098136 | 1.82E-06 | 6.13E-06 |
| NT5C3A | 16.50489 | 10.98503 | -0.58735 | 3.37E-18 | 5.22E-17 |
| SPOCK1 | 3.550541 | 8.873992 | 1.321544 | 1.13E-13 | 1.10E-12 |
| CSPG4P10 | 0.939235 | 0.621809 | -0.59501 | 0.009994 | 0.0167 |
| LRP12 | 0.736146 | 1.259178 | 0.774418 | 1.16E-21 | 2.59E-20 |
| PTGDR2 | 0.499856 | 0.930693 | 0.896794 | 4.33E-08 | 1.91E-07 |
| PTN | 3.146427 | 6.762167 | 1.103771 | 2.02E-18 | 3.20E-17 |
| BICC1 | 1.54947 | 4.500565 | 1.538332 | 1.76E-31 | 1.78E-29 |
| ABCC9 | 0.755225 | 2.996732 | 1.988412 | 1.57E-26 | 6.34E-25 |
| SLIT2 | 0.551671 | 2.473142 | 2.164464 | 8.25E-30 | 5.88E-28 |
| IGHV3-35 | 0.944222 | 2.459357 | 1.381083 | 1.49E-11 | 1.11E-10 |
| IGHV3-60 | 0.406327 | 1.065682 | 1.391064 | 2.94E-10 | 1.83E-09 |
| APOC3 | 1.170188 | 10.27169 | 3.133862 | 6.60E-06 | 2.01E-05 |
| RBPMS2 | 3.057925 | 14.14506 | 2.209673 | 4.26E-25 | 1.42E-23 |
| TTC7B | 0.521234 | 0.889783 | 0.771522 | 6.60E-20 | 1.25E-18 |
| IGHV3-52 | 0.62556 | 1.878663 | 1.586486 | 9.27E-09 | 4.60E-08 |
| FAM126A | 1.23966 | 1.863926 | 0.588401 | 5.24E-13 | 4.71E-12 |
| RHOQ | 6.418652 | 9.996998 | 0.639225 | 4.13E-21 | 8.65E-20 |
| AC087286.2 | 0.478243 | 1.004005 | 1.069952 | 1.02E-21 | 2.31E-20 |
| MRGPRF | 3.252933 | 15.86813 | 2.286319 | 7.05E-28 | 3.43E-26 |
| PRR34-AS1 | 3.292116 | 2.185457 | -0.59108 | 2.26E-06 | 7.51E-06 |
| HAND2-AS1 | 0.199803 | 1.682002 | 3.073532 | 7.80E-18 | 1.15E-16 |
| MYH11 | 25.16447 | 215.0778 | 3.095399 | 1.74E-21 | 3.80E-20 |
| HCFC2 | 1.81568 | 2.793391 | 0.621508 | 6.37E-18 | 9.53E-17 |
| AC112484.3 | 1.430644 | 0.539226 | -1.4077 | 7.28E-06 | 2.20E-05 |
| TNFRSF17 | 1.469681 | 2.666459 | 0.859422 | 5.31E-06 | 1.65E-05 |
| VLDLR | 0.860854 | 1.336579 | 0.634705 | 1.35E-11 | 1.02E-10 |
| PDGFRB | 17.12355 | 38.24076 | 1.159129 | 2.59E-25 | 8.93E-24 |
| SATB1 | 1.945421 | 2.95107 | 0.601156 | 7.14E-12 | 5.59E-11 |
| CALCA | 0.394804 | 7.453466 | 4.238704 | 2.21E-05 | 6.15E-05 |
| TCEAL3 | 3.888091 | 6.726431 | 0.790779 | 1.32E-16 | 1.74E-15 |
| TRABD2B | 0.419239 | 1.220517 | 1.541647 | 9.29E-27 | 3.89E-25 |
| NXPH3 | 0.416889 | 1.176732 | 1.497051 | 1.85E-17 | 2.64E-16 |
| AC099789.1 | 3.139399 | 1.99419 | -0.65469 | 1.84E-05 | 5.19E-05 |
| ROR2 | 1.407935 | 4.855565 | 1.786059 | 3.09E-29 | 1.96E-27 |
| ZFHX4 | 0.333399 | 1.094776 | 1.715313 | 9.01E-27 | 3.78E-25 |
| CCNJL | 0.557318 | 0.917445 | 0.719121 | 1.44E-10 | 9.44E-10 |
| SPIB | 2.018244 | 5.559929 | 1.461966 | 1.31E-08 | 6.36E-08 |
| ZNF385B | 0.382649 | 0.627987 | 0.714715 | 5.20E-15 | 5.81E-14 |
| TRIM40 | 0.970358 | 0.577861 | -0.74779 | 0.000723 | 0.001533 |
| RNF43 | 12.58118 | 6.908687 | -0.86478 | 0.015065 | 0.024206 |
| HMGN2P15 | 0.620907 | 1.040577 | 0.744935 | 3.64E-07 | 1.38E-06 |
| IFFO1 | 1.083168 | 2.019441 | 0.8987 | 1.26E-26 | 5.14E-25 |
| PRDM8 | 0.962463 | 1.49183 | 0.632281 | 3.18E-14 | 3.28E-13 |
| FMO1 | 0.610252 | 1.461171 | 1.259649 | 4.59E-11 | 3.23E-10 |
| EPB41L3 | 1.234098 | 2.40647 | 0.963461 | 2.25E-18 | 3.55E-17 |
| GSN | 28.00666 | 49.47756 | 0.821004 | 1.77E-19 | 3.15E-18 |
| SEMA6C | 1.127473 | 2.257139 | 1.001402 | 8.63E-25 | 2.75E-23 |
| LY9 | 0.366285 | 0.742648 | 1.019711 | 2.32E-10 | 1.46E-09 |
| ANKRD44 | 1.054833 | 1.813086 | 0.781433 | 5.13E-14 | 5.15E-13 |
| HAS2 | 1.291634 | 1.980059 | 0.616346 | 4.96E-08 | 2.16E-07 |
| ERICH4 | 0.366864 | 0.766082 | 1.062254 | 0.000309 | 0.0007 |
| HHIP | 0.289505 | 0.99945 | 1.787548 | 6.06E-19 | 1.01E-17 |
| ARHGEF10 | 1.992351 | 3.078308 | 0.627665 | 3.98E-14 | 4.03E-13 |
| AL133215.2 | 0.982783 | 0.639475 | -0.61998 | 3.75E-10 | 2.30E-09 |
| C5AR1 | 6.611356 | 10.73434 | 0.699216 | 2.03E-11 | 1.50E-10 |
| HSD17B6 | 0.603781 | 1.268134 | 1.070609 | 1.31E-17 | 1.90E-16 |
| CLEC1A | 0.586197 | 1.103092 | 0.912096 | 3.03E-23 | 8.08E-22 |
| CERS4 | 3.026309 | 4.825577 | 0.673142 | 5.90E-14 | 5.89E-13 |
| WFDC1 | 0.930221 | 2.2684 | 1.286029 | 4.87E-20 | 9.30E-19 |
| PRR16 | 1.85361 | 3.137679 | 0.75936 | 1.38E-14 | 1.48E-13 |
| HAGLR | 1.205811 | 1.886274 | 0.645535 | 1.31E-16 | 1.72E-15 |
| RPL12P47 | 3.142057 | 1.916928 | -0.71291 | 4.64E-05 | 0.000122 |
| AC007637.1 | 0.653755 | 1.228614 | 0.91021 | 1.72E-17 | 2.46E-16 |
| FBXO27 | 1.193712 | 1.803236 | 0.595134 | 4.70E-15 | 5.27E-14 |
| AL391335.1 | 0.587184 | 1.052884 | 0.842461 | 0.000152 | 0.000364 |
| OXTR | 0.549292 | 0.912146 | 0.731691 | 1.82E-09 | 1.01E-08 |
| AC002091.2 | 0.487359 | 1.301298 | 1.416894 | 4.14E-20 | 7.93E-19 |
| POLR3GP1 | 0.848852 | 0.537562 | -0.65908 | 0.017052 | 0.027078 |
| IGHV1-14 | 0.838691 | 2.146305 | 1.355644 | 1.25E-09 | 7.15E-09 |
| IGHV3-64 | 4.238774 | 11.27426 | 1.411314 | 3.99E-09 | 2.10E-08 |
| VPS35L | 2.36865 | 3.886082 | 0.714252 | 1.85E-17 | 2.64E-16 |
| LRRN1 | 1.026157 | 3.328589 | 1.697659 | 1.46E-19 | 2.61E-18 |
| HSPB6 | 19.98693 | 158.2733 | 2.985289 | 4.05E-24 | 1.19E-22 |
| C8orf88 | 0.569355 | 3.004931 | 2.399933 | 7.13E-25 | 2.30E-23 |
| ADGRF5 | 4.063371 | 7.260486 | 0.837389 | 6.19E-21 | 1.27E-19 |
| SYNGR1 | 1.262118 | 2.236062 | 0.825113 | 8.80E-22 | 2.01E-20 |
| LMCD1 | 1.961783 | 3.206034 | 0.708624 | 4.32E-17 | 5.95E-16 |
| IGHV3OR16-13 | 2.312039 | 7.22976 | 1.644782 | 3.71E-11 | 2.64E-10 |
| ADARB1 | 1.578784 | 2.861483 | 0.85795 | 6.78E-20 | 1.27E-18 |
| NPTX1 | 0.576407 | 1.539144 | 1.416969 | 4.97E-17 | 6.79E-16 |
| PGM5 | 1.372003 | 8.836022 | 2.687114 | 7.02E-23 | 1.80E-21 |
| MMP17 | 0.603862 | 0.941844 | 0.641268 | 2.75E-13 | 2.57E-12 |
| FBLN1 | 9.478053 | 48.51235 | 2.35569 | 1.82E-42 | 3.08E-38 |
| SMKR1 | 1.711338 | 1.03416 | -0.72667 | 0.003449 | 0.006354 |
| GUCY1A1 | 3.336208 | 11.08174 | 1.731903 | 2.07E-28 | 1.10E-26 |
| GJB3 | 14.03974 | 8.553941 | -0.71486 | 2.79E-07 | 1.08E-06 |
| ZEB1 | 3.380382 | 9.493936 | 1.48982 | 4.06E-30 | 3.06E-28 |
| C1R | 21.99044 | 51.35972 | 1.223761 | 1.53E-28 | 8.25E-27 |
| FZD1 | 4.26005 | 7.041936 | 0.725102 | 7.40E-19 | 1.23E-17 |
| ASF1B | 17.79058 | 11.74112 | -0.59954 | 7.40E-15 | 8.12E-14 |
| SMO | 2.117503 | 5.048405 | 1.253463 | 3.85E-29 | 2.42E-27 |
| ADAMTS8 | 0.518026 | 2.675787 | 2.368867 | 1.48E-25 | 5.35E-24 |
| CCR7 | 2.134934 | 5.071566 | 1.24824 | 6.63E-13 | 5.86E-12 |
| ORM1 | 5.823252 | 18.43686 | 1.662696 | 0.000144 | 0.000346 |
| IGHV1OR21-1 | 0.370502 | 1.242886 | 1.746142 | 8.18E-10 | 4.76E-09 |
| IGHV1OR16-1 | 0.560309 | 0.91648 | 0.709881 | 9.89E-08 | 4.12E-07 |
| TRAV8-3 | 0.822183 | 1.413448 | 0.781688 | 3.41E-06 | 1.10E-05 |
| IL1R2 | 8.6581 | 4.983933 | -0.79677 | 0.006241 | 0.010891 |
| C6orf58 | 3.717006 | 23.70453 | 2.67295 | 0.001031 | 0.002117 |
| GJA5 | 1.811926 | 3.540342 | 0.966364 | 1.65E-22 | 4.10E-21 |
| KRT8P36 | 4.988758 | 2.983708 | -0.74157 | 6.33E-05 | 0.000163 |
| GPR176 | 1.463495 | 2.655928 | 0.859798 | 1.71E-22 | 4.25E-21 |
| C6orf52 | 0.962931 | 0.618801 | -0.63796 | 9.89E-06 | 2.93E-05 |
| E2F2 | 3.490582 | 2.283315 | -0.61234 | 2.40E-08 | 1.10E-07 |
| HOXA13 | 6.34015 | 3.649872 | -0.79667 | 0.002386 | 0.004553 |
| ADGRB2 | 0.641155 | 1.191496 | 0.89403 | 1.43E-14 | 1.53E-13 |
| ASB4 | 0.91384 | 1.820334 | 0.994189 | 0.000926 | 0.001923 |
| TMEM150C | 1.639486 | 2.612101 | 0.671967 | 1.77E-18 | 2.83E-17 |
| RBMS1 | 3.853347 | 6.234425 | 0.694144 | 4.98E-18 | 7.54E-17 |
| GALNT17 | 0.33307 | 0.843041 | 1.339777 | 1.39E-29 | 9.44E-28 |
| UPK3BL1 | 0.914889 | 0.603221 | -0.60091 | 0.027945 | 0.042289 |
| BHLHE22 | 0.264289 | 0.813751 | 1.622472 | 1.26E-25 | 4.59E-24 |
| IGKV2-26 | 0.613163 | 1.548408 | 1.336444 | 1.29E-07 | 5.25E-07 |
| SEMA3G | 1.184504 | 3.061313 | 1.369868 | 5.71E-31 | 5.32E-29 |
| PPP1R3C | 2.082995 | 3.921266 | 0.912661 | 1.12E-19 | 2.03E-18 |
| GNLY | 4.941063 | 2.516087 | -0.97364 | 0.001855 | 0.003609 |
| NCALD | 1.206799 | 1.979371 | 0.713857 | 6.35E-13 | 5.64E-12 |
| RPL10P13 | 1.518077 | 0.834951 | -0.86248 | 3.26E-06 | 1.05E-05 |
| AC092718.4 | 12.29898 | 8.183684 | -0.58772 | 3.47E-11 | 2.48E-10 |
| VASN | 7.033875 | 12.05731 | 0.777517 | 6.50E-23 | 1.66E-21 |
| SLC16A7 | 0.634389 | 1.475261 | 1.21753 | 1.06E-22 | 2.68E-21 |
| NCAPG | 6.0676 | 3.858061 | -0.65325 | 2.75E-13 | 2.57E-12 |
| RPL34P27 | 4.006719 | 2.513665 | -0.67263 | 0.000242 | 0.00056 |
| SPRR2A | 86.05339 | 1.532947 | -5.81085 | 0.000672 | 0.001431 |
| TCL1A | 1.164021 | 2.420096 | 1.055947 | 5.38E-08 | 2.33E-07 |
| SMIM31 | 3.615807 | 2.318715 | -0.64099 | 0.015328 | 0.024584 |
| TEK | 1.3742 | 3.532307 | 1.362018 | 2.64E-37 | 1.66E-34 |
| GLRB | 0.336226 | 0.92485 | 1.459789 | 1.23E-28 | 6.75E-27 |
| FGD5 | 2.225023 | 4.523248 | 1.023539 | 8.72E-33 | 1.18E-30 |
| IGHJ1 | 1.371783 | 4.433165 | 1.692285 | 6.45E-10 | 3.81E-09 |
| FILIP1L | 10.09646 | 25.15108 | 1.316772 | 4.32E-27 | 1.89E-25 |
| LRRC8C | 1.549009 | 2.448308 | 0.660439 | 3.70E-18 | 5.72E-17 |
| LDHA | 165.8123 | 109.2209 | -0.6023 | 2.66E-14 | 2.77E-13 |
| TRGV7 | 1.076803 | 1.752095 | 0.702327 | 1.56E-09 | 8.79E-09 |
| GRID1 | 0.351724 | 0.774929 | 1.139619 | 2.54E-30 | 2.01E-28 |
| PGM5-AS1 | 0.313589 | 1.900726 | 2.599605 | 1.04E-13 | 1.02E-12 |
| CSRP1 | 20.2414 | 56.84329 | 1.489681 | 2.93E-17 | 4.10E-16 |
| PSD | 0.794992 | 3.573051 | 2.168145 | 2.27E-15 | 2.62E-14 |
| DCLK2 | 0.577917 | 1.652073 | 1.515342 | 1.79E-26 | 7.22E-25 |
| ARHGEF25 | 1.439229 | 4.682746 | 1.702059 | 6.93E-27 | 2.95E-25 |
| CDH13 | 1.360543 | 2.165314 | 0.670393 | 7.02E-13 | 6.19E-12 |
| RNF223 | 2.951314 | 1.748537 | -0.75521 | 1.24E-06 | 4.29E-06 |
| CACNA1C | 0.75574 | 2.43645 | 1.688818 | 6.14E-24 | 1.76E-22 |
| SYT11 | 2.802948 | 6.679103 | 1.252709 | 1.81E-32 | 2.24E-30 |
| RPS6KA2 | 3.869491 | 6.527555 | 0.754399 | 1.25E-23 | 3.46E-22 |
| EPHA7 | 0.439517 | 1.936062 | 2.139135 | 2.15E-21 | 4.63E-20 |
| VIP | 0.798224 | 4.33298 | 2.440494 | 1.30E-16 | 1.70E-15 |
| SNORA33 | 8.869303 | 5.845194 | -0.60157 | 5.40E-09 | 2.79E-08 |
| DGKG | 0.328716 | 0.699551 | 1.089588 | 1.96E-10 | 1.25E-09 |
| PLAT | 7.726985 | 13.64633 | 0.820536 | 1.53E-25 | 5.49E-24 |
| CLDN11 | 0.587883 | 1.05744 | 0.846975 | 3.71E-24 | 1.09E-22 |
| VANGL2 | 2.949864 | 5.018328 | 0.766558 | 2.18E-12 | 1.82E-11 |
| TPM2 | 29.27422 | 108.3099 | 1.887462 | 1.33E-14 | 1.43E-13 |
| ARC | 0.59383 | 1.027747 | 0.791364 | 6.70E-06 | 2.04E-05 |
| CAV1 | 16.29196 | 29.92431 | 0.877158 | 7.29E-22 | 1.68E-20 |
| CTIF | 3.188926 | 5.03144 | 0.657901 | 4.65E-16 | 5.78E-15 |
| CHRDL1 | 0.873677 | 6.02976 | 2.786929 | 1.07E-29 | 7.47E-28 |
| GPR162 | 0.284843 | 0.712381 | 1.322482 | 5.15E-37 | 3.12E-34 |
| MATN2 | 4.734612 | 8.696246 | 0.877147 | 3.96E-18 | 6.09E-17 |
| C20orf194 | 1.646638 | 3.523762 | 1.097593 | 9.10E-24 | 2.57E-22 |
| TYMSOS | 2.825594 | 1.686599 | -0.74444 | 2.35E-11 | 1.72E-10 |
| IGKV1-6 | 26.23751 | 69.2643 | 1.400481 | 2.93E-08 | 1.33E-07 |
| IGFBP5 | 48.78523 | 138.8738 | 1.509258 | 7.01E-29 | 4.16E-27 |
| BACE1 | 3.606777 | 6.304716 | 0.805721 | 2.27E-27 | 1.01E-25 |
| COL4A2 | 49.27941 | 97.44018 | 0.983532 | 6.48E-21 | 1.33E-19 |
| SEMA5B | 0.376287 | 0.772361 | 1.03744 | 3.03E-19 | 5.22E-18 |
| UTS2R | 0.427992 | 1.190449 | 1.475851 | 3.47E-07 | 1.32E-06 |
| KIRREL1 | 3.890834 | 9.861623 | 1.341746 | 6.81E-40 | 1.05E-36 |
| MAGEH1 | 3.722967 | 8.514598 | 1.193486 | 7.37E-35 | 1.81E-32 |
| SPC25 | 6.481346 | 4.287085 | -0.5963 | 1.42E-15 | 1.67E-14 |
| AL355353.1 | 2.957778 | 1.90935 | -0.63143 | 2.75E-07 | 1.06E-06 |
| ENAH | 6.960079 | 10.91792 | 0.649522 | 1.20E-11 | 9.12E-11 |
| PER1 | 5.605881 | 12.00413 | 1.098517 | 9.78E-20 | 1.79E-18 |
| LDB2 | 1.25142 | 3.049271 | 1.284898 | 9.16E-41 | 3.11E-37 |
| GCG | 0.097866 | 3.808562 | 5.282299 | 0.012032 | 0.019744 |
| TRBV18 | 0.610568 | 1.047352 | 0.778522 | 5.77E-07 | 2.11E-06 |
| GXYLT2 | 1.291539 | 4.0447 | 1.646942 | 9.71E-28 | 4.65E-26 |
| TYRP1 | 0.668037 | 1.669249 | 1.3212 | 9.96E-15 | 1.08E-13 |
| DTNA | 0.340025 | 1.153438 | 1.762229 | 8.02E-26 | 3.02E-24 |
| AC012618.3 | 0.654533 | 1.0113 | 0.627673 | 4.27E-07 | 1.59E-06 |
| LINC02365 | 0.409452 | 0.760508 | 0.893267 | 0.005417 | 0.00957 |
| IFNG | 1.042701 | 0.473196 | -1.13982 | 0.004701 | 0.008416 |
| RHOJ | 2.494214 | 6.161788 | 1.304764 | 1.40E-30 | 1.20E-28 |
| MARK1 | 1.035471 | 1.8367 | 0.826829 | 5.57E-15 | 6.22E-14 |
| TGFB1I1 | 6.545669 | 17.52086 | 1.420461 | 5.01E-25 | 1.65E-23 |
| AC008443.4 | 1.277897 | 0.762509 | -0.74495 | 0.000184 | 0.000435 |
| TXNIP | 102.5959 | 176.1103 | 0.779507 | 5.51E-13 | 4.93E-12 |
| AC083809.1 | 4.462431 | 0.519736 | -3.10198 | 0.032149 | 0.047996 |
| RGS2 | 14.8565 | 27.06019 | 0.865078 | 1.20E-13 | 1.16E-12 |
| DSP | 58.40575 | 38.53496 | -0.59994 | 1.12E-05 | 3.27E-05 |
| RSPO4 | 0.531873 | 1.332918 | 1.325434 | 0.000184 | 0.000435 |
| ARHGEF26 | 1.083215 | 3.228211 | 1.575415 | 1.76E-14 | 1.86E-13 |
| PTGIS | 2.629604 | 13.90638 | 2.402829 | 1.37E-33 | 2.33E-31 |
| GREM2 | 0.702907 | 3.888104 | 2.467661 | 1.36E-28 | 7.39E-27 |
| PNMA8A | 0.341437 | 1.76096 | 2.36667 | 7.40E-31 | 6.60E-29 |
| IGLV3-9 | 13.50048 | 54.27549 | 2.00729 | 6.68E-07 | 2.42E-06 |
| P3H3 | 2.941956 | 5.09474 | 0.792233 | 2.13E-15 | 2.48E-14 |
| ELFN1-AS1 | 7.06333 | 3.203428 | -1.14073 | 9.37E-06 | 2.78E-05 |
| NID2 | 4.042328 | 7.689703 | 0.927741 | 1.17E-17 | 1.70E-16 |
| NOX1 | 6.372815 | 2.745194 | -1.21502 | 0.003976 | 0.007235 |
| LINC00921 | 0.423863 | 0.668874 | 0.658139 | 2.92E-12 | 2.39E-11 |
| CRLF1 | 0.541027 | 2.453388 | 2.181003 | 1.06E-13 | 1.03E-12 |
| ABCG8 | 0.555207 | 0.87359 | 0.65393 | 3.01E-06 | 9.76E-06 |
| ICAM3 | 0.771733 | 1.273437 | 0.722554 | 3.05E-10 | 1.89E-09 |
| TTC36 | 0.420577 | 0.651818 | 0.632098 | 6.20E-05 | 0.00016 |
| C3orf80 | 1.139881 | 2.12681 | 0.899808 | 1.19E-15 | 1.42E-14 |
| GJB5 | 4.846709 | 2.099677 | -1.20684 | 0.024962 | 0.038207 |
| HAND2 | 1.143876 | 7.73182 | 2.756878 | 8.33E-17 | 1.11E-15 |
| GABBR1 | 1.493121 | 2.330786 | 0.642486 | 9.73E-09 | 4.81E-08 |
| DACT1 | 2.531426 | 5.795845 | 1.195069 | 4.39E-25 | 1.45E-23 |
| KCNJ5 | 0.450587 | 0.79541 | 0.819894 | 2.38E-11 | 1.74E-10 |
| Z99129.4 | 2.681032 | 4.379227 | 0.707888 | 2.22E-15 | 2.56E-14 |
| FAM129C | 0.360212 | 0.68698 | 0.93142 | 4.01E-09 | 2.11E-08 |
| MIR4537 | 1.172253 | 2.19435 | 0.904509 | 1.72E-05 | 4.89E-05 |
| LYG1 | 0.97129 | 0.647415 | -0.58521 | 5.23E-06 | 1.62E-05 |
| GBA3 | 0.627599 | 1.133238 | 0.852537 | 0.001528 | 0.003026 |
| AMBP | 0.710895 | 2.484047 | 1.804983 | 0.000208 | 0.000486 |
| RNU6-1098P | 0.392241 | 0.606811 | 0.629507 | 5.53E-05 | 0.000144 |
| LSAMP | 0.838923 | 2.580828 | 1.621224 | 2.93E-36 | 1.13E-33 |
| SLC51B | 1.25171 | 2.638168 | 1.075636 | 3.40E-14 | 3.50E-13 |
| HSPA4L | 2.22235 | 1.348487 | -0.72074 | 0.000168 | 0.0004 |
| HSPB7 | 2.614911 | 16.33103 | 2.642782 | 2.75E-21 | 5.84E-20 |
| DPYSL3 | 11.87466 | 41.39148 | 1.801448 | 6.15E-29 | 3.70E-27 |
| MPV17L | 1.638691 | 2.464169 | 0.588557 | 3.47E-08 | 1.55E-07 |
| NNMT | 14.09333 | 23.89912 | 0.761945 | 1.55E-13 | 1.49E-12 |
| IGLV4-69 | 36.30215 | 77.08601 | 1.086414 | 4.93E-06 | 1.54E-05 |
| FAM198B | 5.737116 | 12.37872 | 1.109464 | 3.33E-28 | 1.71E-26 |
| SPON2 | 9.896639 | 16.94315 | 0.775692 | 1.29E-12 | 1.10E-11 |
| ST6GALNAC6 | 3.539397 | 6.036353 | 0.770174 | 1.73E-21 | 3.78E-20 |
| PTGER4 | 5.281873 | 7.960732 | 0.591852 | 7.56E-07 | 2.72E-06 |
| AC037198.2 | 0.335655 | 1.047785 | 1.642293 | 2.78E-20 | 5.43E-19 |
| RGS5 | 13.05715 | 31.76047 | 1.282393 | 1.62E-30 | 1.34E-28 |
| AC087741.2 | 0.845682 | 0.541308 | -0.64367 | 0.000264 | 0.000604 |
| SHE | 0.541922 | 1.45238 | 1.42226 | 1.00E-36 | 5.16E-34 |
| NR3C1 | 3.495243 | 6.761963 | 0.952049 | 1.82E-27 | 8.35E-26 |
| CHRDL2 | 5.553576 | 23.09343 | 2.055993 | 3.82E-23 | 1.00E-21 |
| TRAV23DV6 | 0.410436 | 0.768807 | 0.905462 | 3.55E-09 | 1.88E-08 |
| IGHV6-1 | 2.672876 | 10.63321 | 1.992113 | 2.64E-12 | 2.18E-11 |
| PRKAR2B | 1.670262 | 5.18723 | 1.63489 | 3.32E-30 | 2.56E-28 |
| VMO1 | 3.545644 | 6.76188 | 0.931377 | 0.000391 | 0.000869 |
| FAM81A | 2.131862 | 1.357916 | -0.65072 | 6.39E-09 | 3.26E-08 |
| MIR5190 | 0.509951 | 1.236272 | 1.277564 | 1.50E-08 | 7.19E-08 |
| RARB | 1.315519 | 2.157146 | 0.713492 | 9.88E-18 | 1.44E-16 |
| MMRN2 | 3.431124 | 8.459288 | 1.301855 | 2.16E-37 | 1.41E-34 |
| RAI14 | 6.907733 | 11.45816 | 0.730091 | 4.38E-22 | 1.04E-20 |
| FGF14-AS2 | 0.318075 | 0.830039 | 1.383813 | 6.90E-20 | 1.29E-18 |
| AL390719.2 | 7.91996 | 5.263114 | -0.58958 | 2.24E-08 | 1.04E-07 |
| AC138305.3 | 11.85266 | 7.186965 | -0.72176 | 2.04E-08 | 9.53E-08 |
| IGHV3-20 | 8.46843 | 18.15567 | 1.100254 | 7.83E-07 | 2.81E-06 |
| NATD1 | 2.222359 | 3.918948 | 0.818375 | 8.99E-25 | 2.86E-23 |
| RAD51AP1 | 7.293148 | 4.843787 | -0.59041 | 3.44E-13 | 3.17E-12 |
| SNCG | 2.994978 | 5.366957 | 0.841559 | 2.22E-18 | 3.49E-17 |
| SERPING1 | 61.32118 | 109.6092 | 0.837912 | 5.90E-18 | 8.86E-17 |
| MFAP5 | 2.714485 | 9.111414 | 1.746997 | 7.78E-26 | 2.95E-24 |
| RMDN2 | 0.520292 | 0.894086 | 0.781093 | 1.93E-18 | 3.07E-17 |
| ARHGEF15 | 1.393453 | 3.1048 | 1.155837 | 1.42E-30 | 1.21E-28 |
| ARHGAP24 | 1.297815 | 2.344198 | 0.85301 | 1.02E-24 | 3.24E-23 |
| AC069218.1 | 2.079335 | 1.263842 | -0.71831 | 1.17E-10 | 7.76E-10 |
| APOB | 0.672416 | 5.525628 | 3.038712 | 9.38E-14 | 9.21E-13 |
| RPL12P17 | 0.606143 | 0.398596 | -0.60473 | 6.64E-07 | 2.40E-06 |
| IGHJ2 | 4.370555 | 10.39031 | 1.249351 | 4.92E-06 | 1.53E-05 |
| TGFBR1 | 8.256434 | 12.65959 | 0.61664 | 2.36E-15 | 2.72E-14 |
| GIMAP1 | 1.017956 | 1.869132 | 0.876693 | 1.20E-18 | 1.94E-17 |
| CHST2 | 2.660857 | 4.097106 | 0.622715 | 1.98E-18 | 3.15E-17 |
| FBP2 | 0.844921 | 1.61821 | 0.937511 | 0.000699 | 0.001486 |
| TEX45 | 0.67338 | 0.413787 | -0.70253 | 0.011547 | 0.019011 |
| IGHD3-3 | 2.361583 | 3.56701 | 0.594961 | 0.000228 | 0.000528 |
| ANKRD36BP2 | 0.423953 | 0.649271 | 0.614916 | 6.35E-06 | 1.94E-05 |
| PRSS51 | 0.856929 | 1.353887 | 0.659859 | 0.000117 | 0.000286 |
| DYNC2H1 | 0.401121 | 0.768727 | 0.938432 | 1.86E-21 | 4.03E-20 |
| ASPN | 14.95671 | 39.15576 | 1.388432 | 8.42E-18 | 1.24E-16 |
| AC010332.1 | 0.616081 | 1.342656 | 1.123897 | 1.46E-15 | 1.72E-14 |
| ADAMTS4 | 2.888214 | 4.773138 | 0.72476 | 7.44E-08 | 3.16E-07 |
| NDN | 3.866476 | 12.20703 | 1.658621 | 3.02E-34 | 5.62E-32 |
| NALT1 | 0.408427 | 0.936257 | 1.196828 | 0.000979 | 0.002022 |
| ARHGAP32 | 10.0104 | 6.333847 | -0.66035 | 1.77E-12 | 1.49E-11 |
| HBB | 14.37798 | 34.16121 | 1.248498 | 0.002768 | 0.005197 |
| GSTA1 | 18.32911 | 32.85719 | 0.842073 | 1.64E-08 | 7.77E-08 |
| TRBV7-3 | 0.461765 | 0.699712 | 0.599604 | 3.92E-06 | 1.25E-05 |
| IGHV3-47 | 0.640813 | 2.104379 | 1.715419 | 6.09E-12 | 4.80E-11 |
| PPAT | 4.805012 | 3.156089 | -0.6064 | 5.74E-15 | 6.40E-14 |
| SALL1 | 0.368167 | 0.64515 | 0.809273 | 0.000227 | 0.000527 |
| NOTCH3 | 18.68194 | 32.92063 | 0.817347 | 8.33E-17 | 1.11E-15 |
| IGKJ5 | 8.335933 | 14.23986 | 0.772519 | 5.38E-09 | 2.78E-08 |
| CYP1B1 | 2.606254 | 9.567217 | 1.876122 | 9.14E-22 | 2.08E-20 |
| MAMSTR | 0.963807 | 1.506556 | 0.644438 | 1.49E-09 | 8.40E-09 |
| TAP1 | 64.57148 | 38.33427 | -0.75226 | 2.65E-07 | 1.03E-06 |
| MFAP4 | 23.63155 | 109.6177 | 2.213695 | 2.19E-39 | 3.04E-36 |
| AL583785.1 | 0.360162 | 1.011801 | 1.490209 | 3.33E-14 | 3.43E-13 |
| IGLV1-51 | 104.1359 | 239.4218 | 1.201087 | 4.83E-08 | 2.11E-07 |
| KRT6A | 118.1116 | 1.812404 | -6.0261 | 0.012726 | 0.020785 |
| FLNA | 88.851 | 307.6494 | 1.791827 | 1.99E-19 | 3.52E-18 |
| FGFRL1 | 20.72858 | 13.65353 | -0.60235 | 0.031555 | 0.047195 |
| AC069307.1 | 0.847099 | 0.521453 | -0.69999 | 2.18E-11 | 1.60E-10 |
| LDLRAD2 | 0.716027 | 1.331756 | 0.895244 | 9.43E-19 | 1.55E-17 |
| SCTR | 0.54619 | 1.423621 | 1.382091 | 3.89E-14 | 3.96E-13 |
| FAM111B | 5.754371 | 3.700757 | -0.63684 | 2.98E-11 | 2.15E-10 |
| ABCB1 | 1.170227 | 2.101423 | 0.844578 | 2.26E-22 | 5.56E-21 |
| ZNF671 | 0.946302 | 1.497383 | 0.662071 | 2.51E-14 | 2.61E-13 |
| FSTL4 | 0.265886 | 0.793892 | 1.578133 | 3.80E-05 | 0.000102 |
| AC005786.1 | 1.067885 | 0.675332 | -0.66109 | 0.000145 | 0.000348 |
| HOXA10 | 5.325166 | 3.147097 | -0.75881 | 2.29E-06 | 7.60E-06 |
| ZDBF2 | 0.473887 | 1.042406 | 1.137303 | 2.95E-19 | 5.10E-18 |
| NLGN3 | 0.458302 | 1.010058 | 1.140066 | 5.47E-20 | 1.04E-18 |
| IGHJ3 | 12.18424 | 31.847 | 1.386141 | 1.33E-09 | 7.58E-09 |
| PAMR1 | 0.838461 | 2.142245 | 1.353308 | 3.91E-28 | 1.99E-26 |
| IGKV1D-33 | 2.53183 | 7.376144 | 1.542686 | 7.32E-10 | 4.29E-09 |
| HOXA11-AS | 1.644088 | 0.815411 | -1.01169 | 0.004627 | 0.008305 |
| LRP8 | 2.721145 | 1.706091 | -0.67352 | 1.64E-08 | 7.77E-08 |
| HAS1 | 0.242692 | 0.858229 | 1.822238 | 9.63E-15 | 1.05E-13 |
| LRRC32 | 8.134371 | 20.29643 | 1.319124 | 6.20E-28 | 3.06E-26 |
| IGHVIII-67-2 | 0.327138 | 0.946361 | 1.53249 | 3.72E-07 | 1.41E-06 |
| SLC7A11 | 3.980401 | 2.533459 | -0.65181 | 1.35E-08 | 6.53E-08 |
| MYC | 59.27588 | 36.66851 | -0.6929 | 6.35E-11 | 4.39E-10 |
| IGHV3-15 | 86.18248 | 158.2208 | 0.876473 | 1.64E-08 | 7.77E-08 |
| LY6D | 26.15992 | 4.224124 | -2.63063 | 0.00166 | 0.003264 |
| NUPR2 | 1.050108 | 1.785469 | 0.765766 | 0.002252 | 0.004318 |
| KRTAP3-1 | 5.242148 | 0.258809 | -4.3402 | 0.009512 | 0.015957 |
| ADCY9 | 3.432423 | 5.548392 | 0.692842 | 4.06E-18 | 6.24E-17 |
| SOX17 | 1.158365 | 2.463024 | 1.08834 | 1.79E-21 | 3.90E-20 |
| AC007342.9 | 1.697595 | 0.927248 | -0.87247 | 6.79E-08 | 2.90E-07 |
| MICAL2 | 7.622085 | 12.22562 | 0.68165 | 7.70E-14 | 7.61E-13 |
| RGL1 | 2.679697 | 5.81314 | 1.117247 | 3.85E-27 | 1.69E-25 |
| POU6F1 | 0.78198 | 1.594239 | 1.027664 | 9.74E-26 | 3.62E-24 |
| GYPC | 4.091371 | 10.13832 | 1.309163 | 5.54E-26 | 2.14E-24 |
| TGFB3 | 1.976848 | 4.692281 | 1.247087 | 1.38E-17 | 1.99E-16 |
| RCAN1 | 4.819429 | 7.311792 | 0.601363 | 1.97E-13 | 1.87E-12 |
| HSPD1 | 108.596 | 69.80998 | -0.63747 | 2.41E-16 | 3.09E-15 |
| MEIS1 | 1.381662 | 2.904627 | 1.071948 | 9.04E-17 | 1.21E-15 |
| CRCT1 | 13.64339 | 0.21161 | -6.01065 | 0.010618 | 0.017629 |
| JHY | 0.50583 | 0.82375 | 0.703552 | 1.58E-17 | 2.27E-16 |
| PRTN3 | 0.072284 | 1.861216 | 4.686433 | 0.016651 | 0.02649 |
| CAVIN1 | 29.23385 | 76.45098 | 1.386895 | 4.74E-29 | 2.92E-27 |
| CASP5 | 1.908776 | 0.788508 | -1.27545 | 4.61E-06 | 1.45E-05 |
| FCMR | 4.210563 | 8.87457 | 1.075664 | 7.05E-12 | 5.52E-11 |
| TEAD2 | 6.011304 | 9.956837 | 0.72801 | 7.23E-15 | 7.95E-14 |
| TRBV4-2 | 0.609831 | 0.937984 | 0.621153 | 4.94E-08 | 2.15E-07 |
| PLAGL1 | 2.059293 | 4.019598 | 0.964902 | 2.91E-15 | 3.31E-14 |
| AC119396.1 | 0.602497 | 2.161995 | 1.843338 | 4.93E-17 | 6.74E-16 |
| DMBX1 | 0.8319 | 0.504226 | -0.72234 | 0.005317 | 0.009419 |
| PLXDC2 | 3.506413 | 9.485257 | 1.435691 | 2.31E-28 | 1.22E-26 |
| SNORC | 4.139627 | 1.539435 | -1.4271 | 1.90E-17 | 2.70E-16 |
| CRY2 | 3.85801 | 5.866122 | 0.60455 | 4.98E-18 | 7.54E-17 |
| PDGFC | 2.373792 | 4.101738 | 0.789042 | 3.19E-21 | 6.73E-20 |
| DMKN | 4.573676 | 2.431848 | -0.9113 | 0.023028 | 0.035516 |
| KIF3C | 1.500151 | 2.396562 | 0.675858 | 7.08E-19 | 1.18E-17 |
| C16orf54 | 1.375167 | 2.408797 | 0.808706 | 3.05E-10 | 1.89E-09 |
| IGKV2-28 | 2.1813 | 7.057073 | 1.693882 | 2.37E-08 | 1.10E-07 |
| F13A1 | 2.646312 | 7.881569 | 1.5745 | 1.17E-20 | 2.35E-19 |
| LINC01857 | 1.554129 | 2.489883 | 0.679972 | 8.45E-06 | 2.53E-05 |
| AL008733.1 | 0.421362 | 0.645936 | 0.616331 | 0.000132 | 0.00032 |
| FUZ | 2.087911 | 3.289317 | 0.655727 | 3.92E-09 | 2.07E-08 |
| DIO2 | 3.952853 | 8.776527 | 1.150756 | 3.31E-19 | 5.68E-18 |
| HSPA1B | 42.84095 | 24.22512 | -0.82249 | 3.48E-10 | 2.14E-09 |
| MGP | 35.01384 | 144.626 | 2.04633 | 1.13E-26 | 4.68E-25 |
| AC093278.2 | 0.833694 | 1.523507 | 0.869807 | 3.66E-22 | 8.71E-21 |
| CYP7B1 | 0.876621 | 2.141668 | 1.288709 | 1.80E-27 | 8.31E-26 |
| A4GALT | 4.409734 | 6.747985 | 0.613765 | 2.71E-15 | 3.10E-14 |
| TPSB2 | 6.146808 | 12.35691 | 1.007409 | 1.15E-13 | 1.12E-12 |
| CNIH2 | 0.776841 | 1.39194 | 0.841405 | 0.00011 | 0.000271 |
| AL157871.3 | 1.198998 | 0.762815 | -0.65242 | 0.003951 | 0.007192 |
| NUAK1 | 2.020759 | 3.638341 | 0.848383 | 1.25E-25 | 4.55E-24 |
| KCNH2 | 2.844468 | 5.281478 | 0.892783 | 1.69E-11 | 1.26E-10 |
| THPO | 0.513916 | 0.913461 | 0.829812 | 7.58E-13 | 6.68E-12 |
| TMOD1 | 0.856857 | 3.048839 | 1.831134 | 1.38E-18 | 2.22E-17 |
| MXRA7 | 5.209947 | 12.13347 | 1.219651 | 2.72E-24 | 8.15E-23 |
| HSPA12B | 1.626346 | 3.714468 | 1.191521 | 8.52E-33 | 1.16E-30 |
| LRMDA | 0.62178 | 0.948309 | 0.608952 | 1.35E-10 | 8.90E-10 |
| TXNP4 | 2.001449 | 1.180132 | -0.7621 | 4.84E-07 | 1.79E-06 |
| SAMD11 | 0.85675 | 2.566504 | 1.582859 | 7.74E-31 | 6.87E-29 |
| FEV | 0.467084 | 4.282974 | 3.19686 | 7.13E-08 | 3.04E-07 |
| ST3GAL4 | 3.489263 | 5.731127 | 0.715897 | 1.43E-14 | 1.53E-13 |
| LGI4 | 0.440173 | 1.184189 | 1.427758 | 5.50E-24 | 1.59E-22 |
| LAMC3 | 0.699904 | 1.208626 | 0.788138 | 0.003796 | 0.006938 |
| RN7SL473P | 0.832624 | 1.544419 | 0.891327 | 8.88E-07 | 3.16E-06 |
| IGHV3OR16-10 | 0.319785 | 0.960783 | 1.587109 | 6.59E-12 | 5.18E-11 |
| AC092903.1 | 0.476151 | 0.806328 | 0.75995 | 0.000781 | 0.001644 |
| TEAD1 | 5.827215 | 9.279834 | 0.671292 | 1.60E-13 | 1.53E-12 |
| LY86 | 3.849685 | 6.408978 | 0.735354 | 9.11E-11 | 6.14E-10 |
| COX7A1 | 3.919622 | 8.19818 | 1.064589 | 1.70E-17 | 2.44E-16 |
| CENPM | 8.721704 | 5.812709 | -0.5854 | 1.20E-10 | 7.94E-10 |
| C19orf57 | 1.30944 | 0.763638 | -0.77799 | 5.17E-05 | 0.000135 |
| SYNM | 5.592473 | 42.26939 | 2.918055 | 2.19E-17 | 3.09E-16 |
| TIE1 | 2.430516 | 5.26802 | 1.115998 | 9.11E-28 | 4.38E-26 |
| LAMA2 | 1.102599 | 4.316233 | 1.968864 | 1.62E-37 | 1.15E-34 |
| RORA | 0.740643 | 1.313483 | 0.826547 | 9.27E-20 | 1.71E-18 |
| FIBIN | 2.268293 | 5.92709 | 1.385717 | 7.08E-27 | 3.00E-25 |
| FOXF2 | 5.423754 | 12.70662 | 1.228217 | 4.22E-25 | 1.40E-23 |
| GHR | 0.290118 | 1.151982 | 1.989409 | 1.41E-34 | 2.98E-32 |
| ELOVL2 | 0.386563 | 0.781871 | 1.016228 | 3.53E-14 | 3.62E-13 |
| GLIS2 | 4.357887 | 9.354759 | 1.102072 | 7.94E-27 | 3.34E-25 |
| UBE2SP1 | 2.034501 | 1.308953 | -0.63626 | 2.07E-10 | 1.32E-09 |
| AC103563.3 | 0.664474 | 2.386197 | 1.844427 | 2.19E-10 | 1.39E-09 |
| QRSL1 | 14.16863 | 4.684271 | -1.5968 | 0.00066 | 0.001407 |
| AC084880.1 | 0.847958 | 1.424657 | 0.748549 | 3.48E-05 | 9.39E-05 |
| FBLN2 | 7.313704 | 19.93219 | 1.446426 | 4.46E-23 | 1.16E-21 |
| METTL24 | 0.351311 | 1.869075 | 2.411502 | 2.35E-29 | 1.52E-27 |
| AC124916.1 | 0.716511 | 0.459101 | -0.64218 | 0.000155 | 0.000371 |
| PECAM1 | 14.1455 | 31.62678 | 1.160803 | 1.84E-33 | 2.97E-31 |
| IGHV3-38 | 0.428801 | 1.330528 | 1.633619 | 2.79E-11 | 2.02E-10 |
| AL355309.1 | 17.37823 | 10.32274 | -0.75146 | 7.03E-05 | 0.000179 |
| FEZF1-AS1 | 2.191805 | 1.434937 | -0.61113 | 0.014942 | 0.024029 |
| DNAJB5 | 1.387304 | 4.994851 | 1.848158 | 5.84E-24 | 1.68E-22 |
| CMAHP | 1.155454 | 2.119318 | 0.87514 | 1.60E-21 | 3.52E-20 |
| ACYP1 | 5.701455 | 3.741049 | -0.60789 | 1.35E-12 | 1.15E-11 |
| SCARA3 | 3.522604 | 9.247266 | 1.392384 | 6.44E-27 | 2.76E-25 |
| FZD4 | 2.520014 | 5.229746 | 1.053309 | 3.39E-33 | 5.09E-31 |
| EMILIN1 | 17.6591 | 48.46621 | 1.456567 | 2.23E-28 | 1.18E-26 |
| IGLV1-50 | 0.883838 | 2.742243 | 1.633502 | 1.42E-07 | 5.77E-07 |
| SIRPB2 | 0.431549 | 0.71883 | 0.736127 | 1.87E-10 | 1.21E-09 |
| AOC3 | 4.750839 | 20.65909 | 2.120522 | 6.22E-29 | 3.73E-27 |
| COL5A2 | 24.14575 | 41.79688 | 0.791626 | 1.11E-11 | 8.46E-11 |
| AL023284.4 | 4.969386 | 3.09174 | -0.68465 | 5.56E-09 | 2.87E-08 |
| AC009088.4 | 0.716847 | 0.398276 | -0.84789 | 5.57E-07 | 2.04E-06 |
| FOSB | 9.617269 | 18.06807 | 0.909743 | 7.02E-08 | 2.99E-07 |
| ADAMTS10 | 0.767735 | 1.934787 | 1.333495 | 1.55E-29 | 1.05E-27 |
| PJA2 | 15.89297 | 24.44508 | 0.621155 | 1.58E-16 | 2.07E-15 |
| MEF2A | 7.213303 | 11.84472 | 0.715512 | 1.12E-22 | 2.82E-21 |
| SSBP2 | 0.846982 | 1.786444 | 1.076687 | 3.84E-19 | 6.54E-18 |
| RAET1L | 1.738456 | 0.520762 | -1.73911 | 0.001206 | 0.00244 |
| RAI2 | 3.792763 | 11.03173 | 1.540338 | 2.30E-33 | 3.62E-31 |
| GNB4 | 3.330668 | 6.832816 | 1.036669 | 6.01E-25 | 1.96E-23 |
| IQANK1 | 16.30721 | 10.341 | -0.65713 | 3.12E-11 | 2.24E-10 |
| MS4A2 | 0.375085 | 1.035415 | 1.46492 | 3.27E-23 | 8.64E-22 |
| RUSC2 | 4.979437 | 7.813443 | 0.649976 | 5.20E-16 | 6.41E-15 |
| SPIN4 | 5.695585 | 3.41123 | -0.73955 | 3.59E-10 | 2.21E-09 |
| SLC8A2 | 0.281431 | 0.761783 | 1.436599 | 1.48E-09 | 8.36E-09 |
| KIT | 1.374881 | 4.089126 | 1.572485 | 5.97E-31 | 5.54E-29 |
| RAMP3 | 5.149642 | 11.27251 | 1.130265 | 2.15E-27 | 9.69E-26 |
| CHST15 | 5.423808 | 8.187584 | 0.594132 | 3.40E-18 | 5.26E-17 |
| FENDRR | 1.195135 | 4.423557 | 1.888033 | 6.04E-31 | 5.57E-29 |
| CHIA | 0.080401 | 3.850307 | 5.581613 | 0.004882 | 0.008714 |
| AL391422.4 | 1.215874 | 1.957242 | 0.686828 | 1.71E-14 | 1.81E-13 |
| FAM84B | 24.70884 | 15.23487 | -0.69765 | 0.001154 | 0.002343 |
| AC087500.1 | 0.41528 | 0.629008 | 0.598996 | 3.40E-08 | 1.53E-07 |
| TMEM130 | 0.2808 | 0.972345 | 1.791925 | 2.53E-26 | 1.00E-24 |
| IGLV3-1 | 51.46055 | 84.32167 | 0.712437 | 3.53E-06 | 1.13E-05 |
| RCSD1 | 2.036657 | 3.859626 | 0.922258 | 4.42E-18 | 6.77E-17 |
| KRT18P10 | 1.636343 | 0.853258 | -0.93942 | 3.78E-14 | 3.86E-13 |
| PGA3 | 6.311465 | 46.25466 | 2.873552 | 0.025153 | 0.038458 |
| APOA4 | 2.969161 | 17.10681 | 2.526443 | 2.02E-08 | 9.45E-08 |
| SLC7A5 | 50.22767 | 28.98329 | -0.79326 | 3.30E-05 | 8.95E-05 |
| GIF | 3.305798 | 19.32226 | 2.547194 | 0.001522 | 0.003017 |
| FTOP1 | 0.490078 | 0.91466 | 0.900225 | 2.54E-09 | 1.38E-08 |
| FAM228B | 0.659421 | 1.017668 | 0.625995 | 1.37E-13 | 1.32E-12 |
| APOA1 | 9.181313 | 75.93126 | 3.047922 | 2.89E-09 | 1.56E-08 |
| TSPAN33 | 5.169207 | 7.980013 | 0.626448 | 4.33E-11 | 3.06E-10 |
| TNRC6C | 2.188443 | 3.409463 | 0.63964 | 2.85E-13 | 2.66E-12 |
| PEG3 | 0.195766 | 0.886235 | 2.178556 | 2.15E-31 | 2.16E-29 |
| SLCO2A1 | 6.267169 | 16.23085 | 1.372853 | 2.32E-34 | 4.48E-32 |
| HACD1 | 0.874791 | 1.569613 | 0.843398 | 1.01E-05 | 2.99E-05 |
| CD99L2 | 4.510478 | 8.112171 | 0.846808 | 1.77E-21 | 3.87E-20 |
| ADAP2 | 3.083452 | 4.673414 | 0.59993 | 6.40E-13 | 5.67E-12 |
| LRRN2 | 0.567037 | 1.469786 | 1.37409 | 2.03E-20 | 4.00E-19 |
| RASSF8 | 1.086429 | 3.754021 | 1.788843 | 2.32E-34 | 4.48E-32 |
| ETV7 | 9.903113 | 5.790651 | -0.77416 | 3.89E-07 | 1.47E-06 |
| IGHV4-4 | 11.0913 | 20.32986 | 0.874173 | 2.29E-06 | 7.60E-06 |
| FAM20A | 1.049509 | 2.064489 | 0.976071 | 2.74E-16 | 3.49E-15 |
| SCG2 | 1.563143 | 3.148244 | 1.010097 | 2.41E-14 | 2.53E-13 |
| BTBD19 | 1.30746 | 2.136177 | 0.708265 | 5.60E-14 | 5.60E-13 |
| RIMKLB | 0.887062 | 2.254776 | 1.345878 | 3.40E-23 | 8.95E-22 |
| IGHV1-69 | 23.24201 | 54.82485 | 1.238096 | 2.07E-10 | 1.32E-09 |
| PIPOX | 0.794941 | 1.904554 | 1.260533 | 3.33E-10 | 2.05E-09 |
| FKBP7 | 2.850824 | 4.817381 | 0.75687 | 2.12E-20 | 4.17E-19 |
| EXPH5 | 2.654936 | 1.52813 | -0.79691 | 6.07E-12 | 4.79E-11 |
| HMMR | 7.969955 | 5.295896 | -0.5897 | 1.71E-10 | 1.11E-09 |
| LRIG1 | 11.54509 | 17.61454 | 0.609487 | 1.15E-10 | 7.63E-10 |
| AC009948.1 | 1.249402 | 2.048873 | 0.713593 | 2.26E-17 | 3.19E-16 |
| F7 | 0.390693 | 0.765764 | 0.970863 | 0.000174 | 0.000413 |
| CFP | 0.375407 | 0.836817 | 1.156457 | 4.21E-17 | 5.82E-16 |
| IGKV1OR22-1 | 0.270658 | 1.013989 | 1.905501 | 2.81E-08 | 1.28E-07 |
| KRT4 | 140.0668 | 0.681764 | -7.68263 | 0.002343 | 0.004479 |
| AC009812.4 | 0.542559 | 0.824296 | 0.603383 | 3.55E-10 | 2.18E-09 |
| GPRASP1 | 0.499777 | 1.769238 | 1.82377 | 3.27E-29 | 2.07E-27 |
| IGKV1D-8 | 3.032521 | 8.477821 | 1.483176 | 1.47E-10 | 9.61E-10 |
| AC136428.1 | 0.892641 | 4.540109 | 2.346575 | 3.50E-09 | 1.86E-08 |
| ARMCX2 | 2.32656 | 5.181515 | 1.155176 | 3.88E-30 | 2.96E-28 |
| NBEA | 0.547389 | 1.384506 | 1.338733 | 2.07E-23 | 5.64E-22 |
| ADCY5 | 0.991877 | 4.822203 | 2.281459 | 1.23E-32 | 1.65E-30 |
| IGHV1-58 | 6.635014 | 20.21016 | 1.60691 | 1.10E-07 | 4.53E-07 |
| AC034105.1 | 1.167545 | 3.341591 | 1.517056 | 9.36E-10 | 5.42E-09 |
| CLEC10A | 1.055758 | 2.505432 | 1.24678 | 7.28E-15 | 8.01E-14 |
| C16orf45 | 2.661936 | 5.706434 | 1.100114 | 4.09E-25 | 1.36E-23 |
| AC011446.1 | 0.853658 | 0.492455 | -0.79367 | 7.07E-07 | 2.55E-06 |
| IGHV3-74 | 42.18995 | 95.73779 | 1.182189 | 4.52E-08 | 1.98E-07 |
| IGHE | 0.647545 | 1.149762 | 0.828283 | 3.15E-10 | 1.94E-09 |
| FZD2 | 3.874142 | 6.928057 | 0.838574 | 5.00E-11 | 3.50E-10 |
| PCOLCE2 | 0.414133 | 0.717829 | 0.793544 | 4.13E-06 | 1.31E-05 |
| EFS | 1.351733 | 2.708649 | 1.002763 | 1.86E-30 | 1.52E-28 |
| TRAV21 | 0.965603 | 1.569986 | 0.70125 | 4.96E-08 | 2.16E-07 |
| BMP8A | 1.887081 | 3.13914 | 0.734213 | 7.16E-10 | 4.20E-09 |
| GPER1 | 0.87773 | 1.654855 | 0.914855 | 3.30E-16 | 4.16E-15 |
| PTGIR | 0.841728 | 1.729177 | 1.03866 | 1.84E-25 | 6.46E-24 |
| NEURL1 | 1.21765 | 1.85972 | 0.610986 | 7.94E-08 | 3.36E-07 |
| PPP1R1A | 0.364595 | 1.513378 | 2.053406 | 1.34E-15 | 1.59E-14 |
| DSC2 | 9.648902 | 5.205243 | -0.8904 | 7.12E-05 | 0.000181 |
| IGHJ2P | 0.493802 | 1.225268 | 1.311093 | 3.96E-06 | 1.26E-05 |
| CHSY3 | 0.64228 | 1.296448 | 1.01329 | 1.96E-27 | 8.93E-26 |
| LRRK2 | 0.534947 | 1.053014 | 0.977057 | 9.08E-18 | 1.33E-16 |
| SST | 7.576006 | 52.91449 | 2.804153 | 2.28E-10 | 1.44E-09 |
| PTX3 | 0.294374 | 0.887922 | 1.592781 | 1.53E-18 | 2.46E-17 |
| HPN | 1.289758 | 4.469567 | 1.793034 | 4.72E-10 | 2.85E-09 |
| CHRNA3 | 0.385644 | 1.859127 | 2.269283 | 2.94E-08 | 1.33E-07 |
| CLIP3 | 3.775926 | 12.13403 | 1.684156 | 4.69E-30 | 3.50E-28 |
| SERPINF1 | 15.16234 | 44.08002 | 1.539632 | 1.05E-29 | 7.35E-28 |
| TSPAN4 | 3.134301 | 5.679577 | 0.85764 | 3.31E-25 | 1.12E-23 |
| AL031651.2 | 0.433553 | 0.776034 | 0.83991 | 5.36E-14 | 5.37E-13 |
| HOXA11 | 2.753312 | 1.44821 | -0.9269 | 0.000247 | 0.000569 |
| TRAV9-2 | 0.819828 | 1.3627 | 0.733074 | 2.75E-07 | 1.07E-06 |
| IL11RA | 0.755811 | 1.1843 | 0.647937 | 2.17E-13 | 2.04E-12 |
| COL6A2 | 71.58845 | 161.9374 | 1.177638 | 4.46E-23 | 1.16E-21 |
| ATOH8 | 0.501083 | 0.999268 | 0.995822 | 4.46E-18 | 6.82E-17 |
| AC108047.1 | 1.804507 | 1.10016 | -0.71389 | 8.01E-12 | 6.21E-11 |
| HRH2 | 0.510553 | 1.209177 | 1.243892 | 5.49E-21 | 1.13E-19 |
| ZNF528 | 0.62022 | 1.211078 | 0.965441 | 5.10E-21 | 1.05E-19 |
| CFD | 18.70561 | 35.73541 | 0.933883 | 2.75E-05 | 7.55E-05 |
| AC136628.3 | 0.893682 | 1.675625 | 0.906866 | 1.17E-15 | 1.39E-14 |
| TRBV10-3 | 0.765269 | 1.234116 | 0.68944 | 1.55E-06 | 5.28E-06 |
| SCD | 68.98008 | 43.78316 | -0.6558 | 2.44E-06 | 8.06E-06 |
| HAVCR1 | 0.981012 | 1.560679 | 0.669831 | 0.01909 | 0.030013 |
| BANK1 | 1.403424 | 2.514891 | 0.841544 | 2.29E-07 | 8.99E-07 |
| ETNK2 | 0.965618 | 1.823384 | 0.917094 | 5.90E-17 | 7.99E-16 |
| LINC02321 | 0.749919 | 0.467862 | -0.68065 | 0.001932 | 0.003744 |
| TPSD1 | 1.34897 | 2.154517 | 0.675506 | 1.13E-06 | 3.94E-06 |
| ACTA2-AS1 | 0.613914 | 2.260215 | 1.880353 | 1.12E-21 | 2.53E-20 |
| B3GNT6 | 2.874204 | 6.368909 | 1.147884 | 0.028545 | 0.043097 |
| CCL19 | 10.40608 | 26.05171 | 1.323952 | 1.15E-15 | 1.37E-14 |
| WTIP | 0.486485 | 1.019755 | 1.067756 | 6.65E-25 | 2.15E-23 |
| GLIPR1 | 3.821625 | 6.023799 | 0.656487 | 6.67E-19 | 1.11E-17 |
| RNU2-63P | 0.693792 | 0.436831 | -0.66743 | 0.003466 | 0.006383 |
| DYNC1I1 | 0.500836 | 1.045751 | 1.062128 | 4.01E-17 | 5.55E-16 |
| CKB | 26.05405 | 56.54318 | 1.117846 | 2.89E-10 | 1.80E-09 |
| PLEKHH2 | 1.095632 | 1.763076 | 0.686332 | 3.80E-13 | 3.49E-12 |
| AC092279.2 | 0.5614 | 0.868539 | 0.629562 | 3.24E-05 | 8.80E-05 |
| FABP4 | 1.145589 | 7.701763 | 2.749099 | 9.74E-14 | 9.55E-13 |
| AC124312.5 | 0.225277 | 0.779122 | 1.79015 | 8.86E-20 | 1.64E-18 |
| QPRT | 5.275928 | 9.300837 | 0.817936 | 2.28E-09 | 1.25E-08 |
| ITGA1 | 4.448798 | 9.478622 | 1.091262 | 7.93E-21 | 1.61E-19 |
| CD8B2 | 0.411562 | 0.638898 | 0.634475 | 7.42E-05 | 0.000188 |
| DEPP1 | 22.22789 | 36.3492 | 0.709553 | 5.56E-14 | 5.57E-13 |
| CEBPA | 11.63692 | 18.99021 | 0.706547 | 0.005463 | 0.009641 |
| LINC00659 | 3.239051 | 1.70836 | -0.92296 | 0.00205 | 0.003952 |
| FGG | 1.141103 | 3.574094 | 1.647149 | 0.001595 | 0.003149 |
| AC023090.1 | 2.543125 | 1.43678 | -0.82376 | 0.010105 | 0.016878 |
| P4HA3 | 0.595882 | 1.351885 | 1.181875 | 2.24E-17 | 3.17E-16 |
| ZBTB46-AS1 | 0.328216 | 0.911964 | 1.474333 | 3.16E-05 | 8.58E-05 |
| DNAAF3 | 0.690696 | 0.399956 | -0.78821 | 0.000223 | 0.000518 |
| CCDC8 | 0.865328 | 3.420508 | 1.982891 | 1.87E-29 | 1.24E-27 |
| MEIS3 | 1.063346 | 2.095976 | 0.979011 | 2.17E-23 | 5.89E-22 |
| IGHG4 | 156.1363 | 348.1732 | 1.156999 | 7.17E-08 | 3.05E-07 |
| IGLV3-16 | 1.698955 | 3.755193 | 1.144239 | 6.52E-07 | 2.37E-06 |
| PRRT3-AS1 | 4.564776 | 3.021096 | -0.59547 | 6.73E-06 | 2.05E-05 |
| OLFM1 | 0.982988 | 2.405989 | 1.291385 | 2.12E-21 | 4.57E-20 |
| A2M | 65.01739 | 178.798 | 1.459433 | 7.20E-37 | 3.82E-34 |
| DOCK10 | 1.256582 | 2.246542 | 0.838201 | 3.13E-13 | 2.91E-12 |
| KLF15 | 0.816695 | 2.23003 | 1.449193 | 1.07E-14 | 1.16E-13 |
| AP003071.4 | 0.317096 | 1.600467 | 2.335501 | 4.60E-26 | 1.80E-24 |
| CD34 | 4.577855 | 9.438061 | 1.043819 | 1.44E-30 | 1.22E-28 |
| RPSAP54 | 2.597324 | 1.616624 | -0.68404 | 2.27E-09 | 1.24E-08 |
| DDR2 | 2.513003 | 9.17081 | 1.867637 | 3.35E-33 | 5.07E-31 |
| CRISPLD1 | 0.82567 | 3.391899 | 2.038457 | 1.36E-37 | 1.05E-34 |
| PENK | 0.366677 | 0.744679 | 1.022107 | 1.34E-10 | 8.82E-10 |
| GAMT | 3.152854 | 6.239972 | 0.984881 | 2.15E-16 | 2.78E-15 |
| SNORD104 | 23.62831 | 15.53576 | -0.60492 | 4.11E-09 | 2.16E-08 |
| PLN | 3.849822 | 19.78483 | 2.361531 | 3.99E-20 | 7.67E-19 |
| FBXL7 | 1.518989 | 4.334398 | 1.512721 | 1.29E-36 | 5.59E-34 |
| IGLV2-14 | 196.5524 | 336.956 | 0.777646 | 1.30E-07 | 5.29E-07 |
| IGHV1-46 | 32.94329 | 69.83421 | 1.083949 | 8.82E-09 | 4.40E-08 |
| WIPF3 | 1.515604 | 2.426963 | 0.679259 | 9.73E-08 | 4.05E-07 |
| KATNAL1 | 1.167674 | 2.089492 | 0.839515 | 1.30E-19 | 2.34E-18 |
| RGMB | 2.37863 | 4.112938 | 0.790038 | 2.23E-10 | 1.41E-09 |
| GSKIP | 15.92423 | 10.1018 | -0.65661 | 3.50E-10 | 2.15E-09 |
| AC015911.1 | 0.972354 | 0.575771 | -0.75599 | 4.43E-05 | 0.000117 |
| TPH1 | 0.332124 | 2.341068 | 2.817375 | 3.37E-07 | 1.29E-06 |
| SCARA5 | 0.533126 | 2.403956 | 2.172863 | 1.41E-21 | 3.11E-20 |
| SHANK3 | 4.709508 | 8.368353 | 0.829367 | 3.17E-19 | 5.45E-18 |
| IGKV2D-28 | 1.915027 | 5.495882 | 1.520987 | 3.68E-07 | 1.39E-06 |
| C1QTNF12 | 1.580289 | 2.379211 | 0.590294 | 0.009107 | 0.015343 |
| CSF2RB | 3.730656 | 8.271212 | 1.148669 | 6.51E-17 | 8.80E-16 |
| AC106739.1 | 0.912197 | 1.53569 | 0.75147 | 1.22E-11 | 9.30E-11 |
| FCGR2B | 0.626374 | 1.107827 | 0.822636 | 6.69E-11 | 4.60E-10 |
| FNDC4 | 2.23292 | 3.35191 | 0.586052 | 1.92E-13 | 1.81E-12 |
| NPM3 | 16.76019 | 10.15987 | -0.72216 | 4.51E-13 | 4.10E-12 |
| TGFB2 | 1.291123 | 2.331771 | 0.852799 | 1.14E-21 | 2.56E-20 |
| MIR4324 | 0.373782 | 0.788092 | 1.076167 | 6.07E-11 | 4.21E-10 |
| NCKAP1L | 2.598533 | 4.134707 | 0.670088 | 5.48E-12 | 4.36E-11 |
| TNS1 | 9.262528 | 41.78781 | 2.173604 | 2.47E-33 | 3.84E-31 |
| AL133371.2 | 0.525618 | 1.050355 | 0.998791 | 1.28E-12 | 1.10E-11 |
| IGKV1OR22-5 | 0.428963 | 1.212266 | 1.498781 | 8.86E-08 | 3.71E-07 |
| AC026403.1 | 60.95594 | 40.18514 | -0.6011 | 3.09E-07 | 1.19E-06 |
| SLCO4A1 | 15.52062 | 8.913687 | -0.80009 | 1.28E-10 | 8.44E-10 |
| GIMAP6 | 3.478548 | 6.762914 | 0.95916 | 9.94E-22 | 2.26E-20 |
| S100A7 | 187.9185 | 14.93346 | -3.65349 | 0.007462 | 0.012804 |
| AL121992.3 | 1.228608 | 0.778962 | -0.6574 | 0.000169 | 0.000403 |
| PRICKLE2 | 0.938318 | 3.182175 | 1.761864 | 9.20E-29 | 5.24E-27 |
| SMIM2-AS1 | 0.669202 | 0.414558 | -0.69087 | 0.005814 | 0.010218 |
| NREP | 4.164285 | 6.547119 | 0.652791 | 1.41E-21 | 3.11E-20 |
| IGKV3D-15 | 3.97361 | 8.921705 | 1.166869 | 9.13E-08 | 3.81E-07 |
| LINC02038 | 2.337307 | 3.665421 | 0.649131 | 0.000194 | 0.000455 |
| CILP | 0.953313 | 5.099883 | 2.419442 | 4.94E-18 | 7.49E-17 |
| ARHGAP29 | 1.451902 | 2.326753 | 0.680374 | 3.60E-17 | 5.00E-16 |
| GFRA1 | 0.561624 | 2.777997 | 2.306368 | 4.17E-28 | 2.12E-26 |
| AL365181.3 | 7.061436 | 4.580169 | -0.62456 | 2.09E-07 | 8.25E-07 |
| CCM2L | 0.916228 | 1.713259 | 0.902966 | 1.79E-32 | 2.23E-30 |
| IGLV1-44 | 80.95948 | 136.7839 | 0.756627 | 2.78E-07 | 1.07E-06 |
| NACC2 | 5.587295 | 9.372017 | 0.74621 | 3.69E-08 | 1.65E-07 |
| ST6GALNAC5 | 0.904613 | 2.279908 | 1.333603 | 2.16E-21 | 4.64E-20 |
| UTRN | 4.92748 | 8.092893 | 0.715805 | 8.26E-17 | 1.11E-15 |
| OLFML1 | 1.870317 | 4.689173 | 1.326051 | 4.78E-35 | 1.23E-32 |
| C16orf86 | 0.437742 | 0.704242 | 0.685991 | 2.93E-17 | 4.10E-16 |
| MAN1C1 | 1.193614 | 3.170862 | 1.409538 | 7.82E-35 | 1.90E-32 |
| FAM20C | 7.604703 | 17.92286 | 1.236837 | 1.35E-32 | 1.78E-30 |
| NOTCH2 | 7.062953 | 10.63701 | 0.590749 | 5.79E-15 | 6.44E-14 |
| EFNA3 | 8.947897 | 4.051303 | -1.14316 | 5.56E-12 | 4.41E-11 |
| ARHGAP6 | 1.129389 | 1.863372 | 0.722374 | 4.06E-16 | 5.07E-15 |
| LDB3 | 0.397297 | 2.612419 | 2.717096 | 7.94E-18 | 1.17E-16 |
| S100A16 | 169.0603 | 102.9421 | -0.7157 | 7.38E-09 | 3.73E-08 |
| CYTL1 | 0.766065 | 1.609874 | 1.071409 | 4.76E-14 | 4.79E-13 |
| COL8A1 | 3.814131 | 13.50883 | 1.824476 | 6.32E-30 | 4.66E-28 |
| GPX8 | 4.256036 | 7.615322 | 0.839395 | 1.26E-16 | 1.66E-15 |
| CALML3 | 4.674867 | 0.073877 | -5.98365 | 0.022681 | 0.035042 |
| TCEAL7 | 0.728991 | 2.355958 | 1.692341 | 2.65E-32 | 3.12E-30 |
| NRG1 | 0.448705 | 0.762356 | 0.764698 | 0.000102 | 0.000252 |
| DCDC2 | 1.039838 | 1.685151 | 0.696519 | 0.000123 | 0.0003 |
| CYTH3 | 5.848325 | 9.596599 | 0.7145 | 2.65E-22 | 6.46E-21 |
| MIR200CHG | 8.101038 | 4.437468 | -0.86837 | 3.46E-13 | 3.19E-12 |
| ANKRD22 | 20.12599 | 12.01952 | -0.74368 | 3.51E-09 | 1.86E-08 |
| KIF15 | 3.630611 | 2.416753 | -0.58714 | 5.13E-11 | 3.59E-10 |
| IL7R | 3.434875 | 7.402438 | 1.107743 | 1.66E-13 | 1.59E-12 |
| IGHV1-45 | 1.440012 | 3.332118 | 1.210359 | 4.52E-11 | 3.19E-10 |
| GNAL | 0.667833 | 1.421477 | 1.089832 | 3.49E-22 | 8.32E-21 |
| AOX1 | 0.806123 | 1.884679 | 1.225247 | 1.39E-30 | 1.20E-28 |
| BCL6B | 2.098204 | 3.98512 | 0.925469 | 5.99E-22 | 1.40E-20 |
| DIRAS1 | 0.553713 | 0.881454 | 0.670747 | 1.46E-16 | 1.91E-15 |
| COL4A4 | 0.526587 | 1.839014 | 1.804189 | 3.59E-25 | 1.21E-23 |
| TREML2 | 0.627555 | 0.951795 | 0.600908 | 5.93E-08 | 2.56E-07 |
| LINC01996 | 0.567536 | 0.976173 | 0.782425 | 0.018244 | 0.028781 |
| LINC01082 | 0.720746 | 2.143414 | 1.572347 | 1.09E-17 | 1.59E-16 |
| HP | 0.478796 | 0.888961 | 0.89271 | 2.24E-11 | 1.65E-10 |
| EBPL | 24.81285 | 16.34845 | -0.60193 | 4.29E-16 | 5.35E-15 |
| PAGE5 | 0.917262 | 1.716952 | 0.904444 | 0.003905 | 0.007115 |
| TOX2 | 1.522741 | 2.65657 | 0.802894 | 4.32E-17 | 5.95E-16 |
| IGHV1-67 | 1.906525 | 5.105734 | 1.421173 | 1.09E-11 | 8.34E-11 |
| IGKV3D-7 | 0.582768 | 1.763809 | 1.597701 | 2.28E-07 | 8.96E-07 |
| IGHV1-24 | 40.33622 | 74.99863 | 0.894788 | 2.14E-08 | 9.98E-08 |
| BEND5 | 0.358715 | 1.079879 | 1.58996 | 7.30E-30 | 5.27E-28 |
| PHYHD1 | 1.610783 | 2.618294 | 0.700865 | 2.28E-16 | 2.92E-15 |
| MICU3 | 0.316195 | 0.789531 | 1.320182 | 2.20E-27 | 9.84E-26 |
| AC008537.2 | 0.790123 | 1.225134 | 0.632791 | 7.34E-07 | 2.64E-06 |
| GAS6-DT | 0.40252 | 0.608237 | 0.595574 | 2.10E-10 | 1.33E-09 |
| IGHA2 | 196.2578 | 562.2549 | 1.518474 | 8.03E-08 | 3.39E-07 |
| CNFN | 86.33313 | 5.163604 | -4.06346 | 0.019145 | 0.030086 |
| AC010099.1 | 1.088764 | 0.654511 | -0.7342 | 1.96E-05 | 5.51E-05 |
| ARHGAP23 | 6.458439 | 9.988143 | 0.629031 | 6.87E-18 | 1.02E-16 |
| IGHV3-30 | 106.588 | 220.7905 | 1.050633 | 1.24E-08 | 6.04E-08 |
| GOLGA2P10 | 4.688045 | 2.853869 | -0.71607 | 0.011202 | 0.018496 |
| ZNF367 | 5.584841 | 3.635116 | -0.61951 | 4.50E-12 | 3.61E-11 |
| IGKV1-13 | 1.333034 | 3.043026 | 1.190793 | 3.59E-05 | 9.67E-05 |
| PRKAA2 | 0.581328 | 1.362939 | 1.229297 | 1.06E-15 | 1.27E-14 |
| MBNL1-AS1 | 0.517262 | 2.040604 | 1.980028 | 2.06E-17 | 2.92E-16 |
| AC106872.7 | 2.532871 | 1.604231 | -0.65889 | 2.29E-05 | 6.37E-05 |
| PNMA5 | 1.446583 | 2.556241 | 0.821375 | 0.001485 | 0.002952 |
| LRRC4 | 0.535555 | 0.810298 | 0.597419 | 4.90E-14 | 4.93E-13 |
| ADH1A | 0.27175 | 0.996891 | 1.875153 | 1.23E-06 | 4.27E-06 |
| KIAA0355 | 4.032781 | 6.213208 | 0.623563 | 5.96E-23 | 1.53E-21 |
| MFSD4A | 2.780745 | 5.487114 | 0.980576 | 4.67E-08 | 2.05E-07 |
| MIR548V | 0.642594 | 1.036002 | 0.689047 | 0.001311 | 0.002634 |
| TRIM17 | 0.512761 | 0.776951 | 0.599536 | 1.30E-08 | 6.30E-08 |
| LDHAP4 | 4.811641 | 3.174486 | -0.60001 | 1.07E-05 | 3.15E-05 |
| GPX7 | 3.954504 | 7.292846 | 0.882985 | 2.32E-22 | 5.69E-21 |
| GUCY1B1 | 3.977904 | 10.29427 | 1.371761 | 3.17E-30 | 2.46E-28 |
| MAP1B | 3.231264 | 9.997793 | 1.629511 | 1.50E-23 | 4.16E-22 |
| SMOC1 | 2.19111 | 5.757022 | 1.393661 | 4.91E-12 | 3.93E-11 |
| P2RY14 | 1.083949 | 2.895685 | 1.417607 | 9.78E-20 | 1.79E-18 |
| HSPE1P5 | 1.066865 | 0.662482 | -0.68742 | 7.79E-06 | 2.35E-05 |
| FRMD5 | 1.763529 | 1.111857 | -0.66549 | 0.000132 | 0.00032 |
| AP000695.1 | 0.563913 | 0.866258 | 0.619323 | 1.80E-05 | 5.09E-05 |
| AL138799.3 | 1.524207 | 0.899621 | -0.76067 | 0.011563 | 0.019035 |
| CALD1 | 30.78584 | 100.7374 | 1.710261 | 2.25E-25 | 7.81E-24 |
| ARHGEF6 | 2.256857 | 4.412463 | 0.967269 | 1.01E-23 | 2.84E-22 |
| MACROD1 | 13.77504 | 8.563137 | -0.68585 | 1.40E-10 | 9.17E-10 |
| RUNX1T1 | 0.414971 | 1.171858 | 1.497714 | 8.72E-29 | 5.01E-27 |
| BCAM | 16.28847 | 29.23061 | 0.843628 | 6.50E-16 | 7.91E-15 |
| CACNA2D4 | 0.521043 | 0.814182 | 0.64395 | 0.000454 | 0.000998 |
| PALM | 1.846484 | 6.411184 | 1.79581 | 2.82E-36 | 1.11E-33 |
| PLEK2 | 31.50758 | 18.03662 | -0.80477 | 1.95E-14 | 2.06E-13 |
| MYADM | 57.35584 | 90.37243 | 0.655942 | 7.53E-14 | 7.46E-13 |
| IL33 | 4.927885 | 11.41932 | 1.212437 | 1.41E-20 | 2.80E-19 |
| MIR4697HG | 0.885454 | 1.343213 | 0.601199 | 0.010192 | 0.016992 |
| IGHV1-2 | 33.35323 | 77.86922 | 1.223227 | 9.78E-11 | 6.57E-10 |
| POU2F3 | 0.916148 | 0.43971 | -1.05903 | 0.002664 | 0.005022 |
| IL17C | 1.580388 | 0.608547 | -1.37684 | 4.66E-05 | 0.000123 |
| PDZRN3 | 2.561554 | 7.24181 | 1.499331 | 2.76E-25 | 9.43E-24 |
| CFL2 | 2.800508 | 8.31363 | 1.56979 | 1.45E-18 | 2.34E-17 |
| CNTN4 | 0.318662 | 1.007463 | 1.660628 | 2.78E-35 | 8.12E-33 |
| EFEMP2 | 4.313597 | 10.01963 | 1.215866 | 1.12E-30 | 9.71E-29 |
| MIR17HG | 0.673425 | 0.413667 | -0.70305 | 5.97E-05 | 0.000154 |
| RHEX | 0.556391 | 0.92632 | 0.735412 | 8.20E-13 | 7.19E-12 |
| SORCS2 | 0.383166 | 1.578896 | 2.042876 | 1.60E-37 | 1.15E-34 |
| SERTAD4-AS1 | 1.707865 | 4.646401 | 1.44392 | 1.34E-17 | 1.93E-16 |
| BEX3 | 22.6737 | 36.82102 | 0.69951 | 3.17E-16 | 4.01E-15 |
| KCTD12 | 21.34968 | 36.15083 | 0.759814 | 6.53E-18 | 9.77E-17 |
| SPEG | 0.561803 | 2.573407 | 2.195543 | 3.01E-18 | 4.70E-17 |
| PDE10A | 0.589378 | 1.002201 | 0.765906 | 2.88E-17 | 4.04E-16 |
| FSTL1 | 18.75956 | 48.81259 | 1.379627 | 2.61E-35 | 7.78E-33 |
| MATN3 | 1.355095 | 3.318678 | 1.292215 | 1.08E-17 | 1.58E-16 |
| RSPO3 | 2.164362 | 6.882747 | 1.669042 | 1.29E-24 | 4.01E-23 |
| ZNF366 | 0.513792 | 1.120246 | 1.124558 | 1.04E-26 | 4.35E-25 |
| ISX | 0.664276 | 2.073389 | 1.642135 | 0.000225 | 0.000522 |
| LTBP4 | 18.98459 | 38.78474 | 1.030661 | 4.21E-23 | 1.10E-21 |
| LOXL4 | 1.065639 | 2.122687 | 0.994172 | 1.88E-23 | 5.16E-22 |
| FAXDC2 | 1.344348 | 3.571234 | 1.409517 | 4.82E-22 | 1.14E-20 |
| PMP22 | 16.49742 | 30.97828 | 0.909017 | 3.33E-23 | 8.80E-22 |
| REEP2 | 0.706746 | 2.065883 | 1.547494 | 1.11E-18 | 1.81E-17 |
| TRGV3 | 0.520024 | 0.872506 | 0.746586 | 2.90E-09 | 1.57E-08 |
| HK2 | 17.56927 | 10.75664 | -0.70783 | 8.77E-11 | 5.93E-10 |
| AC008764.2 | 1.70074 | 2.802099 | 0.720345 | 7.34E-10 | 4.30E-09 |
| CAMK1D | 2.144883 | 3.596272 | 0.745603 | 2.80E-19 | 4.85E-18 |
| IGLV5-37 | 1.725461 | 6.388877 | 1.88858 | 1.08E-06 | 3.76E-06 |
| ERG | 1.137418 | 2.696348 | 1.245245 | 6.37E-34 | 1.13E-31 |
| NEXN | 4.43114 | 14.51552 | 1.711847 | 2.30E-17 | 3.24E-16 |
| TPRXL | 1.766919 | 1.143229 | -0.62812 | 0.010292 | 0.017147 |
| OGN | 1.801673 | 22.70971 | 3.6559 | 3.35E-31 | 3.27E-29 |
| CPNE7 | 4.189783 | 1.841592 | -1.18592 | 8.53E-06 | 2.55E-05 |
| LY6G6C | 2.778073 | 0.481343 | -2.52895 | 4.40E-05 | 0.000116 |
| SH3PXD2A-AS1 | 2.116106 | 0.933373 | -1.18089 | 0.001905 | 0.003696 |
| ULBP2 | 3.226156 | 2.144684 | -0.58905 | 0.00658 | 0.011429 |
| LRP1 | 16.35243 | 32.24311 | 0.979485 | 1.34E-26 | 5.46E-25 |
| CPQ | 5.076401 | 9.785969 | 0.946909 | 1.16E-29 | 8.01E-28 |
| JPH1 | 4.738977 | 2.81528 | -0.7513 | 1.37E-05 | 3.95E-05 |
| SCARF2 | 5.029798 | 12.03743 | 1.258955 | 1.97E-23 | 5.39E-22 |
| EML1 | 1.253435 | 4.216023 | 1.749996 | 9.45E-36 | 3.20E-33 |
| RECQL4 | 11.26346 | 7.336225 | -0.61854 | 1.42E-12 | 1.21E-11 |
| NOV | 2.221354 | 4.261525 | 0.93993 | 1.47E-17 | 2.11E-16 |
| ICAM2 | 1.778559 | 3.749874 | 1.076133 | 2.00E-16 | 2.59E-15 |
| CCDC136 | 0.400728 | 1.879362 | 2.229548 | 1.42E-19 | 2.55E-18 |
| ZNF710-AS1 | 2.236207 | 3.756265 | 0.748246 | 3.80E-05 | 0.000102 |
| PCDH12 | 1.571484 | 2.757492 | 0.811229 | 3.58E-20 | 6.93E-19 |
| PKIA | 0.780427 | 1.474609 | 0.917998 | 2.25E-18 | 3.55E-17 |
| IGHD3-16 | 0.538962 | 1.352911 | 1.327812 | 1.68E-05 | 4.78E-05 |
| LAMC1 | 23.36754 | 43.29567 | 0.889717 | 1.44E-28 | 7.78E-27 |
| RAB31 | 10.06925 | 19.46478 | 0.950909 | 1.17E-19 | 2.12E-18 |
| FCRL5 | 0.498947 | 0.902895 | 0.855673 | 1.43E-08 | 6.88E-08 |
| CCDC102A | 2.470889 | 4.184502 | 0.760026 | 3.95E-19 | 6.70E-18 |
| FERMT1 | 30.79378 | 19.00627 | -0.69616 | 6.22E-10 | 3.68E-09 |
| APOLD1 | 3.190018 | 5.862971 | 0.878067 | 3.30E-22 | 7.92E-21 |
| DES | 90.65089 | 676.2381 | 2.899138 | 2.87E-14 | 2.97E-13 |
| SHF | 0.828993 | 1.331001 | 0.68308 | 4.92E-16 | 6.10E-15 |
| IFI27 | 85.16783 | 55.40394 | -0.62032 | 1.65E-08 | 7.81E-08 |
| FNDC5 | 0.292192 | 1.024781 | 1.810327 | 5.20E-22 | 1.22E-20 |
| SH3RF3 | 1.612163 | 3.758189 | 1.22104 | 2.81E-32 | 3.28E-30 |
| LY96 | 9.738173 | 14.6985 | 0.593946 | 6.78E-10 | 3.99E-09 |
| SESN3 | 2.714919 | 5.631804 | 1.052688 | 2.39E-21 | 5.11E-20 |
| ITM2A | 7.100668 | 11.6818 | 0.718236 | 6.00E-18 | 9.01E-17 |
| FGF2 | 0.615987 | 1.870451 | 1.602414 | 1.36E-27 | 6.47E-26 |
| PPP2R3A | 1.441861 | 2.507932 | 0.798566 | 6.74E-14 | 6.71E-13 |
| CD93 | 8.832305 | 18.45879 | 1.063446 | 1.06E-26 | 4.43E-25 |
| EVC2 | 0.290676 | 0.7897 | 1.441894 | 6.59E-33 | 9.17E-31 |
| SUSD5 | 0.193237 | 0.807139 | 2.062442 | 7.86E-26 | 2.97E-24 |
| PBX1 | 2.103904 | 4.241087 | 1.011365 | 3.27E-22 | 7.86E-21 |
| FAM107A | 0.657856 | 2.467723 | 1.907337 | 4.11E-31 | 3.94E-29 |
| WNT4 | 1.21143 | 1.935839 | 0.676247 | 2.71E-06 | 8.88E-06 |
| BMF | 3.591103 | 6.334592 | 0.818825 | 4.62E-19 | 7.80E-18 |
| TRAV5 | 0.395655 | 0.685691 | 0.793317 | 2.81E-07 | 1.09E-06 |
| TMOD2 | 0.921643 | 1.593823 | 0.790211 | 3.69E-17 | 5.12E-16 |
| RPL22L1 | 34.9859 | 18.72917 | -0.90149 | 6.20E-08 | 2.66E-07 |
| TRO | 0.289675 | 0.814517 | 1.491508 | 2.91E-34 | 5.49E-32 |
| ARSB | 2.152196 | 3.862244 | 0.84363 | 1.74E-25 | 6.23E-24 |
| CD1C | 0.940064 | 2.57209 | 1.452109 | 2.10E-18 | 3.33E-17 |
| WISP1 | 2.033277 | 4.198392 | 1.04603 | 1.97E-14 | 2.08E-13 |
| IGLV5-48 | 0.931782 | 1.557511 | 0.741178 | 9.25E-06 | 2.75E-05 |
| DSE | 1.334369 | 2.317698 | 0.796535 | 3.83E-18 | 5.90E-17 |
| ATP8B2 | 2.220359 | 6.527171 | 1.555665 | 4.07E-40 | 7.66E-37 |
| PDE5A | 2.902717 | 5.492995 | 0.920189 | 1.06E-13 | 1.04E-12 |
| PLEKHO2 | 6.930835 | 11.00426 | 0.666961 | 2.66E-19 | 4.62E-18 |
| FOSL1 | 22.31968 | 10.33118 | -1.11131 | 9.54E-07 | 3.37E-06 |
| RPL12P50 | 1.11258 | 0.638898 | -0.80025 | 6.96E-05 | 0.000178 |
| SENCR | 0.388288 | 0.711668 | 0.874076 | 1.46E-19 | 2.61E-18 |
| PANX2 | 0.651837 | 1.083256 | 0.732791 | 0.00036 | 0.000806 |
| FCRL2 | 0.325204 | 0.682463 | 1.069407 | 2.04E-08 | 9.53E-08 |
| SARM1 | 0.721791 | 1.098027 | 0.605259 | 1.61E-16 | 2.10E-15 |
| SLC26A2 | 2.347262 | 3.922411 | 0.740761 | 2.61E-12 | 2.16E-11 |
| UPK1B | 3.056097 | 4.676572 | 0.613761 | 0.000272 | 0.000623 |
| FMNL1 | 4.010894 | 6.085161 | 0.601372 | 2.51E-13 | 2.35E-12 |
| DYSF | 3.106727 | 5.808062 | 0.902661 | 1.56E-20 | 3.09E-19 |
| IL6 | 2.657482 | 5.354404 | 1.010666 | 0.000544 | 0.001181 |
| APLP1 | 2.564308 | 4.375206 | 0.770781 | 2.47E-10 | 1.55E-09 |
| RAB3IL1 | 2.845816 | 5.498072 | 0.950083 | 8.77E-21 | 1.77E-19 |
| CCKAR | 0.17653 | 0.977951 | 2.469845 | 1.14E-08 | 5.59E-08 |
| CR1 | 0.32755 | 0.772453 | 1.23773 | 3.49E-13 | 3.22E-12 |
| PSAT1 | 27.78705 | 13.73646 | -1.0164 | 2.79E-16 | 3.54E-15 |
| CLU | 32.8463 | 72.146 | 1.135188 | 2.51E-15 | 2.88E-14 |
| SDC1 | 99.68438 | 63.13071 | -0.65903 | 5.51E-06 | 1.70E-05 |
| IGLV3-10 | 33.01873 | 100.376 | 1.604057 | 3.13E-06 | 1.01E-05 |
| RAB9B | 0.325987 | 1.058392 | 1.69899 | 4.17E-24 | 1.22E-22 |
| RPL12P32 | 0.737857 | 0.481267 | -0.6165 | 1.27E-06 | 4.41E-06 |
| LRRC26 | 11.58151 | 5.421633 | -1.09502 | 5.50E-05 | 0.000143 |
| GUCY1A2 | 0.324542 | 0.712499 | 1.134484 | 3.16E-24 | 9.38E-23 |
| SPRR1B | 161.0196 | 13.22866 | -3.6055 | 0.00013 | 0.000316 |
| AL161431.1 | 4.940466 | 2.001221 | -1.30377 | 9.17E-08 | 3.83E-07 |
| EBF1 | 1.688174 | 3.064807 | 0.860333 | 7.31E-26 | 2.79E-24 |
| HMGCS2 | 34.06879 | 55.6328 | 0.707485 | 4.99E-07 | 1.84E-06 |
| IGHV1-12 | 0.727161 | 2.965237 | 2.027801 | 3.21E-08 | 1.44E-07 |
| NPTXR | 1.301974 | 4.015011 | 1.624703 | 6.31E-27 | 2.72E-25 |
| SOAT2 | 0.267741 | 1.221796 | 2.190096 | 2.35E-08 | 1.08E-07 |
| LIFR | 1.231153 | 2.630488 | 1.09532 | 8.65E-19 | 1.42E-17 |
| MACF1 | 4.649489 | 7.534768 | 0.696491 | 2.81E-16 | 3.57E-15 |
| PERM1 | 0.728611 | 0.455813 | -0.67671 | 1.21E-07 | 4.96E-07 |
| ACTA1 | 0.413625 | 0.811299 | 0.971909 | 0.004292 | 0.007749 |
| PCDHGB7 | 0.679459 | 1.504581 | 1.146903 | 1.42E-27 | 6.73E-26 |
| IQCN | 0.426091 | 0.726418 | 0.769637 | 2.15E-09 | 1.18E-08 |
| SORBS2 | 1.656743 | 3.017459 | 0.864985 | 3.13E-12 | 2.55E-11 |
| FOXN3 | 6.582841 | 10.51839 | 0.676132 | 6.90E-20 | 1.29E-18 |
| SULT2A1 | 0.312847 | 3.555196 | 3.506398 | 0.002234 | 0.004286 |
| IGFBP3 | 39.96095 | 69.26224 | 0.793478 | 8.54E-11 | 5.79E-10 |
| ORM2 | 4.180074 | 9.112157 | 1.124264 | 0.004213 | 0.007628 |
| SEMA3D | 0.387947 | 0.651296 | 0.747455 | 8.11E-20 | 1.51E-18 |
| TRAV16 | 0.536131 | 0.833915 | 0.637315 | 0.00012 | 0.000294 |
| SULT1C4 | 0.459492 | 1.307181 | 1.508347 | 1.03E-28 | 5.74E-27 |
| GAS7 | 2.159101 | 6.028727 | 1.481423 | 5.17E-32 | 5.65E-30 |
| PTPRS | 3.484495 | 6.060441 | 0.798473 | 1.11E-12 | 9.60E-12 |
| CXCL14 | 38.22846 | 67.13446 | 0.812406 | 2.61E-07 | 1.02E-06 |
| NME1 | 21.6136 | 13.75608 | -0.65187 | 3.78E-14 | 3.86E-13 |
| SLC29A4 | 1.743638 | 4.648346 | 1.414617 | 3.42E-08 | 1.53E-07 |
| IGLV3-25 | 105.2885 | 216.9749 | 1.043181 | 8.27E-07 | 2.96E-06 |
| ADAMTS2 | 7.884394 | 12.84878 | 0.704559 | 3.39E-07 | 1.29E-06 |
| SLC26A3 | 1.701652 | 5.634969 | 1.727472 | 0.001766 | 0.003453 |
| FLNC | 6.597811 | 50.02385 | 2.922557 | 1.21E-19 | 2.20E-18 |
| INSM1 | 3.250792 | 11.24135 | 1.789952 | 0.000252 | 0.000579 |
| RET | 0.883763 | 1.616652 | 0.871278 | 2.08E-24 | 6.27E-23 |
| COL8A2 | 1.769725 | 4.746326 | 1.423286 | 1.98E-27 | 9.00E-26 |
| TSPYL5 | 2.131012 | 4.613613 | 1.114358 | 2.24E-23 | 6.05E-22 |
| GRAP2 | 0.613871 | 0.92788 | 0.596003 | 4.07E-07 | 1.53E-06 |
| CDCA2 | 5.054901 | 3.127755 | -0.69256 | 1.48E-11 | 1.11E-10 |
| MIR421 | 0.694287 | 1.182457 | 0.768184 | 1.61E-07 | 6.46E-07 |
| TENM3 | 0.513972 | 1.204883 | 1.229133 | 5.99E-14 | 5.98E-13 |
| SPDYC | 1.872767 | 1.087365 | -0.78434 | 0.006577 | 0.011429 |
| AC087491.1 | 1.582455 | 0.621102 | -1.34926 | 0.000194 | 0.000455 |
| C7 | 3.1077 | 21.41033 | 2.784388 | 1.82E-25 | 6.41E-24 |
| IGHGP | 7.763291 | 12.17336 | 0.648988 | 2.00E-05 | 5.63E-05 |
| TPST1 | 3.307633 | 6.097421 | 0.8824 | 3.06E-22 | 7.39E-21 |
| AC243960.1 | 0.591868 | 0.890267 | 0.588963 | 9.22E-07 | 3.27E-06 |
| GIP | 0.2309 | 0.939761 | 2.025026 | 0.002029 | 0.003915 |
| RDX | 5.067467 | 7.728969 | 0.609011 | 6.68E-13 | 5.90E-12 |
| CCDC152 | 0.581693 | 0.893907 | 0.619867 | 2.44E-12 | 2.02E-11 |
| IGKV2-30 | 14.04197 | 36.79864 | 1.389907 | 8.79E-08 | 3.69E-07 |
| PMAIP1 | 7.501829 | 3.795448 | -0.98297 | 4.47E-11 | 3.16E-10 |
| COL10A1 | 9.326224 | 15.37517 | 0.721238 | 4.54E-06 | 1.43E-05 |
| FAM25A | 1.972884 | 0.245673 | -3.0055 | 0.001041 | 0.002135 |
| MKX | 0.505396 | 1.130427 | 1.161383 | 1.95E-16 | 2.53E-15 |
| IGHV4-34 | 52.55133 | 134.5296 | 1.356124 | 1.02E-07 | 4.22E-07 |
| SNHG25 | 32.4277 | 14.07318 | -1.20428 | 2.36E-08 | 1.09E-07 |
| MZB1 | 7.463338 | 11.61285 | 0.637829 | 3.78E-06 | 1.21E-05 |
| SCARF1 | 1.657926 | 2.585049 | 0.640813 | 1.07E-17 | 1.55E-16 |
| AC006059.1 | 0.365377 | 0.641565 | 0.812209 | 3.29E-17 | 4.59E-16 |
| CELF2 | 2.585059 | 5.7093 | 1.143117 | 1.10E-16 | 1.45E-15 |
| ZNF667-AS1 | 0.62454 | 1.965507 | 1.654036 | 6.08E-33 | 8.59E-31 |
| VSTM4 | 0.876689 | 2.816535 | 1.683784 | 1.79E-32 | 2.23E-30 |
| LXN | 9.098723 | 13.71208 | 0.591712 | 1.57E-07 | 6.34E-07 |
| GPA33 | 19.57032 | 9.527431 | -1.03851 | 0.000792 | 0.001667 |
| BANCR | 5.269648 | 0.275175 | -4.25928 | 0.008292 | 0.014097 |
| PBX3 | 4.265642 | 8.265435 | 0.954328 | 7.13E-17 | 9.59E-16 |
| HSPG2 | 28.36812 | 59.71343 | 1.073785 | 3.21E-23 | 8.51E-22 |
| HVCN1 | 1.75023 | 2.646198 | 0.596376 | 6.90E-15 | 7.61E-14 |
| FRY | 1.010746 | 1.857513 | 0.877953 | 1.50E-25 | 5.39E-24 |
| TP73-AS1 | 0.856928 | 1.923165 | 1.166236 | 6.67E-26 | 2.56E-24 |
| RAB6B | 0.890307 | 1.731732 | 0.959841 | 6.14E-20 | 1.16E-18 |
| TXNP1 | 0.860288 | 0.461261 | -0.89924 | 1.33E-06 | 4.59E-06 |
| NOP56P1 | 1.207381 | 0.721038 | -0.74373 | 3.56E-05 | 9.58E-05 |
| OBSL1 | 2.629229 | 4.812494 | 0.872145 | 7.01E-17 | 9.44E-16 |
| AC147067.2 | 0.518479 | 0.807555 | 0.639274 | 1.24E-07 | 5.06E-07 |
| CXCL11 | 15.33029 | 6.146238 | -1.31861 | 0.021553 | 0.033476 |
| KDR | 3.767406 | 6.943401 | 0.882071 | 2.39E-22 | 5.85E-21 |
| FGL2 | 9.152373 | 19.02677 | 1.055813 | 1.05E-13 | 1.02E-12 |
| SNORA11 | 0.745096 | 1.703247 | 1.19279 | 0.022011 | 0.034109 |
| NLGN4Y | 0.290452 | 0.753779 | 1.375841 | 8.69E-09 | 4.34E-08 |
| AC106900.2 | 1.332472 | 0.765323 | -0.79996 | 5.41E-06 | 1.67E-05 |
| SERTAD4 | 1.670225 | 2.914858 | 0.803384 | 1.22E-10 | 8.04E-10 |
| AC104958.2 | 3.913788 | 1.645057 | -1.25043 | 0.006638 | 0.011523 |
| NR2F1 | 3.823058 | 10.67455 | 1.481377 | 6.47E-30 | 4.75E-28 |
| PCDHB7 | 0.341208 | 0.747236 | 1.130914 | 4.48E-21 | 9.36E-20 |
| OLFML2B | 9.111374 | 19.51966 | 1.099188 | 3.30E-13 | 3.05E-12 |
| VCAN | 9.827059 | 20.37964 | 1.052297 | 2.48E-19 | 4.31E-18 |
| RF02119 | 1.577782 | 2.461243 | 0.641489 | 0.000766 | 0.001616 |
| C19orf84 | 0.326805 | 0.673695 | 1.043664 | 0.008507 | 0.014424 |
| WIPF1 | 6.560171 | 11.15928 | 0.766439 | 3.34E-19 | 5.73E-18 |
| ST3GAL3 | 1.106823 | 2.00327 | 0.855933 | 5.76E-22 | 1.35E-20 |
| MMRN1 | 0.588704 | 2.420937 | 2.039951 | 8.91E-29 | 5.10E-27 |
| PLTP | 32.44117 | 62.04948 | 0.935593 | 4.12E-15 | 4.66E-14 |
| SLC22A31 | 0.815637 | 1.385561 | 0.76447 | 0.000705 | 0.001499 |
| HEG1 | 5.580644 | 13.79355 | 1.30549 | 1.14E-38 | 1.08E-35 |
| ANLN | 14.59242 | 9.400606 | -0.63439 | 3.12E-11 | 2.24E-10 |
| DBF4 | 6.717567 | 4.186899 | -0.68206 | 2.45E-15 | 2.81E-14 |
| TRAV3 | 0.587836 | 1.003728 | 0.771884 | 3.24E-06 | 1.05E-05 |
| TRAJ34 | 0.364837 | 0.636525 | 0.802966 | 8.84E-06 | 2.64E-05 |
| INAFM2 | 8.765188 | 15.95603 | 0.864245 | 5.91E-11 | 4.11E-10 |
| GBGT1 | 1.314976 | 2.464877 | 0.906479 | 4.70E-21 | 9.76E-20 |
| ISLR2 | 0.252751 | 0.844789 | 1.740875 | 1.82E-27 | 8.35E-26 |
| ZCCHC24 | 3.728045 | 13.10277 | 1.813381 | 1.83E-34 | 3.78E-32 |
| KIF7 | 1.010648 | 1.637236 | 0.695982 | 2.08E-19 | 3.66E-18 |
| SGCE | 4.156723 | 9.327625 | 1.166063 | 2.14E-25 | 7.48E-24 |
| CSTB | 141.0312 | 64.03312 | -1.13912 | 8.55E-10 | 4.98E-09 |
| PDGFD | 1.911951 | 4.320065 | 1.176007 | 7.24E-26 | 2.77E-24 |
| GNG2 | 2.378307 | 3.828008 | 0.686659 | 1.63E-18 | 2.60E-17 |
| LINC02550 | 0.306782 | 0.82666 | 1.430079 | 7.74E-20 | 1.45E-18 |
| TPMT | 19.27359 | 12.69951 | -0.60185 | 7.64E-12 | 5.95E-11 |
| FOXS1 | 2.720564 | 5.898457 | 1.116432 | 1.42E-14 | 1.52E-13 |
| LAMA1 | 0.198563 | 1.085549 | 2.450752 | 0.002094 | 0.004034 |
| FMNL3 | 2.9106 | 5.301523 | 0.86509 | 1.43E-26 | 5.80E-25 |
| TUB | 0.256009 | 1.044203 | 2.028137 | 1.07E-35 | 3.48E-33 |
| ZBTB16 | 0.282601 | 1.34982 | 2.255927 | 2.09E-22 | 5.16E-21 |
| RAMP2 | 17.20075 | 32.97224 | 0.938781 | 9.84E-25 | 3.12E-23 |
| ANKDD1A | 0.603235 | 0.955821 | 0.66402 | 1.70E-17 | 2.44E-16 |
| EDIL3 | 4.262131 | 9.175985 | 1.106288 | 7.09E-23 | 1.81E-21 |
| KCNK3 | 0.856594 | 1.893503 | 1.144374 | 2.70E-21 | 5.74E-20 |
| RCN3 | 15.96652 | 24.84736 | 0.638042 | 9.59E-11 | 6.45E-10 |
| ASGR2 | 0.332822 | 1.20863 | 1.86055 | 1.18E-14 | 1.27E-13 |
| SVEP1 | 0.631773 | 2.932224 | 2.214517 | 1.04E-36 | 5.18E-34 |
| C2CD4A | 7.687751 | 3.785286 | -1.02216 | 2.01E-05 | 5.65E-05 |
| AC134879.2 | 0.658706 | 1.943155 | 1.560694 | 3.76E-09 | 1.99E-08 |
| PLEKHM3 | 0.878751 | 1.352881 | 0.622508 | 4.46E-15 | 5.00E-14 |
| DHRS2 | 3.378299 | 1.995614 | -0.75946 | 0.024775 | 0.037955 |
| LPL | 1.518911 | 3.121942 | 1.039407 | 4.03E-16 | 5.03E-15 |
| MLN | 0.407236 | 1.687087 | 2.050598 | 3.80E-10 | 2.33E-09 |
| AL359182.1 | 0.415519 | 0.642594 | 0.628993 | 2.57E-08 | 1.18E-07 |
| CNN1 | 22.78778 | 176.0738 | 2.949848 | 1.94E-19 | 3.43E-18 |
| AL133415.1 | 0.772246 | 1.615001 | 1.064403 | 9.27E-19 | 1.52E-17 |
| STAT5B | 8.129468 | 12.31093 | 0.598707 | 1.11E-21 | 2.51E-20 |
| AL512274.1 | 3.447423 | 2.163943 | -0.67186 | 0.00053 | 0.001153 |
| JAZF1 | 2.674005 | 4.976789 | 0.896213 | 7.12E-28 | 3.45E-26 |
| PRICKLE1 | 0.425453 | 1.147372 | 1.431262 | 4.44E-29 | 2.75E-27 |
| THBS2 | 19.13951 | 38.03233 | 0.990672 | 1.02E-11 | 7.85E-11 |
| GAB3 | 0.755156 | 1.295609 | 0.778783 | 2.61E-16 | 3.33E-15 |
| C10orf91 | 0.866611 | 0.319402 | -1.44001 | 1.38E-09 | 7.85E-09 |
| MSR1 | 2.916487 | 4.508292 | 0.628349 | 7.65E-08 | 3.24E-07 |
| IGHV5-78 | 1.955321 | 3.06377 | 0.647902 | 2.40E-12 | 2.00E-11 |
| TCF4 | 1.75657 | 3.715685 | 1.080867 | 1.46E-33 | 2.44E-31 |
| KRT13 | 185.8085 | 1.142908 | -7.34496 | 0.000151 | 0.000362 |
| CCR8 | 0.451594 | 0.719848 | 0.672667 | 7.69E-09 | 3.87E-08 |
| AC036176.3 | 0.660285 | 0.344844 | -0.93714 | 7.17E-05 | 0.000183 |
| IGKV2D-29 | 9.984204 | 22.3423 | 1.162058 | 1.22E-06 | 4.23E-06 |
| EMID1 | 1.00891 | 2.301815 | 1.189975 | 1.80E-14 | 1.90E-13 |
| CTD-3080P12.3 | 0.297436 | 0.809698 | 1.444806 | 3.15E-06 | 1.02E-05 |
| ZNF542P | 0.571336 | 1.476358 | 1.369631 | 1.76E-30 | 1.45E-28 |
| IGHV3OR16-6 | 0.900196 | 1.9616 | 1.12372 | 1.49E-09 | 8.40E-09 |
| CD300E | 0.519663 | 1.096205 | 1.076869 | 0.000159 | 0.000379 |
| TDRP | 0.476522 | 1.341677 | 1.493422 | 3.97E-30 | 3.02E-28 |
| PSME2P2 | 3.582971 | 2.268305 | -0.65954 | 2.75E-10 | 1.72E-09 |
| CD207 | 0.370335 | 0.786236 | 1.086133 | 5.24E-14 | 5.26E-13 |
| NELL2 | 0.462066 | 0.847571 | 0.875234 | 0.000303 | 0.000687 |
| FAT4 | 0.592808 | 2.163115 | 1.867474 | 3.62E-40 | 7.66E-37 |
| KCTD7 | 1.040793 | 1.71927 | 0.724114 | 9.35E-20 | 1.73E-18 |
| ADRA1B | 0.360173 | 0.678234 | 0.913092 | 1.53E-08 | 7.30E-08 |
| ADRA2A | 9.387561 | 14.12465 | 0.589393 | 2.00E-09 | 1.11E-08 |
| IGLV2-28 | 0.431602 | 1.59962 | 1.889957 | 1.03E-07 | 4.26E-07 |
| PTTG1 | 21.32093 | 12.79547 | -0.73664 | 1.81E-17 | 2.57E-16 |
| HSPB8 | 5.459391 | 19.64615 | 1.847434 | 1.30E-24 | 4.04E-23 |
| PDLIM3 | 4.327471 | 15.76227 | 1.864879 | 2.23E-25 | 7.76E-24 |
| PHYHIP | 0.359913 | 0.720241 | 1.000831 | 6.44E-15 | 7.13E-14 |
| CRYBG2 | 4.822522 | 2.999954 | -0.68485 | 3.09E-05 | 8.40E-05 |
| SETBP1 | 0.80768 | 2.719413 | 1.75144 | 4.65E-33 | 6.74E-31 |
| AC104843.1 | 1.432242 | 0.892874 | -0.68175 | 1.10E-05 | 3.23E-05 |
| RHOB | 96.91289 | 171.5353 | 0.823745 | 2.43E-11 | 1.77E-10 |
| FLT1 | 3.270258 | 5.119239 | 0.646525 | 3.00E-14 | 3.10E-13 |
| KCNG1 | 0.25935 | 0.760929 | 1.552862 | 2.66E-20 | 5.21E-19 |
| CDKN1C | 6.74579 | 11.60436 | 0.782607 | 3.71E-12 | 3.00E-11 |
| TMEM71 | 0.522492 | 0.960937 | 0.879033 | 3.78E-14 | 3.86E-13 |
| CHRM2 | 0.208903 | 1.490649 | 2.835038 | 6.46E-13 | 5.72E-12 |
| TBX10 | 0.597746 | 1.079402 | 0.852627 | 0.000722 | 0.001531 |
| ALOX15 | 0.574247 | 1.065824 | 0.892225 | 0.000861 | 0.001799 |
| TP73 | 1.089538 | 0.71597 | -0.60575 | 0.001949 | 0.003773 |
| GPC3 | 7.483891 | 23.42335 | 1.646087 | 1.12E-19 | 2.03E-18 |
| AC007160.1 | 0.697068 | 0.443767 | -0.6515 | 8.82E-07 | 3.14E-06 |
| BLK | 0.573692 | 1.440344 | 1.328065 | 1.29E-12 | 1.10E-11 |
| FGFBP1 | 11.31033 | 4.469754 | -1.33937 | 1.58E-06 | 5.37E-06 |
| ITIH5 | 0.94876 | 3.356787 | 1.822966 | 1.77E-32 | 2.23E-30 |
| GAPDHP69 | 0.644606 | 0.429666 | -0.5852 | 6.18E-06 | 1.89E-05 |
| RN7SL648P | 0.377764 | 0.64899 | 0.78071 | 7.53E-05 | 0.000191 |
| SNTA1 | 5.315176 | 8.616726 | 0.697022 | 2.13E-11 | 1.56E-10 |
| PSME2P6 | 1.534539 | 0.7863 | -0.96465 | 5.63E-09 | 2.90E-08 |
| CDH2 | 0.772025 | 1.711259 | 1.148339 | 5.39E-17 | 7.33E-16 |
| PLD4 | 0.458791 | 1.292695 | 1.494472 | 8.97E-22 | 2.04E-20 |
| PIEZO2 | 0.38281 | 0.861543 | 1.170295 | 7.23E-22 | 1.67E-20 |
| DTX1 | 1.683935 | 3.604016 | 1.097769 | 2.02E-19 | 3.57E-18 |
| AL049840.5 | 0.679318 | 1.026402 | 0.595438 | 1.48E-05 | 4.25E-05 |
| DIXDC1 | 1.791216 | 5.25304 | 1.552213 | 3.00E-22 | 7.26E-21 |
| SNORD14E | 3.817428 | 2.115258 | -0.85177 | 5.79E-09 | 2.97E-08 |
| HIST1H3C | 0.734937 | 0.477018 | -0.62358 | 0.006104 | 0.010683 |
| CLEC3B | 1.676199 | 5.708081 | 1.767813 | 7.14E-30 | 5.18E-28 |
| ETHE1 | 55.41496 | 34.79477 | -0.67141 | 1.86E-07 | 7.40E-07 |
| AC018629.1 | 5.212589 | 2.585814 | -1.01138 | 1.37E-06 | 4.72E-06 |
| FNDC1 | 5.192891 | 18.10516 | 1.801791 | 5.10E-22 | 1.20E-20 |
| FILIP1 | 0.712693 | 2.862765 | 2.006058 | 2.48E-22 | 6.06E-21 |
| RGS7BP | 0.257523 | 0.927283 | 1.84831 | 3.05E-33 | 4.66E-31 |
| ENPP2 | 3.497858 | 7.112808 | 1.023947 | 2.65E-17 | 3.72E-16 |
| RNF150 | 0.62424 | 2.747716 | 2.138061 | 1.24E-27 | 5.89E-26 |
| BGN | 158.5336 | 299.4478 | 0.917516 | 1.57E-13 | 1.50E-12 |
| IL13RA2 | 0.703394 | 1.148153 | 0.706909 | 0.003062 | 0.005697 |
| AC053503.6 | 0.153691 | 1.115607 | 2.859721 | 6.14E-08 | 2.64E-07 |
| TRBV6-1 | 0.669926 | 1.142717 | 0.770396 | 1.51E-05 | 4.34E-05 |
| MOCS1 | 1.488503 | 2.929512 | 0.976798 | 9.95E-26 | 3.68E-24 |
| SNORD116-4 | 0.440419 | 1.442872 | 1.711994 | 3.75E-19 | 6.38E-18 |
| LIPM | 1.468252 | 0.899194 | -0.70739 | 8.98E-08 | 3.76E-07 |
| AC105460.1 | 20.46887 | 3.598013 | -2.50816 | 0.005838 | 0.010257 |
| PDE1A | 0.56759 | 1.969522 | 1.794923 | 3.67E-41 | 2.31E-37 |
| ZNF134 | 1.911514 | 3.240946 | 0.761699 | 5.91E-16 | 7.24E-15 |
| COL5A1 | 24.65811 | 49.60244 | 1.008349 | 7.92E-16 | 9.61E-15 |
| MEX3B | 0.747416 | 1.226453 | 0.714509 | 3.34E-18 | 5.18E-17 |
| IGKV5-2 | 3.671548 | 14.84053 | 2.015082 | 4.36E-05 | 0.000116 |
| ELN-AS1 | 0.916841 | 1.457408 | 0.668661 | 8.98E-09 | 4.46E-08 |
| AL512413.1 | 1.190905 | 0.779303 | -0.6118 | 0.000107 | 0.000263 |
| SLIT3 | 1.25793 | 5.114771 | 2.023618 | 8.82E-35 | 2.07E-32 |
| MCTP1 | 0.788187 | 1.267917 | 0.68585 | 5.39E-17 | 7.33E-16 |
| CILP2 | 0.856889 | 1.886422 | 1.138472 | 7.96E-09 | 3.99E-08 |
| ASB2 | 1.102169 | 4.646879 | 2.075917 | 9.52E-19 | 1.56E-17 |
| PCP4L1 | 1.011222 | 2.029022 | 1.004684 | 6.01E-15 | 6.67E-14 |
| NES | 5.275519 | 8.765781 | 0.73257 | 2.30E-17 | 3.24E-16 |
| ADAM19 | 3.973605 | 6.926267 | 0.801629 | 3.67E-18 | 5.67E-17 |
| TOX | 2.502875 | 5.392126 | 1.107268 | 3.72E-13 | 3.42E-12 |
| CNR1 | 0.34382 | 1.214671 | 1.820841 | 1.09E-22 | 2.76E-21 |
| AC024075.1 | 2.257041 | 3.454468 | 0.614031 | 1.56E-09 | 8.75E-09 |
| BIK | 24.77585 | 13.73782 | -0.85078 | 2.06E-11 | 1.52E-10 |
| ASAP3 | 2.273463 | 4.016527 | 0.821057 | 8.49E-24 | 2.41E-22 |
| MEOX2 | 0.491263 | 2.15506 | 2.133159 | 6.37E-23 | 1.64E-21 |
| RASGRP2 | 0.778875 | 1.943567 | 1.319244 | 2.70E-22 | 6.57E-21 |
| SLC1A7 | 0.442678 | 0.718609 | 0.698949 | 5.76E-18 | 8.66E-17 |
| TXNP5 | 0.997623 | 0.622287 | -0.68091 | 1.62E-05 | 4.62E-05 |
| LIMS2 | 1.894395 | 6.737088 | 1.830388 | 8.53E-29 | 4.94E-27 |
| PAPPA | 0.883302 | 1.724826 | 0.965472 | 3.84E-08 | 1.70E-07 |
| PLEKHN1 | 2.914842 | 1.770423 | -0.71932 | 8.73E-07 | 3.11E-06 |
| CSDC2 | 0.436405 | 1.249152 | 1.517209 | 3.82E-24 | 1.12E-22 |
| MAP1A | 1.556338 | 5.166562 | 1.731049 | 8.82E-30 | 6.26E-28 |
| TRBJ2-2P | 2.150846 | 3.602804 | 0.744216 | 0.001667 | 0.003277 |
| WSCD2 | 0.282337 | 0.995718 | 1.81832 | 5.08E-16 | 6.26E-15 |
| AL133346.1 | 0.770622 | 1.298175 | 0.75239 | 1.95E-11 | 1.44E-10 |
| MEF2C | 2.003919 | 4.228027 | 1.07716 | 2.10E-30 | 1.71E-28 |
| ARL10 | 0.48546 | 0.918304 | 0.91962 | 2.86E-20 | 5.58E-19 |
| PCDH18 | 2.684001 | 5.899074 | 1.136103 | 1.59E-30 | 1.32E-28 |
| IGSF21 | 0.374152 | 0.626461 | 0.7436 | 1.33E-15 | 1.58E-14 |
| IGKV2-24 | 18.31649 | 49.46392 | 1.433234 | 4.42E-07 | 1.65E-06 |
| ANOS1 | 1.388902 | 2.275146 | 0.712015 | 7.66E-10 | 4.48E-09 |
| CASS4 | 0.459571 | 0.746927 | 0.70068 | 2.90E-11 | 2.10E-10 |
| ADAMTS9 | 1.766351 | 3.38283 | 0.937458 | 5.32E-20 | 1.01E-18 |
| KIR2DL4 | 0.64345 | 0.365244 | -0.81697 | 0.000364 | 0.000815 |
| RNU7-48P | 0.660042 | 0.399807 | -0.72326 | 0.016525 | 0.026307 |
| HECTD2 | 0.715544 | 1.157258 | 0.693598 | 8.47E-14 | 8.35E-13 |
| IGHD3-9 | 1.564364 | 3.769995 | 1.268986 | 1.57E-07 | 6.32E-07 |
| PTPRM | 2.368704 | 5.584511 | 1.237333 | 5.00E-40 | 8.48E-37 |
| SPRR1A | 136.3314 | 5.525309 | -4.62492 | 0.001915 | 0.003714 |
| PLCL1 | 0.409386 | 0.944385 | 1.205913 | 2.84E-30 | 2.22E-28 |
| IGHV4OR15-8 | 0.554655 | 1.150408 | 1.052483 | 3.39E-07 | 1.29E-06 |
| KRT18 | 353.8319 | 214.0371 | -0.7252 | 1.67E-14 | 1.77E-13 |
| SOBP | 1.199364 | 2.715929 | 1.179176 | 1.25E-19 | 2.27E-18 |
| RPS7P3 | 3.638672 | 2.343223 | -0.63492 | 7.06E-05 | 0.00018 |
| PRKN | 0.330244 | 0.713278 | 1.110933 | 2.38E-15 | 2.73E-14 |
| ADAMTS12 | 2.605315 | 4.228971 | 0.698849 | 5.80E-05 | 0.00015 |
| SKA3 | 8.847192 | 5.855624 | -0.5954 | 1.37E-14 | 1.47E-13 |
| RNF122 | 3.592447 | 5.428696 | 0.595639 | 1.16E-13 | 1.13E-12 |
| FZD8 | 4.112188 | 7.901843 | 0.942283 | 5.49E-21 | 1.13E-19 |
| PTPRC | 7.313079 | 11.2416 | 0.620296 | 2.40E-08 | 1.10E-07 |
| EPHB1 | 0.751584 | 1.213598 | 0.691283 | 1.38E-09 | 7.83E-09 |
| NIPSNAP3B | 0.365578 | 0.718115 | 0.974036 | 6.32E-18 | 9.46E-17 |
| HMCN2 | 0.436612 | 1.230113 | 1.494367 | 3.33E-20 | 6.48E-19 |
| RRM2 | 23.77635 | 14.32772 | -0.73072 | 5.04E-16 | 6.22E-15 |
| DZIP1 | 0.667354 | 1.587766 | 1.250474 | 1.52E-32 | 1.97E-30 |
| IGKV1D-27 | 1.59097 | 4.024828 | 1.33902 | 1.08E-07 | 4.45E-07 |
| RPS2P35 | 0.768827 | 0.47075 | -0.7077 | 7.86E-08 | 3.32E-07 |
| ZNF331 | 1.505601 | 2.697732 | 0.841407 | 8.75E-15 | 9.56E-14 |
| ST6GALNAC3 | 0.377689 | 0.685811 | 0.860611 | 1.28E-25 | 4.68E-24 |
| TNS4 | 46.42565 | 18.69632 | -1.31217 | 1.63E-07 | 6.51E-07 |
| PDZD4 | 0.329861 | 1.510518 | 2.195111 | 2.08E-29 | 1.36E-27 |
| TSPAN11 | 0.974355 | 3.063161 | 1.652502 | 5.22E-28 | 2.61E-26 |
| MALL | 4.551294 | 2.84943 | -0.6756 | 0.008903 | 0.015036 |
| PPP1R12B | 4.430424 | 18.91914 | 2.09433 | 1.20E-16 | 1.58E-15 |
| PCDHB5 | 0.544119 | 1.482435 | 1.445974 | 1.73E-25 | 6.18E-24 |
| STEAP4 | 1.208469 | 2.701971 | 1.160832 | 2.02E-16 | 2.61E-15 |
| IGFBP6 | 10.22295 | 17.71376 | 0.793058 | 3.18E-22 | 7.66E-21 |
| PIGCP1 | 2.41984 | 3.981606 | 0.718439 | 3.81E-14 | 3.88E-13 |
| ZFPM2 | 0.395634 | 1.158866 | 1.550474 | 2.41E-28 | 1.26E-26 |
| RGS18 | 0.668503 | 1.139474 | 0.769363 | 5.37E-12 | 4.27E-11 |
| PTMAP5 | 13.69988 | 7.851338 | -0.80315 | 1.16E-13 | 1.13E-12 |
| CTNND2 | 0.225757 | 0.832115 | 1.882014 | 3.39E-24 | 1.00E-22 |
| AL031229.1 | 1.449783 | 0.893139 | -0.69888 | 1.73E-08 | 8.19E-08 |
| MCM10 | 2.953107 | 1.945012 | -0.60246 | 1.77E-13 | 1.69E-12 |
| NKX3-2 | 0.468177 | 1.796493 | 1.940057 | 3.60E-12 | 2.92E-11 |
| ADAM12 | 1.915968 | 3.032594 | 0.662479 | 0.002673 | 0.005036 |
| ANGPTL1 | 0.713002 | 4.49881 | 2.657565 | 1.95E-21 | 4.22E-20 |
| ZNF467 | 2.728234 | 4.53578 | 0.733383 | 1.35E-19 | 2.42E-18 |
| PLVAP | 54.5256 | 94.17275 | 0.788376 | 1.20E-18 | 1.94E-17 |
| STOM | 39.67008 | 65.3873 | 0.720959 | 4.94E-18 | 7.49E-17 |
| RPL29P11 | 3.444671 | 2.291973 | -0.58778 | 1.01E-08 | 4.97E-08 |
| ADAMTS7 | 1.559566 | 2.65103 | 0.765408 | 3.92E-14 | 3.98E-13 |
| GRIK5 | 0.351815 | 1.382465 | 1.974352 | 3.46E-19 | 5.91E-18 |
| CHL1 | 0.465929 | 0.8484 | 0.864634 | 9.26E-17 | 1.23E-15 |
| CYSRT1 | 5.59685 | 1.65307 | -1.75947 | 0.007652 | 0.0131 |
| ECSCR | 2.182003 | 4.131669 | 0.921072 | 3.18E-22 | 7.66E-21 |
| FGA | 3.295945 | 5.389006 | 0.709327 | 0.003099 | 0.005761 |
| TMEM47 | 4.280625 | 12.55853 | 1.552774 | 9.19E-34 | 1.61E-31 |
| AC021074.1 | 3.141749 | 2.062706 | -0.60703 | 1.98E-10 | 1.27E-09 |
| FGF7 | 1.244681 | 5.440373 | 2.12793 | 1.45E-31 | 1.51E-29 |
| SHC2 | 2.358281 | 4.547731 | 0.947411 | 1.16E-18 | 1.88E-17 |
| EPHA3 | 0.911071 | 3.813201 | 2.065367 | 6.36E-29 | 3.80E-27 |
| CMTM3 | 6.737226 | 11.22236 | 0.73615 | 6.87E-18 | 1.02E-16 |
| DOCK4 | 1.318728 | 1.990016 | 0.593633 | 3.93E-16 | 4.92E-15 |
| IGHD3-22 | 1.438633 | 2.770983 | 0.945699 | 4.86E-06 | 1.52E-05 |
| CENPW | 18.59663 | 11.95134 | -0.63787 | 2.69E-12 | 2.22E-11 |
| TRAF3IP3 | 0.69112 | 1.172412 | 0.762471 | 5.60E-09 | 2.88E-08 |
| MIR222HG | 2.144974 | 1.247369 | -0.78207 | 5.53E-09 | 2.85E-08 |
| CELF3 | 0.474803 | 1.14711 | 1.272603 | 2.19E-05 | 6.10E-05 |
| PDGFRL | 1.06345 | 3.016436 | 1.504092 | 7.93E-19 | 1.31E-17 |
| MEIS3P1 | 2.143222 | 5.283562 | 1.30173 | 2.54E-28 | 1.32E-26 |
| FRMD4A | 1.210038 | 1.894877 | 0.647052 | 5.04E-20 | 9.61E-19 |
| SPSB1 | 10.84548 | 17.18932 | 0.664419 | 6.22E-19 | 1.04E-17 |
| GAREM2 | 1.233168 | 1.922732 | 0.640788 | 2.85E-13 | 2.66E-12 |
| IGDCC4 | 0.569832 | 1.504946 | 1.401104 | 1.18E-28 | 6.50E-27 |
| C3AR1 | 6.395657 | 9.817524 | 0.618267 | 3.05E-09 | 1.64E-08 |
| PLPP7 | 0.441625 | 1.418847 | 1.683824 | 6.32E-31 | 5.76E-29 |
| TNFSF11 | 0.816881 | 1.279231 | 0.647079 | 2.45E-08 | 1.13E-07 |
| BMP3 | 0.371125 | 1.585088 | 2.094586 | 1.99E-11 | 1.47E-10 |
| TMEM255A | 0.53518 | 1.083845 | 1.018062 | 2.00E-15 | 2.33E-14 |
| ZNF415 | 0.674086 | 1.349801 | 1.001742 | 4.46E-18 | 6.82E-17 |
| THEMIS2 | 4.18559 | 6.891119 | 0.719307 | 1.17E-12 | 1.00E-11 |
| ZNF549 | 0.39839 | 0.625292 | 0.65035 | 9.47E-11 | 6.38E-10 |
| MPO | 0.05896 | 2.166771 | 5.19966 | 2.67E-09 | 1.45E-08 |
| MS4A4A | 3.970473 | 6.292833 | 0.664399 | 1.28E-09 | 7.31E-09 |
| HIST1H3I | 0.982081 | 0.651387 | -0.59233 | 5.27E-06 | 1.63E-05 |
| NPY | 0.497722 | 1.140528 | 1.196289 | 1.15E-10 | 7.65E-10 |
| SLC22A17 | 1.118294 | 4.160278 | 1.89538 | 2.33E-39 | 3.04E-36 |
| IGHV4-61 | 9.800645 | 25.27172 | 1.366575 | 3.94E-09 | 2.08E-08 |
| LIX1L | 5.721898 | 11.77327 | 1.04095 | 1.71E-27 | 7.93E-26 |
| CLRN3 | 20.50791 | 13.64842 | -0.58745 | 0.000847 | 0.001772 |
| LRRC17 | 0.616036 | 1.730601 | 1.490187 | 1.07E-21 | 2.42E-20 |
| HAS3 | 4.253001 | 1.385951 | -1.6176 | 0.002751 | 0.005168 |
| RNA5SP92 | 0.38973 | 0.783187 | 1.00688 | 2.76E-06 | 9.02E-06 |
| RPL12P6 | 0.880982 | 0.578085 | -0.60783 | 3.31E-09 | 1.77E-08 |
| AC011899.2 | 0.394353 | 0.648779 | 0.718237 | 4.96E-11 | 3.49E-10 |
| AL034397.3 | 0.550566 | 0.996494 | 0.855947 | 2.03E-06 | 6.78E-06 |
| PVT1 | 4.52068 | 2.407556 | -0.90897 | 7.46E-11 | 5.10E-10 |
| MCAM | 12.11615 | 28.20429 | 1.218983 | 1.23E-24 | 3.85E-23 |
| TC2N | 21.3971 | 14.19671 | -0.59186 | 6.72E-08 | 2.87E-07 |
| GSTO2 | 3.366551 | 2.227257 | -0.596 | 9.84E-11 | 6.61E-10 |
| MSI1 | 2.371119 | 3.620559 | 0.610645 | 0.000298 | 0.000677 |
| ZNF71 | 0.741511 | 1.317755 | 0.829544 | 9.47E-18 | 1.39E-16 |
| CTSF | 5.219091 | 10.43088 | 0.998991 | 5.66E-25 | 1.85E-23 |
| IGHV3OR15-7 | 0.602128 | 1.946631 | 1.692838 | 4.02E-10 | 2.45E-09 |
| AKAP6 | 0.227625 | 0.788429 | 1.792324 | 1.17E-11 | 8.90E-11 |
| GVINP1 | 0.807761 | 1.559175 | 0.948783 | 6.69E-11 | 4.60E-10 |
| DTX3 | 2.787656 | 5.002182 | 0.843505 | 4.10E-20 | 7.87E-19 |
| CADM3 | 0.29177 | 1.424915 | 2.28797 | 2.65E-22 | 6.46E-21 |
| FMO3 | 1.278858 | 2.012933 | 0.654443 | 7.70E-12 | 5.98E-11 |
| SLAMF6 | 2.6113 | 3.974431 | 0.60598 | 7.20E-07 | 2.59E-06 |
| TRPC6 | 0.516549 | 0.898114 | 0.797992 | 4.13E-18 | 6.34E-17 |
| S1PR4 | 2.010954 | 3.021176 | 0.58723 | 1.48E-08 | 7.09E-08 |
| GREB1 | 0.235937 | 0.765189 | 1.697412 | 6.43E-29 | 3.83E-27 |
| ROBO1 | 2.642642 | 5.500029 | 1.057458 | 2.27E-25 | 7.87E-24 |
| PCSK1N | 12.8718 | 37.85725 | 1.556356 | 3.32E-15 | 3.76E-14 |
| HOXA4 | 1.085391 | 2.665925 | 1.296421 | 1.68E-17 | 2.40E-16 |
| CD1E | 0.337559 | 0.853091 | 1.33756 | 6.88E-15 | 7.59E-14 |
| CACNB2 | 0.458128 | 1.244727 | 1.442007 | 9.52E-20 | 1.75E-18 |
| IL1RN | 36.67338 | 16.89032 | -1.11854 | 0.009107 | 0.015343 |
| PRUNE2 | 2.058783 | 10.96057 | 2.412459 | 6.98E-16 | 8.48E-15 |
| CD163 | 4.430897 | 7.483843 | 0.756181 | 1.21E-08 | 5.88E-08 |
| ITLN1 | 23.21201 | 39.47988 | 0.766246 | 1.81E-10 | 1.17E-09 |
| PRRX1 | 2.940189 | 5.775039 | 0.973922 | 1.16E-12 | 9.99E-12 |
| GPSM1 | 1.829325 | 3.088681 | 0.75568 | 5.90E-17 | 7.99E-16 |
| BX470102.1 | 3.51691 | 2.2192 | -0.66427 | 1.76E-08 | 8.32E-08 |
| AC004540.1 | 0.470708 | 0.723495 | 0.620149 | 1.10E-14 | 1.19E-13 |
| DAAM2 | 1.532101 | 5.099151 | 1.734745 | 8.27E-32 | 8.87E-30 |
| CGNL1 | 0.989954 | 2.48924 | 1.330272 | 9.19E-24 | 2.59E-22 |
| CYYR1 | 2.687172 | 6.179136 | 1.201317 | 1.86E-36 | 7.69E-34 |
| HOTTIP | 1.781553 | 0.89605 | -0.99148 | 0.03057 | 0.045904 |
| AC007991.4 | 1.152896 | 0.323955 | -1.8314 | 0.004332 | 0.007817 |
| HTR1B | 0.708015 | 1.087035 | 0.618547 | 3.66E-10 | 2.25E-09 |
| DNAJC18 | 0.716903 | 1.455095 | 1.021264 | 7.64E-30 | 5.47E-28 |
| AKT3 | 1.883224 | 5.062957 | 1.426776 | 1.05E-30 | 9.17E-29 |
| BAHCC1 | 1.978162 | 3.05041 | 0.624843 | 5.59E-13 | 5.00E-12 |
| ALDH1B1 | 21.53198 | 35.64018 | 0.727024 | 0.000141 | 0.00034 |
| PDLIM4 | 4.532142 | 8.492671 | 0.906025 | 3.86E-18 | 5.95E-17 |
| CPNE8 | 1.287982 | 1.945268 | 0.594857 | 9.73E-16 | 1.16E-14 |
| NOTCH4 | 1.997906 | 3.574493 | 0.83925 | 1.31E-20 | 2.61E-19 |
| ITGB4 | 106.1241 | 67.9967 | -0.64222 | 3.16E-07 | 1.21E-06 |
| RCBTB2 | 2.358048 | 3.855617 | 0.709368 | 9.73E-23 | 2.47E-21 |
| SMYD1 | 0.257082 | 2.8074 | 3.448934 | 6.14E-05 | 0.000158 |
| GAD1 | 1.057046 | 0.376027 | -1.49113 | 1.13E-10 | 7.55E-10 |
| ADAMTS1 | 3.848418 | 11.4869 | 1.577653 | 5.33E-31 | 5.03E-29 |
| HMCN1 | 0.521628 | 2.032281 | 1.962005 | 1.06E-32 | 1.43E-30 |
| CLMP | 2.140904 | 8.086118 | 1.917227 | 3.15E-28 | 1.62E-26 |
| FDCSP | 19.70474 | 34.23732 | 0.797027 | 9.96E-09 | 4.92E-08 |
| LINC00920 | 1.40841 | 0.780575 | -0.85146 | 3.93E-05 | 0.000105 |
| IL16 | 0.916947 | 1.962331 | 1.097658 | 1.35E-16 | 1.76E-15 |
| ARHGAP31 | 2.321609 | 5.111024 | 1.138487 | 2.25E-33 | 3.56E-31 |
| SORBS1 | 5.49703 | 24.59398 | 2.161581 | 2.93E-19 | 5.06E-18 |
| BTG2 | 44.27064 | 68.01741 | 0.619554 | 1.02E-08 | 5.02E-08 |
| LINC02474 | 2.107999 | 0.872 | -1.27347 | 0.004893 | 0.008733 |
| EIF4E3 | 2.147606 | 3.277272 | 0.609766 | 2.72E-12 | 2.25E-11 |
| PCDHGC3 | 2.289762 | 4.916222 | 1.102352 | 1.90E-23 | 5.20E-22 |
| LONRF2 | 0.30707 | 0.738107 | 1.265264 | 8.61E-13 | 7.53E-12 |
| IGLV6-57 | 44.36696 | 110.9655 | 1.322554 | 1.79E-07 | 7.11E-07 |
| ADAMTSL1 | 0.271391 | 0.834624 | 1.620752 | 1.21E-36 | 5.54E-34 |
| NRXN3 | 0.334963 | 1.579409 | 2.23731 | 1.05E-21 | 2.38E-20 |
| CDON | 0.602791 | 1.672998 | 1.472706 | 1.88E-15 | 2.20E-14 |
| GSC | 0.40791 | 0.959355 | 1.233813 | 3.56E-11 | 2.54E-10 |
| HS3ST2 | 0.919453 | 1.62868 | 0.824856 | 5.60E-07 | 2.05E-06 |
| BEX2 | 1.956416 | 3.718214 | 0.926396 | 3.81E-16 | 4.77E-15 |
| RNF216-IT1 | 0.592448 | 1.003452 | 0.760211 | 1.12E-06 | 3.90E-06 |
| CCDC80 | 4.64192 | 20.13452 | 2.116878 | 2.47E-27 | 1.10E-25 |
| AC024075.3 | 1.16618 | 1.756057 | 0.590549 | 2.12E-08 | 9.88E-08 |
| WWTR1 | 7.825734 | 16.31059 | 1.059511 | 6.04E-22 | 1.41E-20 |
| VCL | 22.89024 | 36.61291 | 0.67762 | 1.98E-11 | 1.46E-10 |
| NXN | 4.648553 | 7.944066 | 0.773096 | 1.78E-13 | 1.70E-12 |
| SERPINB5 | 41.31746 | 19.33415 | -1.0956 | 3.37E-09 | 1.80E-08 |
| CD37 | 7.878139 | 13.60533 | 0.788245 | 1.31E-10 | 8.64E-10 |
| FYN | 4.321549 | 6.949901 | 0.685444 | 1.97E-21 | 4.25E-20 |
| MGST1 | 19.90082 | 11.2656 | -0.8209 | 5.50E-10 | 3.29E-09 |
| IGKV2D-40 | 3.349461 | 13.62929 | 2.02471 | 3.62E-07 | 1.37E-06 |
| ZNF439 | 0.5809 | 0.903734 | 0.637608 | 2.35E-11 | 1.72E-10 |
| FLI1 | 1.460952 | 2.819031 | 0.94829 | 3.78E-25 | 1.27E-23 |
| TGFBR2 | 30.48962 | 48.02196 | 0.655376 | 7.23E-18 | 1.07E-16 |
| TNS2 | 5.515229 | 14.23221 | 1.367667 | 5.84E-39 | 5.83E-36 |
| AL139421.1 | 1.620531 | 1.045103 | -0.63282 | 0.000262 | 0.000602 |
| RTL8B | 3.591024 | 6.889434 | 0.93999 | 2.75E-24 | 8.22E-23 |
| PTGFR | 0.342593 | 1.184337 | 1.789511 | 6.46E-26 | 2.49E-24 |
| LPP | 12.10725 | 27.49325 | 1.183207 | 2.15E-15 | 2.49E-14 |
| CXCL1 | 77.3834 | 48.83902 | -0.66399 | 0.00037 | 0.000826 |
| S1PR3 | 2.366634 | 5.663911 | 1.258962 | 4.05E-25 | 1.35E-23 |
| LGI2 | 0.889296 | 2.968403 | 1.738952 | 1.96E-30 | 1.60E-28 |
| DKK2 | 0.551754 | 1.38912 | 1.332073 | 1.57E-15 | 1.85E-14 |
| AL590004.3 | 0.373441 | 0.75433 | 1.014317 | 4.32E-05 | 0.000115 |
| COL9A1 | 0.553818 | 1.22607 | 1.146557 | 2.90E-11 | 2.10E-10 |
| NR1H4 | 0.377219 | 0.883817 | 1.228347 | 3.75E-07 | 1.42E-06 |
| MAFB | 10.18722 | 16.32923 | 0.680697 | 1.50E-11 | 1.12E-10 |
| GJB2 | 36.06759 | 18.54656 | -0.95955 | 0.000163 | 0.000387 |
| SPRR2D | 20.42753 | 0.672332 | -4.9252 | 0.001575 | 0.003113 |
| CCL2 | 13.04356 | 23.56582 | 0.853358 | 2.79E-13 | 2.60E-12 |
| RFLNB | 3.751011 | 6.66258 | 0.828801 | 3.49E-24 | 1.03E-22 |
| HOXA10-AS | 0.804387 | 0.51702 | -0.63767 | 0.002729 | 0.005133 |
| MEIS3P2 | 0.304886 | 0.882149 | 1.532751 | 1.16E-26 | 4.77E-25 |
| MSN | 42.26946 | 66.1458 | 0.646034 | 1.03E-20 | 2.08E-19 |
| PSME2 | 50.63911 | 31.86457 | -0.6683 | 1.07E-12 | 9.30E-12 |
| DMPK | 4.407262 | 9.878301 | 1.16438 | 7.38E-10 | 4.33E-09 |
| GSTT2B | 0.872761 | 1.355284 | 0.634936 | 0.000117 | 0.000285 |
| CCL21 | 33.77551 | 61.77565 | 0.871061 | 4.13E-16 | 5.15E-15 |
| BTK | 1.248505 | 2.009478 | 0.686619 | 2.42E-12 | 2.01E-11 |
| DCHS1 | 2.57056 | 6.893081 | 1.423066 | 1.75E-37 | 1.19E-34 |
| FBLN5 | 4.796527 | 15.68943 | 1.709731 | 8.04E-41 | 3.11E-37 |
| HMGB1P6 | 11.0866 | 6.66739 | -0.73362 | 2.94E-07 | 1.13E-06 |
| DOK5 | 0.528103 | 1.23788 | 1.228981 | 1.90E-27 | 8.69E-26 |
| MAGEE1 | 0.411353 | 0.951529 | 1.209871 | 2.00E-21 | 4.33E-20 |
| AMOTL1 | 3.65408 | 8.226953 | 1.17085 | 4.77E-23 | 1.24E-21 |
| RNF152 | 1.114606 | 1.698259 | 0.607522 | 2.22E-15 | 2.56E-14 |
| WNT5A-AS1 | 1.372442 | 2.738467 | 0.996623 | 2.67E-14 | 2.77E-13 |
| HSPE1 | 70.48192 | 44.78265 | -0.65431 | 1.10E-16 | 1.45E-15 |
| TMEM45B | 47.72093 | 31.78082 | -0.58647 | 2.51E-10 | 1.58E-09 |
| FADS1 | 2.083764 | 3.647207 | 0.8076 | 9.65E-16 | 1.16E-14 |
| IGHV3-22 | 0.388435 | 0.918966 | 1.242337 | 3.76E-07 | 1.42E-06 |
| IGFBP4 | 129.6936 | 317.4851 | 1.291581 | 6.32E-25 | 2.05E-23 |
| FPR1 | 2.381756 | 4.454531 | 0.903248 | 5.58E-06 | 1.72E-05 |
| CEP112 | 0.510507 | 0.854627 | 0.743364 | 5.05E-21 | 1.04E-19 |
| CERCAM | 6.061137 | 10.95167 | 0.853491 | 8.51E-16 | 1.02E-14 |
| FHL1 | 6.094386 | 34.70477 | 2.509581 | 7.17E-29 | 4.24E-27 |
| RASSF2 | 3.752685 | 8.038646 | 1.099029 | 1.56E-29 | 1.06E-27 |
| ANK2 | 0.351753 | 1.620979 | 2.204233 | 1.05E-30 | 9.17E-29 |
| MIR4539 | 0.491424 | 0.821818 | 0.74185 | 3.66E-05 | 9.83E-05 |
| FOLR1 | 5.659319 | 10.12138 | 0.838706 | 6.22E-05 | 0.00016 |
| FCRL3 | 0.535883 | 1.215697 | 1.181793 | 4.18E-07 | 1.56E-06 |
| PRR7 | 11.14957 | 7.419941 | -0.58751 | 2.35E-07 | 9.22E-07 |
| VWA5B2 | 0.391813 | 1.90625 | 2.282499 | 0.005479 | 0.009669 |
| NKX2-2 | 0.295502 | 1.533573 | 2.375658 | 3.53E-05 | 9.52E-05 |
| BMPR1B | 0.509917 | 0.814866 | 0.676299 | 8.06E-11 | 5.49E-10 |
| MITF | 1.747972 | 2.878462 | 0.719616 | 9.05E-14 | 8.90E-13 |
| IGHV5-51 | 125.0629 | 233.9339 | 0.903448 | 5.92E-10 | 3.52E-09 |
| SEC61G | 60.86485 | 36.82063 | -0.7251 | 6.36E-09 | 3.24E-08 |
| TTR | 2.409853 | 8.017436 | 1.734196 | 3.13E-09 | 1.68E-08 |
| IGLVI-70 | 1.189679 | 2.168203 | 0.865928 | 8.63E-07 | 3.07E-06 |
| IGKV3-20 | 357.9715 | 640.4448 | 0.839229 | 2.98E-07 | 1.15E-06 |
| IL3RA | 3.839701 | 6.333663 | 0.722046 | 5.00E-19 | 8.40E-18 |
| GLI1 | 0.614664 | 2.083171 | 1.760912 | 2.09E-34 | 4.16E-32 |
| EFNB3 | 0.367779 | 0.962775 | 1.38836 | 1.04E-16 | 1.37E-15 |
| SHROOM4 | 1.301855 | 2.363485 | 0.860347 | 7.65E-23 | 1.95E-21 |
| NTM | 0.799402 | 1.552441 | 0.957545 | 1.43E-10 | 9.39E-10 |
| TTYH2 | 1.177168 | 2.452694 | 1.059047 | 5.06E-25 | 1.66E-23 |
| AL032819.2 | 0.202642 | 0.955779 | 2.237745 | 2.76E-10 | 1.73E-09 |
| CD4 | 10.34356 | 15.55984 | 0.589095 | 6.90E-10 | 4.06E-09 |
| SH3BP5 | 1.208802 | 2.435091 | 1.010398 | 3.05E-23 | 8.14E-22 |
| PRR29 | 0.290933 | 0.827694 | 1.508411 | 3.28E-19 | 5.64E-18 |
| TXN | 278.5029 | 168.3794 | -0.72598 | 7.66E-19 | 1.27E-17 |
| CYP2F2P | 0.467838 | 0.940179 | 1.006927 | 2.96E-09 | 1.59E-08 |
| IGHV1-18 | 137.9563 | 253.0263 | 0.875076 | 1.11E-06 | 3.88E-06 |
| CEACAM1 | 24.15929 | 15.20501 | -0.66803 | 2.58E-05 | 7.11E-05 |
| SIM2 | 5.248905 | 3.42089 | -0.61764 | 4.42E-08 | 1.94E-07 |
| SCUBE2 | 0.356344 | 1.235413 | 1.79365 | 2.56E-32 | 3.04E-30 |
| FCGR2C | 0.483405 | 0.904326 | 0.90361 | 1.83E-09 | 1.02E-08 |
| NRIP2 | 1.166776 | 2.351798 | 1.011237 | 5.58E-29 | 3.39E-27 |
| DKK3 | 10.51848 | 22.17565 | 1.07605 | 4.25E-29 | 2.64E-27 |
| CALHM2 | 3.511652 | 6.082422 | 0.792496 | 5.14E-24 | 1.49E-22 |
| AC008808.2 | 0.331513 | 1.28049 | 1.949559 | 1.28E-24 | 4.00E-23 |
| TMEM79 | 3.568754 | 2.259599 | -0.65935 | 0.00316 | 0.005861 |
| TCEAL2 | 1.048965 | 3.380112 | 1.688104 | 2.05E-16 | 2.65E-15 |
| WWC2 | 1.609158 | 2.446262 | 0.604273 | 6.13E-13 | 5.46E-12 |
| BASP1 | 10.35501 | 17.64902 | 0.76926 | 4.96E-16 | 6.14E-15 |
| C5orf46 | 0.640613 | 0.365084 | -0.81123 | 0.014487 | 0.023382 |
| HRASLS5 | 1.010342 | 1.608524 | 0.670894 | 1.02E-10 | 6.80E-10 |
| TRAV41 | 0.503047 | 0.763888 | 0.602668 | 3.06E-05 | 8.33E-05 |
| ADAMTS15 | 0.851401 | 1.439271 | 0.757428 | 2.61E-08 | 1.19E-07 |
| ACKR1 | 3.523708 | 14.97189 | 2.08709 | 6.90E-28 | 3.36E-26 |
| CLEC14A | 5.32919 | 11.60984 | 1.12336 | 1.35E-32 | 1.78E-30 |
| CARMN | 0.361434 | 1.807742 | 2.322387 | 6.27E-22 | 1.46E-20 |
| IGKV3OR2-268 | 2.621475 | 5.318336 | 1.020596 | 2.07E-07 | 8.17E-07 |
| ICA1L | 0.423139 | 0.642484 | 0.602527 | 1.60E-09 | 8.99E-09 |
| KCTD14 | 6.597064 | 3.975794 | -0.73058 | 5.04E-05 | 0.000132 |
| IGKV1OR2-108 | 3.484027 | 10.08463 | 1.53333 | 4.62E-11 | 3.25E-10 |
| RNU6-531P | 2.12309 | 3.20993 | 0.596377 | 4.70E-08 | 2.05E-07 |
| GPX3 | 14.20674 | 28.00231 | 0.978971 | 1.13E-21 | 2.54E-20 |
| AC022034.1 | 0.982484 | 1.801186 | 0.874442 | 2.85E-13 | 2.66E-12 |
| TRBJ2-3 | 2.454173 | 3.929287 | 0.679031 | 0.001372 | 0.002747 |
| TLR10 | 0.698785 | 1.528203 | 1.128914 | 6.86E-10 | 4.04E-09 |
| NRROS | 1.631198 | 2.83587 | 0.797859 | 5.85E-19 | 9.79E-18 |
| PINLYP | 0.404025 | 0.706474 | 0.806193 | 9.18E-17 | 1.23E-15 |
| LINC00987 | 0.361621 | 0.646406 | 0.837963 | 1.42E-19 | 2.55E-18 |
| PI16 | 0.70439 | 4.975721 | 2.82046 | 3.07E-21 | 6.49E-20 |
| IL34 | 1.588632 | 3.309198 | 1.058697 | 1.38E-21 | 3.07E-20 |
| PTGS1 | 3.419565 | 6.649989 | 0.959539 | 1.04E-17 | 1.52E-16 |
| A4GNT | 2.186546 | 5.231807 | 1.258656 | 2.00E-09 | 1.11E-08 |
| TLR7 | 0.672722 | 1.420069 | 1.077878 | 3.89E-14 | 3.96E-13 |
| CENPH | 4.793713 | 3.135951 | -0.61224 | 2.90E-12 | 2.38E-11 |
| SFRP2 | 39.19907 | 123.7851 | 1.658947 | 1.10E-13 | 1.07E-12 |
| ALKAL1 | 0.917274 | 2.156525 | 1.233284 | 9.16E-15 | 9.98E-14 |
| FOXP2 | 0.284162 | 1.091892 | 1.942045 | 8.45E-21 | 1.72E-19 |
| KLF7 | 2.671857 | 4.735385 | 0.825639 | 1.41E-21 | 3.11E-20 |
| LRRC10B | 0.52504 | 1.253261 | 1.255187 | 5.06E-11 | 3.55E-10 |
| C17orf107 | 0.542838 | 0.887781 | 0.709682 | 1.35E-14 | 1.45E-13 |
| PCDHB15 | 0.596609 | 1.000537 | 0.745917 | 1.37E-21 | 3.04E-20 |
| RASGRF2 | 0.741702 | 1.365272 | 0.880276 | 2.44E-24 | 7.33E-23 |
| TACC1 | 9.990665 | 16.57781 | 0.730601 | 1.70E-15 | 1.99E-14 |
| TRBV7-9 | 1.796421 | 2.979494 | 0.729942 | 1.20E-06 | 4.16E-06 |
| RARRES2 | 16.30683 | 31.77161 | 0.962262 | 6.38E-19 | 1.06E-17 |
| SIX2 | 2.52314 | 7.756566 | 1.620198 | 1.55E-15 | 1.82E-14 |
| AC053513.1 | 0.447739 | 0.70959 | 0.664327 | 3.57E-07 | 1.35E-06 |
| NDNF | 1.260569 | 2.526062 | 1.002815 | 2.80E-21 | 5.94E-20 |
| IGLV4-3 | 0.43452 | 2.005459 | 2.206437 | 6.49E-09 | 3.30E-08 |
| IGHD | 7.523993 | 14.24897 | 0.921287 | 4.57E-10 | 2.77E-09 |
| ETS1 | 12.24873 | 18.78031 | 0.616589 | 2.77E-13 | 2.59E-12 |
| NMUR1 | 0.357628 | 0.884228 | 1.30596 | 2.22E-18 | 3.49E-17 |
| AP1S1 | 39.38599 | 25.7157 | -0.61503 | 8.33E-11 | 5.66E-10 |
| ADH4 | 1.488202 | 5.944947 | 1.998094 | 5.95E-08 | 2.56E-07 |
| FYCO1 | 6.101074 | 10.62325 | 0.80009 | 2.32E-13 | 2.18E-12 |
| EDA | 0.671131 | 1.245795 | 0.8924 | 1.37E-07 | 5.55E-07 |
| TSPAN2 | 2.962014 | 6.337081 | 1.09724 | 4.57E-21 | 9.51E-20 |
| AC091959.1 | 0.710822 | 0.463609 | -0.61658 | 0.001653 | 0.003252 |
| ITGA7 | 2.296435 | 8.128431 | 1.823581 | 4.62E-25 | 1.53E-23 |
| CRYBG1 | 18.34394 | 8.938082 | -1.03727 | 1.26E-06 | 4.35E-06 |
| POC1A | 6.485735 | 4.313403 | -0.58844 | 6.77E-13 | 5.98E-12 |
| ELN | 5.430401 | 21.07326 | 1.956283 | 6.68E-37 | 3.68E-34 |
| TAC3 | 0.702895 | 1.6499 | 1.230997 | 8.29E-10 | 4.83E-09 |
| EDNRB | 2.170704 | 5.099169 | 1.232099 | 2.60E-30 | 2.05E-28 |
| FRMD6 | 2.028251 | 3.504447 | 0.788951 | 3.60E-17 | 5.00E-16 |
| FHL5 | 0.587184 | 1.660794 | 1.499989 | 2.12E-26 | 8.45E-25 |
| AC005180.1 | 0.189595 | 0.833492 | 2.136248 | 2.05E-18 | 3.25E-17 |
| NEURL1B | 4.841946 | 8.489761 | 0.810137 | 4.35E-15 | 4.89E-14 |
| ISM1 | 1.131245 | 3.456979 | 1.6116 | 4.46E-19 | 7.55E-18 |
| ITGB3 | 0.643222 | 1.559299 | 1.277509 | 3.38E-25 | 1.14E-23 |
| KLHL29 | 0.787435 | 1.430707 | 0.861496 | 1.20E-19 | 2.18E-18 |
| FNBP1 | 6.192507 | 15.1786 | 1.293443 | 2.54E-23 | 6.84E-22 |
| IRX2 | 7.839882 | 5.076154 | -0.6271 | 0.008508 | 0.014424 |
| LINC01589 | 0.552638 | 1.173023 | 1.085824 | 2.02E-14 | 2.13E-13 |
| PRIMA1 | 0.637805 | 3.813317 | 2.579859 | 3.71E-23 | 9.72E-22 |
| AC007342.5 | 1.490705 | 0.889342 | -0.74518 | 7.32E-08 | 3.11E-07 |
| SPOCK2 | 7.028589 | 14.19163 | 1.013733 | 1.88E-10 | 1.21E-09 |
| EFHD1 | 2.032462 | 3.41744 | 0.749688 | 2.12E-25 | 7.42E-24 |
| CALCRL | 3.833299 | 7.185226 | 0.906447 | 1.51E-24 | 4.67E-23 |
| ACTA2 | 59.91275 | 223.7194 | 1.900755 | 6.07E-25 | 1.97E-23 |
| PDIA2 | 3.956981 | 6.794641 | 0.779997 | 0.000151 | 0.000361 |
| SFRP1 | 0.964174 | 7.715961 | 3.000481 | 4.89E-26 | 1.90E-24 |
| GAP43 | 0.33301 | 0.836026 | 1.327984 | 1.00E-18 | 1.64E-17 |
| IGHV3OR16-11 | 0.455038 | 1.402967 | 1.624421 | 5.97E-08 | 2.57E-07 |
| CEP72 | 3.832412 | 2.471683 | -0.63276 | 3.91E-07 | 1.47E-06 |
| IGHV1OR16-3 | 0.263334 | 0.756095 | 1.521672 | 8.96E-05 | 0.000224 |
| CREB3L3 | 2.517626 | 6.302106 | 1.32377 | 3.71E-08 | 1.65E-07 |
| ZBTB20 | 1.318214 | 2.345649 | 0.831402 | 1.55E-13 | 1.49E-12 |
| PEAK1 | 2.618314 | 4.069217 | 0.636113 | 9.43E-20 | 1.74E-18 |
| AC006483.1 | 3.504849 | 2.195498 | -0.6748 | 4.29E-06 | 1.36E-05 |
| FBXO32 | 6.707945 | 16.09187 | 1.262389 | 1.99E-11 | 1.47E-10 |
| RNF180 | 0.360494 | 1.063984 | 1.561431 | 1.08E-31 | 1.13E-29 |
| ABCA1 | 3.551276 | 5.968025 | 0.748916 | 3.07E-18 | 4.77E-17 |
| IL10RA | 3.628347 | 5.799741 | 0.676676 | 7.26E-14 | 7.19E-13 |
| AC011472.4 | 0.474492 | 0.821068 | 0.791118 | 1.57E-06 | 5.36E-06 |
| HES6 | 10.91766 | 6.424107 | -0.7651 | 0.000241 | 0.000556 |
| TNN | 0.196031 | 1.099585 | 2.487806 | 2.54E-29 | 1.63E-27 |
| RNU1-106P | 2.086288 | 1.354515 | -0.62316 | 6.30E-06 | 1.93E-05 |
| HACD4 | 0.86979 | 2.127616 | 1.290499 | 5.15E-34 | 9.29E-32 |
| GLI3 | 0.677633 | 2.035673 | 1.58693 | 5.45E-28 | 2.70E-26 |
| RELN | 0.107319 | 0.98432 | 3.197224 | 1.19E-26 | 4.91E-25 |
| AL031587.5 | 0.617786 | 1.003693 | 0.700139 | 1.19E-07 | 4.89E-07 |
| SSC5D | 1.77608 | 7.140168 | 2.007261 | 2.23E-28 | 1.18E-26 |
| IGFBP7 | 164.4158 | 365.5965 | 1.152903 | 9.20E-29 | 5.24E-27 |
| LINC01139 | 0.318047 | 0.707959 | 1.154428 | 0.000438 | 0.000966 |
| AASS | 0.811331 | 1.310241 | 0.691471 | 1.14E-14 | 1.24E-13 |
| COL6A3 | 24.77444 | 57.38193 | 1.211744 | 5.91E-21 | 1.21E-19 |
| ACVR1 | 8.723654 | 14.14045 | 0.696824 | 8.05E-15 | 8.81E-14 |
| RASL10B | 0.7763 | 1.185152 | 0.610385 | 2.23E-08 | 1.04E-07 |
| AC104825.1 | 0.720716 | 1.283537 | 0.832622 | 7.23E-15 | 7.95E-14 |
| LBH | 9.810849 | 18.66886 | 0.928184 | 1.06E-25 | 3.90E-24 |
| CPED1 | 1.370146 | 6.609782 | 2.270273 | 1.01E-34 | 2.25E-32 |
| SNORD114-3 | 0.377783 | 0.696435 | 0.882432 | 0.000105 | 0.000259 |
| AC005077.4 | 2.292145 | 0.828285 | -1.4685 | 0.002191 | 0.004207 |
| PNCK | 0.354527 | 1.574884 | 2.151278 | 3.16E-09 | 1.69E-08 |
| POF1B | 31.84626 | 20.96465 | -0.60316 | 9.08E-08 | 3.79E-07 |
| CC2D2A | 1.057944 | 1.982937 | 0.906376 | 8.63E-20 | 1.60E-18 |
| PGC | 460.3481 | 1609.989 | 1.806254 | 3.23E-08 | 1.45E-07 |
| LAYN | 1.351444 | 3.638681 | 1.428913 | 3.78E-25 | 1.27E-23 |
| SLIRP | 19.46298 | 12.86242 | -0.59757 | 7.80E-13 | 6.86E-12 |
| AP000697.1 | 1.308755 | 0.870232 | -0.58872 | 2.10E-05 | 5.87E-05 |
| GAS1 | 3.1346 | 8.543731 | 1.446585 | 7.68E-16 | 9.31E-15 |
| SOX8 | 0.825743 | 2.226816 | 1.431218 | 4.35E-07 | 1.62E-06 |
| TAGLN | 41.10168 | 189.1849 | 2.202528 | 1.16E-22 | 2.90E-21 |
| CHST3 | 2.553122 | 4.528003 | 0.826613 | 4.87E-19 | 8.19E-18 |
| LARP6 | 1.058838 | 1.959242 | 0.887814 | 3.60E-24 | 1.06E-22 |
| WDR86 | 0.406047 | 0.658553 | 0.697652 | 1.47E-14 | 1.57E-13 |
| CDO1 | 0.375306 | 1.171644 | 1.642397 | 2.32E-27 | 1.03E-25 |
| HSD11B1 | 1.766826 | 3.388118 | 0.939324 | 8.62E-15 | 9.42E-14 |
| PID1 | 2.450226 | 5.193533 | 1.083801 | 3.42E-19 | 5.85E-18 |
| CD28 | 0.695403 | 1.378928 | 0.987627 | 2.09E-14 | 2.20E-13 |
| PDK4 | 8.220653 | 28.33598 | 1.78531 | 7.75E-20 | 1.45E-18 |
| CD40LG | 0.91696 | 1.473117 | 0.68394 | 2.40E-08 | 1.10E-07 |
| GADD45B | 12.16513 | 18.56901 | 0.610146 | 1.81E-12 | 1.52E-11 |
| ZNF880 | 1.014131 | 2.198188 | 1.116071 | 4.42E-23 | 1.15E-21 |
| CPNE5 | 2.301295 | 3.457144 | 0.587135 | 1.19E-11 | 9.07E-11 |
| FAM13B | 3.255784 | 4.914084 | 0.593918 | 1.80E-13 | 1.71E-12 |
| RAP2C-AS1 | 0.415965 | 0.778783 | 0.904759 | 4.33E-20 | 8.29E-19 |
| KCNMB1 | 1.570082 | 6.794393 | 2.113505 | 1.56E-18 | 2.50E-17 |
| PBK | 15.24932 | 8.053286 | -0.9211 | 2.38E-14 | 2.49E-13 |
| PLEKHG2 | 4.58065 | 6.908753 | 0.592873 | 1.78E-17 | 2.54E-16 |
| FTLP14 | 4.90959 | 3.122995 | -0.65267 | 1.70E-06 | 5.78E-06 |
| TGFB2-AS1 | 0.874457 | 1.524963 | 0.802314 | 1.15E-11 | 8.79E-11 |
| AC100830.2 | 0.536587 | 0.862443 | 0.684617 | 9.63E-07 | 3.40E-06 |
| TRIM7 | 1.462633 | 0.801588 | -0.86763 | 0.000853 | 0.001783 |
| PCSK1 | 0.524283 | 2.487206 | 2.246107 | 4.76E-14 | 4.79E-13 |
| INKA2 | 0.488764 | 0.868884 | 0.830026 | 1.66E-12 | 1.40E-11 |
| AL513008.1 | 1.131812 | 2.107286 | 0.896751 | 8.50E-06 | 2.55E-05 |
| EMCN | 1.721114 | 3.823766 | 1.151652 | 3.71E-28 | 1.89E-26 |
| IGLV2-11 | 88.62889 | 167.5356 | 0.918618 | 1.32E-07 | 5.37E-07 |
| PTCH2 | 0.607092 | 2.025107 | 1.738011 | 2.20E-27 | 9.84E-26 |
| CNTN1 | 0.721292 | 1.707046 | 1.242847 | 2.37E-19 | 4.15E-18 |
| RASGRP4 | 0.459991 | 0.916131 | 0.993949 | 2.21E-19 | 3.88E-18 |
| AC046185.3 | 0.679745 | 1.086356 | 0.676432 | 6.45E-16 | 7.86E-15 |
| AC007537.1 | 9.655666 | 5.563133 | -0.79548 | 4.13E-14 | 4.18E-13 |
| AC245297.1 | 0.784218 | 1.660624 | 1.082398 | 4.09E-12 | 3.30E-11 |
| CD6 | 1.871292 | 2.902066 | 0.633046 | 1.00E-05 | 2.96E-05 |
| SFMBT2 | 0.890689 | 1.522666 | 0.773606 | 5.61E-15 | 6.26E-14 |
| HSPE1P2 | 2.443568 | 1.50377 | -0.70041 | 4.03E-11 | 2.85E-10 |
| SCGN | 1.415865 | 3.682846 | 1.379138 | 1.20E-10 | 7.93E-10 |
| FUT9 | 0.46359 | 0.896814 | 0.95196 | 0.002235 | 0.004288 |
| CDCA7 | 23.69778 | 13.19539 | -0.84472 | 5.13E-11 | 3.59E-10 |
| HTR7P1 | 0.374851 | 0.626027 | 0.739908 | 1.91E-15 | 2.23E-14 |
| TMEM170B | 0.589059 | 1.050326 | 0.834353 | 9.16E-18 | 1.35E-16 |
| SIX1 | 1.019719 | 0.533646 | -0.93422 | 0.02416 | 0.037093 |
| GPRASP2 | 0.841999 | 1.643724 | 0.965078 | 3.33E-22 | 7.98E-21 |
| NCR3 | 0.485014 | 0.759483 | 0.646992 | 0.000128 | 0.000311 |
| ERO1A | 32.62547 | 18.64626 | -0.80711 | 5.04E-20 | 9.61E-19 |
| CCR10 | 0.507314 | 0.870376 | 0.77876 | 1.52E-16 | 1.99E-15 |
| ACOX2 | 1.317487 | 2.043923 | 0.633552 | 3.18E-13 | 2.95E-12 |
| KREMEN2 | 1.206645 | 0.660687 | -0.86896 | 9.59E-05 | 0.000238 |
| ANPEP | 78.55865 | 145.7823 | 0.891974 | 3.66E-09 | 1.94E-08 |
| C1QTNF3 | 1.091719 | 3.290084 | 1.591523 | 7.25E-17 | 9.74E-16 |
| MFGE8 | 12.54344 | 21.15018 | 0.753736 | 3.46E-19 | 5.91E-18 |
| KLHL13 | 0.635932 | 1.10481 | 0.796854 | 9.88E-14 | 9.68E-13 |
| INHBA | 7.127342 | 11.12717 | 0.642651 | 9.45E-07 | 3.34E-06 |
| FCRL1 | 0.641989 | 1.253981 | 0.965896 | 4.68E-10 | 2.83E-09 |
| ARMCX4 | 0.386778 | 0.696941 | 0.84953 | 4.11E-17 | 5.68E-16 |
| E2F7 | 2.411228 | 1.410145 | -0.77392 | 6.31E-11 | 4.36E-10 |
| CREBRF | 2.146916 | 3.246877 | 0.596787 | 3.27E-16 | 4.13E-15 |
| NOX4 | 0.473999 | 0.769513 | 0.69906 | 1.91E-09 | 1.06E-08 |
| ARX | 0.446016 | 0.677609 | 0.603357 | 0.001531 | 0.003033 |
| NAT8B | 1.310786 | 2.159864 | 0.720508 | 0.000619 | 0.001329 |
| TRIL | 1.176288 | 2.848053 | 1.275735 | 6.37E-27 | 2.74E-25 |
| CDK5R2 | 0.743061 | 1.442482 | 0.957 | 6.16E-06 | 1.89E-05 |
| CYP27A1 | 8.146775 | 13.59865 | 0.739163 | 1.51E-12 | 1.28E-11 |
| FAM229B | 1.00615 | 1.951323 | 0.955608 | 5.67E-20 | 1.07E-18 |
| CLSTN3 | 5.680057 | 9.974336 | 0.812315 | 5.29E-18 | 7.99E-17 |
| LINGO3 | 0.486436 | 1.298923 | 1.416995 | 9.96E-18 | 1.46E-16 |
| SYNPO | 9.246567 | 22.35984 | 1.27392 | 1.68E-31 | 1.71E-29 |
| AC007969.1 | 60.90597 | 39.3461 | -0.63036 | 1.10E-09 | 6.34E-09 |
| KRT78 | 4.099694 | 0.053923 | -6.24847 | 0.033433 | 0.049673 |
| TMEM204 | 5.62808 | 11.05009 | 0.973343 | 1.67E-29 | 1.13E-27 |
| F2RL3 | 2.209772 | 5.256613 | 1.250236 | 5.04E-19 | 8.47E-18 |
| AHNAK2 | 4.174073 | 7.276418 | 0.801773 | 2.99E-08 | 1.36E-07 |
| ITGAM | 1.572293 | 3.258235 | 1.05122 | 8.80E-13 | 7.68E-12 |
| STMN2 | 0.401147 | 1.70691 | 2.089185 | 2.81E-22 | 6.80E-21 |
| MADCAM1 | 0.72648 | 2.072161 | 1.512141 | 8.47E-08 | 3.56E-07 |
| ACTN1 | 29.21589 | 49.10154 | 0.749015 | 2.26E-08 | 1.05E-07 |
| CDC37L1-DT | 1.403972 | 0.702072 | -0.99982 | 0.0013 | 0.002611 |
| PKIG | 9.326492 | 18.24767 | 0.968306 | 2.54E-25 | 8.77E-24 |
| SDC3 | 11.80995 | 20.93172 | 0.825688 | 2.91E-18 | 4.56E-17 |
| AC005006.1 | 0.405357 | 0.744387 | 0.876861 | 7.65E-06 | 2.31E-05 |
| SLC18A1 | 0.182186 | 1.023908 | 2.490601 | 0.00117 | 0.002372 |
| DGUOK-AS1 | 2.472888 | 1.631351 | -0.60013 | 8.19E-06 | 2.46E-05 |
| ADGRL4 | 4.782179 | 8.499401 | 0.829693 | 5.35E-22 | 1.26E-20 |
| EPHA2 | 44.07436 | 28.4183 | -0.63312 | 2.06E-08 | 9.62E-08 |
| CSGALNACT2 | 6.332572 | 10.31859 | 0.704383 | 5.10E-23 | 1.32E-21 |
| ACKR3 | 6.545595 | 11.83675 | 0.854677 | 3.46E-20 | 6.70E-19 |
| SELPLG | 8.029017 | 12.20981 | 0.604745 | 3.49E-09 | 1.86E-08 |
| CD302 | 1.807265 | 3.985205 | 1.140846 | 2.28E-24 | 6.85E-23 |
| IGLC6 | 1.500662 | 2.730154 | 0.863383 | 2.11E-05 | 5.89E-05 |
| CLEC11A | 5.862757 | 11.9895 | 1.03212 | 4.62E-19 | 7.80E-18 |
| HMGA1P4 | 2.161942 | 1.424772 | -0.6016 | 7.27E-05 | 0.000185 |
| MOXD1 | 2.178569 | 4.968825 | 1.189524 | 1.59E-32 | 2.05E-30 |
| TNC | 12.17815 | 38.05447 | 1.643771 | 1.10E-12 | 9.54E-12 |
| AP003071.3 | 0.228743 | 1.049857 | 2.198397 | 5.45E-24 | 1.57E-22 |
| SGCD | 0.898716 | 3.495145 | 1.959416 | 9.16E-32 | 9.71E-30 |
| ALB | 1.605993 | 68.6835 | 5.418426 | 0.033178 | 0.049344 |
| AC241952.1 | 0.58577 | 0.952707 | 0.701697 | 2.74E-12 | 2.26E-11 |
| ATG9B | 1.029458 | 0.527996 | -0.96329 | 6.55E-06 | 2.00E-05 |
| TPBGL | 0.547472 | 0.900693 | 0.718249 | 1.07E-15 | 1.28E-14 |
| SNED1 | 0.920039 | 2.203127 | 1.259785 | 1.59E-30 | 1.32E-28 |
| TBXA2R | 1.003985 | 1.932064 | 0.944405 | 4.40E-21 | 9.20E-20 |
| PCBP3 | 0.344083 | 0.791221 | 1.201324 | 1.13E-17 | 1.64E-16 |
| NKX2-5 | 0.32174 | 1.05792 | 1.717265 | 0.031148 | 0.046678 |
| AL357055.2 | 0.725526 | 0.476 | -0.60807 | 7.75E-06 | 2.34E-05 |
| CBFA2T3 | 0.9537 | 1.771281 | 0.893185 | 1.11E-21 | 2.51E-20 |
| SLC25A10 | 8.882149 | 5.725446 | -0.63352 | 2.55E-15 | 2.92E-14 |
| MIR6784 | 1.079283 | 0.714342 | -0.59539 | 0.02126 | 0.033078 |
| SNORA70G | 0.704341 | 1.099221 | 0.642135 | 0.00026 | 0.000596 |
| IGHV3-7 | 9.030687 | 28.41117 | 1.653551 | 4.27E-09 | 2.23E-08 |
| CT83 | 9.871026 | 4.798773 | -1.04053 | 0.012926 | 0.021077 |
| FRZB | 3.95548 | 12.88156 | 1.703383 | 2.44E-28 | 1.27E-26 |
| PDPN | 6.142792 | 12.76748 | 1.055507 | 2.88E-19 | 4.98E-18 |
| LHFPL6 | 7.513501 | 19.54167 | 1.378996 | 2.67E-31 | 2.64E-29 |
| LINC00926 | 0.920521 | 1.544777 | 0.746877 | 3.55E-07 | 1.35E-06 |
| AC002091.1 | 0.412903 | 0.931355 | 1.173529 | 5.87E-15 | 6.53E-14 |
| CBX7 | 2.438051 | 4.986601 | 1.032329 | 8.07E-18 | 1.19E-16 |
| AC015922.2 | 3.247076 | 7.197614 | 1.148378 | 2.06E-24 | 6.22E-23 |
| PLA2G4C | 0.791721 | 1.72981 | 1.12755 | 5.38E-18 | 8.12E-17 |
| AOAH | 2.330846 | 3.863591 | 0.729089 | 9.66E-10 | 5.58E-09 |
| SRPX2 | 3.734616 | 6.079806 | 0.703065 | 1.41E-12 | 1.20E-11 |
| AC084033.3 | 0.774805 | 1.375904 | 0.828474 | 3.49E-18 | 5.39E-17 |
| NCKAP5L | 2.578764 | 4.236788 | 0.716291 | 4.87E-20 | 9.30E-19 |
| GNAZ | 1.5764 | 3.292326 | 1.062474 | 1.78E-15 | 2.09E-14 |
| TRAV1-2 | 0.547504 | 0.875024 | 0.676454 | 2.58E-05 | 7.11E-05 |
| TMEM88 | 3.058033 | 6.425301 | 1.07116 | 1.90E-23 | 5.20E-22 |
| PSMB9 | 34.56264 | 20.89664 | -0.72594 | 1.02E-05 | 3.01E-05 |
| HIGD1B | 0.768609 | 1.236002 | 0.685358 | 1.71E-18 | 2.74E-17 |
| PYGM | 0.267649 | 1.239449 | 2.211282 | 4.92E-21 | 1.02E-19 |
| EEF1B2P2 | 1.232232 | 0.800206 | -0.62283 | 4.70E-07 | 1.74E-06 |
| MAPK15 | 2.493177 | 1.504791 | -0.72842 | 0.00016 | 0.000382 |
| SNN | 4.977852 | 7.844936 | 0.656238 | 6.30E-15 | 6.98E-14 |
| STAB1 | 4.098616 | 8.50979 | 1.053987 | 1.21E-23 | 3.37E-22 |
| RBP7 | 1.510204 | 3.093307 | 1.034407 | 5.13E-17 | 7.00E-16 |
| SPARCL1 | 30.95417 | 132.9361 | 2.102527 | 4.61E-32 | 5.11E-30 |
| CEP55 | 14.33317 | 9.084259 | -0.65792 | 1.36E-14 | 1.46E-13 |
| KCNA3 | 0.790589 | 1.832073 | 1.212476 | 4.13E-14 | 4.18E-13 |
| S100A6 | 1046.478 | 667.9561 | -0.64772 | 3.87E-10 | 2.37E-09 |
| TACSTD2 | 95.30009 | 61.05536 | -0.64236 | 0.024775 | 0.037955 |
| AC005856.1 | 0.432425 | 0.671686 | 0.635336 | 8.40E-08 | 3.53E-07 |
| IGKC | 1030.846 | 1629.24 | 0.660371 | 7.08E-06 | 2.15E-05 |
| SLC6A19 | 4.643391 | 7.133975 | 0.619527 | 4.64E-06 | 1.46E-05 |
| BAG2 | 3.139011 | 5.383455 | 0.778222 | 0.002681 | 0.00505 |
| NAPSB | 2.402829 | 4.789759 | 0.995219 | 8.01E-12 | 6.21E-11 |
| IL6R | 2.900483 | 4.51184 | 0.637423 | 7.01E-17 | 9.44E-16 |
| GLI2 | 1.25442 | 2.717104 | 1.115049 | 5.87E-20 | 1.11E-18 |
| AC104794.2 | 1.590107 | 2.477775 | 0.639921 | 3.07E-16 | 3.89E-15 |
| CSRP2 | 1.377374 | 2.286406 | 0.731161 | 4.86E-18 | 7.39E-17 |
| LAMA4 | 5.86331 | 13.53327 | 1.206723 | 4.55E-32 | 5.08E-30 |
| VIM | 76.65852 | 133.7649 | 0.803182 | 3.08E-25 | 1.05E-23 |
| CYP2T1P | 1.397574 | 2.571645 | 0.879767 | 3.02E-14 | 3.12E-13 |
| PAX5 | 1.245133 | 2.265 | 0.863211 | 1.19E-05 | 3.48E-05 |
| FOLH1 | 0.89685 | 1.577485 | 0.814687 | 0.009133 | 0.015378 |
| EFL1 | 8.999869 | 4.76104 | -0.91863 | 0.001178 | 0.002387 |
| RPS3AP47 | 8.229444 | 4.565548 | -0.85001 | 4.45E-05 | 0.000118 |
| NCF1B | 0.855269 | 1.395371 | 0.706198 | 3.96E-08 | 1.76E-07 |
| CCR4 | 0.784642 | 1.860429 | 1.245529 | 5.04E-16 | 6.22E-15 |
| GRASP | 2.279448 | 4.300221 | 0.915726 | 1.57E-30 | 1.32E-28 |
| MPP2 | 0.524629 | 1.013141 | 0.949467 | 1.12E-15 | 1.33E-14 |
| PALLD | 14.53626 | 36.53982 | 1.329813 | 7.96E-20 | 1.48E-18 |
| RNU1-70P | 0.283641 | 0.843179 | 1.571772 | 0.018005 | 0.028456 |
| TXNP6 | 2.556886 | 1.530211 | -0.74066 | 7.03E-10 | 4.13E-09 |
| SOX18 | 11.36803 | 18.85851 | 0.730233 | 3.87E-14 | 3.93E-13 |
| JUP | 346.8347 | 195.0927 | -0.83009 | 0.001632 | 0.003215 |
| SERPINE1 | 26.14162 | 47.6797 | 0.867027 | 1.02E-07 | 4.23E-07 |
| EFHC2 | 0.44035 | 0.682984 | 0.6332 | 4.30E-10 | 2.61E-09 |
| NPR3 | 0.299374 | 0.761807 | 1.347476 | 2.22E-16 | 2.86E-15 |
| FGD3 | 1.690159 | 2.678365 | 0.664194 | 1.18E-08 | 5.76E-08 |
| TESPA1 | 0.532329 | 1.019779 | 0.937866 | 5.87E-12 | 4.64E-11 |
| MAP3K3 | 4.212065 | 7.066 | 0.746366 | 9.74E-26 | 3.62E-24 |
| SDC2 | 7.393768 | 15.17037 | 1.036875 | 3.98E-29 | 2.49E-27 |
| CCR2 | 0.567908 | 1.031162 | 0.860543 | 4.68E-13 | 4.24E-12 |
| CST2 | 3.069691 | 8.967566 | 1.546623 | 2.22E-14 | 2.33E-13 |
| SCN1B | 0.664598 | 1.58445 | 1.253429 | 9.93E-29 | 5.58E-27 |
| EVPL | 16.70399 | 9.37377 | -0.83349 | 6.78E-07 | 2.45E-06 |
| RPL7P19 | 1.013975 | 0.653874 | -0.63294 | 8.88E-06 | 2.65E-05 |
| MCC | 1.050734 | 2.35143 | 1.16214 | 1.06E-30 | 9.22E-29 |
| RPL7AP11 | 1.882186 | 1.233385 | -0.60979 | 2.03E-10 | 1.30E-09 |
| CCL11 | 6.592337 | 19.37927 | 1.555652 | 3.31E-19 | 5.68E-18 |
| RIMS3 | 0.57423 | 0.904911 | 0.656148 | 2.12E-10 | 1.35E-09 |
| TSC22D3 | 11.45538 | 25.03498 | 1.12792 | 1.45E-24 | 4.49E-23 |
| TUBB6 | 8.384702 | 16.17496 | 0.947931 | 5.90E-24 | 1.69E-22 |
| DLGAP5 | 8.237779 | 5.413853 | -0.6056 | 2.00E-15 | 2.33E-14 |
| MORN5 | 0.242068 | 1.564973 | 2.692652 | 4.44E-07 | 1.65E-06 |
| AC073343.1 | 0.386153 | 0.771194 | 0.99792 | 9.81E-11 | 6.59E-10 |
| LINC02084 | 0.329771 | 0.808569 | 1.293907 | 6.12E-06 | 1.88E-05 |
| REEP1 | 0.826851 | 2.525716 | 1.610994 | 1.32E-21 | 2.94E-20 |
| TLN1 | 43.53587 | 76.03568 | 0.804472 | 2.05E-16 | 2.65E-15 |
| GNG7 | 1.098055 | 2.282325 | 1.055554 | 5.80E-18 | 8.73E-17 |
| FCER2 | 0.738671 | 1.76194 | 1.254161 | 1.08E-11 | 8.29E-11 |
| UNC5C | 0.39987 | 0.974626 | 1.285317 | 4.21E-31 | 4.01E-29 |
| CACNA1H | 2.983901 | 10.88769 | 1.867426 | 1.23E-25 | 4.52E-24 |
| PIK3R5 | 1.090481 | 2.049073 | 0.910006 | 2.28E-16 | 2.92E-15 |
| EGR3 | 2.317148 | 3.976094 | 0.779002 | 6.22E-10 | 3.68E-09 |
| LRRN4CL | 0.705856 | 2.566115 | 1.862141 | 3.39E-36 | 1.25E-33 |
| LYPD3 | 17.44759 | 2.335033 | -2.90151 | 0.009237 | 0.015542 |
| MEG3 | 0.626606 | 1.699023 | 1.439075 | 1.75E-20 | 3.46E-19 |
| KRTCAP3 | 33.64243 | 21.67396 | -0.63432 | 6.20E-12 | 4.89E-11 |
| PIWIL1 | 1.546251 | 0.732737 | -1.07741 | 1.45E-05 | 4.19E-05 |
| STARD9 | 0.363415 | 0.917522 | 1.336123 | 3.63E-21 | 7.61E-20 |
| SLC7A4 | 1.148213 | 2.10849 | 0.87682 | 1.13E-06 | 3.95E-06 |
| SV2A | 0.991625 | 1.810246 | 0.868319 | 7.62E-27 | 3.21E-25 |
| F12 | 5.098069 | 2.492096 | -1.03259 | 3.61E-09 | 1.92E-08 |
| ARHGAP10 | 1.301724 | 2.033776 | 0.643738 | 1.04E-09 | 6.02E-09 |
| LINC00865 | 0.321245 | 0.707617 | 1.139292 | 9.85E-19 | 1.61E-17 |
| EOGT | 4.434824 | 6.738191 | 0.603484 | 2.49E-14 | 2.60E-13 |
| RF02141 | 1.053116 | 0.537467 | -0.97042 | 0.004527 | 0.008133 |
| SELENOP | 8.725116 | 19.35144 | 1.149195 | 7.29E-22 | 1.68E-20 |
| ARHGAP15 | 0.754066 | 1.314977 | 0.802274 | 6.94E-14 | 6.90E-13 |
| LINC02532 | 0.710812 | 2.724251 | 1.938321 | 7.98E-08 | 3.37E-07 |
| ANKRD29 | 1.054469 | 1.644712 | 0.641319 | 1.58E-08 | 7.54E-08 |
| MRAS | 1.761383 | 4.094155 | 1.216857 | 7.39E-30 | 5.31E-28 |
| ZWINT | 21.68549 | 13.64106 | -0.66877 | 6.65E-16 | 8.09E-15 |
| GJC1 | 0.981109 | 2.720732 | 1.47151 | 8.29E-25 | 2.65E-23 |
| EEF1A2 | 7.333785 | 14.36027 | 0.969452 | 5.50E-10 | 3.29E-09 |
| RYR2 | 0.411801 | 0.676607 | 0.716372 | 8.19E-09 | 4.10E-08 |
| RPL26P37 | 1.173313 | 0.774073 | -0.60005 | 6.72E-05 | 0.000172 |
| ANKRD37 | 1.811991 | 1.084799 | -0.74015 | 0.008058 | 0.013747 |
| LINC02303 | 0.743805 | 0.388281 | -0.93782 | 0.024969 | 0.038215 |
| CXorf21 | 0.881778 | 1.494463 | 0.761141 | 1.03E-11 | 7.90E-11 |
| GINS2 | 6.729721 | 3.764954 | -0.83791 | 1.68E-13 | 1.61E-12 |
| VAT1L | 0.586675 | 1.343302 | 1.195149 | 1.23E-19 | 2.23E-18 |
| ATP10B | 10.83289 | 6.35225 | -0.77008 | 0.001227 | 0.002479 |
| AREG | 26.20457 | 15.46081 | -0.7612 | 0.002367 | 0.004519 |
| MYEF2 | 0.460776 | 0.876932 | 0.9284 | 4.19E-15 | 4.72E-14 |
| AL365361.1 | 0.901439 | 1.595798 | 0.823977 | 5.17E-10 | 3.11E-09 |
| SVIL2P | 0.313575 | 0.732606 | 1.224229 | 2.69E-08 | 1.23E-07 |
| PTGER3 | 0.36131 | 1.515477 | 2.068463 | 1.22E-34 | 2.65E-32 |
| ARMH4 | 0.343991 | 0.732673 | 1.090797 | 2.11E-27 | 9.51E-26 |
| JCHAIN | 138.0893 | 384.0688 | 1.475764 | 3.14E-07 | 1.20E-06 |
| AC093010.3 | 6.408115 | 12.10498 | 0.917629 | 1.71E-18 | 2.74E-17 |
| NR4A3 | 1.565145 | 3.996457 | 1.352425 | 3.15E-17 | 4.41E-16 |
| PLIN4 | 0.994124 | 7.058312 | 2.827826 | 9.67E-14 | 9.49E-13 |
| AC135068.2 | 1.494358 | 3.411882 | 1.191042 | 2.33E-08 | 1.08E-07 |
| ALPI | 1.738698 | 4.420704 | 1.346269 | 0.001362 | 0.002727 |
| WLS | 12.02268 | 19.55173 | 0.701538 | 1.34E-14 | 1.44E-13 |
| CORO2B | 0.441265 | 1.028002 | 1.220126 | 1.12E-28 | 6.24E-27 |
| CXCL2 | 25.94027 | 16.55352 | -0.64806 | 0.00405 | 0.00736 |
| COL17A1 | 14.64085 | 7.14641 | -1.03471 | 0.025916 | 0.039518 |
| SMAGP | 18.17857 | 11.97575 | -0.60212 | 2.19E-14 | 2.30E-13 |
| MEDAG | 2.16626 | 6.525477 | 1.590877 | 3.96E-18 | 6.09E-17 |
| RNU4ATAC | 1.52003 | 1.011998 | -0.58689 | 0.010921 | 0.01809 |
| SARDH | 0.567772 | 0.869943 | 0.615608 | 3.08E-11 | 2.22E-10 |
| DOCK2 | 1.56226 | 2.558787 | 0.711825 | 1.72E-12 | 1.45E-11 |
| CCDC144NL-AS1 | 0.303202 | 0.707192 | 1.221823 | 1.28E-19 | 2.31E-18 |
| MIR3153 | 1.21425 | 0.738076 | -0.71822 | 2.35E-05 | 6.52E-05 |
| RBP5 | 1.869922 | 3.662858 | 0.969992 | 2.16E-21 | 4.64E-20 |
| CTF1 | 3.283358 | 5.377099 | 0.711656 | 8.03E-13 | 7.05E-12 |
| DIRAS3 | 0.860482 | 1.37937 | 0.680793 | 1.70E-17 | 2.44E-16 |
| AC090152.1 | 0.727926 | 1.35991 | 0.901646 | 8.57E-12 | 6.61E-11 |
| FERMT2 | 3.355654 | 11.2322 | 1.742975 | 9.71E-35 | 2.23E-32 |
| LBP | 1.050832 | 2.557086 | 1.282969 | 0.004125 | 0.007479 |
| AC018868.1 | 5.102031 | 3.250757 | -0.6503 | 9.00E-07 | 3.19E-06 |
| CDK1 | 16.14191 | 10.21428 | -0.66022 | 2.45E-16 | 3.13E-15 |
| LUM | 92.87571 | 234.8587 | 1.338419 | 1.83E-26 | 7.36E-25 |
| CLDN12 | 14.95676 | 9.685805 | -0.62685 | 5.38E-06 | 1.67E-05 |
| LINC00520 | 0.876743 | 0.480267 | -0.86832 | 0.0003 | 0.000681 |
| COL15A1 | 17.84823 | 40.10005 | 1.167823 | 2.09E-22 | 5.16E-21 |
| NLRP7 | 0.63129 | 1.160355 | 0.878193 | 9.91E-05 | 0.000245 |
| CCL20 | 66.26901 | 37.74524 | -0.81204 | 3.58E-06 | 1.15E-05 |
| C6orf15 | 4.797936 | 0.860947 | -2.47842 | 0.032692 | 0.04872 |
| RASIP1 | 2.1709 | 4.627431 | 1.091918 | 1.78E-28 | 9.53E-27 |
| HMGA1 | 286.1429 | 186.0428 | -0.6211 | 8.16E-14 | 8.06E-13 |
| PRG4 | 0.233111 | 0.953826 | 2.032707 | 7.49E-17 | 1.01E-15 |
| PCDHGA12 | 0.392434 | 0.977974 | 1.317344 | 8.05E-25 | 2.58E-23 |
| BSPRY | 10.59032 | 6.778598 | -0.64369 | 2.82E-15 | 3.22E-14 |
| CDCP1 | 22.9178 | 14.1369 | -0.697 | 5.71E-13 | 5.10E-12 |
| TSPAN32 | 0.303512 | 0.802159 | 1.402136 | 1.22E-10 | 8.04E-10 |
| CASTOR2 | 3.410051 | 5.252594 | 0.623237 | 3.82E-11 | 2.72E-10 |
| SYPL2 | 0.244599 | 0.816589 | 1.739191 | 2.22E-29 | 1.45E-27 |
| TRAV8-4 | 0.554339 | 0.950838 | 0.778431 | 6.19E-09 | 3.16E-08 |
| MIR4653 | 5.322959 | 3.333384 | -0.67524 | 2.36E-06 | 7.81E-06 |
| EBF4 | 2.520617 | 4.724454 | 0.906371 | 2.26E-21 | 4.85E-20 |
| AC139795.1 | 1.080094 | 1.857277 | 0.782033 | 4.92E-13 | 4.44E-12 |
| AL356535.1 | 4.45098 | 2.930478 | -0.60299 | 8.31E-07 | 2.97E-06 |
| COL1A2 | 128.8542 | 248.9872 | 0.950332 | 3.22E-13 | 2.99E-12 |
| AL354872.2 | 0.645603 | 0.370131 | -0.80261 | 0.000192 | 0.000452 |
| GPR27 | 0.729342 | 1.59414 | 1.128111 | 3.96E-20 | 7.62E-19 |
| KRT8P45 | 4.953967 | 3.00694 | -0.72029 | 1.27E-09 | 7.23E-09 |
| PAPLN | 1.395296 | 4.120597 | 1.562282 | 4.43E-33 | 6.48E-31 |
| ADAM23 | 0.447764 | 1.099376 | 1.295874 | 6.76E-31 | 6.13E-29 |
| GSDMB | 15.67804 | 10.05895 | -0.64026 | 8.75E-05 | 0.000219 |
| RPSAP8 | 1.652537 | 0.925819 | -0.83588 | 0.000216 | 0.000504 |
| KLF12 | 1.149031 | 2.396935 | 1.060773 | 6.72E-20 | 1.27E-18 |
| CYP2B7P | 0.803796 | 1.720056 | 1.097555 | 2.71E-06 | 8.88E-06 |
| OVOL1 | 5.875249 | 3.90659 | -0.58874 | 2.71E-06 | 8.88E-06 |
| LRRC3 | 1.378185 | 2.241226 | 0.701518 | 1.91E-08 | 8.96E-08 |
| NEGR1 | 0.398485 | 1.755473 | 2.139262 | 1.54E-30 | 1.30E-28 |
| TSPAN7 | 3.009529 | 5.5864 | 0.892381 | 2.22E-15 | 2.56E-14 |
| KRT8P10 | 1.227163 | 0.792938 | -0.63005 | 4.92E-07 | 1.82E-06 |
| DNM3OS | 0.474862 | 1.027161 | 1.113083 | 1.19E-20 | 2.37E-19 |
| CIDEC | 1.706674 | 2.871646 | 0.750691 | 0.001615 | 0.003186 |
| L1CAM | 2.717383 | 4.200311 | 0.628278 | 6.63E-12 | 5.21E-11 |
| FHOD3 | 0.441007 | 0.788741 | 0.83875 | 9.52E-20 | 1.75E-18 |
| ZNF362 | 7.98754 | 12.72931 | 0.672331 | 1.59E-22 | 3.96E-21 |
| SMAD9 | 1.557836 | 3.947305 | 1.341324 | 2.32E-25 | 8.02E-24 |
| PRELP | 4.052795 | 20.37237 | 2.329624 | 4.55E-26 | 1.78E-24 |
| CAVIN2 | 3.604983 | 10.55084 | 1.549294 | 9.86E-34 | 1.71E-31 |
| MAPK8IP1 | 2.19433 | 4.101495 | 0.90237 | 8.31E-16 | 1.00E-14 |
| IGKV1OR2-3 | 0.280457 | 0.826978 | 1.560069 | 8.70E-07 | 3.10E-06 |
| SLC7A9 | 1.457898 | 2.457222 | 0.753138 | 3.80E-05 | 0.000102 |
| AL157871.2 | 1.216893 | 0.692066 | -0.81422 | 0.012849 | 0.020969 |
| IGHV1-3 | 7.469985 | 12.86099 | 0.783825 | 7.84E-05 | 0.000198 |
| SMARCD3 | 1.304684 | 3.127276 | 1.261206 | 2.23E-32 | 2.72E-30 |
| TSHZ3 | 1.254729 | 3.694281 | 1.557918 | 2.51E-30 | 2.00E-28 |
| MYO1F | 2.431445 | 3.648305 | 0.585412 | 7.52E-10 | 4.40E-09 |
| CD200 | 2.395227 | 4.68696 | 0.96849 | 1.12E-22 | 2.82E-21 |
| KIAA1755 | 0.785369 | 1.71945 | 1.130505 | 8.75E-24 | 2.47E-22 |
| SMTNL2 | 0.484682 | 1.507098 | 1.636662 | 3.96E-16 | 4.96E-15 |
| AC096733.2 | 0.549664 | 1.063619 | 0.95236 | 7.65E-22 | 1.76E-20 |
| SHISAL2A | 0.499098 | 0.974682 | 0.965608 | 2.05E-12 | 1.72E-11 |
| KRTDAP | 9.937978 | 0.340964 | -4.86526 | 0.012813 | 0.020918 |
| AC069549.1 | 0.556561 | 0.962491 | 0.790234 | 2.77E-06 | 9.04E-06 |
| FAM117A | 3.929258 | 6.478374 | 0.721375 | 2.46E-19 | 4.27E-18 |
| F8 | 0.609648 | 1.247441 | 1.032924 | 6.37E-20 | 1.20E-18 |
| TAAR3P | 0.76932 | 0.410619 | -0.90578 | 0.002994 | 0.005582 |
| GMPSP1 | 1.673941 | 0.990045 | -0.75768 | 3.44E-11 | 2.46E-10 |
| MMP24 | 0.896819 | 1.64731 | 0.877222 | 3.48E-14 | 3.57E-13 |
| AC105219.1 | 0.745546 | 0.475691 | -0.64827 | 0.002624 | 0.004956 |
| AC139085.1 | 0.893872 | 0.558085 | -0.67958 | 4.04E-05 | 0.000108 |
| SIGLEC6 | 0.258016 | 0.835418 | 1.695035 | 1.12E-23 | 3.12E-22 |
| DUSP15 | 0.66421 | 1.038735 | 0.645115 | 1.13E-08 | 5.52E-08 |
| IGLV3-12 | 1.162598 | 2.079883 | 0.83915 | 9.60E-08 | 4.00E-07 |
| RRN3P2 | 0.562471 | 0.858788 | 0.610525 | 9.90E-11 | 6.65E-10 |
| ABCG2 | 1.15671 | 1.735339 | 0.585191 | 1.77E-12 | 1.49E-11 |
| C1QTNF1 | 7.738206 | 12.02458 | 0.635915 | 6.03E-09 | 3.09E-08 |
| IGF1 | 0.196882 | 0.953347 | 2.275668 | 4.38E-30 | 3.29E-28 |
| IGHV3-11 | 29.54027 | 68.35392 | 1.210341 | 1.86E-08 | 8.77E-08 |
| AXL | 9.848779 | 16.6322 | 0.755962 | 3.40E-22 | 8.12E-21 |
| AP001528.3 | 0.246324 | 0.823984 | 1.742062 | 1.55E-27 | 7.26E-26 |
| RPL31P2 | 1.642337 | 1.051793 | -0.6429 | 3.69E-05 | 9.90E-05 |
| MST1R | 17.76765 | 11.14478 | -0.67288 | 2.68E-08 | 1.22E-07 |
| AP000892.3 | 0.796033 | 3.463348 | 2.121268 | 5.10E-21 | 1.05E-19 |
| IL23A | 2.665494 | 1.775144 | -0.58647 | 0.004279 | 0.007734 |
| RHOV | 7.718191 | 2.944927 | -1.39003 | 5.51E-13 | 4.93E-12 |
| LZTS1 | 1.568902 | 3.43735 | 1.131542 | 1.61E-19 | 2.87E-18 |
| TCF21 | 0.739943 | 2.449376 | 1.726929 | 2.72E-40 | 6.60E-37 |
| BCHE | 0.359609 | 1.742062 | 2.276295 | 1.20E-18 | 1.94E-17 |
| ATXN1 | 2.249015 | 3.477226 | 0.628643 | 2.06E-20 | 4.06E-19 |
| CENPX | 37.39289 | 23.05583 | -0.69763 | 7.45E-15 | 8.18E-14 |
| GPR132 | 1.314583 | 2.072671 | 0.656885 | 8.65E-10 | 5.03E-09 |
| VWF | 11.49673 | 25.18388 | 1.131277 | 1.66E-25 | 5.95E-24 |
| CHST13 | 1.614078 | 5.208001 | 1.69002 | 2.72E-06 | 8.91E-06 |
| HHIP-AS1 | 0.366392 | 0.983138 | 1.424008 | 2.02E-24 | 6.11E-23 |
| FAM124A | 0.679405 | 1.756426 | 1.370299 | 8.08E-21 | 1.64E-19 |
| CDT1 | 13.31883 | 8.374868 | -0.66933 | 4.14E-13 | 3.78E-12 |
| PDE9A | 0.860215 | 2.024929 | 1.235102 | 8.96E-19 | 1.47E-17 |
| ABI3BP | 1.450349 | 6.685526 | 2.204641 | 2.00E-27 | 9.04E-26 |
| CDC45 | 6.363743 | 4.192198 | -0.60217 | 5.10E-13 | 4.59E-12 |
| IGLV9-49 | 7.46276 | 17.61179 | 1.238761 | 1.72E-06 | 5.83E-06 |
| PYY | 0.263378 | 2.570597 | 3.286895 | 0.012739 | 0.020806 |
| C3orf70 | 1.385698 | 4.009452 | 1.532792 | 4.92E-21 | 1.02E-19 |
| NFIA | 6.127848 | 9.768201 | 0.672712 | 4.29E-07 | 1.60E-06 |
| C15orf48 | 66.98962 | 33.28501 | -1.00907 | 3.51E-06 | 1.13E-05 |
| SULF1 | 18.5102 | 35.04343 | 0.920823 | 1.66E-11 | 1.24E-10 |
| ADAM33 | 0.539482 | 2.712446 | 2.329948 | 4.39E-35 | 1.15E-32 |
| SLC34A2 | 3.593831 | 6.688146 | 0.896084 | 0.000853 | 0.001783 |
| KANK3 | 1.117135 | 2.357636 | 1.077537 | 7.62E-26 | 2.90E-24 |
| AC000089.1 | 4.388253 | 2.751286 | -0.67354 | 4.62E-08 | 2.02E-07 |
| PGA5 | 1.609981 | 28.92702 | 4.167302 | 0.000411 | 0.000911 |
| AC079150.2 | 1.223655 | 0.732529 | -0.74024 | 0.030528 | 0.04585 |
| TFAP2A-AS1 | 0.748747 | 0.378856 | -0.98283 | 5.59E-13 | 5.00E-12 |
| AC009005.1 | 3.105978 | 1.687632 | -0.88005 | 1.46E-11 | 1.10E-10 |
| RPL39P38 | 1.871732 | 1.236092 | -0.59859 | 6.91E-05 | 0.000177 |
| SH2D3C | 2.573494 | 4.84958 | 0.914132 | 2.61E-23 | 7.03E-22 |
| PDZRN4 | 0.206624 | 1.341499 | 2.698763 | 4.80E-17 | 6.59E-16 |
| SIGLEC14 | 1.189713 | 1.914889 | 0.686647 | 2.40E-06 | 7.94E-06 |
| PRKACB | 5.02693 | 7.962044 | 0.663461 | 3.37E-13 | 3.11E-12 |
| CDKN3 | 11.67052 | 7.322711 | -0.67242 | 1.38E-13 | 1.33E-12 |
| IGHV2-70 | 15.21827 | 33.07756 | 1.120048 | 6.38E-08 | 2.74E-07 |
| AC093535.2 | 0.596999 | 1.110019 | 0.894785 | 1.50E-19 | 2.68E-18 |
| ENTPD1 | 3.522205 | 6.233159 | 0.823484 | 1.75E-29 | 1.17E-27 |
| NEUROD1 | 0.151502 | 1.097061 | 2.856231 | 0.01947 | 0.030522 |
| MNX1-AS1 | 2.752111 | 1.583232 | -0.79767 | 6.23E-11 | 4.31E-10 |
| HES2 | 1.357673 | 0.720424 | -0.91422 | 0.003015 | 0.005614 |
| LINC00641 | 1.26713 | 1.947587 | 0.620123 | 8.91E-07 | 3.16E-06 |
| LAMB2 | 21.94858 | 36.1193 | 0.718642 | 1.63E-17 | 2.35E-16 |
| LDOC1 | 5.335122 | 11.94347 | 1.162629 | 1.25E-26 | 5.10E-25 |
| SLC8A1 | 0.541509 | 1.27546 | 1.235961 | 3.21E-25 | 1.09E-23 |
| MUC6 | 44.34646 | 71.85538 | 0.696277 | 0.015146 | 0.024324 |
| FOXF1 | 4.474008 | 14.80246 | 1.726198 | 3.74E-34 | 6.82E-32 |
| PSORS1C2 | 0.673698 | 0.331199 | -1.0244 | 0.023617 | 0.036337 |
| BARX1 | 6.700396 | 30.52274 | 2.187566 | 2.67E-24 | 8.00E-23 |
| TNXB | 1.032392 | 5.872161 | 2.5079 | 2.40E-35 | 7.28E-33 |
| AL133342.1 | 0.374778 | 0.628972 | 0.746961 | 5.29E-12 | 4.22E-11 |
| C3 | 55.5666 | 131.9826 | 1.248058 | 8.61E-21 | 1.74E-19 |
| PHLDB2 | 1.528368 | 3.766148 | 1.301097 | 3.24E-23 | 8.58E-22 |
| NLGN4X | 0.28265 | 1.036557 | 1.874714 | 2.21E-25 | 7.70E-24 |
| CYP26B1 | 0.916105 | 1.416869 | 0.629121 | 0.020162 | 0.0315 |
| LTBP1 | 7.914566 | 22.33307 | 1.496599 | 1.82E-31 | 1.83E-29 |
| SYT5 | 0.395046 | 0.637583 | 0.690592 | 0.014707 | 0.023682 |
| HTRA1 | 29.40208 | 53.71834 | 0.869496 | 1.33E-17 | 1.92E-16 |
| NARS2 | 7.569858 | 4.978664 | -0.60451 | 1.08E-08 | 5.30E-08 |
| IGLV3-21 | 109.7331 | 183.9298 | 0.745157 | 4.70E-08 | 2.05E-07 |
| FLRT2 | 0.231093 | 0.803752 | 1.798274 | 6.61E-35 | 1.67E-32 |
| MXRA8 | 17.50645 | 47.09255 | 1.427612 | 1.92E-27 | 8.76E-26 |
| JAM2 | 1.16112 | 4.203025 | 1.85591 | 8.93E-35 | 2.07E-32 |
| FREM1 | 0.344855 | 0.826169 | 1.260447 | 3.58E-19 | 6.12E-18 |
| ST3GAL6 | 0.588927 | 1.001343 | 0.765775 | 2.37E-13 | 2.22E-12 |
| NUP62CL | 2.462055 | 1.527505 | -0.68869 | 2.19E-07 | 8.62E-07 |
| GIMAP8 | 1.849174 | 3.630242 | 0.973185 | 3.24E-22 | 7.79E-21 |
| IGLJ3 | 0.418563 | 0.866584 | 1.049896 | 0.000397 | 0.000882 |
| CEP85L | 0.580421 | 0.978427 | 0.753364 | 2.47E-14 | 2.58E-13 |
| RPS19P7 | 3.017683 | 1.835587 | -0.7172 | 1.10E-05 | 3.22E-05 |
| SNRPGP2 | 11.00461 | 7.278919 | -0.59631 | 4.52E-15 | 5.08E-14 |
| TM6SF1 | 0.487554 | 0.96605 | 0.986537 | 2.46E-19 | 4.27E-18 |
| ENOX1 | 0.619982 | 1.520691 | 1.294429 | 1.78E-27 | 8.25E-26 |
| NHSL2 | 0.381387 | 1.580715 | 2.051251 | 2.58E-34 | 4.93E-32 |
| CHST1 | 1.579186 | 2.710023 | 0.779124 | 1.10E-12 | 9.54E-12 |
| ZNF287 | 0.576293 | 0.919088 | 0.673401 | 2.91E-15 | 3.31E-14 |
| RNU5B-1 | 0.924884 | 0.493182 | -0.90715 | 0.000458 | 0.001006 |
| PDE7B | 0.41978 | 1.680047 | 2.000795 | 2.27E-30 | 1.83E-28 |
| TP53INP1 | 5.207821 | 7.877579 | 0.597073 | 1.44E-12 | 1.22E-11 |
| GAL3ST4 | 2.793538 | 4.354852 | 0.640531 | 6.56E-11 | 4.52E-10 |
| AF001548.1 | 0.351123 | 2.221204 | 2.661294 | 6.76E-17 | 9.12E-16 |
| HSD17B14 | 3.664025 | 6.430799 | 0.811569 | 5.59E-12 | 4.44E-11 |
| STARD13 | 2.69819 | 4.098627 | 0.603149 | 1.30E-10 | 8.53E-10 |
| LAMB1 | 17.75939 | 27.6879 | 0.640673 | 4.21E-17 | 5.82E-16 |
| RPL7P13 | 0.656099 | 0.416843 | -0.65441 | 1.13E-06 | 3.95E-06 |
| TCF7L1 | 1.979821 | 4.656655 | 1.233924 | 2.80E-29 | 1.79E-27 |
| SNRPN | 2.888414 | 6.07512 | 1.072636 | 1.76E-25 | 6.27E-24 |
| MTTP | 1.47385 | 2.269743 | 0.622939 | 0.00246 | 0.00468 |
| IGLV1-40 | 145.3972 | 292.3844 | 1.007867 | 1.46E-06 | 5.01E-06 |
| ABCA8 | 0.283242 | 1.768149 | 2.642135 | 5.52E-31 | 5.17E-29 |
| TNFSF12 | 5.831073 | 11.50195 | 0.980045 | 1.29E-24 | 4.01E-23 |
| AC142381.1 | 0.830204 | 2.416205 | 1.541205 | 6.25E-11 | 4.32E-10 |
| SERPINF2 | 1.654421 | 4.017424 | 1.279944 | 2.45E-07 | 9.58E-07 |
| AFAP1 | 5.723483 | 8.763094 | 0.614547 | 1.95E-16 | 2.53E-15 |
| PNOC | 0.436649 | 0.763208 | 0.805604 | 7.96E-11 | 5.42E-10 |
| PHC1 | 1.307347 | 2.181267 | 0.738525 | 2.05E-18 | 3.25E-17 |
| ITGBL1 | 0.744839 | 3.182281 | 2.09506 | 3.41E-26 | 1.35E-24 |
| SLC2A3 | 5.473993 | 9.505536 | 0.796174 | 2.10E-09 | 1.16E-08 |
| HOXD8 | 0.760358 | 1.196091 | 0.653577 | 5.22E-19 | 8.75E-18 |
| CD209 | 2.295972 | 3.611961 | 0.653677 | 7.73E-09 | 3.89E-08 |
| PDE4B | 1.971009 | 3.714489 | 0.91423 | 2.07E-16 | 2.67E-15 |
| ZDHHC2 | 3.821513 | 6.384922 | 0.740525 | 4.09E-09 | 2.15E-08 |
| F2R | 14.08579 | 29.36276 | 1.059747 | 7.68E-28 | 3.71E-26 |
| HCLS1 | 5.076635 | 8.064439 | 0.667702 | 3.91E-13 | 3.58E-12 |
| IFITM1 | 192.1293 | 106.304 | -0.85388 | 0.0002 | 0.000469 |
| ZNF334 | 0.344302 | 0.829303 | 1.268225 | 5.77E-16 | 7.07E-15 |
| EIF5AL1 | 2.082255 | 1.065664 | -0.96639 | 1.71E-11 | 1.27E-10 |
| AC005262.1 | 1.590686 | 0.975478 | -0.70547 | 2.16E-08 | 1.00E-07 |
| MPZ | 0.622598 | 3.053363 | 2.294025 | 2.30E-11 | 1.69E-10 |
| NMU | 15.14468 | 6.76258 | -1.16317 | 7.21E-05 | 0.000183 |
| DSG3 | 11.54895 | 2.216978 | -2.38109 | 8.14E-05 | 0.000205 |
| PRRT1 | 0.838957 | 1.333971 | 0.669059 | 7.65E-08 | 3.24E-07 |
| AC037487.3 | 1.327047 | 0.740275 | -0.84209 | 1.85E-06 | 6.23E-06 |
| COMP | 4.27431 | 16.263 | 1.92783 | 5.79E-15 | 6.44E-14 |
| THBS1 | 28.29274 | 73.59327 | 1.379142 | 8.76E-23 | 2.23E-21 |
| LYL1 | 1.55508 | 2.513003 | 0.692424 | 2.57E-15 | 2.94E-14 |
| CRISPLD2 | 6.503303 | 17.07754 | 1.392856 | 9.16E-32 | 9.71E-30 |
| TCP11L1 | 2.047632 | 3.09269 | 0.594906 | 2.69E-15 | 3.07E-14 |
| MYO5A | 1.68989 | 2.78127 | 0.718815 | 3.28E-21 | 6.91E-20 |
| GNG11 | 4.375923 | 9.179074 | 1.068761 | 6.68E-28 | 3.28E-26 |
| DNAJB4 | 4.207413 | 6.364368 | 0.597084 | 1.29E-14 | 1.39E-13 |
| BNC2 | 0.42542 | 1.642042 | 1.948531 | 1.18E-28 | 6.50E-27 |
| ZIC2 | 3.229096 | 1.591887 | -1.02039 | 0.009236 | 0.015542 |
| CDK14 | 2.390208 | 3.752326 | 0.650649 | 1.37E-22 | 3.44E-21 |
| IGKV4-1 | 243.3127 | 526.377 | 1.113285 | 2.85E-09 | 1.54E-08 |
| TIMP3 | 2.497151 | 6.944691 | 1.475628 | 4.65E-26 | 1.81E-24 |
| LINC01781 | 0.535732 | 1.170492 | 1.127532 | 2.09E-10 | 1.33E-09 |
| ULK2 | 1.250193 | 1.875922 | 0.585449 | 4.81E-13 | 4.35E-12 |
| ITGA5 | 12.52409 | 29.45914 | 1.234009 | 3.58E-20 | 6.93E-19 |
| COL16A1 | 3.920758 | 8.232655 | 1.070225 | 2.54E-20 | 4.99E-19 |
| TRAV26-1 | 0.688464 | 1.040361 | 0.595633 | 3.71E-06 | 1.19E-05 |
| IGKV6-21 | 7.564632 | 11.80831 | 0.64246 | 6.75E-05 | 0.000173 |
| POPDC2 | 1.017893 | 4.241802 | 2.059091 | 4.04E-17 | 5.60E-16 |
| LAMC2 | 43.16222 | 27.71288 | -0.63921 | 0.002121 | 0.004082 |
| FABP3 | 5.533043 | 10.77185 | 0.96112 | 2.20E-12 | 1.83E-11 |
| RNLS | 0.380568 | 0.663936 | 0.80289 | 9.77E-19 | 1.60E-17 |
| TRPC1 | 0.467763 | 1.158044 | 1.307841 | 4.35E-28 | 2.19E-26 |
| NCAM1 | 0.636051 | 1.133133 | 0.833102 | 6.06E-19 | 1.01E-17 |
| ITGA11 | 2.953488 | 6.133121 | 1.054202 | 3.95E-14 | 4.00E-13 |
| PTMAP2 | 22.09954 | 13.6213 | -0.69815 | 1.35E-08 | 6.53E-08 |
| PPP1R16B | 2.32032 | 4.743804 | 1.031721 | 1.93E-15 | 2.25E-14 |
| ZBTB10 | 1.97613 | 3.052584 | 0.627353 | 3.72E-13 | 3.42E-12 |
| IRX3 | 1.442141 | 2.195704 | 0.606472 | 0.01114 | 0.018405 |
| KCNAB1 | 0.385794 | 0.612407 | 0.666662 | 1.39E-19 | 2.51E-18 |
| SSPN | 2.401178 | 5.915883 | 1.300851 | 3.00E-23 | 8.02E-22 |
| ZNF569 | 0.452463 | 0.739053 | 0.707878 | 6.20E-16 | 7.57E-15 |
| PTPRB | 2.07331 | 3.537848 | 0.770936 | 5.76E-22 | 1.35E-20 |
| COX4I2 | 1.776562 | 2.784284 | 0.648219 | 2.31E-15 | 2.65E-14 |
| CDC42EP5 | 92.72448 | 57.10783 | -0.69926 | 4.40E-05 | 0.000116 |
| ELANE | 0.092573 | 7.096511 | 6.260379 | 3.75E-19 | 6.38E-18 |
| IL24 | 1.99546 | 4.337832 | 1.120253 | 3.85E-06 | 1.23E-05 |
| LAMP5 | 1.193785 | 2.717289 | 1.186625 | 2.47E-12 | 2.05E-11 |
| IGHV3OR16-9 | 1.569619 | 5.227926 | 1.735824 | 3.57E-11 | 2.55E-10 |
| TRBV20-1 | 2.195873 | 3.918842 | 0.835632 | 8.79E-08 | 3.69E-07 |
| IL32 | 65.37237 | 42.04352 | -0.6368 | 0.001423 | 0.002839 |
| LMOD1 | 7.115482 | 41.59535 | 2.547389 | 7.47E-24 | 2.13E-22 |
| CAND2 | 0.407831 | 1.524128 | 1.901941 | 4.79E-29 | 2.94E-27 |
| AC091042.1 | 2.936766 | 1.702039 | -0.78696 | 0.000288 | 0.000657 |
| AL162231.2 | 0.765051 | 1.189481 | 0.636705 | 8.87E-09 | 4.42E-08 |
| GSDME | 0.664892 | 1.349623 | 1.021364 | 1.77E-19 | 3.15E-18 |
| PDE2A | 0.625759 | 1.955677 | 1.643989 | 2.20E-33 | 3.51E-31 |
| LINC01671 | 0.293058 | 0.930262 | 1.666452 | 0.011608 | 0.019102 |
| LHX6 | 0.540728 | 1.199203 | 1.149101 | 2.38E-28 | 1.25E-26 |
| CLIC4 | 26.91344 | 56.53336 | 1.070776 | 1.81E-24 | 5.54E-23 |
| TBKBP1 | 2.169619 | 3.519031 | 0.697737 | 4.25E-15 | 4.79E-14 |
| AC105942.1 | 1.966066 | 3.616286 | 0.879197 | 8.11E-16 | 9.81E-15 |
| CAP2 | 2.012636 | 5.794496 | 1.525597 | 4.28E-17 | 5.91E-16 |
| C1QL1 | 1.124702 | 3.011488 | 1.420934 | 4.85E-15 | 5.43E-14 |
| STUM | 0.228768 | 1.004622 | 2.134697 | 2.65E-25 | 9.07E-24 |
| TRAV12-1 | 0.84294 | 1.455464 | 0.787977 | 4.76E-07 | 1.77E-06 |
| RPL34P26 | 1.288124 | 0.710074 | -0.85923 | 0.000269 | 0.000617 |
| RNU6-529P | 1.225667 | 1.999504 | 0.706074 | 3.11E-05 | 8.46E-05 |
| JPH2 | 1.102237 | 6.415164 | 2.541052 | 6.54E-21 | 1.33E-19 |
| IGLV7-43 | 11.75302 | 45.62653 | 1.956841 | 1.60E-06 | 5.45E-06 |
| IL20RA | 4.543917 | 2.514938 | -0.85341 | 3.91E-13 | 3.58E-12 |
| WASF3 | 1.122657 | 2.668443 | 1.249081 | 6.77E-22 | 1.57E-20 |
| WWC3 | 5.168143 | 8.167101 | 0.660178 | 4.46E-17 | 6.13E-16 |
| ASCL2 | 28.99762 | 16.32269 | -0.82906 | 0.026438 | 0.040219 |
| CHI3L2 | 1.092294 | 1.91238 | 0.808008 | 4.52E-10 | 2.74E-09 |
| NAALADL1 | 0.962665 | 1.499514 | 0.639389 | 4.39E-14 | 4.42E-13 |
| AC104041.1 | 0.453092 | 0.740531 | 0.708756 | 0.00912 | 0.015362 |
| ASB5 | 0.212699 | 1.089399 | 2.356646 | 5.75E-07 | 2.11E-06 |
| PADI1 | 1.314083 | 0.280724 | -2.22683 | 0.001816 | 0.003542 |
| CXorf67 | 0.339938 | 3.358259 | 3.304369 | 1.70E-08 | 8.04E-08 |
| UBE2FP1 | 1.379349 | 0.855486 | -0.68917 | 0.0011 | 0.002245 |
| PLA2G12B | 0.821415 | 1.795642 | 1.128317 | 0.021202 | 0.032991 |
| CACNA2D1 | 1.418168 | 3.322276 | 1.228143 | 7.94E-22 | 1.82E-20 |
| TAC1 | 0.217845 | 0.97315 | 2.15936 | 1.43E-12 | 1.21E-11 |
| NRXN2 | 0.264566 | 1.083309 | 2.033747 | 8.72E-25 | 2.78E-23 |
| AC069213.3 | 1.473775 | 0.952252 | -0.6301 | 5.17E-05 | 0.000135 |
| CLGN | 0.523369 | 0.856916 | 0.711326 | 9.09E-10 | 5.26E-09 |
| GZMK | 1.977745 | 3.584813 | 0.858042 | 4.14E-08 | 1.83E-07 |
| SERPINB1 | 92.7605 | 59.89043 | -0.63119 | 4.24E-11 | 3.00E-10 |
| STON1 | 0.888248 | 3.389782 | 1.932159 | 2.06E-29 | 1.35E-27 |
| GATA5 | 3.259207 | 8.054822 | 1.305332 | 5.48E-05 | 0.000143 |
| MARVELD1 | 12.74363 | 23.56055 | 0.886597 | 2.71E-20 | 5.30E-19 |
| SYNC | 0.582226 | 2.74346 | 2.236346 | 1.08E-26 | 4.45E-25 |
| ILK | 2.398174 | 4.256538 | 0.827744 | 6.30E-17 | 8.52E-16 |
| PHLDA2 | 99.3322 | 62.46142 | -0.6693 | 3.16E-11 | 2.27E-10 |
| AL669983.1 | 3.366697 | 2.066607 | -0.70407 | 1.01E-10 | 6.76E-10 |
| LINC02544 | 0.389886 | 0.720413 | 0.88577 | 3.69E-06 | 1.18E-05 |
| IGHV3-21 | 97.0494 | 154.8442 | 0.674026 | 1.56E-06 | 5.31E-06 |
| AP001086.1 | 1.245652 | 0.811931 | -0.61747 | 2.72E-08 | 1.24E-07 |
| IGHV4-31 | 26.23586 | 60.49278 | 1.205223 | 4.81E-09 | 2.50E-08 |
| GPR15 | 0.379006 | 1.047601 | 1.466795 | 1.36E-08 | 6.55E-08 |
| KCNIP3 | 0.32505 | 0.759911 | 1.225169 | 1.11E-20 | 2.23E-19 |
| PLPPR4 | 0.530068 | 1.570331 | 1.566819 | 9.44E-21 | 1.90E-19 |
| EVI2B | 11.0103 | 17.86481 | 0.698268 | 9.54E-10 | 5.51E-09 |
| PGBD1 | 0.906097 | 1.447544 | 0.675869 | 3.59E-14 | 3.67E-13 |
| RPL23AP12 | 2.085763 | 1.295723 | -0.68682 | 0.00535 | 0.009469 |
| PLA2G5 | 0.320831 | 0.879114 | 1.454237 | 2.71E-12 | 2.23E-11 |
| KLHDC8B | 3.626496 | 6.13083 | 0.757506 | 6.72E-20 | 1.27E-18 |
| KLHL6 | 1.084127 | 1.811534 | 0.740678 | 1.14E-10 | 7.59E-10 |
| CBLC | 45.47893 | 29.94118 | -0.60307 | 5.21E-06 | 1.62E-05 |
| FCER1A | 0.483425 | 2.432064 | 2.330818 | 2.30E-22 | 5.65E-21 |
| PRSS3 | 54.33771 | 32.70865 | -0.73228 | 1.18E-10 | 7.85E-10 |
| LTBP2 | 8.108894 | 22.14366 | 1.449317 | 2.96E-31 | 2.90E-29 |
| CD274 | 3.70337 | 1.478011 | -1.32518 | 0.023086 | 0.035583 |
| ENG | 26.41548 | 50.40225 | 0.932105 | 2.05E-28 | 1.09E-26 |
| TRBV2 | 0.827838 | 1.256101 | 0.601531 | 2.69E-07 | 1.04E-06 |
| GSTM5 | 0.246792 | 0.846854 | 1.778816 | 1.36E-28 | 7.39E-27 |
| TSPYL2 | 2.654664 | 4.287459 | 0.691594 | 2.89E-15 | 3.28E-14 |
| GPC6 | 2.132189 | 5.9301 | 1.475721 | 6.37E-33 | 8.92E-31 |
| LAX1 | 1.20171 | 2.168454 | 0.851578 | 1.28E-10 | 8.44E-10 |
| TSPY26P | 0.987607 | 2.147422 | 1.120597 | 1.53E-21 | 3.37E-20 |
| PI15 | 0.803726 | 2.917541 | 1.859977 | 2.52E-09 | 1.38E-08 |
| HEYL | 4.919972 | 13.49003 | 1.455172 | 6.07E-28 | 3.00E-26 |
| IGKV1D-39 | 6.585143 | 17.82685 | 1.436765 | 1.12E-09 | 6.45E-09 |
| IGHV2-5 | 9.298975 | 21.47452 | 1.207483 | 1.15E-08 | 5.64E-08 |
| CD19 | 1.566841 | 2.372851 | 0.598762 | 3.46E-08 | 1.55E-07 |
| CDC42EP1 | 147.838 | 98.11154 | -0.59152 | 1.58E-07 | 6.36E-07 |
| ZNF570 | 0.534955 | 0.936049 | 0.807168 | 4.90E-18 | 7.45E-17 |
| SLC9A9 | 1.017065 | 2.276319 | 1.162291 | 2.02E-24 | 6.11E-23 |
| KLHDC1 | 0.792608 | 1.201289 | 0.599904 | 1.46E-11 | 1.10E-10 |
| OLFML3 | 6.854524 | 19.5029 | 1.508561 | 4.06E-32 | 4.56E-30 |
| OSR1 | 0.49849 | 0.942304 | 0.918628 | 1.14E-10 | 7.59E-10 |
| PITX1 | 27.39011 | 17.51211 | -0.6453 | 0.00246 | 0.00468 |
| IGLV2-23 | 83.84859 | 185.4244 | 1.144972 | 4.75E-08 | 2.07E-07 |
| SLC1A5 | 60.11416 | 36.45442 | -0.72161 | 6.45E-10 | 3.81E-09 |
| SIGLEC1 | 2.109608 | 3.512226 | 0.735411 | 4.45E-08 | 1.95E-07 |
| KLK5 | 5.408725 | 0.27062 | -4.32095 | 0.012572 | 0.020555 |
| FADS2 | 3.644014 | 6.282653 | 0.785845 | 2.43E-18 | 3.82E-17 |
| ZBTB47 | 3.470334 | 7.150541 | 1.042978 | 1.35E-25 | 4.91E-24 |
| TRPS1 | 1.365853 | 2.337384 | 0.775093 | 5.32E-15 | 5.94E-14 |
| CRYAB | 3.990846 | 11.97976 | 1.585832 | 1.19E-18 | 1.93E-17 |
| RBMS1P1 | 0.38369 | 0.645293 | 0.750012 | 4.98E-14 | 5.00E-13 |
| AL365356.4 | 0.747566 | 0.327336 | -1.19143 | 9.62E-06 | 2.85E-05 |
| AC087588.2 | 1.700384 | 0.936391 | -0.86068 | 7.26E-09 | 3.67E-08 |
| TMEM191A | 1.190083 | 0.76499 | -0.63755 | 8.56E-07 | 3.05E-06 |
| MKI67 | 24.17163 | 15.94273 | -0.60042 | 9.17E-11 | 6.18E-10 |
| LOXL1 | 8.205175 | 15.68406 | 0.934693 | 3.40E-21 | 7.15E-20 |
| MIEN1 | 98.75105 | 58.24167 | -0.76174 | 0.000188 | 0.000442 |
| THSD7A | 0.34606 | 0.838107 | 1.276112 | 6.24E-27 | 2.69E-25 |
| AC093840.1 | 1.811756 | 1.162412 | -0.64027 | 4.12E-08 | 1.82E-07 |
| MIR6835 | 1.197499 | 0.795647 | -0.58982 | 0.00018 | 0.000425 |
| IGHV3-23 | 174.487 | 283.5422 | 0.700444 | 2.68E-07 | 1.04E-06 |
| GIMAP4 | 9.705872 | 14.60099 | 0.589137 | 1.23E-12 | 1.06E-11 |
| ISG15 | 99.30759 | 64.20205 | -0.62928 | 0.0011 | 0.002245 |
| SYNPO2 | 5.501387 | 46.75414 | 3.087227 | 1.28E-24 | 4.00E-23 |
| GC | 2.906711 | 17.89076 | 2.621755 | 7.72E-09 | 3.88E-08 |
| TRBJ2-2 | 0.679823 | 1.225195 | 0.849779 | 2.01E-05 | 5.64E-05 |
| SERPINB3 | 8.17999 | 1.352423 | -2.59655 | 0.003651 | 0.006696 |
| TRBV29-1 | 1.514568 | 2.39423 | 0.660656 | 3.12E-08 | 1.41E-07 |
| NEUROG3 | 1.085704 | 1.784103 | 0.716568 | 0.000943 | 0.001955 |
| BACH2 | 0.362223 | 0.743914 | 1.038259 | 5.65E-19 | 9.46E-18 |
| TRBJ2-7 | 1.877543 | 3.0509 | 0.700389 | 4.02E-05 | 0.000107 |
| IGHV3-62 | 0.933032 | 3.242153 | 1.796953 | 2.60E-09 | 1.41E-08 |
| AF131215.5 | 1.012641 | 1.603706 | 0.663287 | 2.15E-06 | 7.15E-06 |
| NPR1 | 0.848037 | 2.463046 | 1.538245 | 4.28E-33 | 6.31E-31 |
| MIR938 | 0.589467 | 1.01008 | 0.776987 | 0.001198 | 0.002425 |
| RCAN2 | 3.073323 | 10.85399 | 1.820353 | 1.77E-36 | 7.50E-34 |
| AC079921.2 | 0.498942 | 0.931044 | 0.899977 | 8.85E-14 | 8.71E-13 |
| CH25H | 1.378371 | 4.155858 | 1.592182 | 2.28E-32 | 2.74E-30 |
| AFAP1L1 | 1.536888 | 2.844195 | 0.888008 | 9.55E-26 | 3.56E-24 |
| IGLV10-54 | 16.6855 | 32.18136 | 0.94763 | 5.38E-08 | 2.33E-07 |
| RNF144A | 2.432175 | 4.593504 | 0.917348 | 2.30E-21 | 4.93E-20 |
| GPR161 | 0.981708 | 1.979689 | 1.011908 | 1.03E-24 | 3.27E-23 |
| DLC1 | 2.331368 | 5.749739 | 1.30232 | 2.95E-35 | 8.44E-33 |
| ADGRF1 | 1.582383 | 0.654337 | -1.27399 | 3.67E-05 | 9.86E-05 |
| SBSPON | 1.731459 | 2.874119 | 0.731131 | 2.79E-09 | 1.51E-08 |
| PPP1R14A | 4.517029 | 15.67247 | 1.794787 | 1.62E-23 | 4.47E-22 |
| CTSG | 0.359381 | 1.783732 | 2.311313 | 2.80E-21 | 5.94E-20 |
| TTC3P1 | 0.677133 | 1.08358 | 0.678294 | 1.74E-12 | 1.46E-11 |
| HTRA3 | 15.43417 | 25.33205 | 0.714836 | 3.18E-11 | 2.28E-10 |
| STOX2 | 0.423271 | 0.838546 | 0.986309 | 2.02E-19 | 3.57E-18 |
| DIP2C | 2.701746 | 4.498279 | 0.735481 | 1.02E-16 | 1.35E-15 |
| IGLV3-27 | 10.54588 | 32.53914 | 1.625496 | 2.09E-06 | 6.97E-06 |
| COLEC11 | 0.39997 | 0.97929 | 1.291845 | 1.08E-12 | 9.36E-12 |
| AC023794.3 | 0.459922 | 0.76239 | 0.72914 | 1.72E-09 | 9.60E-09 |
| MAD2L1 | 7.501735 | 4.542932 | -0.7236 | 6.15E-16 | 7.52E-15 |
| HABP2 | 6.850255 | 10.76833 | 0.652565 | 5.33E-07 | 1.96E-06 |
| CES1 | 5.187839 | 9.263792 | 0.836469 | 5.66E-18 | 8.52E-17 |
| AC112491.1 | 49.78009 | 33.04871 | -0.59097 | 3.33E-05 | 9.02E-05 |
| PKD1 | 3.981382 | 7.279517 | 0.870573 | 1.02E-13 | 9.95E-13 |
| NFATC4 | 1.039363 | 2.516011 | 1.275439 | 1.47E-28 | 7.92E-27 |
| AL136084.3 | 1.402562 | 4.195831 | 1.580892 | 1.04E-19 | 1.90E-18 |
| AC024995.1 | 6.261192 | 3.824048 | -0.71134 | 3.71E-06 | 1.19E-05 |
| SNHG15 | 5.471078 | 3.611877 | -0.59908 | 3.32E-16 | 4.19E-15 |
| PBXIP1 | 18.24437 | 32.69358 | 0.841556 | 5.26E-26 | 2.04E-24 |
| PCDHB4 | 0.478714 | 1.132627 | 1.242436 | 1.01E-29 | 7.06E-28 |
| ZNF354C | 0.353905 | 0.83059 | 1.230772 | 2.96E-27 | 1.31E-25 |
| RAB23 | 2.190198 | 5.69824 | 1.379455 | 4.80E-16 | 5.96E-15 |
| GEM | 10.73263 | 24.30001 | 1.178954 | 4.74E-21 | 9.84E-20 |
| ZNF329 | 1.58387 | 2.382611 | 0.589089 | 1.98E-10 | 1.27E-09 |
| SELL | 5.49036 | 9.880357 | 0.847662 | 1.14E-10 | 7.59E-10 |
| CHST10 | 0.838521 | 1.52327 | 0.861253 | 1.13E-21 | 2.54E-20 |
| NID1 | 19.61654 | 43.1275 | 1.136538 | 1.04E-34 | 2.30E-32 |
| AC090559.1 | 0.700983 | 1.451177 | 1.049773 | 5.62E-17 | 7.62E-16 |
| CLIP4 | 1.355718 | 2.493785 | 0.87928 | 6.65E-18 | 9.92E-17 |
| DPEP3 | 0.081736 | 1.57561 | 4.268799 | 1.65E-07 | 6.60E-07 |
| FRAS1 | 1.261871 | 2.299675 | 0.865866 | 0.001886 | 0.003665 |
| VGLL3 | 0.688634 | 2.036148 | 1.564034 | 5.05E-21 | 1.04E-19 |
| NLRP3 | 0.483765 | 0.99215 | 1.036251 | 1.83E-16 | 2.38E-15 |
| HOXA9 | 2.545434 | 0.876601 | -1.53792 | 0.006834 | 0.011825 |
| CD36 | 0.902264 | 2.663846 | 1.561889 | 1.36E-24 | 4.20E-23 |
| THY1 | 14.62739 | 30.062 | 1.039269 | 2.99E-18 | 4.66E-17 |
| PCAT19 | 0.822439 | 1.415929 | 0.783769 | 9.43E-20 | 1.74E-18 |
| NUMBL | 3.752744 | 5.649707 | 0.59023 | 2.75E-19 | 4.77E-18 |
| MYOC | 0.547565 | 2.549437 | 2.219078 | 9.87E-12 | 7.59E-11 |
| TENM4 | 0.421119 | 0.891107 | 1.081373 | 1.19E-18 | 1.93E-17 |
| SPRR3 | 213.9392 | 4.966148 | -5.42893 | 2.85E-05 | 7.81E-05 |
| AC102945.2 | 0.63474 | 1.506996 | 1.247437 | 9.34E-17 | 1.24E-15 |
| RPS15P5 | 2.069678 | 1.330552 | -0.63738 | 6.38E-07 | 2.32E-06 |
| BCL2 | 1.400823 | 2.344554 | 0.743039 | 3.47E-11 | 2.48E-10 |
| FOXC1 | 10.69813 | 6.936834 | -0.62501 | 0.010987 | 0.01818 |
| RPL23AP75 | 1.175512 | 0.766217 | -0.61746 | 5.69E-06 | 1.75E-05 |
| ACTG2 | 51.62726 | 404.3061 | 2.969243 | 6.05E-17 | 8.18E-16 |
| AFF3 | 0.238029 | 0.772657 | 1.698691 | 3.71E-23 | 9.72E-22 |
| TFAP2A | 4.656039 | 2.175885 | -1.0975 | 1.17E-12 | 1.00E-11 |
| DPYSL4 | 0.389355 | 0.654739 | 0.749833 | 8.60E-17 | 1.15E-15 |
| KIAA0513 | 2.30285 | 3.660709 | 0.668703 | 7.27E-13 | 6.41E-12 |
| SERPINA4 | 5.3048 | 8.697901 | 0.713369 | 0.000109 | 0.000268 |
| RNU6-1176P | 0.553457 | 1.215241 | 1.134698 | 4.52E-07 | 1.68E-06 |
| PYGO1 | 0.55499 | 1.300916 | 1.228995 | 1.49E-27 | 7.00E-26 |
| NFASC | 0.595966 | 2.392326 | 2.005112 | 2.86E-28 | 1.48E-26 |
| UGT2B17 | 0.873901 | 2.788945 | 1.674178 | 0.013777 | 0.022337 |
| TMEM25 | 1.716397 | 2.579082 | 0.587474 | 2.36E-09 | 1.29E-08 |
| CLEC2L | 0.441788 | 0.850396 | 0.944781 | 1.34E-14 | 1.45E-13 |
| CLIC6 | 5.687086 | 8.552412 | 0.588642 | 4.81E-09 | 2.50E-08 |
| OBSCN | 0.619266 | 0.951456 | 0.619577 | 5.10E-08 | 2.21E-07 |
| AC245128.3 | 0.412663 | 0.761791 | 0.88443 | 0.011451 | 0.018869 |
| MAF | 5.011702 | 9.146036 | 0.867846 | 3.16E-24 | 9.38E-23 |
| IKZF1 | 1.949812 | 3.654085 | 0.906175 | 9.78E-13 | 8.52E-12 |
| DNAJB1 | 56.60607 | 36.89342 | -0.61759 | 3.25E-09 | 1.73E-08 |
| ORAI2 | 2.387377 | 3.622551 | 0.60158 | 8.49E-15 | 9.28E-14 |
| LINC00654 | 0.571964 | 1.007508 | 0.816795 | 3.40E-17 | 4.73E-16 |
| PHACTR1 | 0.59542 | 0.966248 | 0.698485 | 9.24E-18 | 1.36E-16 |
| PCDH7 | 3.454692 | 6.247733 | 0.854776 | 2.07E-10 | 1.32E-09 |
| COL4A5 | 1.37795 | 3.239661 | 1.23332 | 8.14E-19 | 1.34E-17 |
| MIR4482 | 1.100099 | 1.703945 | 0.631245 | 6.03E-05 | 0.000156 |
| ALPL | 2.56959 | 5.692233 | 1.147456 | 9.63E-18 | 1.41E-16 |
| TLR1 | 1.306288 | 2.202832 | 0.753887 | 1.06E-11 | 8.10E-11 |
| S100A14 | 122.4153 | 75.35723 | -0.69997 | 0.000374 | 0.000834 |
| SAMD4A | 1.362672 | 2.867255 | 1.073232 | 8.11E-16 | 9.81E-15 |
| IGLV2-18 | 9.396766 | 15.76396 | 0.746394 | 4.35E-08 | 1.91E-07 |
| CCDC3 | 5.461939 | 11.11225 | 1.024665 | 8.05E-25 | 2.58E-23 |
| SERPINB13 | 1.54815 | 0.028936 | -5.74153 | 0.008294 | 0.0141 |
| ECM2 | 1.494163 | 3.418769 | 1.19414 | 6.51E-27 | 2.78E-25 |
| AC254633.1 | 1.005101 | 1.602465 | 0.672952 | 4.74E-11 | 3.34E-10 |
| CRIP1 | 3.691069 | 2.365315 | -0.64201 | 0.006834 | 0.011825 |
| GPR4 | 2.941985 | 4.520624 | 0.619732 | 2.58E-12 | 2.13E-11 |
| AC011450.1 | 0.450002 | 0.793811 | 0.818865 | 5.24E-13 | 4.71E-12 |
| SNRPF | 25.37443 | 16.8415 | -0.59136 | 1.55E-13 | 1.49E-12 |
| C1QTNF7 | 0.253304 | 1.370072 | 2.435308 | 2.01E-34 | 4.06E-32 |
| TTLL7 | 0.723233 | 1.538494 | 1.088986 | 8.89E-15 | 9.69E-14 |
| MIR210HG | 1.888366 | 1.145521 | -0.72113 | 1.46E-08 | 6.98E-08 |
| WNT2B | 0.552749 | 1.622758 | 1.553751 | 4.43E-22 | 1.05E-20 |
| IGHV4-28 | 8.823625 | 20.58882 | 1.222417 | 2.96E-08 | 1.34E-07 |
| TNFSF8 | 0.670554 | 1.411827 | 1.074137 | 3.82E-15 | 4.32E-14 |
| ZNF25 | 1.797962 | 3.048956 | 0.761953 | 7.79E-19 | 1.29E-17 |
| RBM24 | 0.285253 | 0.845583 | 1.567704 | 1.18E-17 | 1.71E-16 |
| PLPP1 | 12.10227 | 24.40157 | 1.011696 | 8.08E-32 | 8.73E-30 |
| MID2 | 1.606599 | 3.370017 | 1.068746 | 4.77E-22 | 1.13E-20 |
| STC1 | 8.496025 | 13.29083 | 0.645571 | 1.66E-06 | 5.62E-06 |
| DOK7 | 1.734393 | 0.986065 | -0.81468 | 0.032017 | 0.047818 |
| IGKV1-33 | 2.897423 | 8.043504 | 1.473054 | 4.96E-10 | 2.99E-09 |
| IGLV2-8 | 25.37857 | 61.34609 | 1.273361 | 3.29E-07 | 1.26E-06 |
| FZD7 | 5.322436 | 11.40955 | 1.100083 | 2.98E-17 | 4.16E-16 |
| ATP1A2 | 0.380494 | 2.793197 | 2.875973 | 2.09E-23 | 5.68E-22 |
| ELOVL4 | 0.410581 | 0.757304 | 0.883207 | 1.44E-25 | 5.21E-24 |
| MAMLD1 | 0.8589 | 1.68413 | 0.971441 | 1.78E-24 | 5.45E-23 |
| ARMCX1 | 1.920179 | 4.62076 | 1.266889 | 7.32E-29 | 4.28E-27 |
| MDFIC | 3.476284 | 6.226903 | 0.840969 | 2.78E-22 | 6.75E-21 |
| IGHV4-39 | 115.4968 | 248.8435 | 1.107386 | 7.11E-06 | 2.16E-05 |
| AC015912.3 | 3.759295 | 2.320176 | -0.69623 | 4.23E-12 | 3.40E-11 |
| ITGA4 | 1.896615 | 3.430262 | 0.854892 | 4.29E-15 | 4.82E-14 |
| PRRG4 | 10.1746 | 6.297469 | -0.69213 | 6.03E-12 | 4.76E-11 |
| MYEOV | 13.96405 | 8.911006 | -0.64806 | 4.49E-06 | 1.41E-05 |
| AC026410.2 | 1.021779 | 0.672785 | -0.60287 | 0.000575 | 0.001243 |
| KLK10 | 23.87514 | 15.57759 | -0.61604 | 0.00346 | 0.006372 |
| IGKV1D-16 | 4.615003 | 16.97832 | 1.87929 | 1.26E-08 | 6.12E-08 |
| PAX9 | 1.664413 | 0.403826 | -2.04321 | 2.98E-06 | 9.68E-06 |
| AC087343.1 | 3.155659 | 1.799115 | -0.81065 | 1.94E-06 | 6.52E-06 |
| SLC9A3-AS1 | 2.904294 | 1.858713 | -0.64388 | 0.032095 | 0.047923 |
| AGPAT4 | 0.715622 | 1.141636 | 0.673833 | 1.81E-17 | 2.57E-16 |
| XPNPEP2 | 2.309268 | 5.393451 | 1.223773 | 3.62E-13 | 3.32E-12 |
| KLHL5 | 5.293704 | 9.455247 | 0.836838 | 7.57E-22 | 1.75E-20 |
| TRBV5-1 | 0.895828 | 1.516635 | 0.75958 | 3.20E-08 | 1.44E-07 |
| MPEG1 | 9.334446 | 15.26159 | 0.709269 | 3.54E-13 | 3.26E-12 |
| PLXDC1 | 1.616734 | 3.130477 | 0.9533 | 1.07E-25 | 3.93E-24 |
| SERPINB4 | 2.476274 | 0.529312 | -2.22598 | 0.001645 | 0.003238 |
| SNAI2 | 4.001496 | 6.939219 | 0.794234 | 5.89E-10 | 3.50E-09 |
| TIMP2 | 34.6258 | 79.71848 | 1.203067 | 2.38E-30 | 1.90E-28 |
| MAPK10 | 0.241164 | 0.847284 | 1.812828 | 3.54E-35 | 9.83E-33 |
| APOD | 19.25686 | 81.31701 | 2.078185 | 1.10E-27 | 5.27E-26 |
| AQP1 | 41.42635 | 63.95774 | 0.62657 | 3.13E-12 | 2.55E-11 |
| MAP9 | 0.739617 | 1.589457 | 1.103683 | 6.13E-21 | 1.26E-19 |
| PERP | 97.54708 | 52.70118 | -0.88826 | 3.78E-14 | 3.86E-13 |
| IGHV3-66 | 6.860944 | 17.59108 | 1.358365 | 2.56E-08 | 1.18E-07 |
| AC124312.2 | 0.270543 | 0.752497 | 1.475828 | 4.07E-15 | 4.60E-14 |
| CLDN6 | 6.871688 | 10.72367 | 0.642063 | 0.00829 | 0.014097 |
| KCNN3 | 0.77563 | 1.320268 | 0.767389 | 1.15E-31 | 1.21E-29 |
| CHGA | 9.408933 | 42.15555 | 2.16362 | 2.40E-08 | 1.10E-07 |
| RPL41P1 | 33.31412 | 20.20613 | -0.72134 | 0.000371 | 0.000829 |
| COLEC12 | 0.819219 | 2.595458 | 1.663668 | 2.99E-27 | 1.32E-25 |
| STK32B | 0.377608 | 0.665319 | 0.817156 | 2.47E-23 | 6.65E-22 |
| FMOD | 20.09411 | 54.67354 | 1.44407 | 3.09E-29 | 1.96E-27 |
| PARD3B | 1.969443 | 2.993242 | 0.603921 | 3.92E-14 | 3.98E-13 |
| RFX6 | 0.198368 | 0.986193 | 2.313688 | 3.75E-07 | 1.42E-06 |
| GSTM2 | 0.557411 | 1.156682 | 1.053179 | 1.81E-21 | 3.93E-20 |
| LINC00540 | 0.407446 | 0.617692 | 0.600277 | 0.004828 | 0.008627 |
| PDZK1IP1 | 106.5404 | 60.90835 | -0.80669 | 2.89E-07 | 1.12E-06 |
| LATS2 | 5.868155 | 8.966311 | 0.611608 | 1.79E-19 | 3.18E-18 |
| AC016747.1 | 1.100823 | 1.761138 | 0.677925 | 3.72E-14 | 3.81E-13 |
| LMO3 | 0.249443 | 0.792267 | 1.667273 | 2.57E-21 | 5.49E-20 |
| CD248 | 12.97177 | 24.53939 | 0.919725 | 1.21E-14 | 1.31E-13 |
| CDC25A | 3.646284 | 2.198208 | -0.7301 | 4.45E-14 | 4.49E-13 |
| RNA5SP530 | 0.562988 | 0.971429 | 0.787005 | 0.007276 | 0.012512 |
| SAPCD2 | 18.43981 | 10.80905 | -0.77058 | 3.84E-14 | 3.91E-13 |
| CYS1 | 0.984313 | 4.300057 | 2.127167 | 5.06E-27 | 2.20E-25 |
| GGTA1P | 0.72646 | 1.420681 | 0.967627 | 1.03E-16 | 1.36E-15 |
| H2AFY2 | 4.847882 | 8.126034 | 0.745197 | 6.40E-14 | 6.38E-13 |
| ATP6V1B1 | 0.573944 | 0.874782 | 0.608015 | 0.000358 | 0.000801 |
| PRSS23 | 5.700726 | 11.19822 | 0.974052 | 2.50E-20 | 4.90E-19 |
| CLC | 0.788788 | 2.679185 | 1.764086 | 3.63E-07 | 1.38E-06 |
| TRAV17 | 0.73731 | 1.259177 | 0.772136 | 3.00E-05 | 8.17E-05 |
| SCT | 1.265137 | 3.966057 | 1.648411 | 2.38E-08 | 1.10E-07 |
| LRCH2 | 0.349787 | 1.019305 | 1.543038 | 1.07E-36 | 5.18E-34 |
| CBX6 | 5.517188 | 11.38681 | 1.045359 | 2.71E-19 | 4.69E-18 |
| FAM198B-AS1 | 0.507995 | 0.885433 | 0.801569 | 5.04E-16 | 6.22E-15 |
| COL14A1 | 3.117633 | 17.15721 | 2.460292 | 6.97E-36 | 2.41E-33 |
| MIR100HG | 0.927998 | 3.429956 | 1.885997 | 1.85E-24 | 5.64E-23 |
| GTSF1 | 1.856372 | 3.840249 | 1.048714 | 7.83E-07 | 2.81E-06 |
| LEPR | 0.79524 | 1.874224 | 1.236832 | 3.99E-20 | 7.67E-19 |
| IGKV1-5 | 186.393 | 354.8627 | 0.928913 | 5.22E-07 | 1.92E-06 |
| FAM49A | 1.448938 | 2.96621 | 1.033624 | 1.31E-23 | 3.63E-22 |
| CPXM2 | 2.342864 | 10.97968 | 2.228491 | 1.80E-25 | 6.38E-24 |
| LYAR | 14.60536 | 9.69237 | -0.59158 | 6.63E-13 | 5.86E-12 |
| SLC16A4 | 2.149107 | 3.346001 | 0.6387 | 5.90E-14 | 5.89E-13 |
| HIST1H2AJ | 1.010176 | 0.585636 | -0.78653 | 2.68E-08 | 1.22E-07 |
| CALCOCO1 | 5.474037 | 8.22856 | 0.588035 | 2.59E-16 | 3.31E-15 |
| ABCC6P2 | 0.696218 | 1.173886 | 0.753682 | 0.002329 | 0.004453 |
| LRRC37A17P | 0.784552 | 1.272383 | 0.697592 | 4.38E-12 | 3.51E-11 |
| H3F3AP6 | 9.333554 | 3.422143 | -1.44753 | 2.57E-06 | 8.45E-06 |
| CD1D | 0.731889 | 1.482146 | 1.01799 | 3.46E-20 | 6.70E-19 |
| COL4A1 | 56.82002 | 95.69794 | 0.752089 | 2.30E-16 | 2.95E-15 |
| APLNR | 7.078452 | 16.78452 | 1.245625 | 6.64E-24 | 1.90E-22 |
| IL6ST | 13.01294 | 24.21077 | 0.895702 | 5.38E-25 | 1.76E-23 |
| SVIL | 8.695742 | 23.87208 | 1.456943 | 1.26E-14 | 1.36E-13 |
| RPL7AP50 | 6.744068 | 3.857146 | -0.80609 | 3.44E-12 | 2.80E-11 |
| MILR1 | 1.74946 | 2.799108 | 0.678058 | 1.19E-13 | 1.15E-12 |
| AC239859.6 | 0.998347 | 1.887904 | 0.919172 | 4.06E-11 | 2.87E-10 |
| TSHZ2 | 1.571971 | 2.882074 | 0.874533 | 6.84E-19 | 1.14E-17 |
| AP001528.2 | 0.272887 | 0.813582 | 1.575982 | 3.55E-31 | 3.42E-29 |
| C4B | 1.326944 | 3.832264 | 1.53009 | 1.51E-23 | 4.19E-22 |
| FN1 | 62.95046 | 131.1768 | 1.059224 | 1.06E-12 | 9.18E-12 |
| WNT5A | 3.94011 | 6.521326 | 0.72693 | 7.47E-05 | 0.00019 |
| IRF4 | 1.29304 | 2.102809 | 0.701551 | 4.95E-09 | 2.57E-08 |
| HDGFL3 | 0.869821 | 1.831871 | 1.074528 | 1.13E-28 | 6.29E-27 |
| SIGLEC7 | 0.906592 | 1.394124 | 0.620834 | 7.29E-08 | 3.10E-07 |
| SCN4B | 0.286422 | 1.230875 | 2.103471 | 1.12E-36 | 5.29E-34 |
| LRRC4B | 0.701968 | 1.803354 | 1.361206 | 5.54E-25 | 1.82E-23 |
| CPVL | 9.645594 | 14.58406 | 0.59645 | 1.70E-09 | 9.50E-09 |
| RPS26P6 | 2.422833 | 1.587505 | -0.60993 | 0.00176 | 0.003443 |
| CYR61 | 28.03467 | 62.79974 | 1.163547 | 1.69E-15 | 1.98E-14 |
| C11orf95 | 3.183476 | 4.986558 | 0.647442 | 3.37E-13 | 3.11E-12 |
| PSAPL1 | 1.693755 | 4.736474 | 1.483588 | 3.72E-06 | 1.19E-05 |
| CDH11 | 3.962048 | 9.576169 | 1.273202 | 7.08E-27 | 3.00E-25 |
| FBN2 | 0.422174 | 0.712419 | 0.754887 | 1.19E-12 | 1.02E-11 |
| COL18A1 | 19.6496 | 33.58468 | 0.773303 | 5.70E-15 | 6.35E-14 |
| PLPPR2 | 8.09021 | 12.13749 | 0.585221 | 1.33E-12 | 1.14E-11 |
| MIF | 98.50356 | 63.08174 | -0.64295 | 3.04E-11 | 2.19E-10 |
| ANKRD33B | 0.475954 | 0.769235 | 0.692601 | 2.46E-19 | 4.27E-18 |
| GABARAPL1 | 5.863406 | 10.50762 | 0.841625 | 4.16E-21 | 8.72E-20 |
| ADGRA2 | 5.499799 | 15.09016 | 1.456157 | 2.69E-36 | 1.08E-33 |
| EDARADD | 0.507933 | 0.772273 | 0.604474 | 6.59E-05 | 0.000169 |
| AL356356.1 | 0.64784 | 1.077682 | 0.734222 | 1.66E-07 | 6.66E-07 |
| GPR20 | 0.371907 | 0.99463 | 1.419217 | 4.80E-27 | 2.09E-25 |
| ADPRH | 1.675039 | 2.652243 | 0.663018 | 7.31E-27 | 3.09E-25 |
| KCND3 | 2.232627 | 3.411179 | 0.611528 | 1.60E-08 | 7.62E-08 |
| CARTPT | 0.516266 | 2.78095 | 2.429393 | 2.95E-07 | 1.14E-06 |
| MIR378H | 0.792636 | 1.331077 | 0.747864 | 9.52E-05 | 0.000237 |
| MAMDC2 | 0.643325 | 2.567155 | 1.99655 | 1.16E-23 | 3.24E-22 |
| PRKCB | 1.568443 | 3.764326 | 1.263059 | 8.85E-16 | 1.06E-14 |
| IGHV4-55 | 4.608653 | 12.09432 | 1.391912 | 7.64E-09 | 3.85E-08 |
| AC006122.1 | 1.868411 | 1.199118 | -0.63984 | 1.70E-07 | 6.80E-07 |
| TGFBI | 22.72694 | 37.61787 | 0.727015 | 4.24E-11 | 3.00E-10 |
| CFH | 6.110483 | 12.13137 | 0.989384 | 1.88E-19 | 3.34E-18 |
| FAM167B | 3.736927 | 5.854116 | 0.647599 | 5.67E-12 | 4.50E-11 |
| RASD1 | 4.173885 | 9.465325 | 1.181261 | 1.35E-09 | 7.65E-09 |
| POMC | 0.357955 | 1.271488 | 1.82867 | 9.96E-16 | 1.19E-14 |
| KCNMA1 | 0.929471 | 5.065525 | 2.44623 | 7.79E-19 | 1.29E-17 |
| RAB34 | 9.646345 | 14.65241 | 0.603083 | 2.35E-13 | 2.21E-12 |
| AL627230.2 | 1.108442 | 0.719966 | -0.62253 | 0.000572 | 0.001237 |
| HIC1 | 1.907986 | 4.716218 | 1.30558 | 1.55E-28 | 8.31E-27 |
| SPART | 2.447171 | 4.894557 | 1.000064 | 1.81E-22 | 4.50E-21 |
| RECK | 0.937009 | 2.678914 | 1.515514 | 4.08E-41 | 2.31E-37 |
| SH3RF3-AS1 | 0.574617 | 1.488823 | 1.3735 | 8.62E-29 | 4.97E-27 |
| SLC45A1 | 0.417335 | 0.926947 | 1.15128 | 3.04E-30 | 2.36E-28 |
| CYCSP55 | 0.761544 | 0.472642 | -0.68818 | 1.33E-07 | 5.42E-07 |
| POLR3G | 1.870134 | 0.873971 | -1.09748 | 4.58E-18 | 6.98E-17 |
| SHISA4 | 3.681162 | 6.570872 | 0.835924 | 2.27E-19 | 3.97E-18 |
| CSF2RA | 1.905196 | 3.026096 | 0.667519 | 6.39E-11 | 4.42E-10 |
| AGTR1 | 0.342665 | 1.08718 | 1.66572 | 3.54E-16 | 4.44E-15 |
| MEI1 | 0.565958 | 0.948255 | 0.74458 | 4.70E-09 | 2.45E-08 |
| AP002387.1 | 8.47633 | 5.560914 | -0.60812 | 8.47E-05 | 0.000213 |
| TEF | 2.87886 | 4.36376 | 0.600074 | 3.02E-11 | 2.18E-10 |
| TNFSF9 | 7.691071 | 3.285221 | -1.22719 | 0.000389 | 0.000866 |
| SHISAL1 | 0.443151 | 2.300876 | 2.376313 | 2.28E-21 | 4.89E-20 |
| FRG1JP | 0.716364 | 1.152243 | 0.685681 | 8.34E-05 | 0.00021 |
| ITGB1BP2 | 0.36823 | 0.712501 | 0.952285 | 2.39E-09 | 1.31E-08 |
| KCNE2 | 1.872473 | 4.1616 | 1.152193 | 5.90E-06 | 1.82E-05 |
| ATP1B2 | 0.604088 | 1.956201 | 1.695223 | 4.06E-30 | 3.06E-28 |
| TRABD2A | 3.857386 | 2.372963 | -0.70093 | 0.008828 | 0.014922 |
| PREX1 | 5.18718 | 9.310094 | 0.843845 | 9.10E-21 | 1.84E-19 |
| FKBP5 | 4.737324 | 7.436097 | 0.650473 | 3.42E-08 | 1.53E-07 |
| ZNF436-AS1 | 0.501304 | 0.792519 | 0.660759 | 5.48E-17 | 7.44E-16 |
| GPR183 | 6.127954 | 12.80535 | 1.063269 | 4.83E-19 | 8.13E-18 |
| IGHV3-33 | 91.07294 | 140.3296 | 0.623725 | 5.84E-08 | 2.52E-07 |
| GFOD1 | 1.345504 | 2.069663 | 0.621249 | 1.58E-06 | 5.37E-06 |
| MANEAL | 8.163538 | 5.404701 | -0.59498 | 4.86E-06 | 1.52E-05 |
| PDE3A | 1.556902 | 4.299418 | 1.465463 | 1.44E-26 | 5.85E-25 |
| IGHV3-41 | 0.661531 | 1.797251 | 1.441911 | 7.35E-12 | 5.73E-11 |
| TPSAB1 | 9.398994 | 18.40551 | 0.969559 | 1.76E-17 | 2.52E-16 |
| THBS3 | 3.516332 | 5.451713 | 0.632638 | 4.28E-18 | 6.55E-17 |
| SULT2B1 | 8.454022 | 2.795858 | -1.59635 | 2.07E-08 | 9.67E-08 |
| MPRIPP1 | 0.420073 | 0.691959 | 0.720046 | 5.63E-16 | 6.91E-15 |
| RAB39A | 0.411635 | 0.657829 | 0.676347 | 2.02E-09 | 1.12E-08 |
| ADRA1D | 0.727629 | 1.288601 | 0.824531 | 3.97E-13 | 3.62E-12 |
| TRIM15 | 10.39145 | 5.360357 | -0.955 | 4.85E-15 | 5.43E-14 |
| RPL31P49 | 2.19328 | 1.429928 | -0.61715 | 1.51E-05 | 4.33E-05 |
| ANGPT1 | 0.44175 | 1.113257 | 1.333486 | 9.61E-29 | 5.42E-27 |
| SMPX | 0.898942 | 2.779342 | 1.628443 | 1.98E-05 | 5.58E-05 |
| TTC28 | 1.339214 | 3.408547 | 1.34777 | 2.03E-35 | 6.37E-33 |
| SCML1 | 6.613581 | 4.264238 | -0.63314 | 1.37E-07 | 5.55E-07 |
| MEOX1 | 0.730855 | 2.15694 | 1.561328 | 1.23E-24 | 3.85E-23 |
| CHST7 | 1.396206 | 2.546829 | 0.867191 | 3.15E-25 | 1.07E-23 |
| PARP15 | 1.002186 | 1.554497 | 0.633297 | 8.68E-06 | 2.60E-05 |
| PDE8B | 0.574648 | 1.034151 | 0.847696 | 2.66E-16 | 3.38E-15 |
| CR2 | 3.744093 | 7.811975 | 1.061071 | 3.92E-14 | 3.98E-13 |
| AL022718.1 | 33.95115 | 21.96235 | -0.62843 | 0.000472 | 0.001034 |
| CCDC69 | 4.910532 | 13.30879 | 1.438428 | 4.82E-23 | 1.25E-21 |
| AC005180.2 | 0.25992 | 0.877329 | 1.755048 | 1.44E-17 | 2.07E-16 |
| RGS9 | 0.373955 | 0.80976 | 1.114628 | 8.95E-15 | 9.76E-14 |
| PLPP3 | 7.821889 | 15.0836 | 0.947392 | 1.59E-38 | 1.42E-35 |
| VASH1 | 2.679011 | 4.551573 | 0.764665 | 4.32E-21 | 9.04E-20 |
| MYH10 | 3.906936 | 9.055749 | 1.212796 | 2.84E-25 | 9.70E-24 |
| CNRIP1 | 1.444429 | 3.4829 | 1.269789 | 9.94E-35 | 2.25E-32 |
| AGT | 20.35808 | 41.5104 | 1.027871 | 1.10E-18 | 1.79E-17 |
| SLC2A4 | 0.727058 | 3.134614 | 2.108146 | 1.59E-11 | 1.19E-10 |
| IGKV3-15 | 81.61188 | 162.9576 | 0.997646 | 1.81E-06 | 6.09E-06 |
| AL136380.1 | 10.16405 | 6.2017 | -0.71274 | 0.000501 | 0.001092 |
| FLT4 | 1.327618 | 2.300261 | 0.792958 | 2.43E-19 | 4.25E-18 |
| IGHA1 | 1034.893 | 2809.923 | 1.441049 | 1.29E-07 | 5.26E-07 |
| CENPE | 4.079864 | 2.636018 | -0.63016 | 3.17E-12 | 2.58E-11 |
| PRKG1 | 1.003009 | 2.853541 | 1.508419 | 2.25E-32 | 2.73E-30 |
| RFTN2 | 0.535094 | 1.033633 | 0.949861 | 5.35E-30 | 3.98E-28 |
| IGHV3OR16-15 | 0.339372 | 0.752153 | 1.148159 | 4.25E-07 | 1.59E-06 |
| AGAP1-IT1 | 0.735011 | 0.482247 | -0.60799 | 0.000641 | 0.001372 |
| TMSB15A | 0.477123 | 0.843171 | 0.821465 | 5.07E-09 | 2.63E-08 |
| KCNA5 | 0.1803 | 0.830696 | 2.203923 | 5.66E-22 | 1.33E-20 |
| TNFAIP8L3 | 0.795012 | 2.087117 | 1.392463 | 4.35E-25 | 1.44E-23 |
| CDC42EP3 | 2.768713 | 6.323769 | 1.191569 | 7.29E-22 | 1.68E-20 |
| AC112907.2 | 0.937399 | 0.564179 | -0.73251 | 0.005 | 0.008916 |
| Z83851.2 | 1.286877 | 0.822536 | -0.64572 | 1.87E-10 | 1.21E-09 |
| MAP3K20 | 6.858438 | 11.45863 | 0.740483 | 3.40E-06 | 1.09E-05 |
| BST1 | 1.582235 | 2.599474 | 0.716256 | 2.74E-14 | 2.84E-13 |
| ROR1 | 2.090708 | 3.515088 | 0.749569 | 4.95E-13 | 4.47E-12 |
| PTGDS | 10.12884 | 32.82704 | 1.696415 | 2.32E-22 | 5.69E-21 |
| GJC2 | 0.940907 | 1.573426 | 0.741786 | 4.65E-16 | 5.78E-15 |
| MYMX | 0.324341 | 0.691055 | 1.09129 | 6.20E-16 | 7.57E-15 |
| OXT | 0.897786 | 1.470761 | 0.712119 | 0.000176 | 0.000418 |
| IGHV1-17 | 0.369195 | 0.648914 | 0.813645 | 4.98E-08 | 2.17E-07 |
| UBXN10-AS1 | 0.605921 | 1.36091 | 1.16737 | 3.03E-05 | 8.25E-05 |
| AL049555.1 | 7.89866 | 4.476642 | -0.81919 | 1.22E-13 | 1.18E-12 |
| NPR2 | 1.69073 | 2.984251 | 0.819723 | 1.32E-22 | 3.31E-21 |
| IGHV7-81 | 0.915568 | 1.706047 | 0.897919 | 1.14E-05 | 3.33E-05 |
| ADAMTS5 | 1.088867 | 1.845921 | 0.761513 | 1.78E-13 | 1.70E-12 |
| EFCC1 | 1.155022 | 2.165036 | 0.90647 | 1.10E-18 | 1.79E-17 |
| TACR2 | 1.804191 | 11.20696 | 2.634971 | 2.48E-07 | 9.67E-07 |
| UBE2T | 27.12567 | 17.17138 | -0.65965 | 1.16E-17 | 1.68E-16 |
| IGKV2D-30 | 1.384711 | 3.664101 | 1.403874 | 1.69E-09 | 9.43E-09 |
| ARRDC5 | 0.413257 | 0.639049 | 0.628888 | 8.41E-07 | 3.00E-06 |
| OLFML2A | 4.494537 | 7.014709 | 0.642211 | 2.47E-16 | 3.16E-15 |
| SHISA3 | 0.685457 | 3.921263 | 2.516181 | 8.79E-26 | 3.30E-24 |
| EVC | 1.396994 | 4.357624 | 1.641216 | 1.29E-36 | 5.59E-34 |
| GPM6B | 0.720764 | 1.51216 | 1.069013 | 1.22E-11 | 9.30E-11 |
| BEX4 | 5.300405 | 12.26872 | 1.210811 | 7.94E-26 | 3.00E-24 |
| NPTX2 | 1.358739 | 2.348538 | 0.789495 | 1.75E-10 | 1.13E-09 |
| FGD1 | 2.322189 | 3.813579 | 0.71566 | 2.77E-23 | 7.44E-22 |
| SFN | 161.9855 | 62.76846 | -1.36775 | 2.94E-18 | 4.59E-17 |
| PODNL1 | 1.489915 | 2.37669 | 0.673724 | 3.81E-07 | 1.44E-06 |
| SYT8 | 5.407321 | 3.259411 | -0.7303 | 0.008292 | 0.014097 |
| PIK3CG | 1.351321 | 2.326141 | 0.783568 | 8.78E-16 | 1.06E-14 |
| TUBB4A | 0.327493 | 0.979831 | 1.58107 | 1.63E-11 | 1.22E-10 |
| CASQ2 | 0.614423 | 4.148836 | 2.755402 | 7.86E-22 | 1.81E-20 |
| THRB | 1.812393 | 2.883255 | 0.669803 | 1.25E-07 | 5.11E-07 |
| S100A11P1 | 1.092103 | 0.674213 | -0.69583 | 3.31E-07 | 1.26E-06 |
| AC037198.1 | 0.428738 | 1.04639 | 1.287253 | 8.18E-16 | 9.87E-15 |
| PLSCR4 | 3.117622 | 7.119418 | 1.191314 | 3.07E-26 | 1.22E-24 |
| MS4A1 | 3.532136 | 5.956249 | 0.753863 | 8.52E-11 | 5.78E-10 |
| RASL12 | 2.411186 | 6.206937 | 1.364138 | 8.21E-35 | 1.96E-32 |
| QKI | 2.791949 | 5.076051 | 0.862434 | 1.82E-25 | 6.41E-24 |
| IGLV4-60 | 6.082793 | 13.53309 | 1.153685 | 3.82E-05 | 0.000102 |
| ST8SIA4 | 1.422261 | 2.155389 | 0.599763 | 1.94E-12 | 1.63E-11 |
| TLR5 | 1.163808 | 1.777327 | 0.610856 | 3.16E-13 | 2.93E-12 |
| MET | 34.22204 | 19.17265 | -0.83588 | 0.000371 | 0.000829 |
| TMEM229A | 0.44789 | 0.737722 | 0.719933 | 6.85E-08 | 2.92E-07 |
| AGR3 | 63.79561 | 38.67192 | -0.72217 | 0.005528 | 0.009746 |
| LSP1 | 9.382369 | 15.23162 | 0.699045 | 1.76E-11 | 1.31E-10 |
| DOCK8 | 2.23638 | 3.61449 | 0.692627 | 5.99E-11 | 4.16E-10 |
| PKD2 | 3.909829 | 8.449304 | 1.111727 | 1.24E-29 | 8.49E-28 |
| CREB3L2 | 10.54879 | 15.98753 | 0.599869 | 6.96E-20 | 1.30E-18 |
| GHRL | 1.861827 | 4.10741 | 1.14151 | 2.66E-08 | 1.22E-07 |
| CHRD | 1.043621 | 1.832947 | 0.812566 | 8.52E-33 | 1.16E-30 |
| FPR2 | 0.554557 | 0.885711 | 0.6755 | 0.025851 | 0.039423 |
| ACVRL1 | 3.74487 | 7.332722 | 0.969433 | 5.00E-28 | 2.50E-26 |
| MIR3671 | 2.019626 | 3.035705 | 0.587943 | 2.50E-05 | 6.91E-05 |
| COL6A1 | 67.00328 | 121.9735 | 0.864265 | 2.17E-17 | 3.07E-16 |
| EGFLAM | 0.687065 | 1.19986 | 0.804347 | 3.28E-12 | 2.67E-11 |
| AC104083.1 | 3.829885 | 11.06447 | 1.530562 | 2.40E-26 | 9.53E-25 |
| CASC8 | 1.205051 | 0.633282 | -0.92818 | 2.19E-05 | 6.12E-05 |
| CXCL3 | 24.42071 | 11.84764 | -1.04351 | 3.04E-08 | 1.38E-07 |
| RERG | 0.971232 | 4.125421 | 2.086654 | 3.16E-34 | 5.83E-32 |
| SLC6A14 | 15.89455 | 7.675625 | -1.05018 | 5.97E-05 | 0.000154 |
| RTL8C | 49.24411 | 74.44632 | 0.596249 | 2.24E-11 | 1.65E-10 |
| RGN | 0.950208 | 2.558953 | 1.429239 | 1.41E-15 | 1.66E-14 |
| HIF3A | 0.801074 | 4.005458 | 2.32196 | 3.81E-16 | 4.77E-15 |
| CPLANE1 | 0.992203 | 1.556015 | 0.649149 | 6.35E-13 | 5.64E-12 |
| IGHV3-48 | 40.56407 | 67.9295 | 0.743836 | 9.36E-07 | 3.31E-06 |
| CRMP1 | 1.331476 | 2.617158 | 0.974974 | 5.93E-34 | 1.06E-31 |
| KRT15 | 6.224016 | 1.021042 | -2.6078 | 1.66E-07 | 6.65E-07 |
| TSPAN9 | 3.96558 | 8.631688 | 1.122111 | 7.24E-29 | 4.27E-27 |
| P2RY10 | 1.710607 | 3.088943 | 0.852605 | 1.36E-08 | 6.56E-08 |
| ZNF469 | 1.327916 | 2.031388 | 0.613301 | 1.82E-06 | 6.14E-06 |
| AC026740.1 | 2.012643 | 1.163304 | -0.79086 | 0.000127 | 0.000309 |
| TKTL1 | 0.946017 | 2.566934 | 1.440108 | 4.78E-06 | 1.50E-05 |
| MIR374B | 0.366588 | 0.654121 | 0.835397 | 9.82E-06 | 2.91E-05 |
| FEZ1 | 0.856502 | 1.937511 | 1.177676 | 2.51E-29 | 1.62E-27 |
| PREX2 | 0.532235 | 1.397552 | 1.392768 | 2.01E-32 | 2.47E-30 |
| CCDC9B | 1.854215 | 2.979569 | 0.684295 | 1.27E-07 | 5.19E-07 |
| TUBA1A | 20.43404 | 48.78722 | 1.255529 | 1.06E-24 | 3.33E-23 |
| AP001453.2 | 4.041776 | 2.416188 | -0.74226 | 1.81E-12 | 1.52E-11 |
| FAM171A2 | 0.711454 | 1.190332 | 0.742521 | 4.06E-09 | 2.13E-08 |
| BVES | 0.630557 | 2.711259 | 2.104265 | 4.79E-28 | 2.41E-26 |
| FP236383.3 | 3.391578 | 5.677026 | 0.743178 | 0.022853 | 0.035279 |
| SYNE1 | 0.629851 | 1.850243 | 1.554631 | 6.25E-31 | 5.73E-29 |
| CDYL2 | 1.305718 | 2.242977 | 0.780571 | 1.37E-17 | 1.98E-16 |
| PIP4P2 | 2.514535 | 4.307604 | 0.776594 | 6.31E-14 | 6.29E-13 |
| BHMT2 | 0.293042 | 1.314417 | 2.165243 | 3.76E-35 | 1.03E-32 |
| SLC38A11 | 0.885119 | 1.440872 | 0.702999 | 4.70E-05 | 0.000124 |
| MIR1245A | 0.39683 | 0.676472 | 0.769508 | 1.55E-06 | 5.28E-06 |
| GALNT14 | 0.792701 | 1.237094 | 0.642106 | 1.20E-06 | 4.16E-06 |
| TRAV6 | 0.425687 | 0.673226 | 0.661299 | 4.22E-06 | 1.33E-05 |
| CYSLTR1 | 0.444856 | 1.015196 | 1.190348 | 1.56E-21 | 3.43E-20 |
| GGH | 20.47617 | 11.59946 | -0.81989 | 0.000209 | 0.000488 |
| AC124319.1 | 1.630079 | 1.006734 | -0.69526 | 1.36E-05 | 3.94E-05 |
| TWIST2 | 1.014197 | 3.443187 | 1.763407 | 2.65E-25 | 9.07E-24 |
| CLIC2 | 2.654411 | 5.151248 | 0.95653 | 3.81E-25 | 1.28E-23 |
| GLIPR2 | 4.572961 | 7.737487 | 0.758736 | 4.58E-16 | 5.69E-15 |
| IL1RL1 | 0.364854 | 0.795995 | 1.12544 | 4.65E-14 | 4.69E-13 |
| OPN1SW | 1.16506 | 1.767254 | 0.601105 | 2.93E-08 | 1.33E-07 |
| CYCSP45 | 0.740426 | 0.4416 | -0.74562 | 4.70E-07 | 1.74E-06 |
| CPE | 6.084863 | 19.2283 | 1.659934 | 5.28E-29 | 3.22E-27 |
| OLR1 | 2.590463 | 3.930351 | 0.601448 | 1.98E-05 | 5.58E-05 |
| MMP2 | 38.88295 | 111.3176 | 1.517473 | 4.31E-28 | 2.17E-26 |
| CHIC1 | 1.202839 | 1.871724 | 0.637923 | 2.61E-12 | 2.16E-11 |
| MRC1 | 3.216336 | 5.393488 | 0.7458 | 2.44E-10 | 1.54E-09 |
| FXYD6 | 1.498297 | 5.298961 | 1.822385 | 1.97E-34 | 4.02E-32 |
| CTH | 3.969007 | 2.396526 | -0.72783 | 0.002912 | 0.005444 |
| SFRP4 | 11.69012 | 42.18733 | 1.85152 | 1.11E-22 | 2.81E-21 |
| CNTNAP1 | 1.729893 | 4.982296 | 1.526128 | 7.99E-32 | 8.68E-30 |
| PHLDB1 | 1.611831 | 3.564364 | 1.144944 | 2.23E-35 | 6.89E-33 |
| SCIMP | 1.56739 | 2.570928 | 0.713925 | 1.03E-10 | 6.89E-10 |
| RAB37 | 0.701188 | 1.163224 | 0.730256 | 7.44E-12 | 5.79E-11 |
| ZMAT1 | 0.438793 | 0.832428 | 0.923785 | 1.48E-12 | 1.25E-11 |
| TRBJ2-4 | 0.596909 | 1.090649 | 0.869603 | 2.71E-06 | 8.88E-06 |
| MIR8071-1 | 1.070364 | 1.704709 | 0.671423 | 2.20E-06 | 7.31E-06 |
| CTSK | 24.9347 | 56.13194 | 1.170667 | 8.64E-22 | 1.97E-20 |
| SFTPD | 0.189397 | 1.539277 | 3.022771 | 7.97E-12 | 6.18E-11 |
| CALHM5 | 0.575144 | 1.270706 | 1.143635 | 1.48E-25 | 5.35E-24 |
| S100A2 | 44.00991 | 2.803373 | -3.97259 | 3.89E-10 | 2.38E-09 |
| ANKRD6 | 0.489572 | 0.948065 | 0.953464 | 2.03E-23 | 5.54E-22 |
| CACNA2D2 | 0.495262 | 1.069618 | 1.11083 | 1.05E-19 | 1.92E-18 |
| ADRB2 | 0.480142 | 0.798432 | 0.733708 | 4.35E-17 | 5.99E-16 |
