# Supplementary material for: Identification of immunotherapy-related subtypes, characterization of tumor microenvironment infiltration, and development of a prognostic signature in gastric carcinoma
Source: Aging (Albany NY). 2024 Jun 25;16(14):11185–207. doi: 10.18632/aging.205968 (PMC11315391; doi:10.18632/aging.205968)
Supplement: Supplementary Table 3 [file aging-16-205968-s003.docx]

| **Supplementary Table 3. GO annotation analysis of DEGs between two clusters.** | | |
| --- | --- | --- |
| Term | Count | FDR |
| GO:0007155~cell adhesion | 187 | 2.83E-37 |
| GO:0006955~immune response | 166 | 2.94E-33 |
| GO:0050853~B cell receptor signaling pathway | 73 | 3.41E-29 |
| GO:0006911~phagocytosis, engulfment | 68 | 3.41E-29 |
| GO:0006910~phagocytosis, recognition | 63 | 3.64E-29 |
| GO:0002377~immunoglobulin production | 59 | 4.35E-27 |
| GO:0006958~complement activation, classical pathway | 68 | 9.90E-27 |
| GO:0050871~positive regulation of B cell activation | 60 | 1.55E-26 |
| GO:0002250~adaptive immune response | 135 | 1.74E-21 |
| GO:0030198~extracellular matrix organization | 72 | 7.42E-21 |
| GO:0001525~angiogenesis | 91 | 5.90E-20 |
| GO:0042742~defense response to bacterium | 80 | 9.90E-16 |
| GO:0006954~inflammatory response | 112 | 2.52E-14 |
| GO:0007160~cell-matrix adhesion | 45 | 1.24E-11 |
| GO:0045087~innate immune response | 137 | 3.61E-11 |
| GO:0007507~heart development | 67 | 2.13E-10 |
| GO:0098609~cell-cell adhesion | 61 | 3.02E-10 |
| GO:0042060~wound healing | 40 | 5.93E-09 |
| GO:0007411~axon guidance | 57 | 6.29E-09 |
| GO:0007204~positive regulation of cytosolic calcium ion concentration | 52 | 6.82E-09 |
| GO:0030199~collagen fibril organization | 29 | 1.04E-08 |
| GO:0009887~animal organ morphogenesis | 47 | 1.35E-08 |
| GO:0016477~cell migration | 72 | 2.11E-08 |
| GO:0007166~cell surface receptor signaling pathway | 78 | 3.98E-08 |
| GO:0007165~signal transduction | 229 | 4.25E-08 |
| GO:0070374~positive regulation of ERK1 and ERK2 cascade | 63 | 9.32E-08 |
| GO:0030335~positive regulation of cell migration | 68 | 1.07E-07 |
| GO:0007275~multicellular organism development | 60 | 2.29E-07 |
| GO:0008360~regulation of cell shape | 48 | 2.33E-07 |
| GO:0001570~vasculogenesis | 27 | 9.57E-07 |
| GO:0032496~response to lipopolysaccharide | 46 | 2.72E-06 |
| GO:0006935~chemotaxis | 39 | 4.48E-06 |
| GO:0051897~positive regulation of protein kinase B signaling | 40 | 4.48E-06 |
| GO:0001822~kidney development | 38 | 5.44E-06 |
| GO:0014068~positive regulation of phosphatidylinositol 3-kinase signaling | 30 | 9.40E-06 |
| GO:0045766~positive regulation of angiogenesis | 46 | 1.22E-05 |
| GO:0043410~positive regulation of MAPK cascade | 45 | 1.54E-05 |
| GO:0007156~homophilic cell adhesion via plasma membrane adhesion molecules | 47 | 1.92E-05 |
| GO:0001938~positive regulation of endothelial cell proliferation | 28 | 2.21E-05 |
| GO:0010628~positive regulation of gene expression | 103 | 3.31E-05 |
| GO:0008285~negative regulation of cell proliferation | 94 | 4.62E-05 |
| GO:0050731~positive regulation of peptidyl-tyrosine phosphorylation | 31 | 5.91E-05 |
| GO:0007229~integrin-mediated signaling pathway | 34 | 5.91E-05 |
| GO:0007267~cell-cell signaling | 56 | 5.91E-05 |
| GO:0007399~nervous system development | 86 | 7.54E-05 |
| GO:0019722~calcium-mediated signaling | 30 | 8.77E-05 |
| GO:0010976~positive regulation of neuron projection development | 37 | 9.55E-05 |
| GO:0048251~elastic fiber assembly | 10 | 1.05E-04 |
| GO:0010811~positive regulation of cell-substrate adhesion | 18 | 1.05E-04 |
| GO:0060070~canonical Wnt signaling pathway | 31 | 1.05E-04 |
| GO:0090090~negative regulation of canonical Wnt signaling pathway | 40 | 1.08E-04 |
| GO:0007159~leukocyte cell-cell adhesion | 16 | 1.42E-04 |
| GO:0071560~cellular response to transforming growth factor beta stimulus | 24 | 1.51E-04 |
| GO:0001503~ossification | 28 | 1.51E-04 |
| GO:0060326~cell chemotaxis | 25 | 1.63E-04 |
| GO:0048565~digestive tract development | 15 | 1.93E-04 |
| GO:0045669~positive regulation of osteoblast differentiation | 26 | 2.23E-04 |
| GO:0033674~positive regulation of kinase activity | 25 | 2.71E-04 |
| GO:0086091~regulation of heart rate by cardiac conduction | 18 | 3.09E-04 |
| GO:0043542~endothelial cell migration | 16 | 3.19E-04 |
| GO:0030334~regulation of cell migration | 32 | 3.26E-04 |
| GO:0001501~skeletal system development | 37 | 3.41E-04 |
| GO:0030336~negative regulation of cell migration | 44 | 3.41E-04 |
| GO:0043406~positive regulation of MAP kinase activity | 26 | 4.26E-04 |
| GO:0008217~regulation of blood pressure | 26 | 4.26E-04 |
| GO:0048146~positive regulation of fibroblast proliferation | 21 | 5.23E-04 |
| GO:0032331~negative regulation of chondrocyte differentiation | 13 | 5.54E-04 |
| GO:0016055~Wnt signaling pathway | 47 | 5.54E-04 |
| GO:0042493~response to drug | 63 | 5.89E-04 |
| GO:0002040~sprouting angiogenesis | 15 | 6.54E-04 |
| GO:0034446~substrate adhesion-dependent cell spreading | 21 | 6.54E-04 |
| GO:0010951~negative regulation of endopeptidase activity | 38 | 6.54E-04 |
| GO:0009612~response to mechanical stimulus | 22 | 7.02E-04 |
| GO:0007189~adenylate cyclase-activating G-protein coupled receptor signaling pathway | 35 | 7.05E-04 |
| GO:0070527~platelet aggregation | 18 | 7.36E-04 |
| GO:0007517~muscle organ development | 29 | 0.001058 |
| GO:0048661~positive regulation of smooth muscle cell proliferation | 23 | 0.001191 |
| GO:0019934~cGMP-mediated signaling | 13 | 0.001359 |
| GO:0008016~regulation of heart contraction | 15 | 0.001359 |
| GO:0030324~lung development | 27 | 0.001366 |
| GO:0030501~positive regulation of bone mineralization | 17 | 0.001556 |
| GO:0003094~glomerular filtration | 11 | 0.001561 |
| GO:0003151~outflow tract morphogenesis | 18 | 0.001792 |
| GO:0006508~proteolysis | 79 | 0.001942 |
| GO:0042542~response to hydrogen peroxide | 19 | 0.001961 |
| GO:0016525~negative regulation of angiogenesis | 33 | 0.002086 |
| GO:0010595~positive regulation of endothelial cell migration | 22 | 0.002179 |
| GO:0007169~transmembrane receptor protein tyrosine kinase signaling pathway | 34 | 0.002179 |
| GO:0001886~endothelial cell morphogenesis | 9 | 0.002215 |
| GO:0033627~cell adhesion mediated by integrin | 16 | 0.00227 |
| GO:0007219~Notch signaling pathway | 31 | 0.002822 |
| GO:0014911~positive regulation of smooth muscle cell migration | 12 | 0.002822 |
| GO:0032355~response to estradiol | 30 | 0.003048 |
| GO:0030194~positive regulation of blood coagulation | 10 | 0.003368 |
| GO:0032760~positive regulation of tumor necrosis factor production | 29 | 0.003902 |
| GO:0001649~osteoblast differentiation | 32 | 0.004024 |
| GO:0003148~outflow tract septum morphogenesis | 12 | 0.004259 |
| GO:0001755~neural crest cell migration | 17 | 0.004542 |
| GO:0050919~negative chemotaxis | 15 | 0.004709 |
| GO:0051145~smooth muscle cell differentiation | 10 | 0.005652 |
| GO:0018108~peptidyl-tyrosine phosphorylation | 34 | 0.005675 |
| GO:0051591~response to cAMP | 17 | 0.005817 |
| GO:0001666~response to hypoxia | 41 | 0.006712 |
| GO:0030036~actin cytoskeleton organization | 41 | 0.006712 |
| GO:0008284~positive regulation of cell proliferation | 97 | 0.006713 |
| GO:0001658~branching involved in ureteric bud morphogenesis | 16 | 0.006877 |
| GO:0071526~semaphorin-plexin signaling pathway | 14 | 0.007101 |
| GO:0010718~positive regulation of epithelial to mesenchymal transition | 17 | 0.007223 |
| GO:0045446~endothelial cell differentiation | 9 | 0.007223 |
| GO:0030168~platelet activation | 20 | 0.007349 |
| GO:0035633~maintenance of permeability of blood-brain barrier | 13 | 0.007834 |
| GO:0042730~fibrinolysis | 10 | 0.00856 |
| GO:0009611~response to wounding | 21 | 0.008632 |
| GO:0050804~modulation of synaptic transmission | 20 | 0.008873 |
| GO:0007010~cytoskeleton organization | 34 | 0.008873 |
| GO:0007193~adenylate cyclase-inhibiting G-protein coupled receptor signaling pathway | 18 | 0.008918 |
| GO:0007519~skeletal muscle tissue development | 18 | 0.008918 |
| GO:0001569~branching involved in blood vessel morphogenesis | 13 | 0.010428 |
| GO:0009617~response to bacterium | 30 | 0.010428 |
| GO:0001654~eye development | 16 | 0.01066 |
| GO:0010629~negative regulation of gene expression | 60 | 0.01066 |
| GO:0051216~cartilage development | 19 | 0.010725 |
| GO:0030178~negative regulation of Wnt signaling pathway | 17 | 0.010773 |
| GO:0071625~vocalization behavior | 9 | 0.011352 |
| GO:0007596~blood coagulation | 24 | 0.011404 |
| GO:0032570~response to progesterone | 14 | 0.011435 |
| GO:0060412~ventricular septum morphogenesis | 14 | 0.011435 |
| GO:0050679~positive regulation of epithelial cell proliferation | 21 | 0.011575 |
| GO:0048844~artery morphogenesis | 11 | 0.011876 |
| GO:1904646~cellular response to beta-amyloid | 16 | 0.012886 |
| GO:0007157~heterophilic cell-cell adhesion via plasma membrane cell adhesion molecules | 17 | 0.012987 |
| GO:0009410~response to xenobiotic stimulus | 49 | 0.014485 |
| GO:0045907~positive regulation of vasoconstriction | 14 | 0.014485 |
| GO:0043588~skin development | 14 | 0.014485 |
| GO:2001046~positive regulation of integrin-mediated signaling pathway | 8 | 0.014485 |
| GO:1903238~positive regulation of leukocyte tethering or rolling | 8 | 0.014485 |
| GO:0034113~heterotypic cell-cell adhesion | 12 | 0.014762 |
| GO:0051209~release of sequestered calcium ion into cytosol | 15 | 0.015253 |
| GO:0090023~positive regulation of neutrophil chemotaxis | 11 | 0.016389 |
| GO:0048557~embryonic digestive tract morphogenesis | 9 | 0.016814 |
| GO:0043116~negative regulation of vascular permeability | 9 | 0.016814 |
| GO:0008015~blood circulation | 13 | 0.016814 |
| GO:0061028~establishment of endothelial barrier | 10 | 0.017011 |
| GO:0048048~embryonic eye morphogenesis | 7 | 0.017476 |
| GO:0098742~cell-cell adhesion via plasma-membrane adhesion molecules | 14 | 0.017851 |
| GO:0001937~negative regulation of endothelial cell proliferation | 14 | 0.017851 |
| GO:0007043~cell-cell junction assembly | 14 | 0.017851 |
| GO:0035176~social behavior | 17 | 0.018437 |
| GO:1900026~positive regulation of substrate adhesion-dependent cell spreading | 15 | 0.018437 |
| GO:0048010~vascular endothelial growth factor receptor signaling pathway | 12 | 0.018978 |
| GO:0070098~chemokine-mediated signaling pathway | 20 | 0.019109 |
| GO:0007200~phospholipase C-activating G-protein coupled receptor signaling pathway | 19 | 0.020348 |
| GO:0002027~regulation of heart rate | 13 | 0.021156 |
| GO:0051496~positive regulation of stress fiber assembly | 17 | 0.022284 |
| GO:0050766~positive regulation of phagocytosis | 16 | 0.022603 |
| GO:0048839~inner ear development | 16 | 0.022603 |
| GO:0001568~blood vessel development | 15 | 0.022603 |
| GO:0006939~smooth muscle contraction | 10 | 0.022982 |
| GO:0035567~non-canonical Wnt signaling pathway | 10 | 0.022982 |
| GO:0060307~regulation of ventricular cardiac muscle cell membrane repolarization | 10 | 0.022982 |
| GO:0060021~palate development | 19 | 0.023469 |
| GO:0051770~positive regulation of nitric-oxide synthase biosynthetic process | 9 | 0.023479 |
| GO:0071711~basement membrane organization | 9 | 0.023479 |
| GO:0035987~endodermal cell differentiation | 12 | 0.023844 |
| GO:0051281~positive regulation of release of sequestered calcium ion into cytosol | 12 | 0.023844 |
| GO:0030155~regulation of cell adhesion | 18 | 0.024348 |
| GO:0001657~ureteric bud development | 13 | 0.02575 |
| GO:0045880~positive regulation of smoothened signaling pathway | 13 | 0.02575 |
| GO:0001837~epithelial to mesenchymal transition | 15 | 0.026706 |
| GO:0022617~extracellular matrix disassembly | 15 | 0.026706 |
| GO:0036120~cellular response to platelet-derived growth factor stimulus | 11 | 0.027379 |
| GO:0046718~viral entry into host cell | 25 | 0.02785 |
| GO:0008544~epidermis development | 22 | 0.028234 |
| GO:0030182~neuron differentiation | 37 | 0.028234 |
| GO:0007612~learning | 18 | 0.028234 |
| GO:0001771~immunological synapse formation | 7 | 0.028234 |
| GO:0030154~cell differentiation | 111 | 0.028404 |
| GO:0043552~positive regulation of phosphatidylinositol 3-kinase activity | 12 | 0.029865 |
| GO:0051491~positive regulation of filopodium assembly | 12 | 0.029865 |
| GO:0010881~regulation of cardiac muscle contraction by regulation of the release of sequestered calcium ion | 10 | 0.029983 |
| GO:0006952~defense response | 21 | 0.030268 |
| GO:0090131~mesenchyme migration | 5 | 0.031122 |
| GO:0046069~cGMP catabolic process | 5 | 0.031122 |
| GO:1905007~positive regulation of epithelial to mesenchymal transition involved in endocardial cushion formation | 5 | 0.031122 |
| GO:0001764~neuron migration | 28 | 0.031122 |
| GO:0000122~negative regulation of transcription from RNA polymerase II promoter | 150 | 0.031529 |
| GO:0048514~blood vessel morphogenesis | 9 | 0.031529 |
| GO:0042308~negative regulation of protein import into nucleus | 8 | 0.031529 |
| GO:0001955~blood vessel maturation | 6 | 0.03258 |
| GO:0014012~peripheral nervous system axon regeneration | 6 | 0.03258 |
| GO:0051450~myoblast proliferation | 6 | 0.03258 |
| GO:2000969~positive regulation of AMPA receptor activity | 6 | 0.03258 |
| GO:0001954~positive regulation of cell-matrix adhesion | 11 | 0.03368 |
| GO:0043491~protein kinase B signaling | 16 | 0.035573 |
| GO:0071773~cellular response to BMP stimulus | 12 | 0.036675 |
| GO:0001974~blood vessel remodeling | 13 | 0.037725 |
| GO:0016339~calcium-dependent cell-cell adhesion via plasma membrane cell adhesion molecules | 14 | 0.037725 |
| GO:0050729~positive regulation of inflammatory response | 26 | 0.037739 |
| GO:0051056~regulation of small GTPase mediated signal transduction | 27 | 0.037739 |
| GO:0071260~cellular response to mechanical stimulus | 22 | 0.039981 |
| GO:0007420~brain development | 51 | 0.040379 |
| GO:0043032~positive regulation of macrophage activation | 9 | 0.042996 |
| GO:0007263~nitric oxide mediated signal transduction | 9 | 0.042996 |
| GO:0050901~leukocyte tethering or rolling | 9 | 0.042996 |
| GO:0048041~focal adhesion assembly | 11 | 0.042996 |
| GO:0032967~positive regulation of collagen biosynthetic process | 11 | 0.042996 |
| GO:0030593~neutrophil chemotaxis | 21 | 0.04343 |
| GO:0006936~muscle contraction | 22 | 0.043692 |
| GO:0060048~cardiac muscle contraction | 14 | 0.043692 |
| GO:0055074~calcium ion homeostasis | 12 | 0.043692 |
| GO:0051482~positive regulation of cytosolic calcium ion concentration involved in phospholipase C-activating G-protein coupled signaling pathway | 12 | 0.043692 |
| GO:0042476~odontogenesis | 12 | 0.043692 |
| GO:0035025~positive regulation of Rho protein signal transduction | 12 | 0.043692 |
| GO:0048566~embryonic digestive tract development | 8 | 0.043692 |
| GO:0046849~bone remodeling | 8 | 0.043692 |
| GO:0033630~positive regulation of cell adhesion mediated by integrin | 8 | 0.043692 |
| GO:0030855~epithelial cell differentiation | 23 | 0.043692 |
| GO:0050829~defense response to Gram-negative bacterium | 23 | 0.043692 |
| GO:0030326~embryonic limb morphogenesis | 13 | 0.043692 |
| GO:0007188~adenylate cyclase-modulating G-protein coupled receptor signaling pathway | 16 | 0.047292 |
| GO:0010596~negative regulation of endothelial cell migration | 10 | 0.048755 |
