# Supplementary material for: Identification of immunotherapy-related subtypes, characterization of tumor microenvironment infiltration, and development of a prognostic signature in gastric carcinoma
Source: Aging (Albany NY). 2024 Jun 25;16(14):11185–207. doi: 10.18632/aging.205968 (PMC11315391; doi:10.18632/aging.205968)
Supplement: Supplementary Table 4 [file aging-16-205968-s004.pdf]

Supplementary Table 4. KEGG pathway enrichment analysis of DEGs between two clusters.

Term	Count	FDR
hsa04512:ECM-receptor interaction	51	1.33E-20
hsa04510:Focal adhesion	73	1.70E-15
hsa04151:PI3K-Akt signaling pathway	95	4.90E-11
hsa04514:Cell adhesion molecules	54	3.41E-10
hsa05146:Amoebiasis	41	5.81E-10
hsa04974:Protein digestion and absorption	41	6.96E-10
hsa05144:Malaria	27	8.93E-10
hsa04610:Complement and coagulation cascades	36	1.42E-09
hsa05200:Pathways in cancer	120	3.12E-09
hsa05414:Dilated cardiomyopathy	36	5.88E-08
hsa04060:Cytokine-cytokine receptor interaction	74	1.79E-07
hsa05410:Hypertrophic cardiomyopathy	33	4.69E-07
hsa05412:Arrhythmogenic right ventricular cardiomyopathy	30	4.69E-07
hsa04020:Calcium signaling pathway	60	5.21E-06
hsa04270:Vascular smooth muscle contraction	40	5.87E-06
hsa04640:Hematopoietic cell lineage	32	1.58E-05
hsa05205:Proteoglycans in cancer	52	1.91E-05
hsa04022:cGMP-PKG signaling pathway	44	4.79E-05
hsa04933:AGE-RAGE signaling pathway in diabetic complications	31	5.36E-05
hsa04360:Axon guidance	46	8.23E-05
hsa04611:Platelet activation	35	1.02E-04
hsa05165:Human papillomavirus infection	71	1.02E-04
hsa04080:Neuroactive ligand-receptor interaction	75	1.91E-04
hsa04261:Adrenergic signaling in cardiomyocytes	39	2.00E-04
hsa04061:Viral protein interaction with cytokine and cytokine receptor	29	3.55E-04
hsa05418:Fluid shear stress and atherosclerosis	36	4.51E-04
hsa04015:Rap1 signaling pathway	47	0.00126
hsa04921:Oxytocin signaling pathway	37	0.0017
hsa04924:Renin secretion	21	0.002423
hsa04010:MAPK signaling pathway	59	0.003256
hsa04810:Regulation of actin cytoskeleton	46	0.005231
hsa05217:Basal cell carcinoma	19	0.005347
hsa04062:Chemokine signaling pathway	41	0.008065
hsa05202:Transcriptional misregulation in cancer	41	0.008697
hsa04340:Hedgehog signaling pathway	17	0.009668
hsa04926:Relaxin signaling pathway	30	0.010632
hsa04916:Melanogenesis	25	0.011776
hsa04371:Apelin signaling pathway	31	0.01651
hsa04024:cAMP signaling pathway	44	0.018462
hsa04540:Gap junction	22	0.019733
hsa04350:TGF-beta signaling pathway	23	0.020045
hsa05150:Staphylococcus aureus infection	23	0.025764
hsa01521:EGFR tyrosine kinase inhibitor resistance	20	0.026021
hsa04713:Circadian entrainment	23	0.028093
hsa04145:Phagosome	32	0.029498

hsa05222:Small cell lung cancer	22	0.029929
hsa04925:Aldosterone synthesis and secretion	23	0.029929
hsa03320:PPAR signaling pathway	19	0.029929
