# Supplementary material for: Identification of immunotherapy-related subtypes, characterization of tumor microenvironment infiltration, and development of a prognostic signature in gastric carcinoma
Source: Aging (Albany NY). 2024 Jun 25;16(14):11185–207. doi: 10.18632/aging.205968 (PMC11315391; doi:10.18632/aging.205968)
Supplement: Supplementary Table 5 [file aging-16-205968-s005.docx]

| **Supplementary Table 5. Univariate Cox regression analysis of DEGs presented significant correlations with the OS of GC patients.** | | | | |
| --- | --- | --- | --- | --- |
| Gene symbol | HR | HR.95L | HR.95H | *P*-value |
| FAM19A5 | 1.312107 | 1.010102 | 1.704408 | 0.041823 |
| CTGF | 1.258924 | 1.073155 | 1.476851 | 0.004703 |
| FGL1 | 1.204459 | 1.0246 | 1.415891 | 0.024166 |
| COL3A1 | 1.202209 | 1.046662 | 1.380873 | 0.009185 |
| MAP7D3 | 1.43286 | 1.031098 | 1.991169 | 0.032163 |
| RETN | 1.453736 | 1.081641 | 1.953837 | 0.01313 |
| INMT | 1.161636 | 1.00531 | 1.342272 | 0.042176 |
| TCN2 | 1.301928 | 1.047302 | 1.61846 | 0.017492 |
| KCNQ1 | 0.795367 | 0.673618 | 0.93912 | 0.006914 |
| PCDHGB5 | 1.466604 | 1.0176 | 2.113727 | 0.040022 |
| SELE | 1.253722 | 1.040887 | 1.510075 | 0.017212 |
| APBB1 | 1.311194 | 1.017682 | 1.689358 | 0.036124 |
| FES | 1.308296 | 1.022092 | 1.674644 | 0.032888 |
| MAP3K12 | 1.413981 | 1.030451 | 1.94026 | 0.031891 |
| AEBP1 | 1.182202 | 1.034037 | 1.351596 | 0.014291 |
| TBCEL | 1.437881 | 1.023673 | 2.01969 | 0.036177 |
| EFEMP1 | 1.204564 | 1.068512 | 1.357939 | 0.002337 |
| ANTXR2 | 1.203045 | 1.018355 | 1.421231 | 0.029716 |
| SYDE1 | 1.278981 | 1.035987 | 1.578969 | 0.022089 |
| RGS4 | 1.277182 | 1.094432 | 1.490447 | 0.001901 |
| BOC | 1.223167 | 1.025528 | 1.458895 | 0.025071 |
| MSRB3 | 1.145181 | 1.015781 | 1.291064 | 0.026698 |
| NRP1 | 1.696094 | 1.321445 | 2.176961 | 3.34E-05 |
| PODN | 1.151999 | 1.025404 | 1.294223 | 0.017203 |
| AVPR1A | 1.669496 | 1.160542 | 2.401652 | 0.005737 |
| GGT5 | 1.343609 | 1.084141 | 1.665176 | 0.006978 |
| FBN1 | 1.350994 | 1.120576 | 1.628793 | 0.001615 |
| MAP6 | 1.402066 | 1.050209 | 1.871807 | 0.021891 |
| PEAR1 | 1.377197 | 1.018155 | 1.862851 | 0.037829 |
| DAB2 | 1.419821 | 1.145671 | 1.759573 | 0.001363 |
| SLC18A2 | 1.428808 | 1.076003 | 1.897292 | 0.013654 |
| ANTXR1 | 1.248729 | 1.06321 | 1.466618 | 0.006792 |
| LPAR1 | 1.220605 | 1.015243 | 1.467509 | 0.033929 |
| TMEM100 | 1.178266 | 1.002256 | 1.385185 | 0.046893 |
| CX3CR1 | 1.294849 | 1.02347 | 1.638184 | 0.031296 |
| NAP1L3 | 1.330372 | 1.020435 | 1.734447 | 0.034906 |
| SELP | 1.272942 | 1.060638 | 1.527741 | 0.009532 |
| COPZ2 | 1.300718 | 1.051183 | 1.609487 | 0.015551 |
| ADH1B | 1.145143 | 1.010315 | 1.297963 | 0.033962 |
| FAM110B | 1.27789 | 1.045237 | 1.562328 | 0.016782 |
| EHD2 | 1.232762 | 1.024139 | 1.483883 | 0.026959 |
| PXDN | 1.332071 | 1.094861 | 1.620673 | 0.004161 |
| SLC7A2 | 1.267745 | 1.112515 | 1.444635 | 0.000371 |
| COL12A1 | 1.167368 | 1.002764 | 1.358992 | 0.045983 |
| TMTC1 | 1.343799 | 1.100643 | 1.640673 | 0.003713 |
| COL1A1 | 1.143846 | 1.017316 | 1.286112 | 0.02464 |
| PCDH17 | 1.279479 | 1.044463 | 1.567377 | 0.017308 |
| ARHGEF17 | 1.280568 | 1.009633 | 1.624207 | 0.041449 |
| TMEM200B | 1.393773 | 1.051408 | 1.847622 | 0.02097 |
| PALMD | 1.441448 | 1.014295 | 2.04849 | 0.041438 |
| EDNRA | 1.315832 | 1.099543 | 1.574667 | 0.002738 |
| KLF9 | 1.227833 | 1.033325 | 1.458955 | 0.019674 |
| ROBO4 | 1.469322 | 1.055751 | 2.044901 | 0.02251 |
| GPR34 | 1.315123 | 1.056503 | 1.63705 | 0.014208 |
| VTN | 1.178848 | 1.054255 | 1.318165 | 0.003889 |
| NRP2 | 1.28292 | 1.022211 | 1.610121 | 0.031595 |
| DUSP1 | 1.304266 | 1.113215 | 1.528105 | 0.001012 |
| PEG10 | 1.159417 | 1.020975 | 1.316631 | 0.022613 |
| CTSO | 1.224839 | 1.006563 | 1.490449 | 0.042838 |
| KCNJ8 | 1.400774 | 1.138293 | 1.723781 | 0.001455 |
| SPARC | 1.295161 | 1.109885 | 1.511365 | 0.001025 |
| SLC5A5 | 1.192296 | 1.004255 | 1.415547 | 0.044595 |
| NTN4 | 1.26843 | 1.034241 | 1.555649 | 0.022415 |
| NOVA2 | 1.615462 | 1.010993 | 2.581342 | 0.04489 |
| MYB | 0.798419 | 0.688653 | 0.925681 | 0.002851 |
| MFAP2 | 1.248399 | 1.05457 | 1.477853 | 0.009962 |
| GLT8D2 | 1.328997 | 1.113954 | 1.585553 | 0.001587 |
| RBMS3 | 1.502575 | 1.145651 | 1.970699 | 0.003255 |
| GREM1 | 1.117286 | 1.009687 | 1.236353 | 0.031829 |
| MPDZ | 1.270304 | 1.057338 | 1.526164 | 0.010604 |
| CSF1R | 1.220942 | 1.031883 | 1.44464 | 0.02004 |
| PRKD1 | 1.388547 | 1.117537 | 1.725279 | 0.003046 |
| PDE1B | 1.909167 | 1.3281 | 2.744463 | 0.000479 |
| FOLR2 | 1.2289 | 1.043297 | 1.447522 | 0.013612 |
| PDGFRA | 1.250206 | 1.060389 | 1.474001 | 0.007864 |
| PROS1 | 1.245869 | 1.059583 | 1.464906 | 0.007806 |
| MYCT1 | 1.564333 | 1.098548 | 2.22761 | 0.013097 |
| PCDHB6 | 1.39691 | 1.01105 | 1.930031 | 0.042704 |
| THBD | 1.389063 | 1.101207 | 1.752163 | 0.005543 |
| ZNF521 | 1.410142 | 1.139835 | 1.744551 | 0.001549 |
| SERPINE2 | 1.151069 | 1.001792 | 1.322589 | 0.047119 |
| GFPT2 | 1.234763 | 1.031764 | 1.477702 | 0.021381 |
| GIPC3 | 1.926799 | 1.17492 | 3.159835 | 0.009358 |
| LOX | 1.315293 | 1.116087 | 1.550055 | 0.001073 |
| PARVA | 1.284587 | 1.026355 | 1.60779 | 0.028731 |
| HLX | 1.739145 | 1.170494 | 2.584058 | 0.006159 |
| FCN1 | 1.350686 | 1.110118 | 1.643387 | 0.002666 |
| FGFR1 | 1.251036 | 1.00854 | 1.551837 | 0.041618 |
| BATF2 | 0.807106 | 0.685339 | 0.950507 | 0.01022 |
| ELMO1 | 1.382937 | 1.062689 | 1.799694 | 0.015849 |
| VSNL1 | 0.869914 | 0.76451 | 0.98985 | 0.034449 |
| VEGFC | 1.385435 | 1.088001 | 1.76418 | 0.008194 |
| GNAI1 | 1.392286 | 1.103603 | 1.756483 | 0.005247 |
| STARD8 | 1.616296 | 1.196491 | 2.183396 | 0.001754 |
| VSTM2L | 1.18136 | 1.026219 | 1.359956 | 0.020326 |
| DLG4 | 1.333854 | 1.018239 | 1.747297 | 0.036513 |
| OMD | 1.191666 | 1.04239 | 1.36232 | 0.01023 |
| PLXNC1 | 1.418435 | 1.076477 | 1.86902 | 0.013008 |
| SNCA | 1.220807 | 1.017647 | 1.464525 | 0.031688 |
| TRIM29 | 0.86935 | 0.766991 | 0.985369 | 0.028483 |
| SLC24A3 | 1.206889 | 1.003581 | 1.451385 | 0.045723 |
| CYP2U1 | 1.632103 | 1.202953 | 2.214351 | 0.001649 |
| CXCR4 | 1.237721 | 1.074294 | 1.426009 | 0.003159 |
| SRPX | 1.144253 | 1.017127 | 1.287269 | 0.024924 |
| HGF | 1.547997 | 1.101603 | 2.175278 | 0.01182 |
| DCN | 1.186535 | 1.042022 | 1.35109 | 0.009847 |
| HECW2 | 1.402341 | 1.048157 | 1.876209 | 0.022809 |
| ANGPTL2 | 1.166566 | 1.015278 | 1.340399 | 0.029712 |
| AKAP12 | 1.237498 | 1.055097 | 1.451432 | 0.008814 |
| DCLK1 | 1.409613 | 1.093187 | 1.817629 | 0.008124 |
| KCNE4 | 1.378517 | 1.072592 | 1.771698 | 0.012165 |
| IL1R1 | 1.251884 | 1.013154 | 1.546867 | 0.037432 |
| CDH6 | 1.726611 | 1.254662 | 2.376087 | 0.000801 |
| MYL9 | 1.099753 | 1.000987 | 1.208263 | 0.047645 |
| EID1 | 1.411681 | 1.085632 | 1.835653 | 0.010078 |
| ZNF662 | 1.425226 | 1.02504 | 1.98165 | 0.035115 |
| LIN7A | 1.688725 | 1.238278 | 2.303029 | 0.000933 |
| SNX29 | 1.391169 | 1.035032 | 1.869847 | 0.028657 |
| RBP4 | 1.129842 | 1.018721 | 1.253083 | 0.020827 |
| FAP | 1.243929 | 1.060999 | 1.458399 | 0.007155 |
| SPON1 | 1.120001 | 1.009258 | 1.242895 | 0.032889 |
| ADAMTSL3 | 1.290394 | 1.004407 | 1.657811 | 0.046112 |
| ISLR | 1.168676 | 1.019154 | 1.340135 | 0.025642 |
| GJA1 | 1.287696 | 1.115884 | 1.485962 | 0.000539 |
| CXorf36 | 1.521269 | 1.137967 | 2.033677 | 0.004618 |
| SMARCA1 | 1.265845 | 1.057705 | 1.514943 | 0.010109 |
| EBF3 | 1.27557 | 1.000371 | 1.626475 | 0.049651 |
| C1QTNF2 | 1.491046 | 1.025168 | 2.168638 | 0.036617 |
| GPNMB | 1.181011 | 1.029712 | 1.354541 | 0.01738 |
| PROC | 1.23706 | 1.026796 | 1.490383 | 0.02521 |
| FAM69B | 1.256802 | 1.00631 | 1.569647 | 0.043859 |
| TFPI2 | 1.304642 | 1.102297 | 1.54413 | 0.001984 |
| SH3BGRL | 1.25934 | 1.053791 | 1.504983 | 0.011205 |
| RNF217 | 1.541749 | 1.117608 | 2.126854 | 0.008356 |
| TUSC3 | 1.25928 | 1.057014 | 1.50025 | 0.009862 |
| SEMA6A | 1.161505 | 1.016572 | 1.327101 | 0.027689 |
| PCOLCE | 1.174806 | 1.007254 | 1.370229 | 0.040167 |
| DEGS2 | 0.849569 | 0.727992 | 0.99145 | 0.038552 |
| JAM3 | 1.220905 | 1.051229 | 1.417967 | 0.008939 |
| CDH5 | 1.261944 | 1.0221 | 1.558069 | 0.030525 |
| CPA3 | 1.147471 | 1.020293 | 1.290503 | 0.021724 |
| SPOCK1 | 1.152803 | 1.021163 | 1.301412 | 0.021535 |
| LRP12 | 1.63376 | 1.076539 | 2.4794 | 0.021083 |
| BICC1 | 1.438522 | 1.113196 | 1.858921 | 0.00544 |
| ABCC9 | 1.381419 | 1.088044 | 1.753899 | 0.007984 |
| SLIT2 | 1.21418 | 1.044589 | 1.411305 | 0.011462 |
| APOC3 | 1.158333 | 1.016535 | 1.31991 | 0.027376 |
| FAM126A | 1.58499 | 1.053982 | 2.383527 | 0.02693 |
| RHOQ | 1.354528 | 1.034958 | 1.772774 | 0.027089 |
| HCFC2 | 1.472559 | 1.045735 | 2.073594 | 0.026689 |
| PDGFRB | 1.255882 | 1.058898 | 1.489509 | 0.00886 |
| ROR2 | 1.227553 | 1.048438 | 1.437268 | 0.010841 |
| RNF43 | 0.778564 | 0.659916 | 0.918543 | 0.003005 |
| FMO1 | 1.24879 | 1.00596 | 1.550237 | 0.044028 |
| EPB41L3 | 1.304064 | 1.064473 | 1.597582 | 0.010372 |
| HHIP | 1.382628 | 1.001372 | 1.909042 | 0.049035 |
| C5AR1 | 1.272334 | 1.047167 | 1.545917 | 0.015361 |
| CLEC1A | 1.413858 | 1.04609 | 1.910919 | 0.024252 |
| LRRN1 | 1.211876 | 1.035502 | 1.418292 | 0.016634 |
| ADARB1 | 1.324032 | 1.033419 | 1.69637 | 0.026421 |
| FBLN1 | 1.137895 | 1.023408 | 1.265189 | 0.016958 |
| ZEB1 | 1.25524 | 1.030018 | 1.52971 | 0.024253 |
| FZD1 | 1.349135 | 1.021393 | 1.782041 | 0.034941 |
| ASF1B | 0.813847 | 0.683304 | 0.969331 | 0.020935 |
| ADAMTS8 | 1.258319 | 1.044753 | 1.51554 | 0.015465 |
| GPR176 | 1.579914 | 1.174096 | 2.126002 | 0.002531 |
| E2F2 | 0.748411 | 0.618024 | 0.906307 | 0.003005 |
| HOXA13 | 0.879184 | 0.773902 | 0.99879 | 0.047865 |
| RBMS1 | 1.426649 | 1.116012 | 1.823752 | 0.004568 |
| SLC16A7 | 1.419934 | 1.02948 | 1.958478 | 0.032594 |
| TEK | 1.303197 | 1.060132 | 1.601993 | 0.011924 |
| FILIP1L | 1.187766 | 1.011015 | 1.395416 | 0.036329 |
| LRRC8C | 1.498656 | 1.060339 | 2.118164 | 0.021913 |
| RPS6KA2 | 1.336569 | 1.016898 | 1.756732 | 0.037515 |
| PLAT | 1.211111 | 1.010517 | 1.451524 | 0.038153 |
| CLDN11 | 1.220595 | 1.054769 | 1.412491 | 0.007457 |
| CAV1 | 1.182967 | 1.043457 | 1.34113 | 0.008681 |
| CHRDL1 | 1.209611 | 1.031556 | 1.4184 | 0.019161 |
| GPR162 | 1.397209 | 1.03073 | 1.893989 | 0.031164 |
| C20orf194 | 1.38752 | 1.041424 | 1.848634 | 0.025272 |
| IGFBP5 | 1.19547 | 1.036209 | 1.379208 | 0.014382 |
| BACE1 | 1.37748 | 1.008024 | 1.882347 | 0.044418 |
| COL4A2 | 1.221812 | 1.013293 | 1.473241 | 0.035881 |
| SEMA5B | 1.78981 | 1.133648 | 2.82576 | 0.012478 |
| MAGEH1 | 1.501898 | 1.155975 | 1.951336 | 0.002326 |
| PER1 | 1.328541 | 1.077857 | 1.637529 | 0.007752 |
| LDB2 | 1.440529 | 1.177837 | 1.761809 | 0.00038 |
| GCG | 1.177338 | 1.03524 | 1.33894 | 0.012857 |
| RHOJ | 1.249614 | 1.02365 | 1.525458 | 0.028549 |
| RGS2 | 1.289584 | 1.109078 | 1.499469 | 0.000948 |
| NID2 | 1.406492 | 1.119685 | 1.766765 | 0.003373 |
| DACT1 | 1.224904 | 1.01595 | 1.476835 | 0.033521 |
| AMBP | 1.291873 | 1.060229 | 1.574126 | 0.011084 |
| DPYSL3 | 1.136475 | 1.016958 | 1.270037 | 0.024034 |
| NNMT | 1.257933 | 1.07336 | 1.474245 | 0.004591 |
| RGS5 | 1.202963 | 1.034858 | 1.398376 | 0.016123 |
| NR3C1 | 1.50883 | 1.120021 | 2.032613 | 0.00682 |
| FAM81A | 0.750338 | 0.591655 | 0.951581 | 0.017819 |
| RARB | 1.421046 | 1.016746 | 1.986112 | 0.039668 |
| MMRN2 | 1.427202 | 1.115967 | 1.825239 | 0.004595 |
| RAI14 | 1.391474 | 1.114661 | 1.737031 | 0.00351 |
| SNCG | 1.331766 | 1.12553 | 1.575793 | 0.000845 |
| MFAP5 | 1.128123 | 1.001744 | 1.270446 | 0.046734 |
| ARHGEF15 | 1.426686 | 1.035865 | 1.96496 | 0.029577 |
| TGFBR1 | 1.476403 | 1.057401 | 2.061438 | 0.022156 |
| ASPN | 1.23036 | 1.05835 | 1.430327 | 0.006975 |
| NDN | 1.160608 | 1.013002 | 1.329722 | 0.031865 |
| HBB | 1.148409 | 1.027815 | 1.283154 | 0.0145 |
| NOTCH3 | 1.214052 | 1.002153 | 1.470755 | 0.047486 |
| CYP1B1 | 1.166617 | 1.045306 | 1.302006 | 0.005943 |
| TAP1 | 0.836576 | 0.700323 | 0.999337 | 0.049151 |
| HAS1 | 1.34626 | 1.022433 | 1.77265 | 0.034175 |
| LRRC32 | 1.254221 | 1.064691 | 1.47749 | 0.006731 |
| TGFB3 | 1.246251 | 1.046226 | 1.484518 | 0.013655 |
| RCAN1 | 1.452214 | 1.128975 | 1.868002 | 0.003681 |
| PLXDC2 | 1.29392 | 1.082342 | 1.546858 | 0.004676 |
| PDGFC | 1.259716 | 1.023517 | 1.550423 | 0.029304 |
| F13A1 | 1.180226 | 1.028989 | 1.353691 | 0.017865 |
| MGP | 1.122276 | 1.021068 | 1.233517 | 0.016741 |
| CYP7B1 | 1.406204 | 1.051839 | 1.879955 | 0.021385 |
| NUAK1 | 1.331851 | 1.044143 | 1.698836 | 0.021008 |
| THPO | 1.300236 | 1.03087 | 1.639986 | 0.026646 |
| HSPA12B | 1.28061 | 1.008324 | 1.626423 | 0.042568 |
| C19orf57 | 0.509417 | 0.326146 | 0.795673 | 0.003031 |
| TIE1 | 1.417206 | 1.072421 | 1.87284 | 0.014224 |
| LAMA2 | 1.322007 | 1.091043 | 1.601865 | 0.004381 |
| FIBIN | 1.365841 | 1.094542 | 1.704387 | 0.005788 |
| GHR | 1.311845 | 1.09857 | 1.566526 | 0.002714 |
| ELOVL2 | 1.494012 | 1.130418 | 1.974554 | 0.00478 |
| GLIS2 | 1.363204 | 1.044256 | 1.779571 | 0.022703 |
| FBLN2 | 1.140002 | 1.000852 | 1.298499 | 0.048521 |
| PECAM1 | 1.275707 | 1.047398 | 1.553782 | 0.01551 |
| ACYP1 | 0.724076 | 0.534026 | 0.981763 | 0.037668 |
| FZD4 | 1.303637 | 1.038489 | 1.636483 | 0.022284 |
| SIRPB2 | 1.789454 | 1.082163 | 2.959022 | 0.023349 |
| COL5A2 | 1.257951 | 1.066593 | 1.48364 | 0.006416 |
| ADAMTS10 | 1.454086 | 1.017449 | 2.078107 | 0.039888 |
| PJA2 | 1.391369 | 1.099589 | 1.760573 | 0.005949 |
| MEF2A | 1.395542 | 1.041291 | 1.870311 | 0.025695 |
| SSBP2 | 1.260286 | 1.01744 | 1.561096 | 0.034151 |
| GNB4 | 1.324812 | 1.049963 | 1.671608 | 0.017743 |
| KIT | 1.216116 | 1.039276 | 1.423047 | 0.014668 |
| RAMP3 | 1.254705 | 1.019174 | 1.544667 | 0.032435 |
| APOA1 | 1.094 | 1.013272 | 1.181159 | 0.021615 |
| SLCO2A1 | 1.29515 | 1.105395 | 1.51748 | 0.001376 |
| RASSF8 | 1.422917 | 1.114499 | 1.816685 | 0.00466 |
| FAM20A | 1.364907 | 1.024223 | 1.818913 | 0.033726 |
| FKBP7 | 1.842615 | 1.260953 | 2.692592 | 0.001588 |
| EXPH5 | 0.768212 | 0.600098 | 0.983422 | 0.036382 |
| GPRASP1 | 1.33116 | 1.005386 | 1.762494 | 0.045774 |
| ARMCX2 | 1.303956 | 1.09849 | 1.547853 | 0.002416 |
| FZD2 | 1.198556 | 1.005842 | 1.428192 | 0.042858 |
| PCOLCE2 | 1.306716 | 1.075323 | 1.587901 | 0.007139 |
| EFS | 1.383854 | 1.060462 | 1.805865 | 0.016746 |
| CHSY3 | 1.466557 | 1.094121 | 1.96577 | 0.010415 |
| CLIP3 | 1.153811 | 1.016544 | 1.309614 | 0.026837 |
| SERPINF1 | 1.213629 | 1.059916 | 1.389635 | 0.005077 |
| COL6A2 | 1.171426 | 1.014294 | 1.352901 | 0.031311 |
| HRH2 | 1.597985 | 1.025269 | 2.49062 | 0.038436 |
| GLIPR1 | 1.213349 | 1.03291 | 1.425309 | 0.018566 |
| DYNC1I1 | 1.277238 | 1.06219 | 1.535825 | 0.009286 |
| FABP4 | 1.163791 | 1.029328 | 1.315818 | 0.01546 |
| ITGA1 | 1.220622 | 1.003482 | 1.484749 | 0.046071 |
| P4HA3 | 1.776003 | 1.216763 | 2.592275 | 0.002913 |
| CCDC8 | 1.269849 | 1.021833 | 1.578063 | 0.03118 |
| A2M | 1.20415 | 1.032967 | 1.403701 | 0.01757 |
| CD34 | 1.320065 | 1.062993 | 1.639307 | 0.011979 |
| DDR2 | 1.210779 | 1.038511 | 1.411624 | 0.014584 |
| GAMT | 1.299675 | 1.117633 | 1.511368 | 0.000663 |
| FBXL7 | 1.372881 | 1.11132 | 1.696002 | 0.003296 |
| FNDC4 | 1.298412 | 1.009093 | 1.670683 | 0.042322 |
| SLCO4A1 | 0.832593 | 0.716724 | 0.967194 | 0.016564 |
| ARHGAP29 | 1.650696 | 1.130225 | 2.410843 | 0.009503 |
| GFRA1 | 1.287452 | 1.054885 | 1.571292 | 0.012933 |
| OLFML1 | 1.358397 | 1.103018 | 1.672902 | 0.003942 |
| MAN1C1 | 1.255191 | 1.020305 | 1.54415 | 0.031545 |
| EFNA3 | 0.767418 | 0.632047 | 0.931782 | 0.007505 |
| CYTL1 | 1.585345 | 1.223784 | 2.053729 | 0.000485 |
| COL8A1 | 1.161497 | 1.022058 | 1.319959 | 0.021771 |
| TCEAL7 | 1.375496 | 1.103424 | 1.714653 | 0.00458 |
| ANKRD22 | 0.851424 | 0.742926 | 0.975766 | 0.020739 |
| KIF15 | 0.803923 | 0.654899 | 0.986858 | 0.03694 |
| BCL6B | 1.34055 | 1.036474 | 1.733836 | 0.025556 |
| HP | 1.248626 | 1.017482 | 1.532279 | 0.033512 |
| PHYHD1 | 1.281833 | 1.017179 | 1.615344 | 0.03535 |
| ZNF367 | 0.64342 | 0.45957 | 0.90082 | 0.010219 |
| GPX7 | 1.307966 | 1.08899 | 1.570973 | 0.00408 |
| CALD1 | 1.139301 | 1.012467 | 1.282023 | 0.030332 |
| RUNX1T1 | 1.408425 | 1.015776 | 1.952854 | 0.039992 |
| CNTN4 | 1.70044 | 1.16053 | 2.491532 | 0.006454 |
| EFEMP2 | 1.228011 | 1.030349 | 1.463592 | 0.021798 |
| SORCS2 | 1.495541 | 1.141378 | 1.959599 | 0.003512 |
| FSTL1 | 1.262787 | 1.06698 | 1.494529 | 0.006645 |
| MATN3 | 1.434627 | 1.19121 | 1.727784 | 0.000142 |
| ERG | 1.753687 | 1.219803 | 2.521242 | 0.002424 |
| OGN | 1.105051 | 1.004957 | 1.215113 | 0.039204 |
| LRP1 | 1.301557 | 1.0316 | 1.642157 | 0.026264 |
| PCDH12 | 1.489346 | 1.105367 | 2.006712 | 0.008832 |
| PKIA | 1.33 | 1.059397 | 1.669723 | 0.014006 |
| LAMC1 | 1.3146 | 1.071995 | 1.612108 | 0.008592 |
| RAB31 | 1.272158 | 1.05811 | 1.529507 | 0.010441 |
| APOLD1 | 1.222075 | 1.020929 | 1.462852 | 0.028837 |
| LY96 | 1.237423 | 1.03742 | 1.475983 | 0.017865 |
| CD93 | 1.281628 | 1.057864 | 1.552723 | 0.011259 |
| TRO | 1.371785 | 1.069446 | 1.759597 | 0.012828 |
| DSE | 1.335175 | 1.040936 | 1.712585 | 0.022855 |
| ATP8B2 | 1.29835 | 1.037641 | 1.624561 | 0.022427 |
| UPK1B | 1.242179 | 1.086956 | 1.419569 | 0.001451 |
| DYSF | 1.277918 | 1.010396 | 1.616271 | 0.04073 |
| IL6 | 1.14753 | 1.012392 | 1.300708 | 0.031349 |
| LRRC26 | 0.865556 | 0.764815 | 0.979566 | 0.022197 |
| GUCY1A2 | 2.155234 | 1.40016 | 3.317505 | 0.000484 |
| EBF1 | 1.226245 | 1.005525 | 1.495415 | 0.043969 |
| PCDHGB7 | 1.562025 | 1.053699 | 2.315576 | 0.026393 |
| ADAMTS2 | 1.224292 | 1.010939 | 1.482671 | 0.038328 |
| C7 | 1.105617 | 1.016449 | 1.202607 | 0.01927 |
| TPST1 | 1.264718 | 1.029804 | 1.55322 | 0.025085 |
| COL10A1 | 1.123077 | 1.016242 | 1.241144 | 0.022854 |
| FRY | 1.522762 | 1.055839 | 2.196173 | 0.024399 |
| NLGN4Y | 1.381679 | 1.023448 | 1.8653 | 0.034744 |
| OLFML2B | 1.265913 | 1.081687 | 1.481515 | 0.003298 |
| VCAN | 1.311341 | 1.11942 | 1.536166 | 0.000787 |
| MMRN1 | 1.293452 | 1.087735 | 1.538074 | 0.003597 |
| PLTP | 1.252358 | 1.070577 | 1.465004 | 0.00492 |
| HEG1 | 1.281609 | 1.038693 | 1.581334 | 0.020666 |
| SGCE | 1.275062 | 1.097235 | 1.48171 | 0.00152 |
| PDGFD | 1.317028 | 1.067206 | 1.625331 | 0.010286 |
| FOXS1 | 1.305598 | 1.059614 | 1.608686 | 0.012293 |
| RAMP2 | 1.293875 | 1.044272 | 1.603139 | 0.018467 |
| EDIL3 | 1.283994 | 1.002677 | 1.64424 | 0.047574 |
| ASGR2 | 1.274731 | 1.016432 | 1.59867 | 0.035638 |
| SVEP1 | 1.206038 | 1.055448 | 1.378115 | 0.005906 |
| LPL | 1.239227 | 1.049133 | 1.463765 | 0.011587 |
| JAZF1 | 1.351508 | 1.073981 | 1.700749 | 0.010212 |
| PRICKLE1 | 1.404069 | 1.17326 | 1.680286 | 0.000212 |
| THBS2 | 1.14681 | 1.022384 | 1.286379 | 0.0194 |
| TCF4 | 1.277728 | 1.028457 | 1.587416 | 0.026872 |
| ADRA1B | 1.50289 | 1.173944 | 1.924007 | 0.001228 |
| HSPB8 | 1.121457 | 1.010166 | 1.24501 | 0.031583 |
| PDLIM3 | 1.114586 | 1.004547 | 1.236679 | 0.040805 |
| PHYHIP | 1.905332 | 1.227456 | 2.957571 | 0.00406 |
| SETBP1 | 1.176883 | 1.003441 | 1.380303 | 0.045264 |
| FLT1 | 1.524796 | 1.072676 | 2.16748 | 0.018727 |
| TMEM71 | 1.352485 | 1.014057 | 1.80386 | 0.039882 |
| GPC3 | 1.16878 | 1.061673 | 1.286694 | 0.001471 |
| CDH2 | 1.2513 | 1.058965 | 1.478568 | 0.008468 |
| FNDC1 | 1.164249 | 1.047667 | 1.293804 | 0.004728 |
| RGS7BP | 1.601642 | 1.121688 | 2.286962 | 0.009546 |
| RNF150 | 1.14915 | 1.005203 | 1.313711 | 0.041754 |
| BGN | 1.228288 | 1.054306 | 1.43098 | 0.008326 |
| MOCS1 | 1.422472 | 1.07962 | 1.874204 | 0.012266 |
| PDE1A | 1.354117 | 1.104871 | 1.65959 | 0.003491 |
| COL5A1 | 1.179278 | 1.015273 | 1.369776 | 0.0309 |
| SLIT3 | 1.19682 | 1.021291 | 1.402516 | 0.026397 |
| MEOX2 | 1.300931 | 1.075469 | 1.57366 | 0.006744 |
| SLC1A7 | 1.310001 | 1.037148 | 1.654636 | 0.023448 |
| PAPPA | 1.268584 | 1.061272 | 1.516393 | 0.00897 |
| PCDH18 | 1.300028 | 1.057392 | 1.598342 | 0.012795 |
| IGSF21 | 1.47697 | 1.060179 | 2.057615 | 0.021143 |
| PTPRM | 1.333659 | 1.077566 | 1.650616 | 0.008129 |
| PLCL1 | 1.951049 | 1.34046 | 2.839764 | 0.000483 |
| ADAMTS12 | 1.41394 | 1.098047 | 1.820711 | 0.007253 |
| DZIP1 | 1.45529 | 1.043683 | 2.029225 | 0.026964 |
| ZNF331 | 1.429335 | 1.118137 | 1.827147 | 0.004355 |
| ST6GALNAC3 | 1.834385 | 1.252287 | 2.687058 | 0.001839 |
| PCDHB5 | 1.315192 | 1.103995 | 1.566792 | 0.002157 |
| STEAP4 | 1.372875 | 1.094642 | 1.721828 | 0.006097 |
| IGFBP6 | 1.179671 | 1.018753 | 1.366007 | 0.027226 |
| ZFPM2 | 1.39308 | 1.054373 | 1.840592 | 0.019675 |
| RGS18 | 1.447175 | 1.095505 | 1.911734 | 0.009265 |
| ADAM12 | 1.359994 | 1.088294 | 1.699525 | 0.00685 |
| PLVAP | 1.302135 | 1.055816 | 1.60592 | 0.013601 |
| STOM | 1.286051 | 1.052794 | 1.570988 | 0.013747 |
| FGF7 | 1.260562 | 1.043509 | 1.522763 | 0.016319 |
| CMTM3 | 1.341615 | 1.086481 | 1.656661 | 0.00632 |
| PDGFRL | 1.304678 | 1.131175 | 1.504793 | 0.000259 |
| MPO | 1.349246 | 1.031682 | 1.764558 | 0.028686 |
| MS4A4A | 1.327505 | 1.071717 | 1.644343 | 0.009481 |
| SLC22A17 | 1.277517 | 1.057385 | 1.543477 | 0.011141 |
| CLRN3 | 0.902743 | 0.82063 | 0.993071 | 0.035478 |
| LRRC17 | 1.335884 | 1.061312 | 1.681492 | 0.013631 |
| PVT1 | 0.751504 | 0.590529 | 0.95636 | 0.020192 |
| PRRX1 | 1.213139 | 1.034266 | 1.422949 | 0.0176 |
| CYYR1 | 1.308951 | 1.061764 | 1.613684 | 0.011695 |
| DNAJC18 | 1.471941 | 1.013608 | 2.137523 | 0.042258 |
| AKT3 | 1.420715 | 1.096075 | 1.841508 | 0.007977 |
| CPNE8 | 1.736628 | 1.344823 | 2.242582 | 2.33E-05 |
| ITGB4 | 0.847192 | 0.724365 | 0.990846 | 0.037983 |
| RCBTB2 | 1.395622 | 1.009106 | 1.930185 | 0.043931 |
| GAD1 | 0.762425 | 0.615485 | 0.944446 | 0.01302 |
| ADAMTS1 | 1.277875 | 1.086048 | 1.503586 | 0.003131 |
| HMCN1 | 1.361251 | 1.04529 | 1.772717 | 0.022098 |
| CCDC80 | 1.182413 | 1.029153 | 1.358496 | 0.017997 |
| PTGFR | 1.430675 | 1.064457 | 1.922888 | 0.017596 |
| FBLN5 | 1.2422 | 1.052606 | 1.465943 | 0.010267 |
| DOK5 | 1.564935 | 1.071178 | 2.28629 | 0.020588 |
| MAGEE1 | 1.376905 | 1.034271 | 1.833048 | 0.028468 |
| AMOTL1 | 1.291055 | 1.024383 | 1.627148 | 0.030461 |
| CERCAM | 1.205218 | 1.013017 | 1.433885 | 0.035216 |
| GLI1 | 1.415609 | 1.077798 | 1.8593 | 0.01247 |
| EFNB3 | 1.342513 | 1.035466 | 1.74061 | 0.026216 |
| SCUBE2 | 1.2973 | 1.034284 | 1.627199 | 0.02435 |
| BASP1 | 1.266576 | 1.079259 | 1.486405 | 0.003803 |
| ADAMTS15 | 1.367835 | 1.003712 | 1.864053 | 0.04732 |
| CLEC14A | 1.252146 | 1.023711 | 1.531555 | 0.02867 |
| GPX3 | 1.301924 | 1.128553 | 1.501929 | 0.000296 |
| TLR7 | 1.298561 | 1.060227 | 1.590473 | 0.011562 |
| SFRP2 | 1.103665 | 1.020072 | 1.194109 | 0.014108 |
| KLF7 | 1.446563 | 1.067984 | 1.95934 | 0.017087 |
| RASGRF2 | 2.066665 | 1.285574 | 3.322333 | 0.002726 |
| TACC1 | 1.310124 | 1.080862 | 1.588016 | 0.005919 |
| ADH4 | 1.149445 | 1.03336 | 1.27857 | 0.010344 |
| EDA | 1.413094 | 1.014045 | 1.969178 | 0.041117 |
| EDNRB | 1.416223 | 1.087247 | 1.84474 | 0.009875 |
| FRMD6 | 1.346232 | 1.108237 | 1.635336 | 0.002741 |
| FHL5 | 1.477715 | 1.100142 | 1.984872 | 0.009489 |
| ACTA2 | 1.142369 | 1.016111 | 1.284316 | 0.025919 |
| SFRP1 | 1.105682 | 1.003174 | 1.218666 | 0.042991 |
| CREB3L3 | 1.165564 | 1.030403 | 1.318455 | 0.014842 |
| RNF180 | 1.610689 | 1.05059 | 2.469391 | 0.028791 |
| ABCA1 | 1.443058 | 1.13494 | 1.834825 | 0.002764 |
| TNN | 1.34074 | 1.027548 | 1.749391 | 0.03076 |
| GLI3 | 1.297188 | 1.013214 | 1.660751 | 0.039008 |
| IGFBP7 | 1.271116 | 1.084896 | 1.4893 | 0.002996 |
| AASS | 1.475668 | 1.009454 | 2.157201 | 0.044586 |
| COL6A3 | 1.176304 | 1.012791 | 1.366215 | 0.033469 |
| LBH | 1.441401 | 1.136337 | 1.828364 | 0.002584 |
| POF1B | 0.846624 | 0.722599 | 0.991937 | 0.039386 |
| CC2D2A | 1.46535 | 1.123355 | 1.911464 | 0.004836 |
| GAS1 | 1.149746 | 1.018736 | 1.297605 | 0.023778 |
| LARP6 | 1.263344 | 1.046297 | 1.525415 | 0.015077 |
| CDO1 | 1.342651 | 1.074765 | 1.677308 | 0.00946 |
| HSD11B1 | 1.218877 | 1.002424 | 1.482068 | 0.04723 |
| PDK4 | 1.132104 | 1.019806 | 1.256768 | 0.019916 |
| GADD45B | 1.293785 | 1.040097 | 1.60935 | 0.020722 |
| PCSK1 | 1.172454 | 1.007586 | 1.364298 | 0.039618 |
| EMCN | 1.338372 | 1.069091 | 1.675479 | 0.010995 |
| PTCH2 | 1.382382 | 1.075941 | 1.776103 | 0.011328 |
| CNTN1 | 1.407861 | 1.137194 | 1.742949 | 0.001689 |
| GPRASP2 | 1.376124 | 1.046355 | 1.809822 | 0.022363 |
| MFGE8 | 1.254658 | 1.029689 | 1.528779 | 0.02444 |
| KLHL13 | 1.436394 | 1.118987 | 1.843833 | 0.004478 |
| INHBA | 1.297405 | 1.051344 | 1.601056 | 0.015241 |
| NOX4 | 1.457394 | 1.163149 | 1.826075 | 0.001063 |
| F2RL3 | 1.265967 | 1.006791 | 1.591862 | 0.043604 |
| CD302 | 1.255983 | 1.022935 | 1.542126 | 0.029517 |
| MOXD1 | 1.21655 | 1.038539 | 1.425073 | 0.015164 |
| FRZB | 1.139086 | 1.007648 | 1.287668 | 0.037364 |
| PDPN | 1.195295 | 1.009636 | 1.415096 | 0.03833 |
| SRPX2 | 1.376006 | 1.133313 | 1.670671 | 0.001264 |
| TMEM88 | 1.275331 | 1.028114 | 1.581993 | 0.026956 |
| HIGD1B | 1.952541 | 1.283672 | 2.969931 | 0.001766 |
| STAB1 | 1.251486 | 1.019737 | 1.535904 | 0.031791 |
| RBP7 | 1.289105 | 1.047071 | 1.587086 | 0.016689 |
| SPARCL1 | 1.129661 | 1.018898 | 1.252464 | 0.020583 |
| LAMA4 | 1.32883 | 1.090618 | 1.619072 | 0.004794 |
| VIM | 1.368114 | 1.125637 | 1.662824 | 0.001638 |
| GRASP | 1.314152 | 1.001528 | 1.724361 | 0.048726 |
| SERPINE1 | 1.31692 | 1.158389 | 1.497145 | 2.59E-05 |
| NPR3 | 1.645304 | 1.211907 | 2.233691 | 0.001412 |
| SDC2 | 1.469403 | 1.174089 | 1.838995 | 0.000774 |
| CST2 | 1.212784 | 1.055871 | 1.393016 | 0.006352 |
| MCC | 1.648456 | 1.197892 | 2.268492 | 0.002152 |
| TSC22D3 | 1.20786 | 1.01755 | 1.433763 | 0.030862 |
| TUBB6 | 1.235457 | 1.031536 | 1.479692 | 0.021601 |
| ARHGAP10 | 1.289442 | 1.029195 | 1.615496 | 0.027094 |
| PTGER3 | 1.409244 | 1.027218 | 1.933348 | 0.033468 |
| NR4A3 | 1.296907 | 1.023878 | 1.642741 | 0.031113 |
| CORO2B | 1.476511 | 1.047981 | 2.08027 | 0.025887 |
| CTF1 | 1.224416 | 1.009519 | 1.485058 | 0.039765 |
| FERMT2 | 1.198603 | 1.043926 | 1.3762 | 0.010177 |
| LBP | 1.191925 | 1.035189 | 1.372392 | 0.014657 |
| LUM | 1.217054 | 1.066298 | 1.389125 | 0.003598 |
| COL15A1 | 1.202847 | 1.028174 | 1.407195 | 0.021053 |
| PCDHGA12 | 1.771642 | 1.145641 | 2.739704 | 0.010133 |
| COL1A2 | 1.180834 | 1.034405 | 1.347992 | 0.013866 |
| ADAM23 | 1.276475 | 1.002235 | 1.625755 | 0.047922 |
| TSPAN7 | 1.176763 | 1.011802 | 1.368619 | 0.034668 |
| CIDEC | 1.17787 | 1.02264 | 1.356663 | 0.02318 |
| SMARCD3 | 1.404031 | 1.071759 | 1.839314 | 0.01378 |
| TSHZ3 | 1.239296 | 1.021834 | 1.503038 | 0.029304 |
| CD200 | 1.522694 | 1.122335 | 2.065869 | 0.006904 |
| F8 | 1.691441 | 1.109202 | 2.579307 | 0.014631 |
| SIGLEC6 | 1.430232 | 1.042106 | 1.962913 | 0.02674 |
| AXL | 1.369172 | 1.132677 | 1.655047 | 0.001164 |
| LZTS1 | 1.291851 | 1.036988 | 1.609351 | 0.022377 |
| VWF | 1.309795 | 1.093961 | 1.568212 | 0.003309 |
| PDE9A | 1.320854 | 1.048507 | 1.663942 | 0.018176 |
| PYY | 1.226856 | 1.026346 | 1.466538 | 0.024731 |
| PHLDB2 | 1.311558 | 1.097899 | 1.566797 | 0.002795 |
| HTRA1 | 1.255821 | 1.061403 | 1.48585 | 0.007946 |
| FLRT2 | 1.39316 | 1.137783 | 1.705856 | 0.00133 |
| MXRA8 | 1.23316 | 1.04976 | 1.448601 | 0.010739 |
| FREM1 | 1.295123 | 1.041774 | 1.610084 | 0.019889 |
| ST3GAL6 | 1.596446 | 1.177451 | 2.164539 | 0.002598 |
| TM6SF1 | 1.393101 | 1.038454 | 1.868864 | 0.026988 |
| ENOX1 | 1.344434 | 1.041903 | 1.734809 | 0.022872 |
| PDE7B | 1.204079 | 1.009921 | 1.435564 | 0.038453 |
| LAMB1 | 1.275808 | 1.019536 | 1.596498 | 0.033248 |
| MTTP | 1.135719 | 1.004268 | 1.284375 | 0.042578 |
| ABCA8 | 1.22647 | 1.057006 | 1.423103 | 0.00713 |
| ITGBL1 | 1.22411 | 1.008724 | 1.485486 | 0.040566 |
| SLC2A3 | 1.307268 | 1.09705 | 1.557768 | 0.00274 |
| ZDHHC2 | 1.254475 | 1.029033 | 1.529307 | 0.024889 |
| F2R | 1.442803 | 1.117047 | 1.863557 | 0.004989 |
| THBS1 | 1.217968 | 1.070918 | 1.38521 | 0.002668 |
| CRISPLD2 | 1.215549 | 1.037193 | 1.424577 | 0.015908 |
| MYO5A | 1.479337 | 1.127276 | 1.94135 | 0.004744 |
| GNG11 | 1.327029 | 1.115479 | 1.5787 | 0.001406 |
| DNAJB4 | 1.276318 | 1.008581 | 1.615128 | 0.042245 |
| BNC2 | 1.210842 | 1.013198 | 1.44704 | 0.035363 |
| FABP3 | 1.210877 | 1.006981 | 1.456058 | 0.041961 |
| ZBTB10 | 1.456394 | 1.087671 | 1.950114 | 0.011596 |
| SSPN | 1.22277 | 1.046671 | 1.428497 | 0.011248 |
| ELANE | 1.268299 | 1.045605 | 1.538424 | 0.015837 |
| PDE2A | 1.353895 | 1.054939 | 1.737571 | 0.017308 |
| LHX6 | 1.67477 | 1.204573 | 2.328504 | 0.002163 |
| ASCL2 | 0.889555 | 0.809856 | 0.977097 | 0.014535 |
| CHI3L2 | 1.217558 | 1.014383 | 1.461429 | 0.034577 |
| CACNA2D1 | 1.278371 | 1.014725 | 1.610517 | 0.03716 |
| CLGN | 1.487845 | 1.092676 | 2.025927 | 0.011646 |
| STON1 | 1.169773 | 1.002175 | 1.3654 | 0.046868 |
| GPR15 | 1.357218 | 1.033208 | 1.782836 | 0.028185 |
| PLA2G5 | 1.468867 | 1.024924 | 2.105103 | 0.036256 |
| ENG | 1.310485 | 1.055489 | 1.627084 | 0.014321 |
| GPC6 | 1.296187 | 1.036948 | 1.620237 | 0.022689 |
| PI15 | 1.209307 | 1.018866 | 1.435344 | 0.029725 |
| HEYL | 1.245518 | 1.066919 | 1.454013 | 0.005432 |
| SLC9A9 | 1.297263 | 1.031622 | 1.631306 | 0.025996 |
| OLFML3 | 1.193025 | 1.019761 | 1.395729 | 0.027499 |
| SLC1A5 | 0.819242 | 0.684251 | 0.980865 | 0.029986 |
| THSD7A | 1.765316 | 1.265644 | 2.462257 | 0.000815 |
| NPR1 | 1.395404 | 1.094667 | 1.778761 | 0.007139 |
| RCAN2 | 1.1558 | 1.003191 | 1.331625 | 0.045063 |
| CH25H | 1.193537 | 1.021207 | 1.394948 | 0.026167 |
| AFAP1L1 | 1.69244 | 1.217568 | 2.35252 | 0.001739 |
| DLC1 | 1.327781 | 1.087025 | 1.62186 | 0.005479 |
| HTRA3 | 1.311742 | 1.088812 | 1.580316 | 0.0043 |
| COLEC11 | 1.432567 | 1.081131 | 1.898242 | 0.012309 |
| HABP2 | 1.15422 | 1.043674 | 1.276476 | 0.005236 |
| NFATC4 | 1.360162 | 1.000276 | 1.849531 | 0.049795 |
| PCDHB4 | 1.445884 | 1.092104 | 1.91427 | 0.010014 |
| GEM | 1.233011 | 1.04504 | 1.454791 | 0.013065 |
| SELL | 1.179005 | 1.010315 | 1.375861 | 0.036597 |
| NID1 | 1.343771 | 1.076879 | 1.676808 | 0.008907 |
| CLIP4 | 1.36831 | 1.081704 | 1.730853 | 0.008926 |
| VGLL3 | 1.425547 | 1.143478 | 1.777196 | 0.001623 |
| CD36 | 1.355608 | 1.136116 | 1.617504 | 0.000735 |
| THY1 | 1.238015 | 1.052991 | 1.455551 | 0.009734 |
| DPYSL4 | 1.304638 | 1.018491 | 1.671179 | 0.035292 |
| PYGO1 | 1.679165 | 1.180766 | 2.387937 | 0.003916 |
| PCDH7 | 1.192182 | 1.023032 | 1.389301 | 0.024346 |
| COL4A5 | 1.156309 | 1.001758 | 1.334704 | 0.04726 |
| TLR1 | 1.35061 | 1.043337 | 1.748379 | 0.022484 |
| CCDC3 | 1.265435 | 1.034756 | 1.54754 | 0.021865 |
| ECM2 | 1.340626 | 1.100205 | 1.633585 | 0.003649 |
| GPR4 | 1.393104 | 1.063533 | 1.824802 | 0.016075 |
| MID2 | 1.403616 | 1.087649 | 1.811374 | 0.00917 |
| STC1 | 1.354107 | 1.129216 | 1.623786 | 0.00107 |
| DOK7 | 0.675239 | 0.502352 | 0.907627 | 0.009262 |
| FZD7 | 1.340073 | 1.088299 | 1.650093 | 0.005836 |
| ELOVL4 | 1.397425 | 1.114506 | 1.752162 | 0.003741 |
| MAMLD1 | 1.320674 | 1.009103 | 1.728446 | 0.042768 |
| ARMCX1 | 1.344653 | 1.118903 | 1.615951 | 0.001588 |
| MDFIC | 1.326748 | 1.043485 | 1.686905 | 0.021036 |
| PLXDC1 | 1.625351 | 1.144599 | 2.308026 | 0.006631 |
| SNAI2 | 1.294366 | 1.083075 | 1.546877 | 0.004545 |
| TIMP2 | 1.305578 | 1.100167 | 1.54934 | 0.002266 |
| MAPK10 | 1.573233 | 1.09637 | 2.257506 | 0.013921 |
| APOD | 1.146887 | 1.060935 | 1.239801 | 0.000564 |
| CLDN6 | 1.145482 | 1.016899 | 1.290324 | 0.025364 |
| COLEC12 | 1.281926 | 1.072383 | 1.532414 | 0.006384 |
| FMOD | 1.15359 | 1.00093 | 1.329534 | 0.048519 |
| CD248 | 1.193938 | 1.009028 | 1.412735 | 0.038956 |
| CDC25A | 0.775959 | 0.621334 | 0.969063 | 0.025279 |
| PRSS23 | 1.245962 | 1.05944 | 1.465323 | 0.007865 |
| LRCH2 | 1.452527 | 1.117812 | 1.887469 | 0.005217 |
| COL14A1 | 1.166917 | 1.0253 | 1.328095 | 0.019363 |
| FAM49A | 1.384678 | 1.030309 | 1.860931 | 0.030933 |
| COL4A1 | 1.304627 | 1.057994 | 1.608753 | 0.012873 |
| FN1 | 1.208154 | 1.035584 | 1.40948 | 0.01619 |
| CPVL | 1.229241 | 1.061568 | 1.423397 | 0.005807 |
| CYR61 | 1.233655 | 1.077238 | 1.412784 | 0.002401 |
| CDH11 | 1.271446 | 1.078189 | 1.499343 | 0.004305 |
| FBN2 | 1.309636 | 1.047793 | 1.636913 | 0.01778 |
| GABARAPL1 | 1.260753 | 1.008103 | 1.576722 | 0.042286 |
| ADPRH | 1.270068 | 1.000868 | 1.611674 | 0.049172 |
| CARTPT | 1.177046 | 1.008925 | 1.373182 | 0.038177 |
| CFH | 1.210649 | 1.043745 | 1.404242 | 0.011548 |
| RAB34 | 1.289543 | 1.09867 | 1.513576 | 0.001862 |
| RECK | 1.421273 | 1.142682 | 1.767786 | 0.001588 |
| SLC45A1 | 1.711205 | 1.072423 | 2.730472 | 0.024244 |
| ATP1B2 | 1.345146 | 1.048726 | 1.725349 | 0.019566 |
| MANEAL | 0.771246 | 0.622524 | 0.955499 | 0.01748 |
| PDE3A | 1.29706 | 1.037921 | 1.620898 | 0.02218 |
| TRIM15 | 0.856107 | 0.736782 | 0.994757 | 0.042499 |
| ANGPT1 | 1.306559 | 1.024602 | 1.666106 | 0.03109 |
| CHST7 | 1.342381 | 1.027879 | 1.753112 | 0.03063 |
| PDE8B | 1.378396 | 1.047036 | 1.814623 | 0.022161 |
| AGT | 1.15307 | 1.028579 | 1.292629 | 0.014551 |
| FLT4 | 1.726043 | 1.127599 | 2.642096 | 0.011977 |
| PRKG1 | 1.335671 | 1.010688 | 1.765153 | 0.041881 |
| RFTN2 | 1.486792 | 1.098301 | 2.0127 | 0.010265 |
| TNFAIP8L3 | 1.351671 | 1.079208 | 1.69292 | 0.0087 |
| BST1 | 1.477936 | 1.116775 | 1.955895 | 0.006285 |
| ADAMTS5 | 1.603856 | 1.162133 | 2.213476 | 0.004052 |
| OLFML2A | 1.254666 | 1.017754 | 1.546727 | 0.033604 |
| BEX4 | 1.233362 | 1.061399 | 1.433185 | 0.006186 |
| PLSCR4 | 1.23954 | 1.033483 | 1.486681 | 0.020612 |
| QKI | 1.688706 | 1.202205 | 2.372081 | 0.00251 |
| PKD2 | 1.276655 | 1.034904 | 1.574878 | 0.022592 |
| CHRD | 1.242326 | 1.047258 | 1.473729 | 0.012781 |
| FPR2 | 1.322839 | 1.003367 | 1.744031 | 0.047278 |
| EGFLAM | 1.707435 | 1.269301 | 2.296802 | 0.000406 |
| RERG | 1.166844 | 1.022496 | 1.33157 | 0.022012 |
| FEZ1 | 1.291236 | 1.028294 | 1.621414 | 0.027798 |
| TUBA1A | 1.247278 | 1.03119 | 1.508647 | 0.022823 |
| CLIC2 | 1.327693 | 1.032128 | 1.707897 | 0.027378 |
| OPN1SW | 1.711086 | 1.13965 | 2.569047 | 0.009587 |
| CPE | 1.200219 | 1.062463 | 1.355837 | 0.003346 |
| MMP2 | 1.202938 | 1.025888 | 1.410544 | 0.02293 |
| SFRP4 | 1.09738 | 1.002268 | 1.201518 | 0.044543 |
| CNTNAP1 | 1.260935 | 1.049161 | 1.515455 | 0.013453 |
| CTSK | 1.229137 | 1.058445 | 1.427354 | 0.006839 |
| ANKRD6 | 1.480512 | 1.1039 | 1.985609 | 0.008793 |

HR: Hazard ratio.
